# Supplementary material for: [3+3]-Annulation of Cyclic Nitronates with Vinyl Diazoacetates: Diastereoselective Synthesis of Partially Saturated [1,2]Oxazino[2,3-b][1,2]oxazines and Their Base-Promoted Ring Contraction to Pyrrolo[1,2-b][1,2]oxazine Derivatives
Source: Molecules. 2023 Mar 28;28(7):3025. doi: 10.3390/molecules28073025 (PMC10096057; doi:10.3390/molecules28073025)

Supplementary materials

[3+3]-Annulation of Cyclic Nitronates with Vinyl Diazoacetates: Diastereoselective Synthesis of Partially Saturated [1,2]Oxazino[2,3-*b*][1,2]oxazines and Their Base-Promoted Ring Contraction to Pyrrolo[1,2-*b*][1,2]oxazine Derivatives

List of starting nitronates 1 and vinyl diazoacetates 2

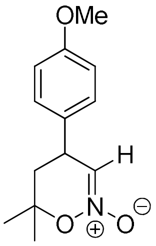
1a

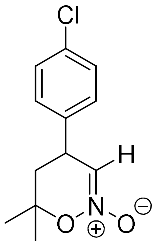
1b

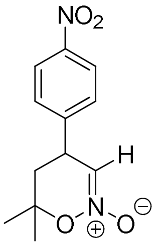
1c

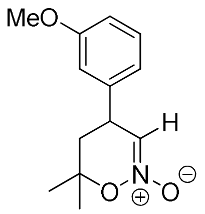
1d

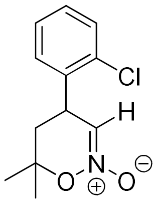
1e

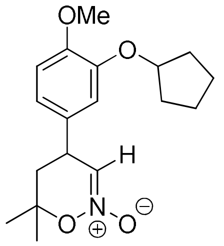
1f

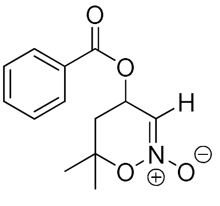
1g

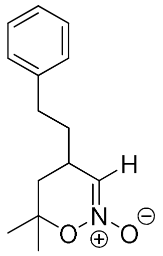
1h

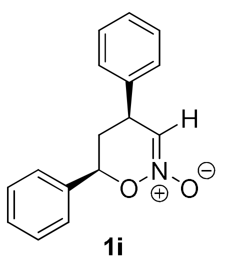
1i

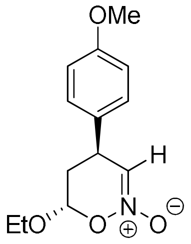
1j

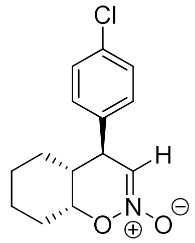
1k

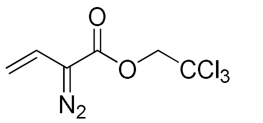
2a

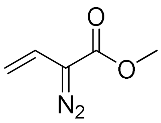
2b

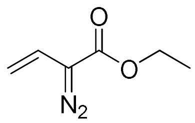
2c

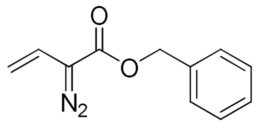
2d

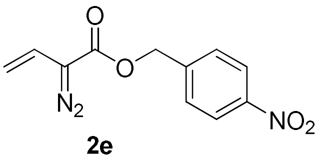
2e

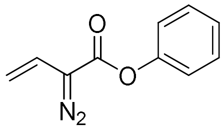
2f

Starting nitronates **1a**,**b**,**g-j** were prepared according to the literature procedures: **1a** [s1], **1b** [s2], **1g** [s3], **1h** [s4], **1i** [s4], **1j** [s3].
Starting vinyl diazoacetates **2b-d** were prepared according to the literature procedures: **2b** [s5], **2c** [s6], **2d** [s6].

X-ray crystallography

Table S1. Crystal data and structure refinement parameters for **3b, 3p, and 4b**

	**3b**	**3p**	**4b**
Empirical formula	$C_{18}H_{23}NO_5$	$C_{20}H_{21}Cl_4NO_4$	$C_{18}H_{21}NO_4$
Formula weight	333.37	481.18	315.36
T, K	100	100	100
Crystal system	Triclinic	Monoclinic	Orthorhombic
Space group	P-1	$P2_1/c$	$P2_12_12_1$
Z	2	4	4
a, Å	6.2326(2)	14.6132(3)	9.4015(3)
b, Å	11.4806(3)	11.9428(2)	10.3300(3)
c, Å	12.8224(3)	12.2860(2)	16.9651(4)
α, °	112.9640(10)	90	90
β, °	93.655(2)	93.8750(10)	90
γ, °	100.291(2)	90	90
V, $Å^3$	822.12(4)	2139.28(7)	1647.61(8)
D_{calc} (g cm^{-1})	1.347	1.494	1.271
Linear absorption, μ (cm^{-1})	0.98	5.81	0.9
F(000)	356	992	672
$2\theta_{max}$, °	58	58	56
Reflections measured	11065	27809	20562
Independent reflections	4366	5680	3988
Observed reflections [$I > 2\sigma(I)$]	3329	5377	3688
Parameters	221	262	214
R1	0.0443	0.0287	0.0334
wR2	0.1108	0.0748	0.0796
GOF	1.026	1.021	1.046
$\Delta\rho_{max}$/ $\Delta\rho_{min}$ (e $Å^{-3}$)	0.343/-0.273	0.779/-0.582	0.284/-0.215

Preparation of starting compounds

6,6-Dimethyl-4-(4-nitrophenyl)-5,6-dihydro-4H-1,2-oxazine 2-oxide 1c

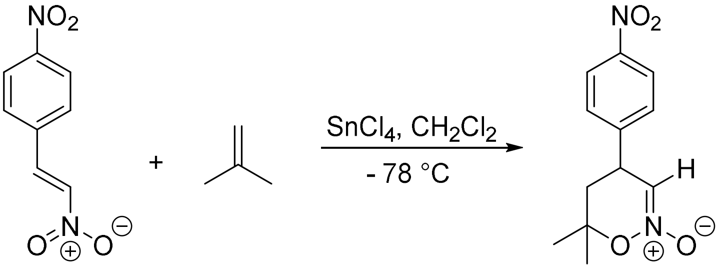

Starting nitroalkene was prepared according to the literature procedure [s7].
To a solution of (*E*)-1-nitro-4-(2-nitrovinyl)benzene (290 mg, 1.49 mmol) in freshly distilled CH_2Cl_2 (14 mL) $SnCl_4$ (0.21 ml, 0.47 g, 1.80 mmol) was added at -78 °C under argon atmosphere. The resulting solution was stirred for 5 min and isobutylene (ca. 0.80 g, 14.3 mmol) was added. The reaction mixture was warmed up to -20 °C and maintained at the same temperature overnight. The mixture poured into EtOAc (130 mL) / $NaHCO_3$ (sat. aq., 100 mL). Organic layer was washed with H_2O (100 mL), brine (100 mL), dried over Na_2SO_4 and evaporated. Column chromatography (eluent: PE/EtOAc, 3:1, then 1:2) afforded 105 mg (28%) of target nitronate as light yellow powder.
R_f = 0.29 (EtOAc, UV, anisaldehyde).
mp = 97-99 °C (THF).
^{1}H NMR (300 MHz, $CDCl_3$): δ 1.48 (s, 3H, Me(6)), 1.54 (s, 3H, Me(6)), 1.85 (dd, *J* = 13.7, 10.9 Hz, 1H, CH_{2ax}(5)), 2.11 (dd, *J* = 13.7, 7.4 Hz, 1H, CH_{2eq}(5)), 4.00 (ddd, *J* = 10.9, 7.4, 2.9 Hz, 1H, CH(4)), 6.42 (d, *J* = 2.9 Hz, 1H, CH(3)=N), 7.45 (d, *J* = 8.7 Hz, 2H, CH_{Ar}), 8.23 (d, *J* = 8.7 Hz, 2H, CH_{Ar}).
^{13}C NMR (75 MHz, DEPT, $CDCl_3$): δ 22.6 (Me(6)), 22.7 (Me(6)), 38.6 (CH(4)), 39.1 (CH_2(5)), 82.7 (C(6)), 111.6 (CH(3)=N), 124.5 (CH_{Ar}), 128.6 (CH_{Ar}), 147.2 (C_{Ar}), 147.5 (C_{Ar}).
HRMS (ESI): *m/z* calcd. for $[C_{12}H_{14}N_2O_4+H^+]$: 251.1026, found: 251.1031.

4-(3-Methoxyphenyl)-6,6-dimethyl-5,6-dihydro-4H-1,2-oxazine 2-oxide 1d

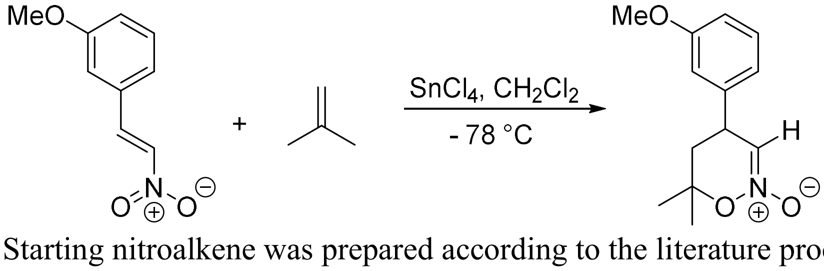

Starting nitroalkene was prepared according to the literature procedure [s8].
To a solution of (*E*)-1-methoxy-3-(2-nitrovinyl)benzene (278 mg, 1.55 mmol) in freshly distilled CH_2Cl_2 (15 mL) $SnCl_4$ (0.22 ml, 0.48 g, 1.86 mmol) was added at -78 °C under argon atmosphere. The resulting solution was stirred for 5 min and isobutylene (ca. 0.40 g, 7.1 mmol) was added. The reaction mixture was stirred for 15 min at -78 °C and poured into EtOAc (130 mL) / $NaHCO_3$ (sat. aq., 100 mL). Organic layer was washed with H_2O (100 mL), brine (100 mL), dried over Na_2SO_4 and evaporated. Crystallization of the residue from PE/EtOAc (10:1) afforded 233 mg (64%) of target nitronate as brownish powder.
mp = 80-82 °C (PE/EtOAc, 3:1).
^{1}H NMR (300 MHz, COSY, $CDCl_3$): δ 1.46 (s, 3H, Me(6)), 1.52 (s, 3H, Me(6)), 1.86 (dd, *J* = 13.8, 11.6 Hz, 1H, CH_{2ax}(5)), 2.11 (dd, *J* = 13.8, 7.3 Hz, 1H, CH_{2eq}(5)), 3.81 (s, 3H, OMe), 3.77-3.84 (m, overlapped, 1H, CH(4)), 6.43 (d, *J* = 3.0 Hz, 1H, CH(3)=N), 6.76 (s, 1H, CH_{Ar}), 6.81-6.86 (m, 2H, CH_{Ar}), 7.26-7.31 (m, 1H, CH_{Ar}).
^{13}C NMR (75 MHz, DEPT, HSQC, $CDCl_3$): δ 22.7 (Me(6)), 27.8 (Me(6)), 38.8 (CH(4)), 39.3 (CH_2(5)), 55.3 (OMe), 82.6 (C(6)), 113.2 and 113.5 (2×CH_{Ar} and CH(3)=N), 119.8 (CH_{Ar}), 130.2 (CH_{Ar}), 141.3 (C_{Ar}), 160.2 ($\underline{C}_{Ar}$–OMe).

HRMS (ESI): *m/z* calcd. for [$C_{13}H_{17}NO_3$+H^+]: 236.1281, found: 236.1277.

4-(2-Chlorophenyl)-6,6-dimethyl-5,6-dihydro-4H-1,2-oxazine 2-oxide 1e

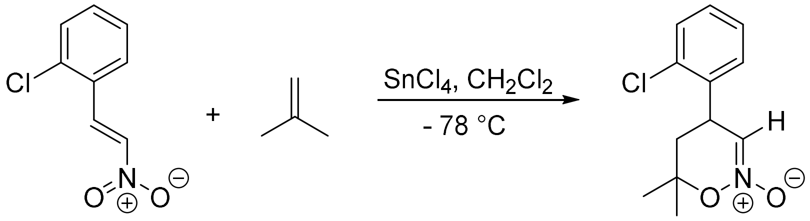

Starting nitroalkene was prepared according to the literature procedure [s9].
To a solution of (*E*)-1-chloro-2-(2-nitrovinyl)benzene (0.37 g, 2.02 mmol) in freshly distilled CH_2Cl_2 (13 mL) $SnCl_4$ (0.28 ml, 0.62 g, 2.40 mmol) was added at -78 °C under argon atmosphere. The resulting solution was stirred for 5 min and isobutylene (ca. 0.40 g, 7.1 mmol) was added. The reaction mixture was stirred for 10 min at -78 °C and poured into EtOAc (130 mL) / $NaHCO_3$ (sat. aq., 100 mL). Organic layer was washed with H_2O (100 mL), brine (100 mL), dried over Na_2SO_4 and evaporated. Crystallization of the residue from Et_2O afforded 0.34 g (71%) of target nitronate as white powder.
mp = 114-115 °C (Et_2O).
^{1}H NMR (300 MHz, $CDCl_3$): δ 1.46 (s, 3H, Me(6)), 1.55 (s, 3H, Me(6)), 1.72 (app t, *J* = 12.3, 1H, CH_{2ax}(5)), 2.27 (dd, *J* = 13.5, 7.2 Hz, 1H, CH_{2eq}(5)), 4.36 (m, app t, *J* = 7.6 Hz, 1H, CH(4)), 6.43 (br s, 1H, CH(3)=N), 7.19-7.45 (m, 4H, CH_{Ar}).
^{13}C NMR (75 MHz, DEPT, $CDCl_3$): δ 22.5 (Me(6)), 27.7 (Me(6)), 35.7 (CH(4)), 37.0 (CH_2(5)), 82.8 (C(6)), 112.7 (CH(3)=N), 127.9 (CH_{Ar}), 128.8 (CH_{Ar}), 129.1 (CH_{Ar}), 130.1 (CH_{Ar}), 133.2 (C_{Ar}), 137.3 (2×C_{Ar}).
HRMS (ESI): *m/z* calcd. for [$C_{12}H_{14}ClNO_2$+H^+]: 240.0786, found: 240.0789.

4-(3-(Cyclopentyloxy)-4-methoxyphenyl)-6,6-dimethyl-5,6-dihydro-4H-1,2-oxazine 2-oxide 1f

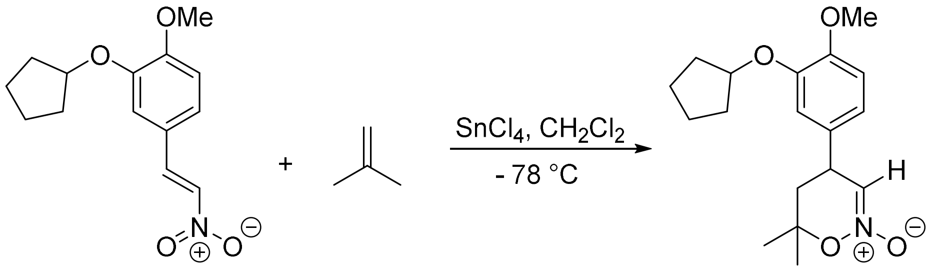

Starting nitroalkene was prepared according to the literature procedure [s10].
To a solution of (*E*)-2-(cyclopentyloxy)-1-methoxy-4-(2-nitrovinyl)benzene (0.37 g, 1.43 mmol) in freshly distilled CH_2Cl_2 (14 mL) $SnCl_4$ (0.21 ml, 0.47 g, 1.80 mmol) was added at -78 °C under argon atmosphere. The resulting solution was stirred for 5 min and isobutylene (ca. 0.40 g, 7.1 mmol) was added. The reaction mixture was stirred for 5 min at -78 °C and poured into EtOAc (130 mL) / $NaHCO_3$ (sat. aq., 100 mL). Organic layer was washed with H_2O (100 mL), brine (100 mL), dried over Na_2SO_4 and evaporated. Column chromatography (eluent: PE/EtOAc, 2:1, then 1:2) afforded 0.20 g (44%) of target nitronate as colorless oil, which solidified upon storage in a fridge.
R_f = 0.16 (PE/EtOAc, 1:1, UV, anisaldehyde).
mp = 96-98 °C (EtOAc).
^{1}H NMR (300 MHz, $CDCl_3$): δ 1.47 (s, 3H, Me(6)), 1.52 (s, 3H, Me(6)), 1.59-1.68 (m, 2H, CH_{2cycl}), 1.80-1.97 (m, 7H, 3×CH_{2cycl} and CH_{2ax}(5)), 2.10 (dd, *J* = 13.8, 7.3 Hz, 1H, CH_{2eq}(5)), 3.76 (ddd, *J* = 11.5, 7.3, 3.1 Hz, 1H, CH(4)), 3.85 (s, 3H, OMe), 4.76-4.81 (m, 1H, CH_{cycl}–O), 6.44 (d, *J* = 3.0 Hz, 1H, CH(3)=N), 6.72 (d, *J* = 2.1 Hz, 1H, CH_{Ar}), 6.76 (dd, *J* = 8.2, 2.1 Hz, 1H, CH_{Ar}), 6.85 (d, *J* = 8.2 Hz, 1H, CH_{Ar}).

^{13}C NMR (75 MHz, DEPT, CDCl$_3$): δ 22.7 (Me(6)), 24.0 (CH$_{2cycl}$), 27.8 (Me(6)), 32.77 and 32.80 (CH$_{2cycl}$), 38.4 (CH(4)), 39.5 (CH$_2$(5)), 56.1 (OMe), 80.6 (C(6)), 82.6 (CH$_{cycl}$–O), 112.4 (CH$_{Ar}$), 113.9 (CH(3)=N), 114.2 (CH$_{Ar}$), 119.7 (CH$_{Ar}$), 132.0 (C$_{Ar}$), 148.2 ($\underline{C}_{Ar}$–O), 149.7 ($\underline{C}_{Ar}$–O).
HRMS (ESI): *m/z* calcd. for [$C_{18}H_{25}NO_4$+H$^+$]: 320.1856, found: 320.1857.

(4S*,4aR*,8aR*)-4-(4-Chlorophenyl)-4a,5,6,7,8,8a-hexahydro-4H-benzo[e][1,2]oxazine 2-oxide 1k

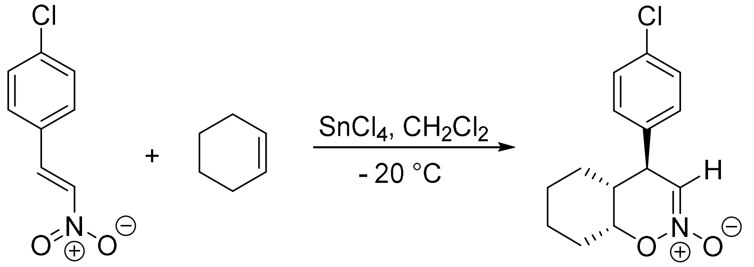

Starting nitroalkene was prepared according to the literature procedure [s11].
To a solution of (*E*)-1-chloro-4-(2-nitrovinyl)benzene (0.92 g, 5.0 mmol) in freshly distilled CH_2Cl_2 (25 mL) $SnCl_4$ (0.64 ml, 1.42 g, 5.5 mmol) was added at -78 °C under argon atmosphere. The resulting solution was stirred for 5 min and cyclohexene (1.01 mL, 0.82 g, 10 mmol) was added. The reaction mixture was warmed up to -20 °C and maintained at the same temperature for 4 d. Then the reaction mixture was poured into EtOAc (130 mL) / $NaHCO_3$ (sat. aq., 100 mL). Organic layer was washed with H_2O (100 mL), brine (100 mL), dried over Na_2SO_4 and evaporated. Column chromatography (eluent: PE/EtOAc, 2:1, then 1:1) afforded crude nitronate as oil, which was crystallized from PE/EtOAc (5:1) to give 0.16 g of target nitronate as white powder. Recrystallization of mother liquor afforded 0.43 g of target nitronate as light grey powder. Total yield: 0.59 g (45%).
R_f = 0.36 (PE/EtOAc, 1:1, UV, anisaldehyde).
mp = 94-95 °C (PE/EtOAc, 5:1).
^{1}H NMR (300 MHz, CDCl$_3$): δ 1.25-1.86 (m, 8H) and 2.07-2.15 (m, 1H) (4×CH$_2$ and CH(4a)), 3.50 (dd, *J* = 4.4, 1.3 Hz, 1H, CH(4)), 4.53-4.57 (m, 1H, CH(8a)–O), 6.42 (d, *J* = 4.4 Hz, 1H, CH(3)=N), 7.18 (d, *J* = 8.5 Hz, 2H, CH$_{Ar}$), 7.37 (d, *J* = 8.5 Hz, 2H, CH$_{Ar}$).
^{13}C NMR (75 MHz, DEPT, CDCl$_3$): δ 20.0, 24.5, 27.6, and 28.6 (4×CH$_2$), 39.2 (CH), 44.8 (CH), 76.5 (CH–O), 111.3 (CH(3)=N), 129.0 (CH$_{Ar}$), 129.3 (CH$_{Ar}$), 133.7 (C$_{Ar}$), 139.9 (C$_{Ar}$).
HRMS (ESI): *m/z* calcd. for [$C_{14}H_{16}ClNO_2$+H$^+$]: 266.0942, found: 266.0947.

2,2,2-Trichloroethyl 3-oxobutanoate

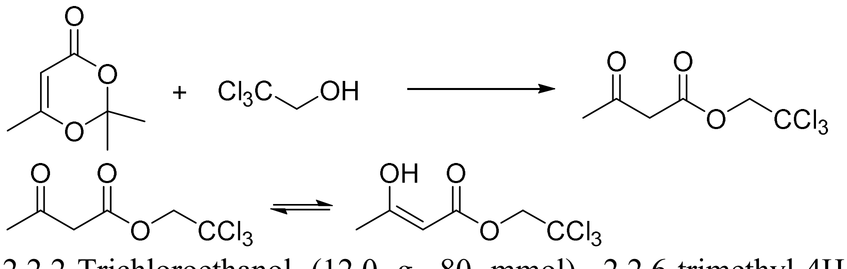

2,2,2-Trichloroethanol (12.0 g, 80 mmol), 2,2,6-trimethyl-4H-1,3-dioxin-4-one (11.4 g, 80 mmol, 1 equiv.) and *p*-xylene (20 mL) was placed in a flask equipped with a Vigreux column and a distilling link. The flask was stirred and heated to 160 °C (oil bath) for 40 min. until the acetone was distilled off (vapor temp. 56 °C). Then the apparatus was connected to a vacuum pump and xylene was distilled off at 60 mmHg (vapor temp. 60-61 °C). The residue was transferred to another apparatus and distilled under reduced pressure to give 15.4 g (82%) of target acetoacetate as colorless oil.
bp 70-77 °C/1.7 mmHg. (lit. [s12] 115 °C/10 mmHg.)

After the distillation the ratio of keto-form:enol-form = 45:55. Upon standing the amount of keto-form increased.
Keto-form:
^{1}H NMR (300 MHz, $CDCl_3$): δ 2.34 (s, 3H, Me), 3.63 (s, 2H, CH_2), 4.82 (s, 2H, OCH_2).
^{13}C NMR (75 MHz, DEPT, HSQC, HMBC, $CDCl_3$): δ 30.3 (Me), 49.5 (CH_2), 74.4 (OCH_2), 94.4 (CCl_3), 165.5 (CO_2), 199.2 (C=O).
Enol-form:
^{1}H NMR (300 MHz, $CDCl_3$): δ 2.04 (s, 3H, Me), 4.81 (s, 2H, OCH_2), 5.18 (s, 1H, =CH), 11.68 (s, 1H, OH).
^{13}C NMR (75 MHz, DEPT, HSQC, HMBC, $CDCl_3$): δ 21.4 (Me), 73.4 (OCH_2), 88.8 (=CH), 95.0 (CCl_3), 170.6 (CO_2), 177.7 (=C–O).
NMR matches previously reported data [s13].

2,2,2-Trichloroethyl 2-diazobut-3-enoate 2a

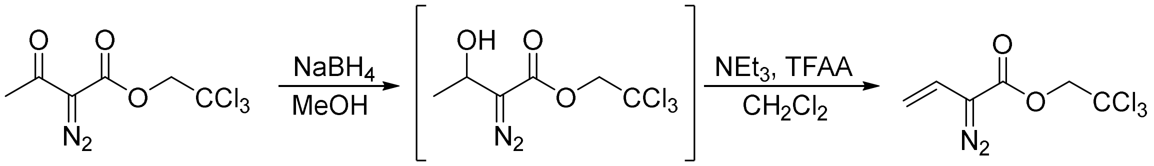

Following a modified literature procedure [s14] to a stirring solution of 2,2,2-trichloroethyl 3-oxobutanoate (1.32 g, 5.1 mmol) in MeOH (5 mL) $NaBH_4$ (0.23 g, 6.1 mmol) was added portionwise during 20 min at 0 °C under an argon atmosphere. The resulting mixture was slowly warmed up to r.t. (appr. 0.5 h), stirred for 0.5 h, and H_2O (0.50 mL) was added. The reaction mixture was stirred for another 0.5 h and transferred into CH_2Cl_2 (40 mL) / H_2O (30 mL). The aqueous layer was washed with CH_2Cl_2 (2 × 20 mL). The combined organic layer was washed with brine (40 mL), dried (Na_2SO_4) and evaporated. The residue was dissolved in CH_2Cl_2 (10 mL) and cooled to 0 °C. To the resulting solution NEt_3 (2.8 mL, 2.0 g, 20 mmol) was added. Then a solution of TFAA (1.10 mL, 1.66 g, 7.9 mmol) in CH_2Cl_2 (4 mL) was added dropwise during 5 min. The reaction mixture was maintained at the same temperature overnight, then concentrated to *ca*. 1.0 mL and subjected to column chromatography on silica gel (eluent: PE, then PE/CH_2Cl_2, 10:1) to give 0.63 g (51%) of target vinyl diazoacetate **2a** as orange oil. It was immediately dissolved in freshly distilled THF and placed over MS 4 Å. The resulting solution can be stored at -20 °C for several months. Concentration of **2a** was determined by ^{1}H NMR with the use of dimethyl terephthalate as internal standard.
R_f = 0.59 (PE/EtOAc, 3:1, UV, anisaldehyde).
NMR matches previously reported data [s15].

4-Nitrobenzyl 2-diazobut-3-enoate 2e

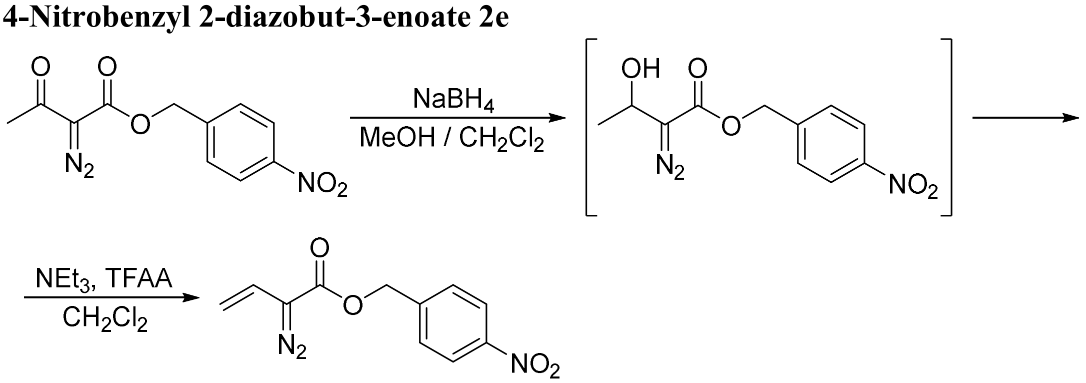

Starting diazoacetoacetate was prepared according to the literature procedure [s16].
Following a modified literature procedure [s14] to a stirring suspension of 4-nitrobenzyl 2-diazo-3-oxobutanoate (0.40 g, 1.54 mmol) in MeOH / CH_2Cl_2 (3 mL / 1 mL) $NaBH_4$ (70 mg, 1.84 mmol) was added portionwise during 5 min at 0 °C under an argon atmosphere. The resulting mixture was slowly warmed up to r.t. (appr. 0.5 h), stirred for 2 h, and H_2O (0.15 mL) was

added. The reaction mixture was stirred for another 0.5 h and transferred into CH_2Cl_2 (25 mL) / H_2O (15 mL). The aqueous layer was washed with CH_2Cl_2 (2 × 10 mL). The combined organic layer was washed with brine (20 mL), dried (Na_2SO_4) and evaporated. The residue was dissolved in CH_2Cl_2 (3.2 mL) and cooled to 0 °C. To the resulting solution NEt_3 (0.86 mL, 0.62 g, 6.1 mmol) was added, then a solution of TFAA (0.32 mL, 0.48 g, 2.30 mmol) in CH_2Cl_2 (0.75 mL) was added dropwise. The reaction mixture was warmed to r.t. and stirred for 3 h, then concentrated to appr. 0.5 mL and subjected to column chromatography on silica gel (eluent: PE, then PE/CH_2Cl_2, 3:1) to give 0.27 g (74%) of target vinyl diazoacetate **2e** as orange powder.
R_f = 0.44 (PE/EtOAc, 3:1, UV, anisaldehyde).
mp = 78-80 °C (THF).
^{1}H NMR (300 MHz, $CDCl_3$): δ 4.93 (d, J = 17.4 Hz, 1H, =CH_{2trans}), 5.18 (d, J = 11.0 Hz, 1H, =CH_{2cis}), 5.36 (s, 2H, OCH_2), 6.19 (dd, J = 17.4, 11.0 Hz, 1H, =CH), 7.54 (d, J = 8.7 Hz, 2H, CH_{Ar}), 8.25 (d, J = 8.7 Hz, 2H, CH_{Ar}).
^{13}C NMR (75 MHz, DEPT, HMBC, $CDCl_3$): δ 65.2 (CH_2), 108.2 (=CH_2), 119.9 (=CH), 123.9 (CH_{Ar}), 128.4 (CH_{Ar}), 143.0 (C_{Ar}), 147.8 (C_{Ar}–NO_2), 164.3 (CO_2). C=N_2 was not observed due to broadening / low intensity.

Phenyl 2-diazobut-3-enoate 2f

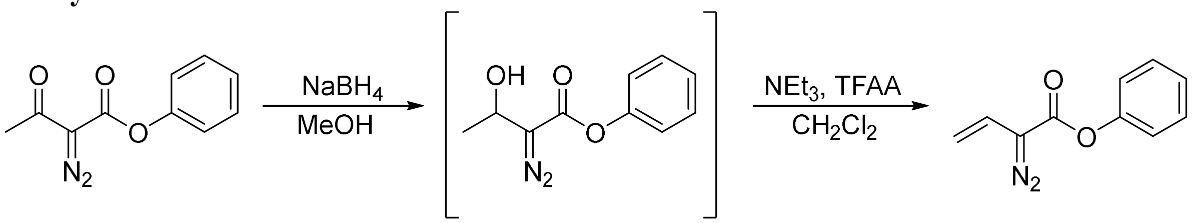

Starting diazoacetoacetate was prepared according to the literature procedure [s17].
Following a modified literature procedure [s14] to a stirring solution of phenyl 2-diazo-3-oxobutanoate (0.43 g, 2.11 mmol) in MeOH (2.1 mL) $NaBH_4$ (100 mg, 2.63 mmol) was added portionwise during 5 min at 0 °C under an argon atmosphere. The resulting mixture was slowly warmed up to r.t. (appr. 0.5 h), stirred for 2 h, and H_2O (0.21 mL) was added. The reaction mixture was stirred for another 0.5 h and transferred into CH_2Cl_2 (25 mL) / H_2O (15 mL). The aqueous layer was washed with CH_2Cl_2 (2 × 10 mL). The combined organic layer was washed with brine (20 mL), dried (Na_2SO_4) and evaporated. The residue was dissolved in CH_2Cl_2 (1.7 mL) and cooled to 0 °C. To the resulting solution NEt_3 (1.0 mL, 0.72 g, 7.1 mmol) was added, then a solution of TFAA (0.41 mL, 0.62 g, 2.94 mmol) in CH_2Cl_2 (4 mL) was added dropwise. The reaction mixture was warmed to r.t. and stirred for 3 h, then concentrated to appr. 0.5 mL and subjected to column chromatography on silica gel (eluent: PE, then PE/CH_2Cl_2, 9:1) to give 0.14 g (35%) of target vinyl diazoacetate **2f** as orange oil. The residue was immediately dissolved in freshly distilled THF. Concentration of vinyl diazoacetate **2f** was determined by ^{1}H NMR with the use of dimethyl terephthalate as internal standard.
R_f = 0.56 (PE/EtOAc, 3:1, UV, anisaldehyde).
NMR matches previously reported data [s18].

Table S2. Optimization table for the synthesis of pyrrolooxazine 4a

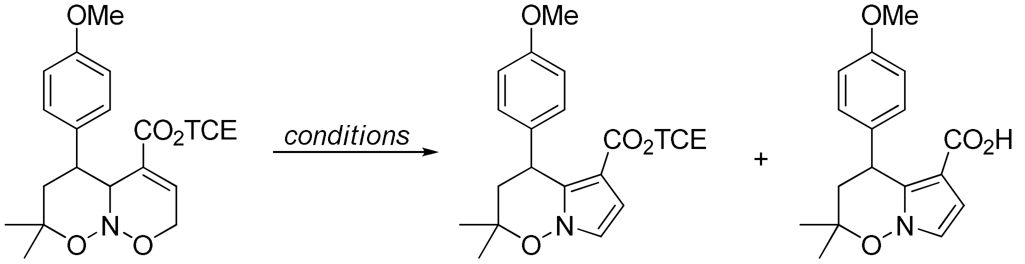

3a 4a 5

№	Solvent	Base (equiv.)	Additive (equiv.)	Time	Recovery of **3a**, %	**4a**, %	**5**, %	Comments
1	MeCN	DBU (3)	-	20 min	0	44	0	
2	THF	DBU (2)	-	1.5 h	0	48	0	
3	THF	NEt_3 (2)	-	1 d	-	-	-	no reaction (TLC)
4	THF	TCEONa (2, as soln in TCE)	(≈ 20 equiv. of TCE)	1 d	0	35	0	
5	THF	TMG (2)	-	2 d	26	6	0	
6	THF	DBN (2)	-	1 d	0	12	11	
7	THF	*t*-BuONa (2)	*t*-BuOH (20)	1 d	0	13	37	
8	-	Py (≈ 60 equiv)	-		-	-	-	no reaction (TLC)
9	THF	DBU (2)	TCE (10)	1 d	0	55	5	
10	THF	DBU (2)	HFIP (10)	3 d	69	22	0	
11	MeCN	DBU (2)	TCE (10)	1 d	0	46	4	
12	CH_2Cl_2	DBU (2)	TCE (10)	1 d	0	78	0	
13	DCE	DBU (2)	TCE (10)	1 d	0	69	0	
14	CH_2Cl_2	DBU (2)	TCE (5)	1 d	0	62	0	
15	CH_2Cl_2	DBU (2)	TCE (5)	4 d	22	45	0	at 0 °C
16	CH_2Cl_2	DBU (2)	-	1.5 h	0	53	0	
17	CH_2Cl_2	DBU (1)	TCE (10)	1 d	52	37	0	

Reactions were performed at r.t., unless otherwise mentioned. Yields were determined by ^1H NMR with internal standard.
TCE = CCl_3CH_2-

Table S3. Optimization of the synthesis of pyrrolooxazine 4b

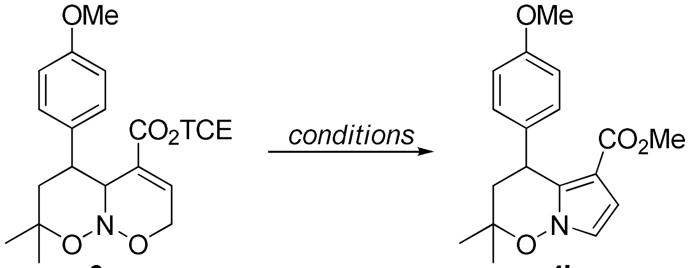

3a 4b

№	Solvent (v/v)	Base (equiv.)	Time	Yield 4b, %
1	MeOH (0.2 M)	MeONa (2)	1 d	53
2	MeOH / THF (2:1)	MeONa (2)	1 d	34
3	MeOH / CH_2Cl_2 (2:1)	MeONa (2)	1 d	57
4	MeOH / THF (2:1)	DBU (2)	5 h	56
5	MeOH / MeCN (2:1)	DBU (2)	12 h	64
6	MeOH / CH_2Cl_2 (2:1)	DBU (2)	1 d	60

Reactions were performed at r.t. at 0.2 M in pure MeOH, or at 0.13 M if a solvent mixture was used.

References:

s1. Ushakov, P. Yu.; Tabolin, A. A.; Ioffe, S. L.; Sukhorukov, A. Yu. *Eur. J. Org. Chem.* **2019**, 1888-1892. doi: 10.1002/ejoc.201801761.

s2. Gorbacheva, E. O.; Tabolin, A. A.; Novikov, R. A.; Khomutova, Yu. A.; Nelyubina, Yu. V.; Tomilov, Yu. V.; Ioffe, S. L. *Org. Lett.* **2013**, *15*, 350-353. doi: 10.1021/ol303292c

s3. Smirnov, V. O.; Ioffe, S. L.; Tishkov, A. A.; Khomutova, Yu. A.; Nesterov, I. D.; Antipin, M. Yu.; Smit, W. A.; Tartakovsky, V. A. *J. Org. Chem.* **2004**, *69*, 8485-8488. doi: 10.1021/jo048944k.

s4. Mikhaylov, A. A.; Dilman, A. D.; Khomutova, Yu. A.; Arkhipov, D. E.; Korlyukov, A. A.; Ioffe, S. L. *Eur. J. Org. Chem.* **2013**, 5670-5677. doi: 10.1002/ejoc.201300511.

s5. Davies, H. M. L.; Hougland, P. W.; Cantrell Jr., W. R. *Synth. Commun.* **1992**, *22*, 971-978. doi: 10.1080/00397919208019285.

s6. Wu, J.-Q.; Yang, Z.; Zhang, S.-S.; Jiang, C.-Y.; Li, Q.; Huang, Z.-S.; Wang, H. *ACS. Catal.* **2015**, *5*, 6453-6457. doi: 10.1021/acscatal.5b01801.

s7. Xu, C.; Du, J.; Ma, L.; Li, G.; Tao, M.; Zhang, W. *Tetrahedron* **2013**, *69*, 4749-4757. doi: 10.1016/j.tet.2013.02.084.

s8. Ramirez-Osuna, M.; Chavez, D.; Hernandez, L.; Mollins, E.; Somanathan, R.; Aguirre, G. *Molecules*, **2005**, *10*, 295-301. doi: 10.3390/10010295.

s9. Jakubec, P.; Cockfield, D. M.; Hynes, P. S.; Cleator, E.; Dixon, D. J. *Tetrahedron Asymm.* **2011**, *22*, 1147-1155. doi: 10.1016/j.tetasy.2011.06.002.

s10. Wen, L.; Tang, F.; Ge, C.; Wang, X.; Han, Z.; Wu, J. *Synth. Commun.* **2012**, *42*, 3288-3295. doi: 10.1080/00397911.2011.580880.

s11. Fadeeva, A. A.; Ioffe, S. L.; Tabolin, A. A. *Synthesis* **2020**, *52*, 2679-2688. doi: 10.1055/s-0040-1707396.

s12. Korte, F.; Wusten, F. *Justus Liebigs Ann. Chem.* **1961**, *647*, 18-22. doi: 10.1002/jlac.19616470104.

s13. Dolle, F.; Hinnen, F.; Valette, H.; Fuseau, C.; Duval, R.; Peglion, J.-L.; Crouzel, C. *Bioorg. Med. Chem. Lett.* **1997**, *5*, 749-764. doi: 10.1016/S0968-0896(97)00024-2.

s14. Hansen, J; Li, B.; Dikarev, E.; Autschbach, J.; Davies, H. M. L. *J. Org. Chem.* **2009**, *74*, 6564-6571. doi: 10.1021/jo900998s.

s15. Chinthapally, K.; Massaro, N. P.; Ton, S.; Gardner, E. D.; Sharma, I. *Tetrahedron Lett.* **2019**, *60*, 151253. doi: 10.1016/j.tetlet.2019.151253.

s16. Shved, A. S.; Tabolin, A. A.; Novikov, R. A.; Nelyubina, Yu. A.; Timofeev, V. P.; Ioffe, S. L. *Eur. J. Org. Chem.* **2016**, 5569-5578. doi: 10.1002/ejoc.201600952.

s17. Rossbach, J.; Baumeister, J.; Harms, K.; Koert, U. *Eur. J. Org. Chem.* **2013**, 662-665. doi: 10.1002/ejoc.201201517.

s18. Sarabia, F. J.; Li, Q.; Ferreira, E. M. *Angew. Chem. Int. Ed.* **2018,** *57*, 11015-11019. doi: 10.1002/anie.201805732.

Copies of NMR spectra

6,6-Dimethyl-4-(4-nitrophenyl)-5,6-dihydro-4H-1,2-oxazine 2-oxide 1c

^{1}H NMR (300 MHz, $CDCl_3$)

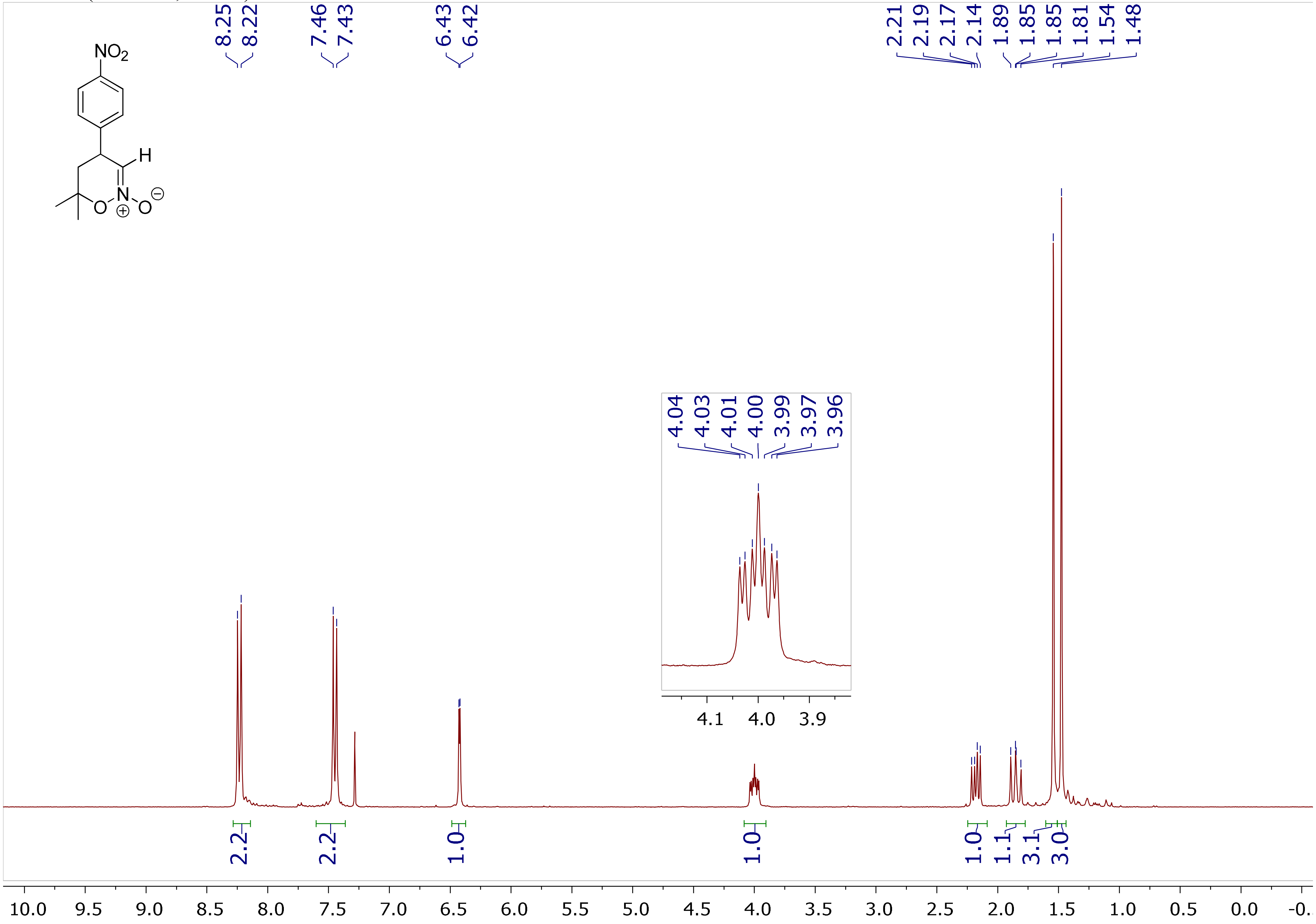

^{13}C NMR (75 MHz, $CDCl_3$)

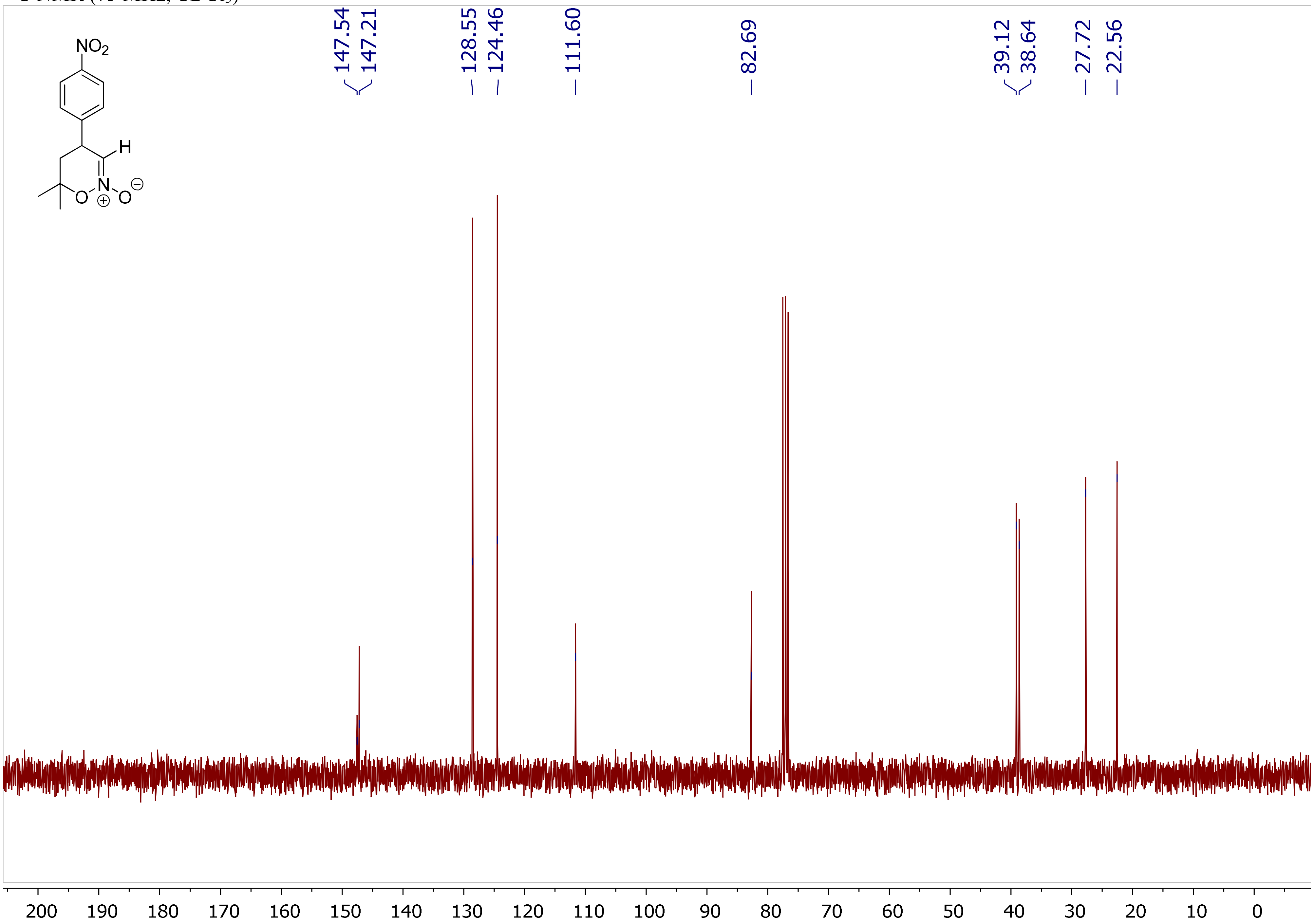

^{13}C DEPT 135 (75 MHz, $CDCl_3$)

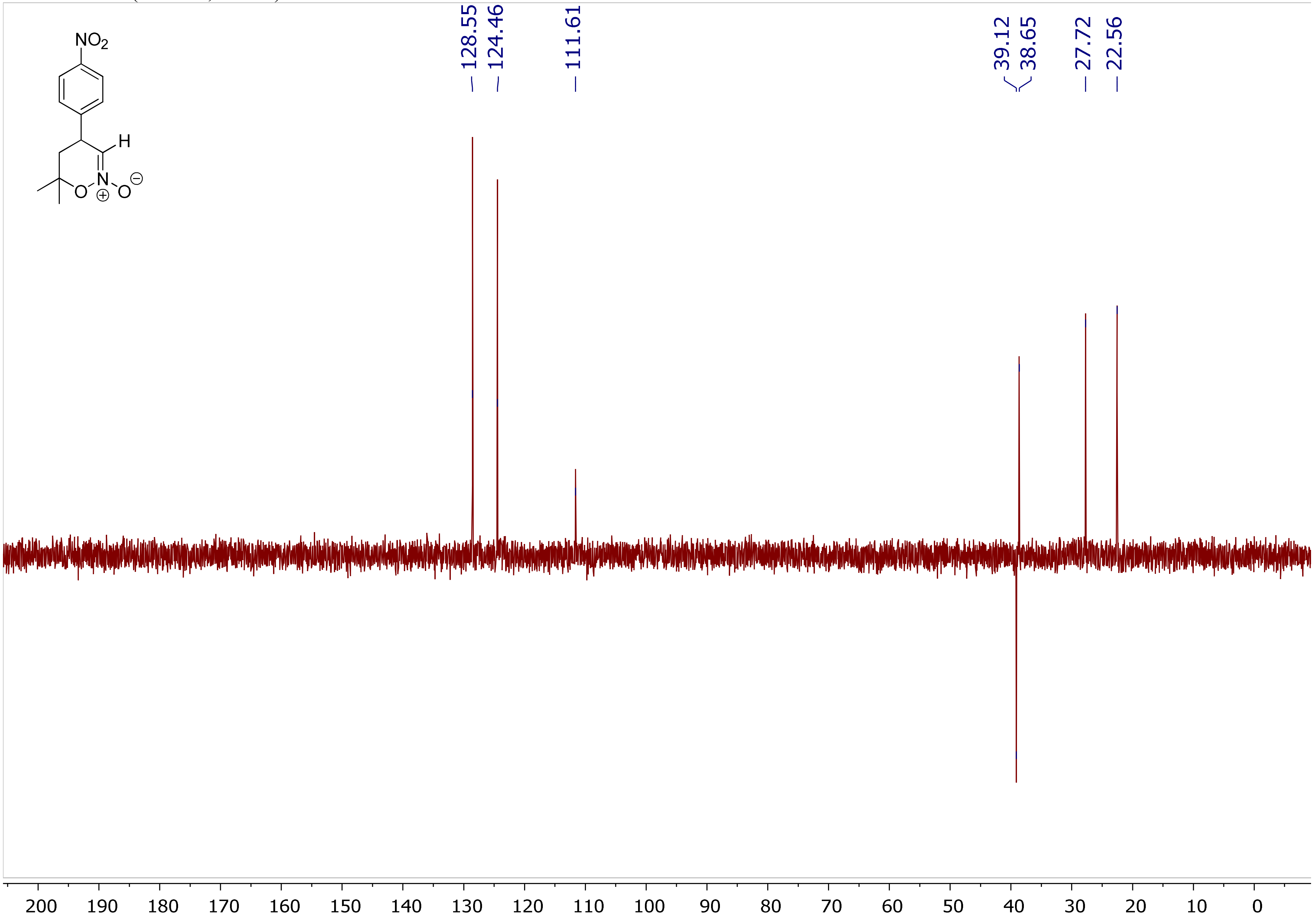

^{1}H NMR (300 MHz, $CDCl_3$)

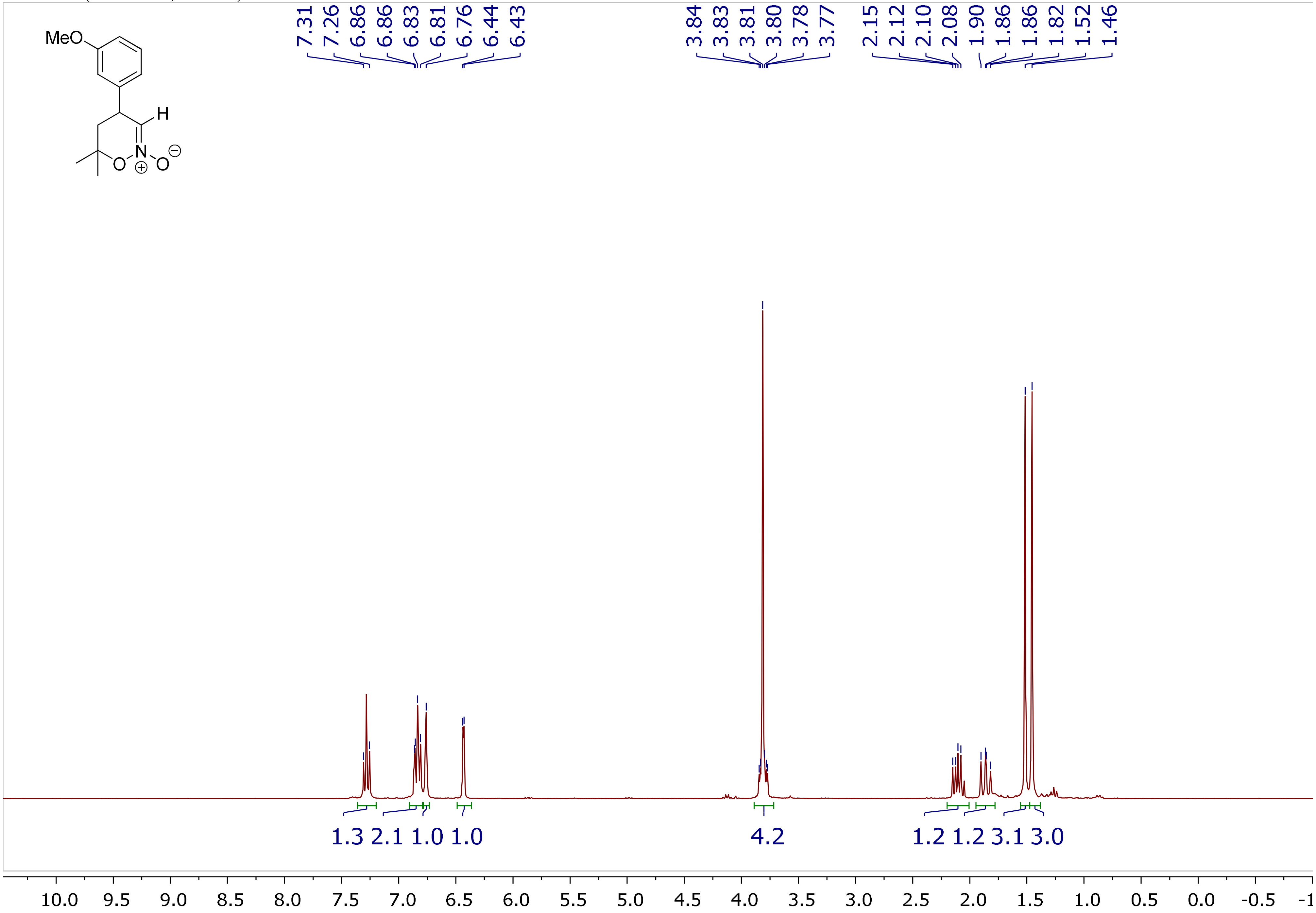

^{13}C NMR (75 MHz, $CDCl_3$)

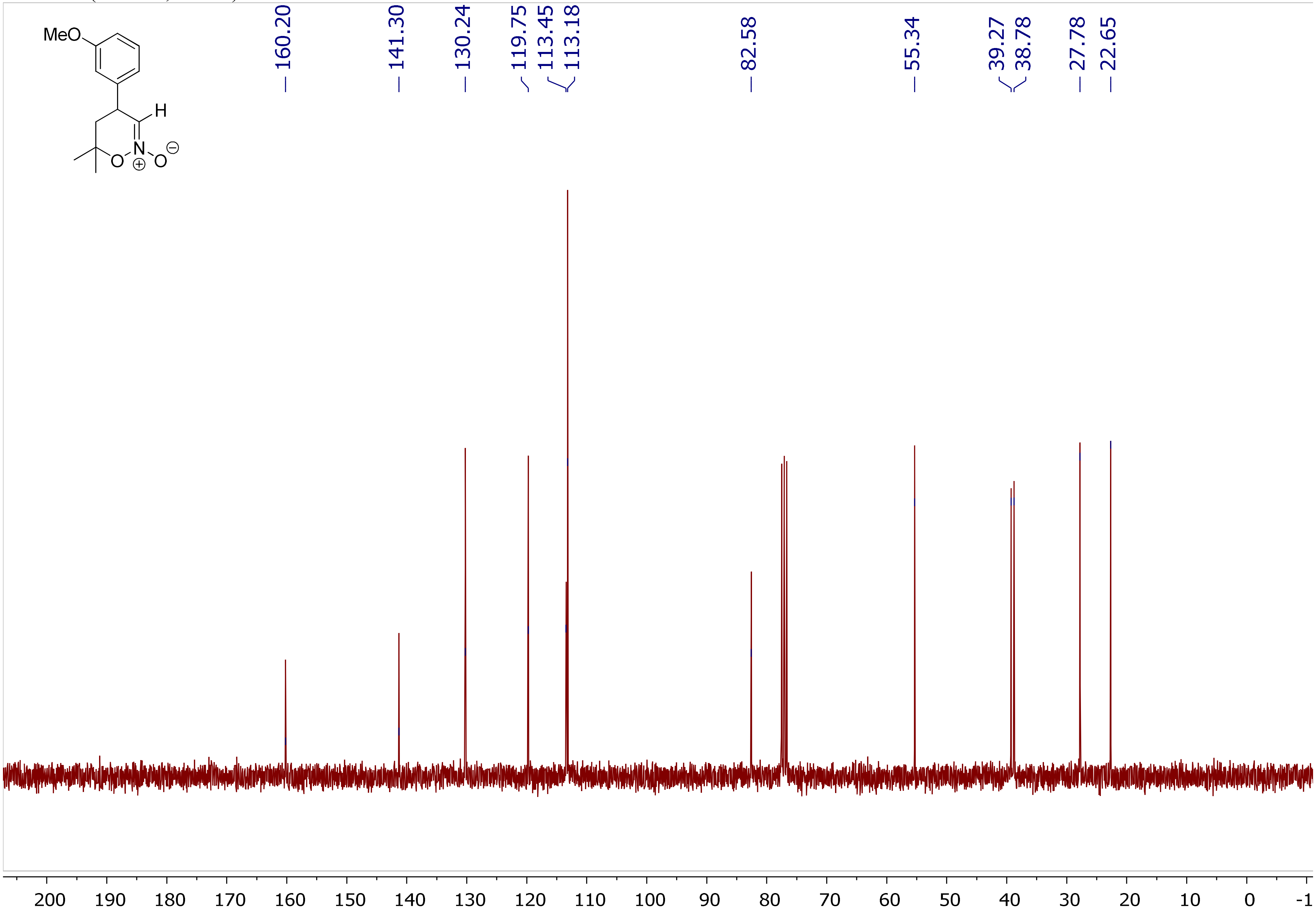

^{13}C DEPT 135 (75 MHz, $CDCl_3$)

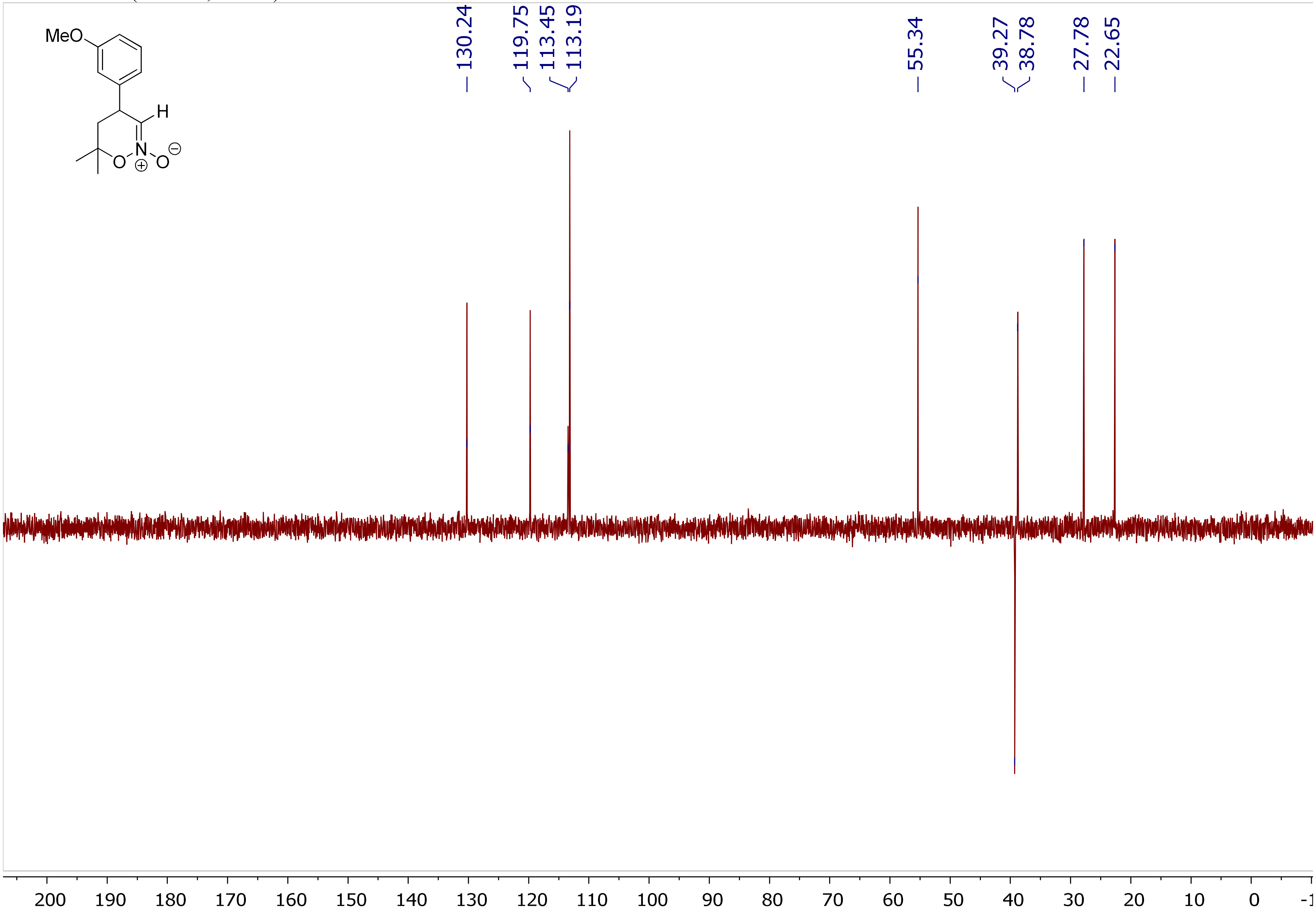

^{1}H-^{1}H COSY

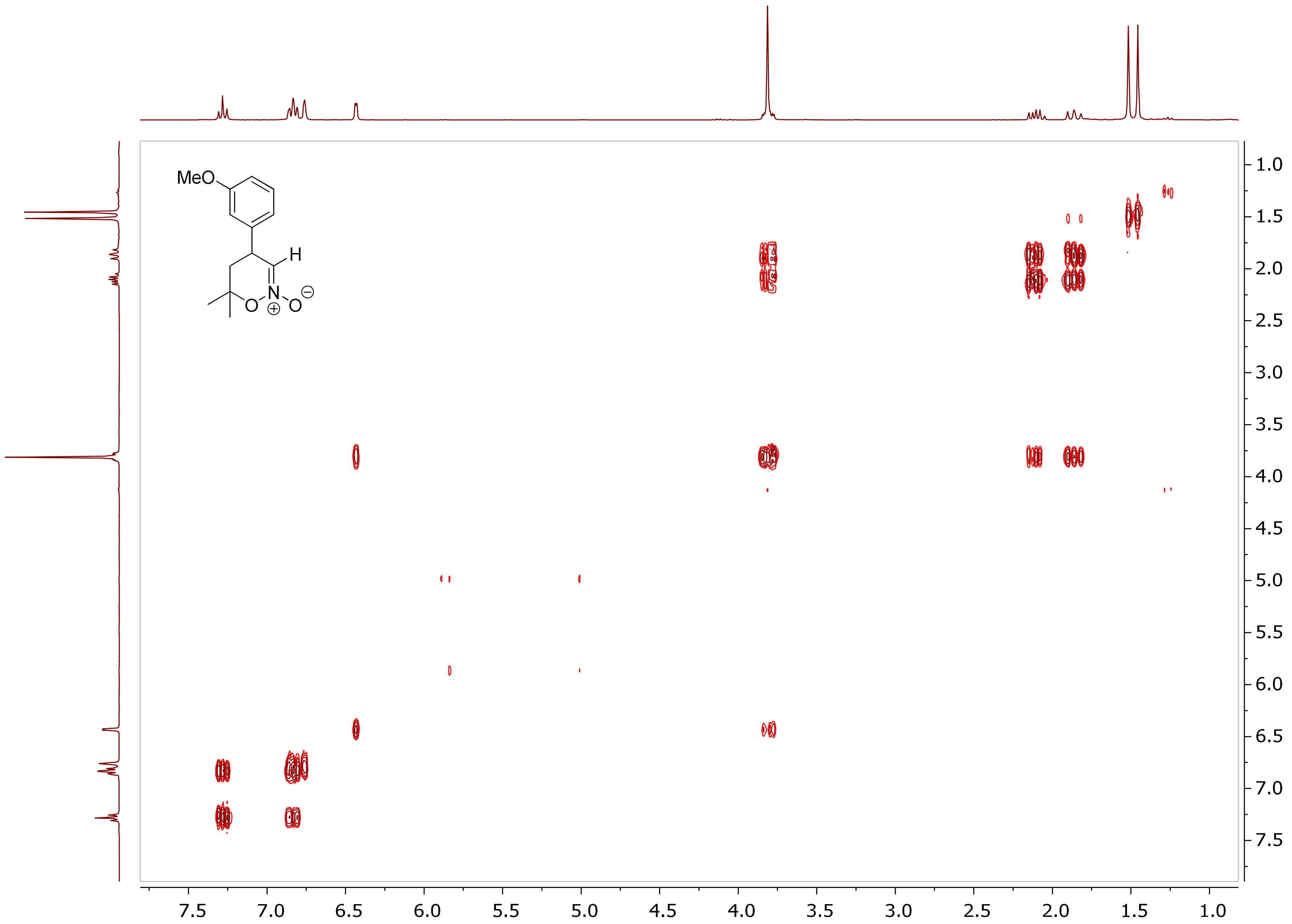

^{1}H-^{13}C HSQC

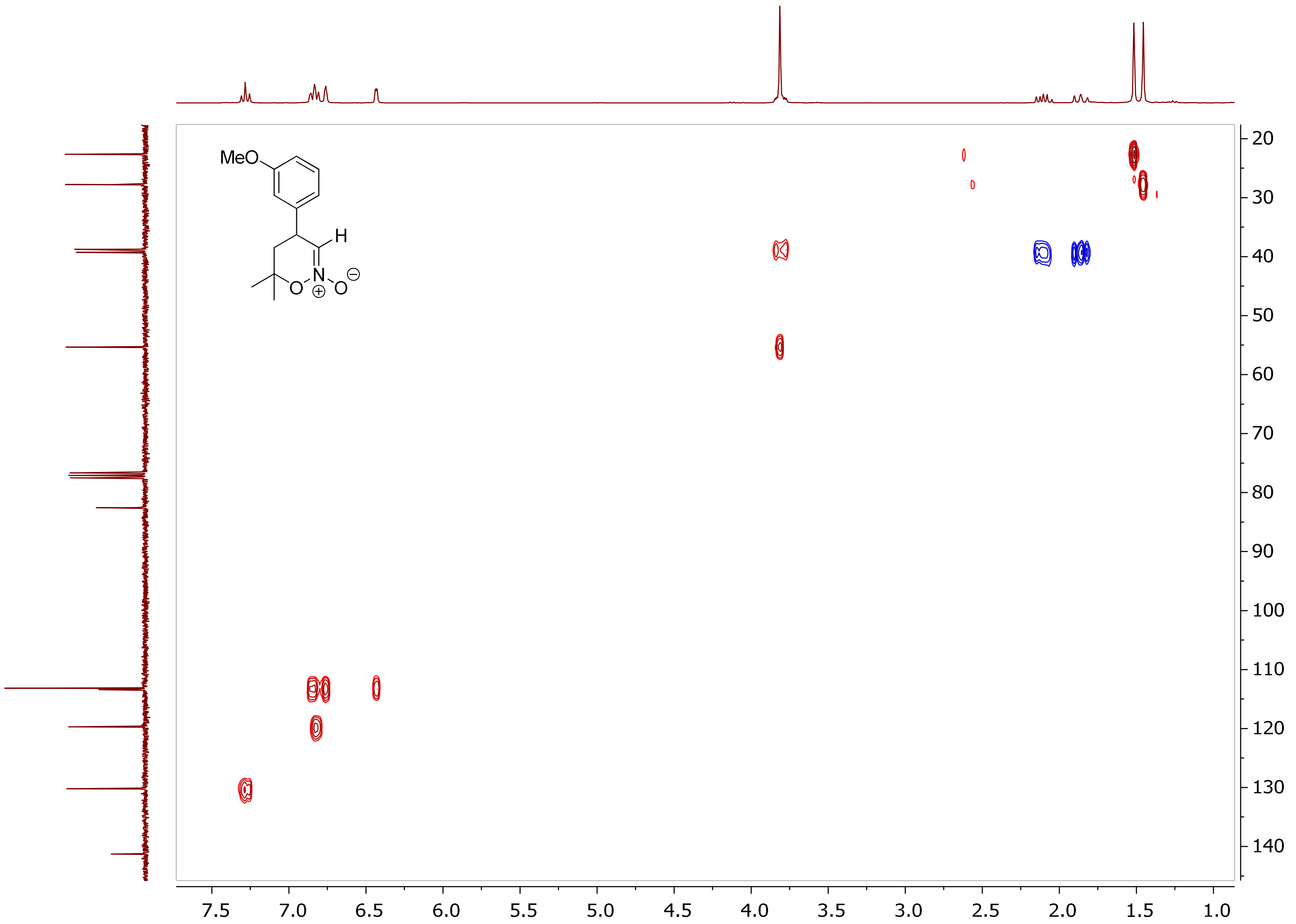
MeO
H
N
O
O
20
30
40
50
60
70
80
90
100
110
120
130
140
7.5
7.0
6.5
6.0
5.5
5.0
4.5
4.0
3.5
3.0
2.5
2.0
1.5
1.0

4-(2-Chlorophenyl)-6,6-dimethyl-5,6-dihydro-4H-1,2-oxazine 2-oxide 1e

^{1}H NMR (300 MHz, $CDCl_3$)

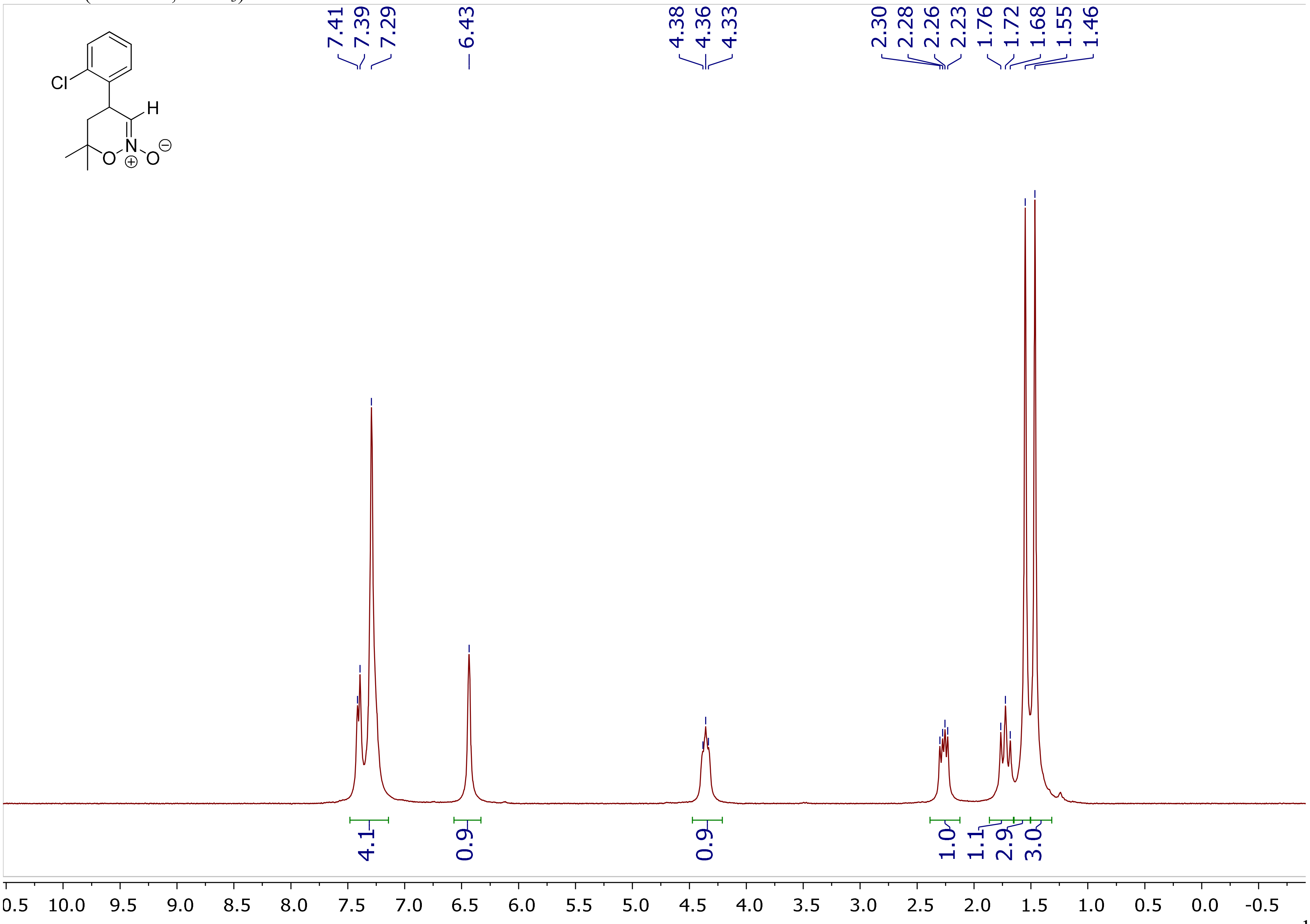

^{13}C NMR (75 MHz, $CDCl_3$)

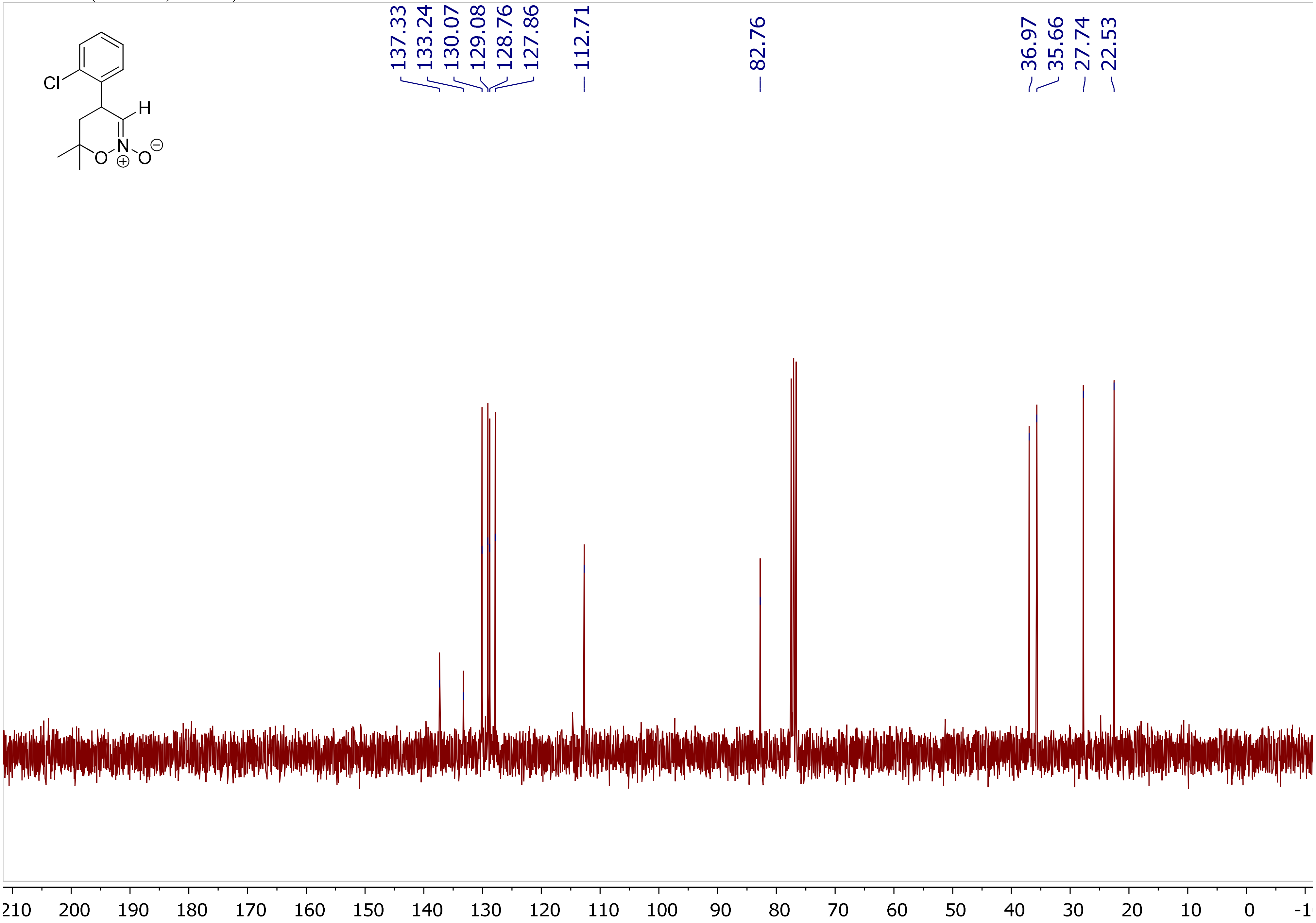

^{13}C DEPT 135 (75 MHz, $CDCl_3$)

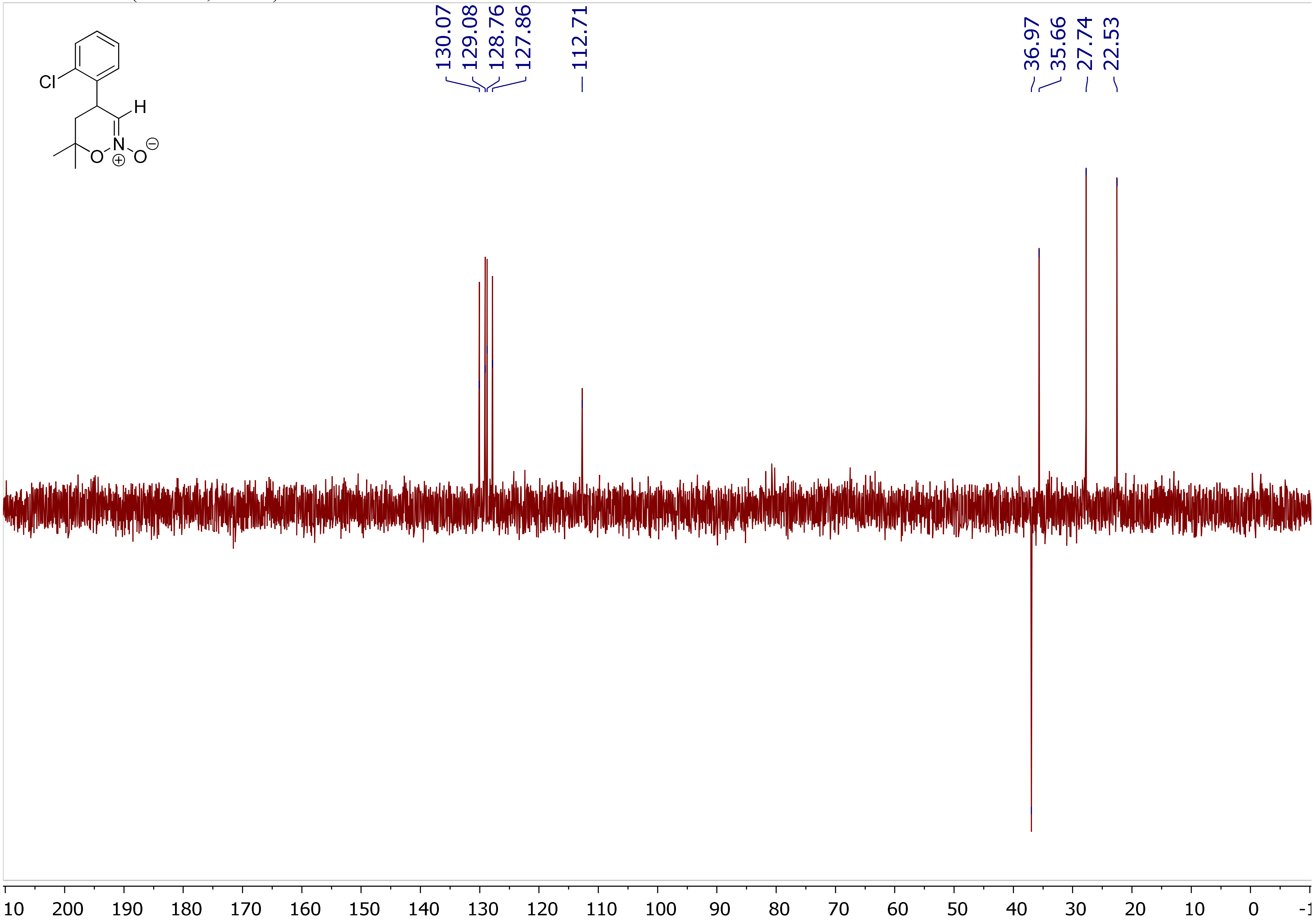

4-(3-(Cyclopentyloxy)-4-methoxyphenyl)-6,6-dimethyl-5,6-dihydro-4H-1,2-oxazine 2-oxide 1f

^{1}H NMR (300 MHz, $CDCl_3$)

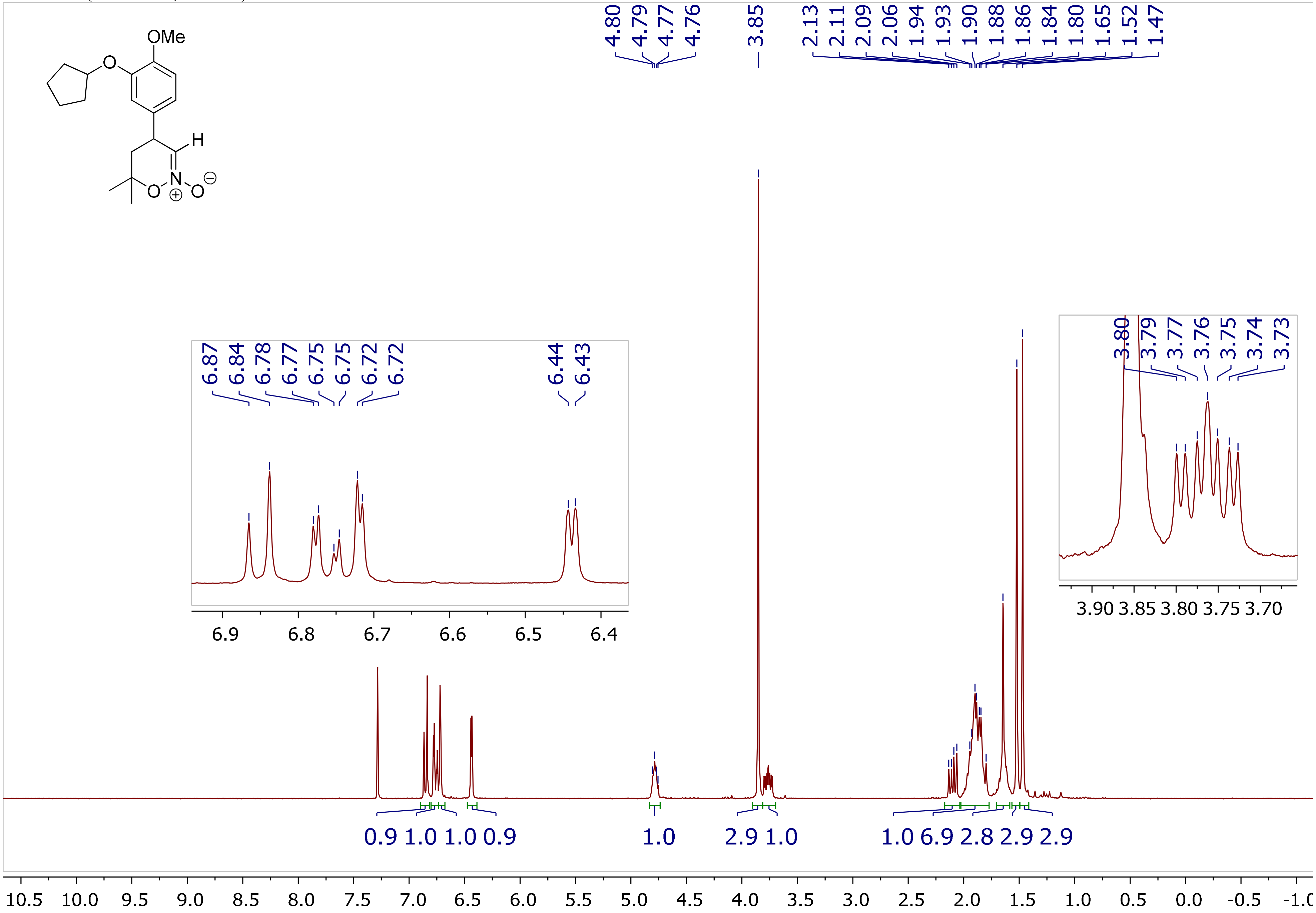

^{13}C NMR (75 MHz, $CDCl_3$)

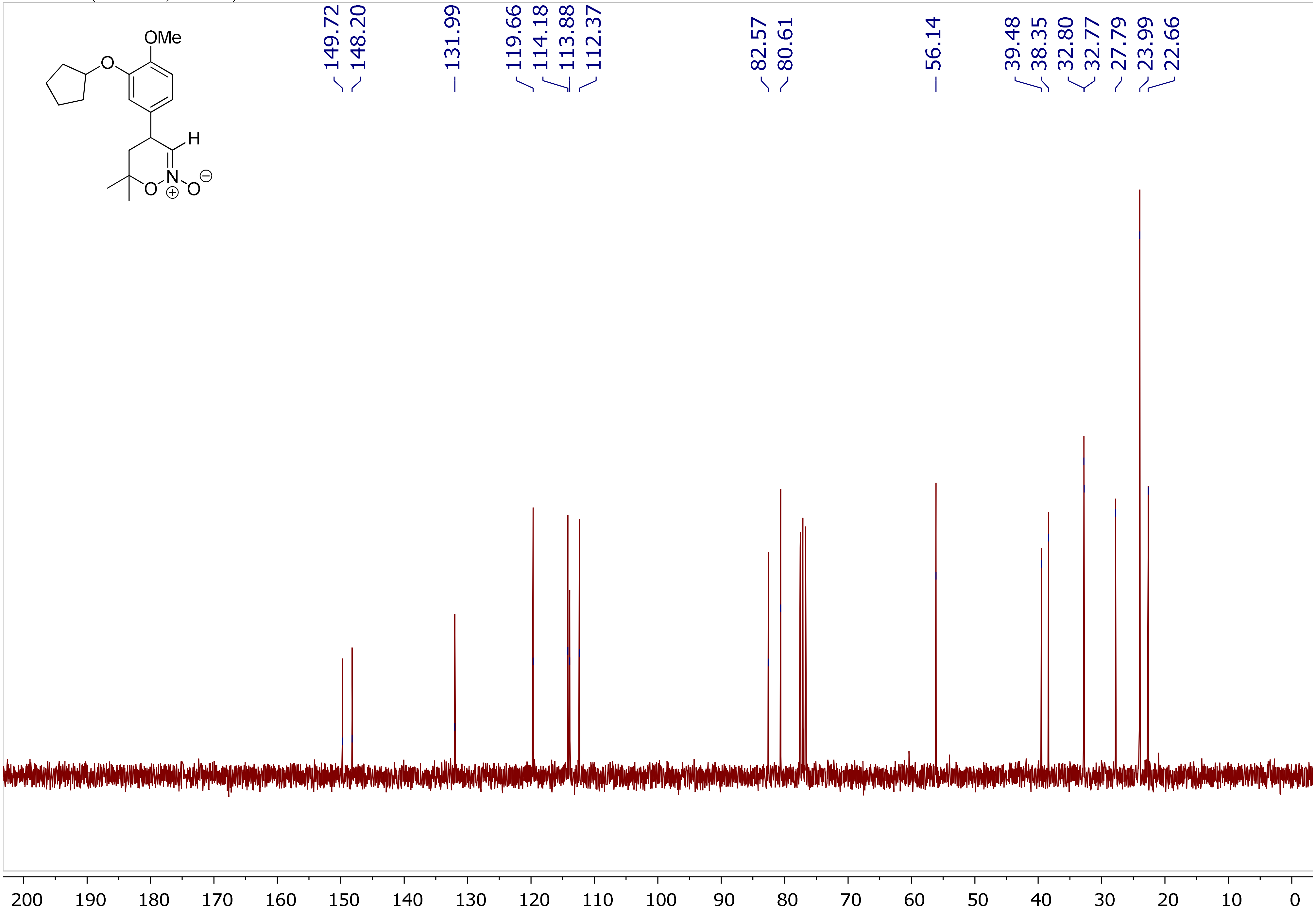

^{13}C DEPT 135 (75 MHz, $CDCl_3$)

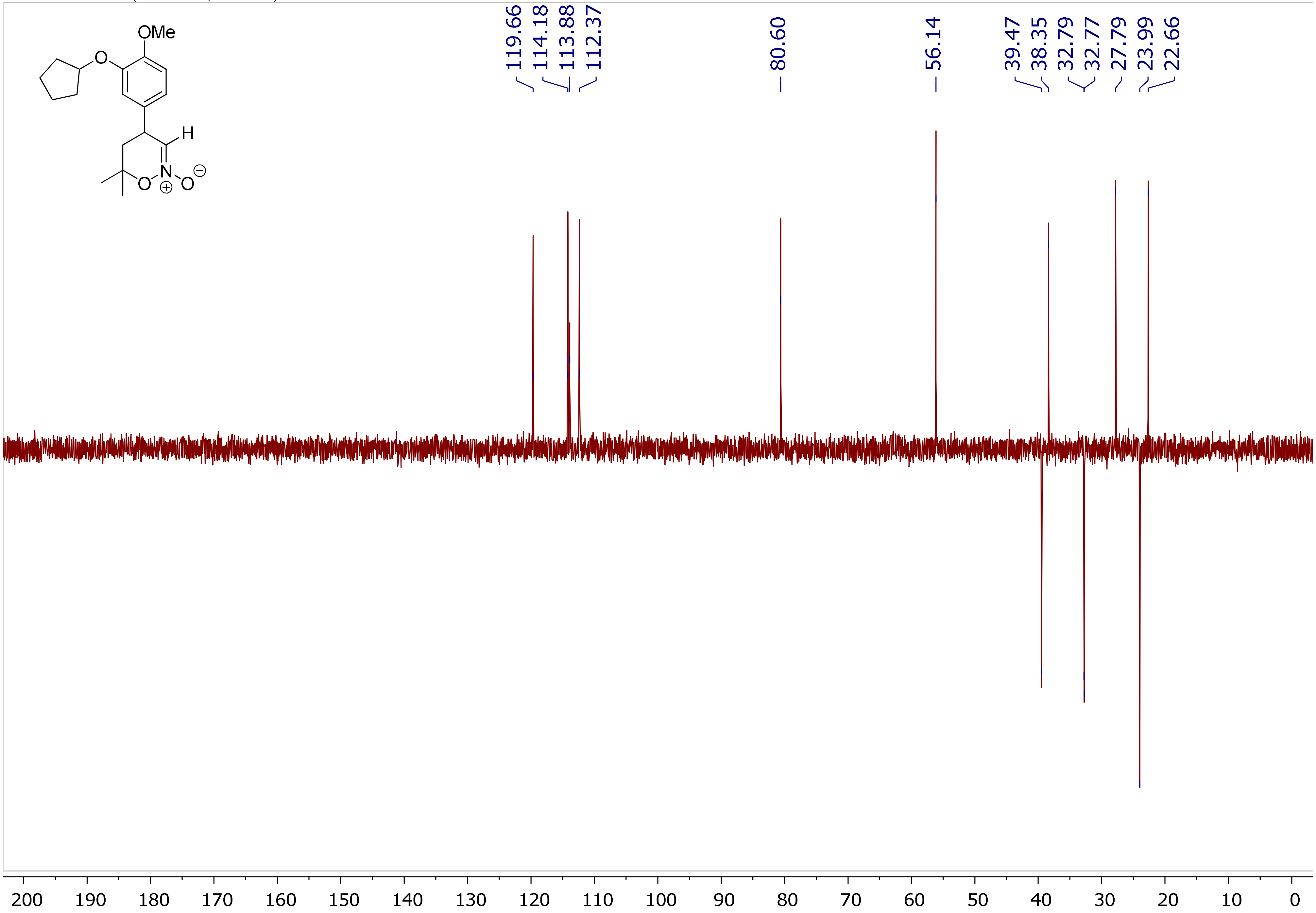

(4S*,4aR*,8aR*)-4-(4-Chlorophenyl)-4a,5,6,7,8,8a-hexahydro-4H-benzo[e][1,2]oxazine 2-oxide 1k

^{1}H NMR (300 MHz, $CDCl_3$)

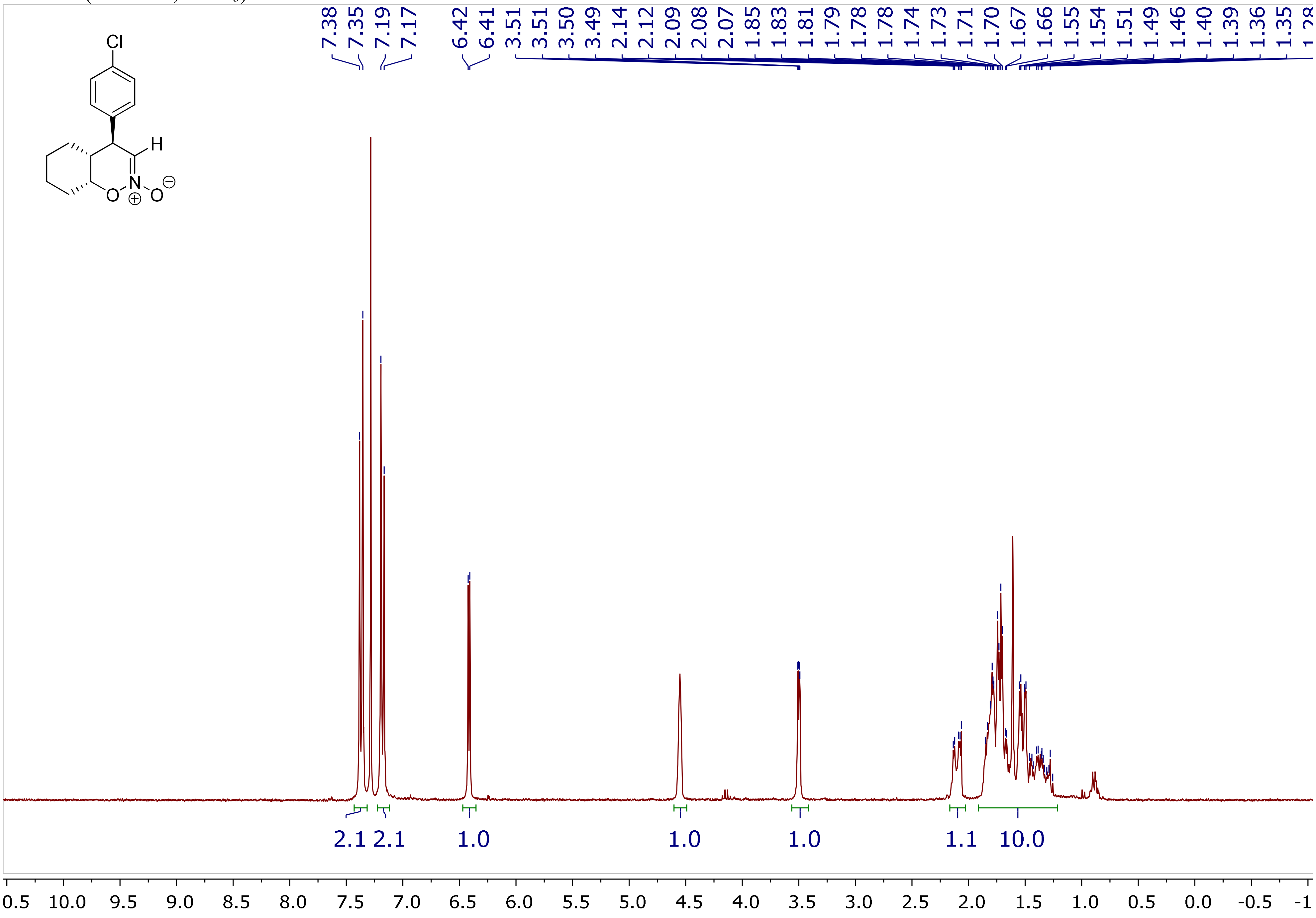

^{13}C NMR (75 MHz, $CDCl_3$)

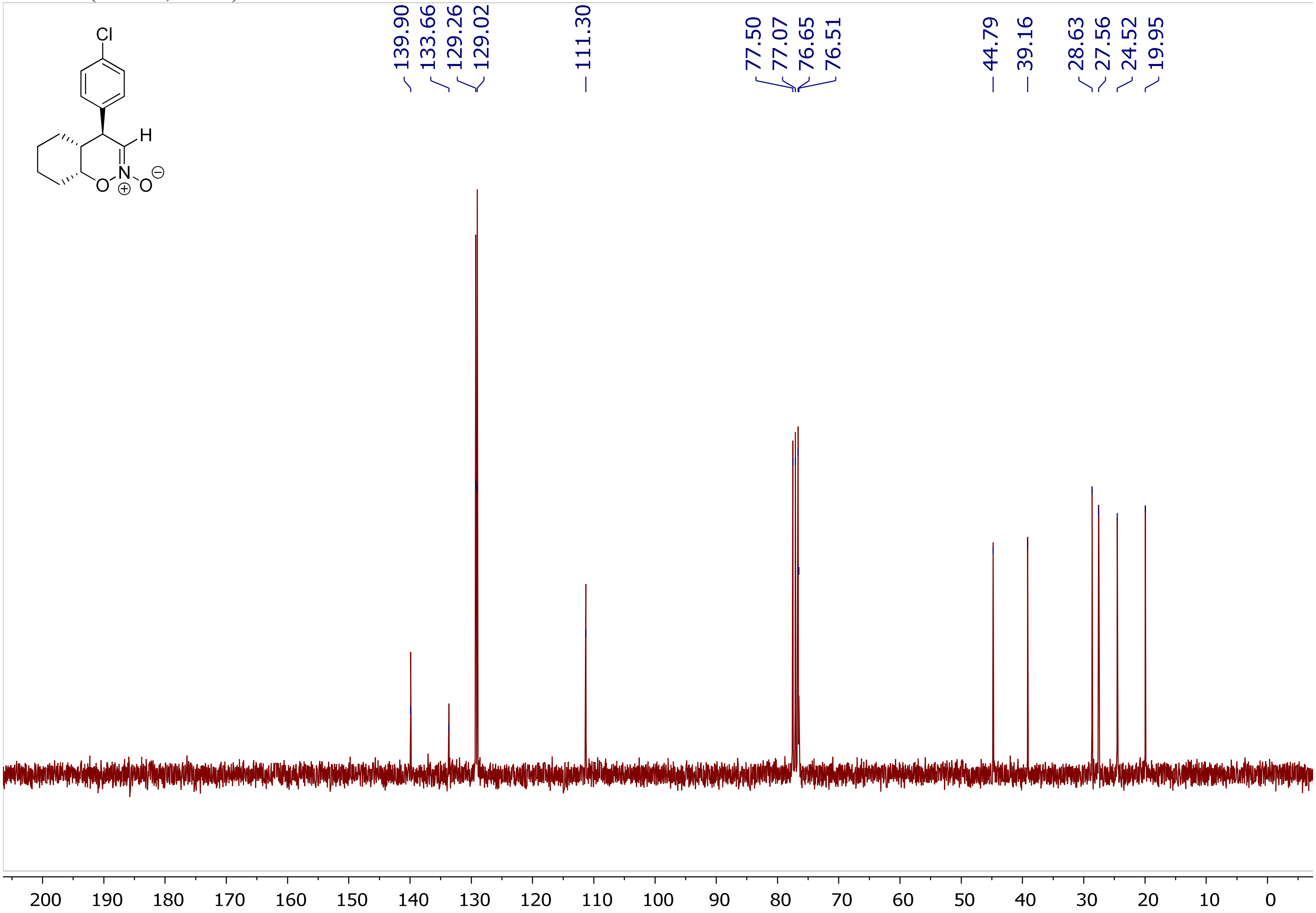

^{13}C DEPT 135 (75 MHz, $CDCl_3$)

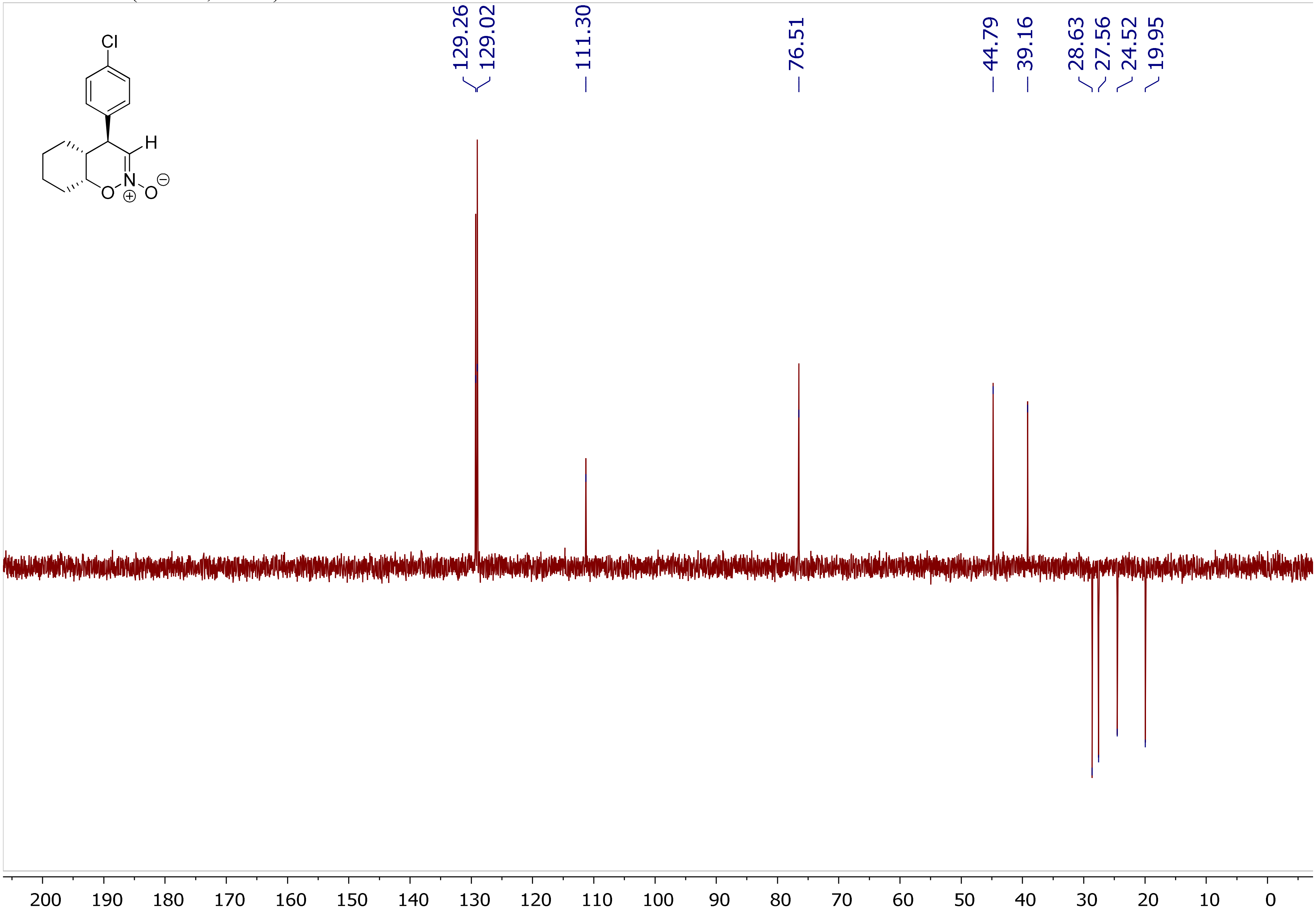

2,2,2-Trichloroethyl 3-oxobutanoate

^{1}H NMR (300 MHz, $CDCl_3$)

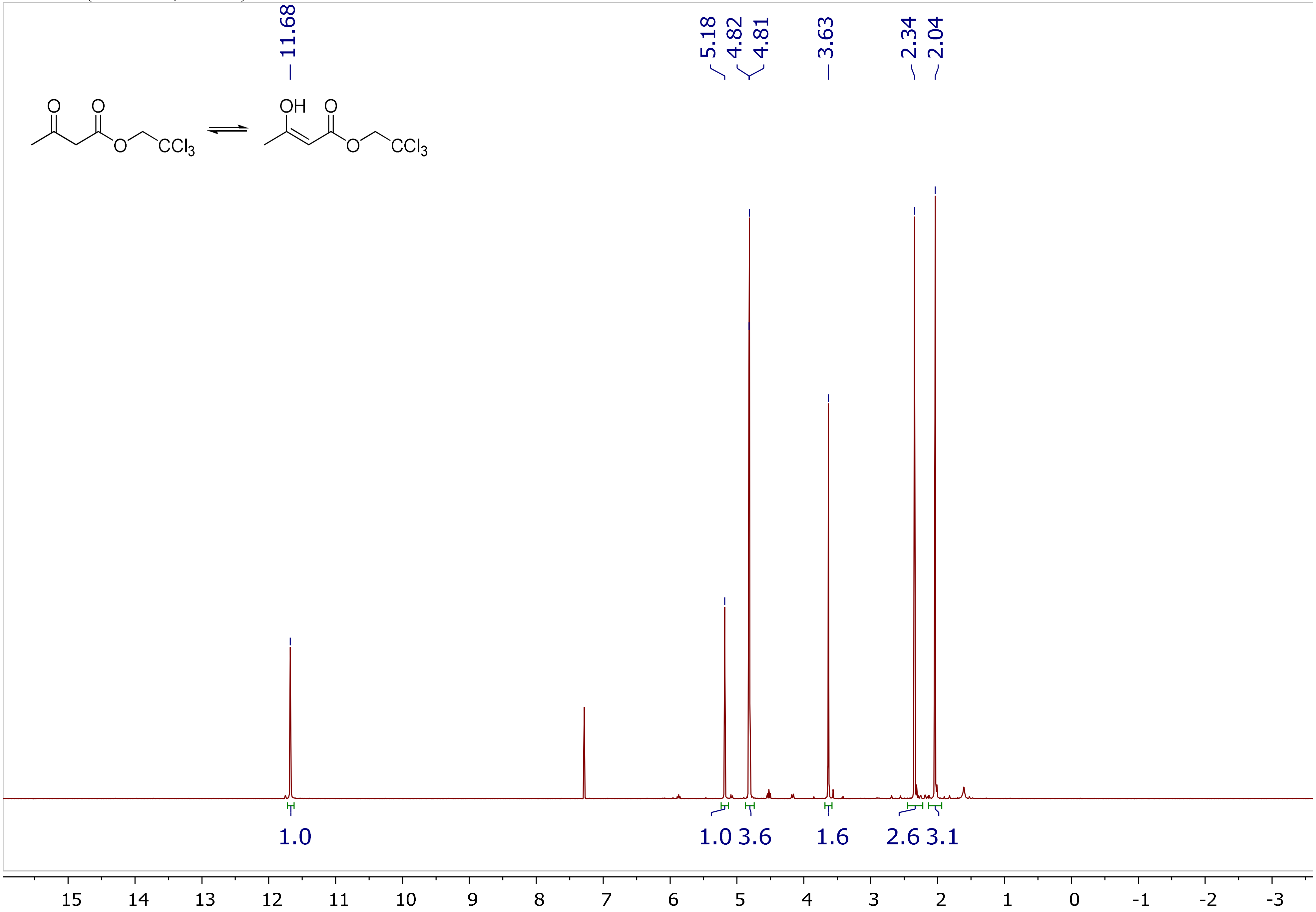

^{13}C NMR (75 MHz, $CDCl_3$)

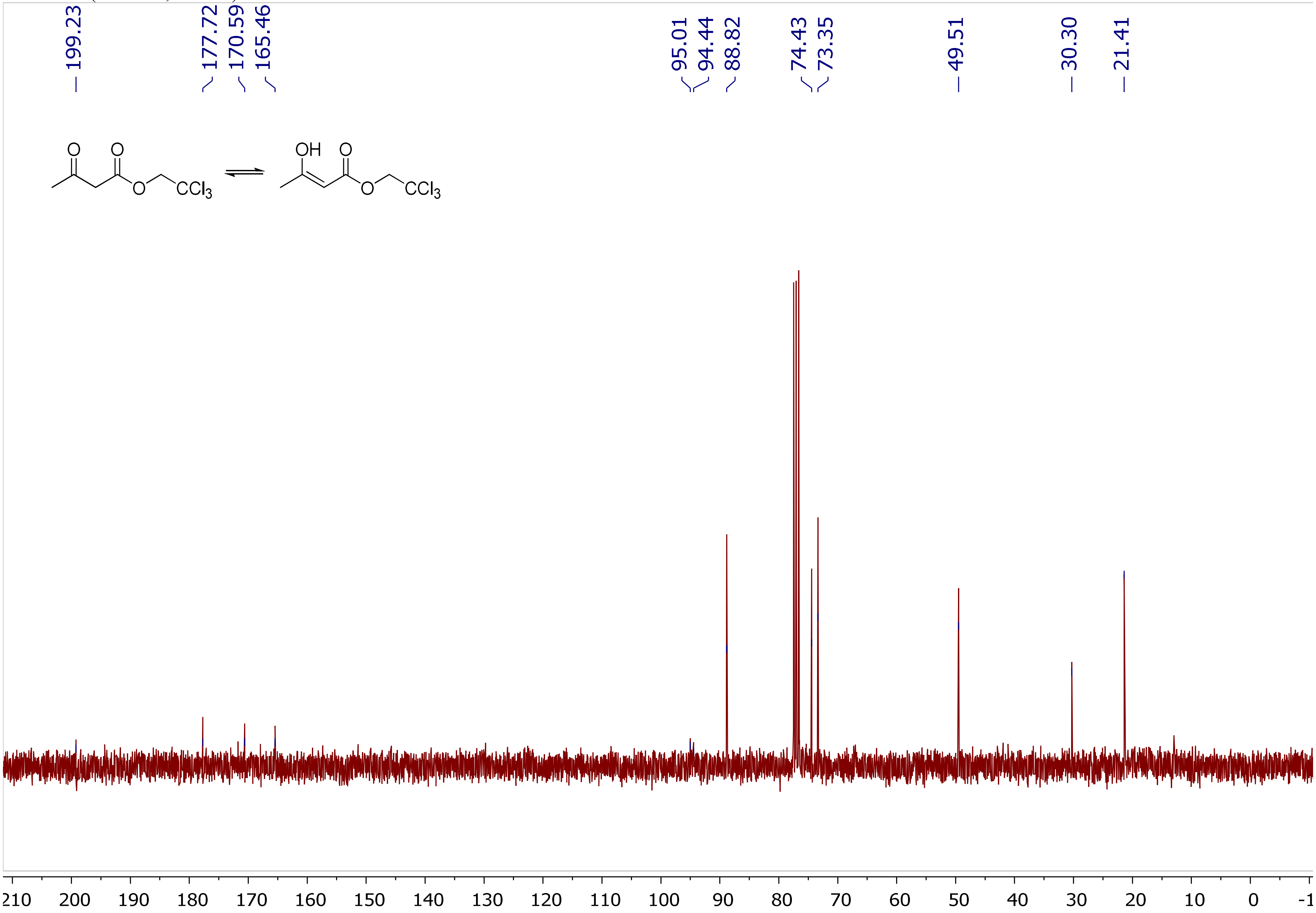

^{13}C DEPT 135 (75 MHz, $CDCl_3$)

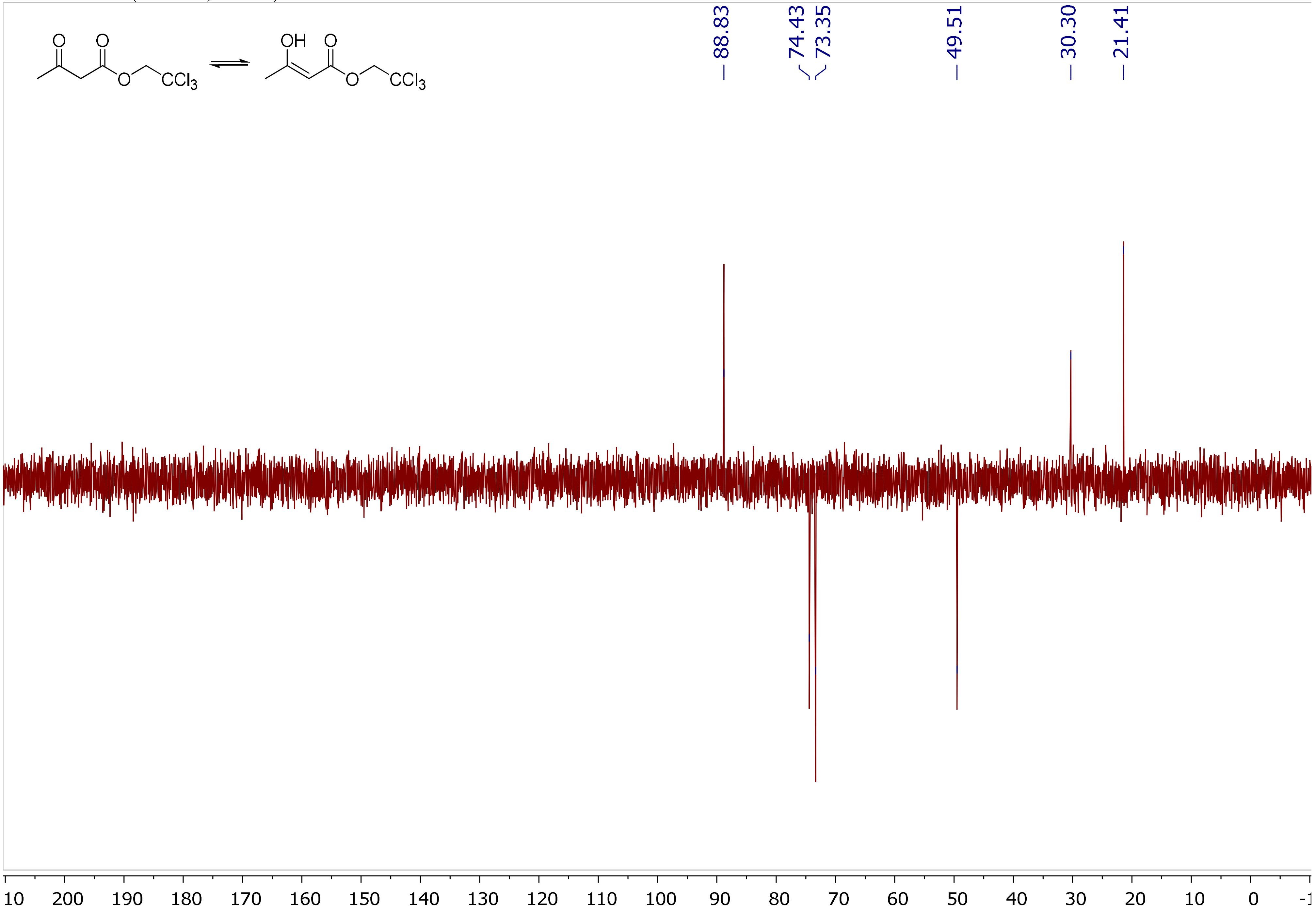

^1H-^{13}C HSQC

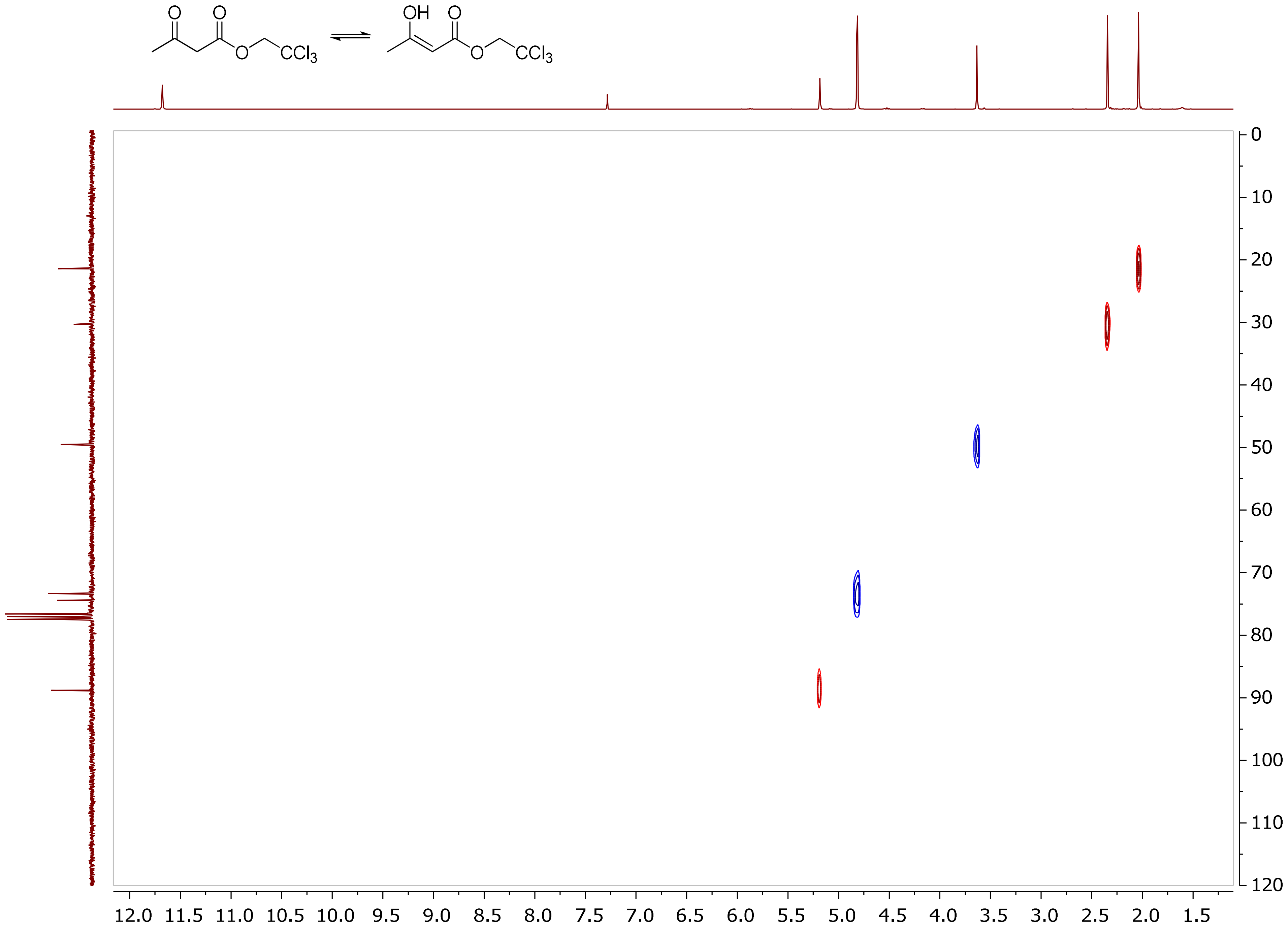

OH
CCl3
12.0 11.5 11.0 10.5 10.0 9.5 9.0 8.5 8.0 7.5 7.0 6.5 6.0 5.5 5.0 4.5 4.0 3.5 3.0 2.5 2.0 1.5
0 10 20 30 40 50 60 70 80 90 100 110 120

^{1}H-^{13}C HMBC

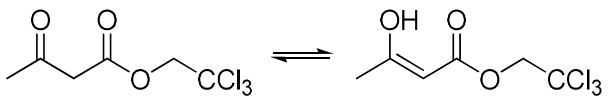

O
O
O
CCl3
OH
O
O
CCl3

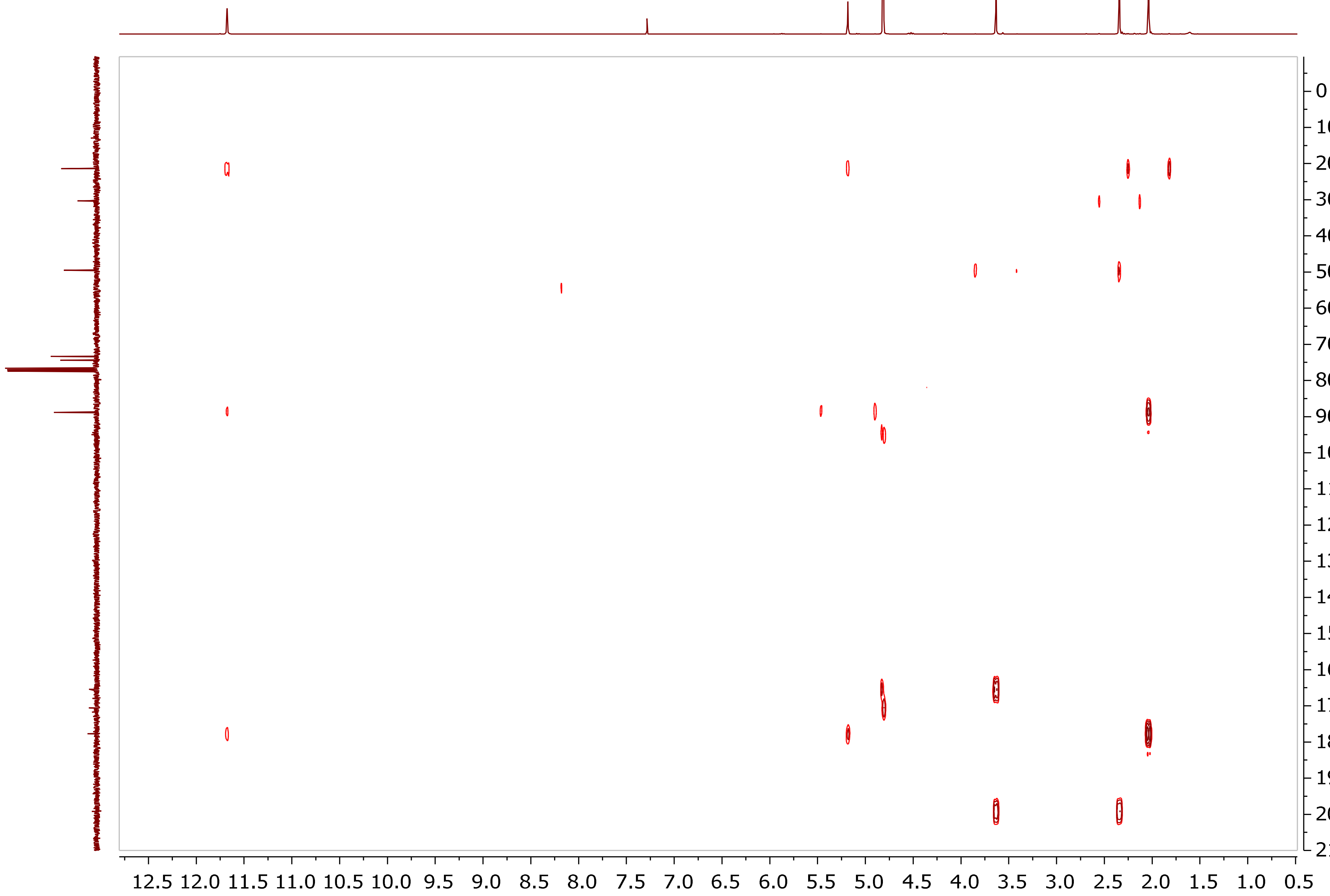

0
10
20
30
40
50
60
70
80
90
100
110
120
130
140
150
160
170
180
190
200
210
12.5 12.0 11.5 11.0 10.5 10.0 9.5 9.0 8.5 8.0 7.5 7.0 6.5 6.0 5.5 5.0 4.5 4.0 3.5 3.0 2.5 2.0 1.5 1.0 0.5

2,2,2-Trichloroethyl 2-diazobut-3-enoate 2a
^{1}H NMR (300 MHz, $CDCl_3$)

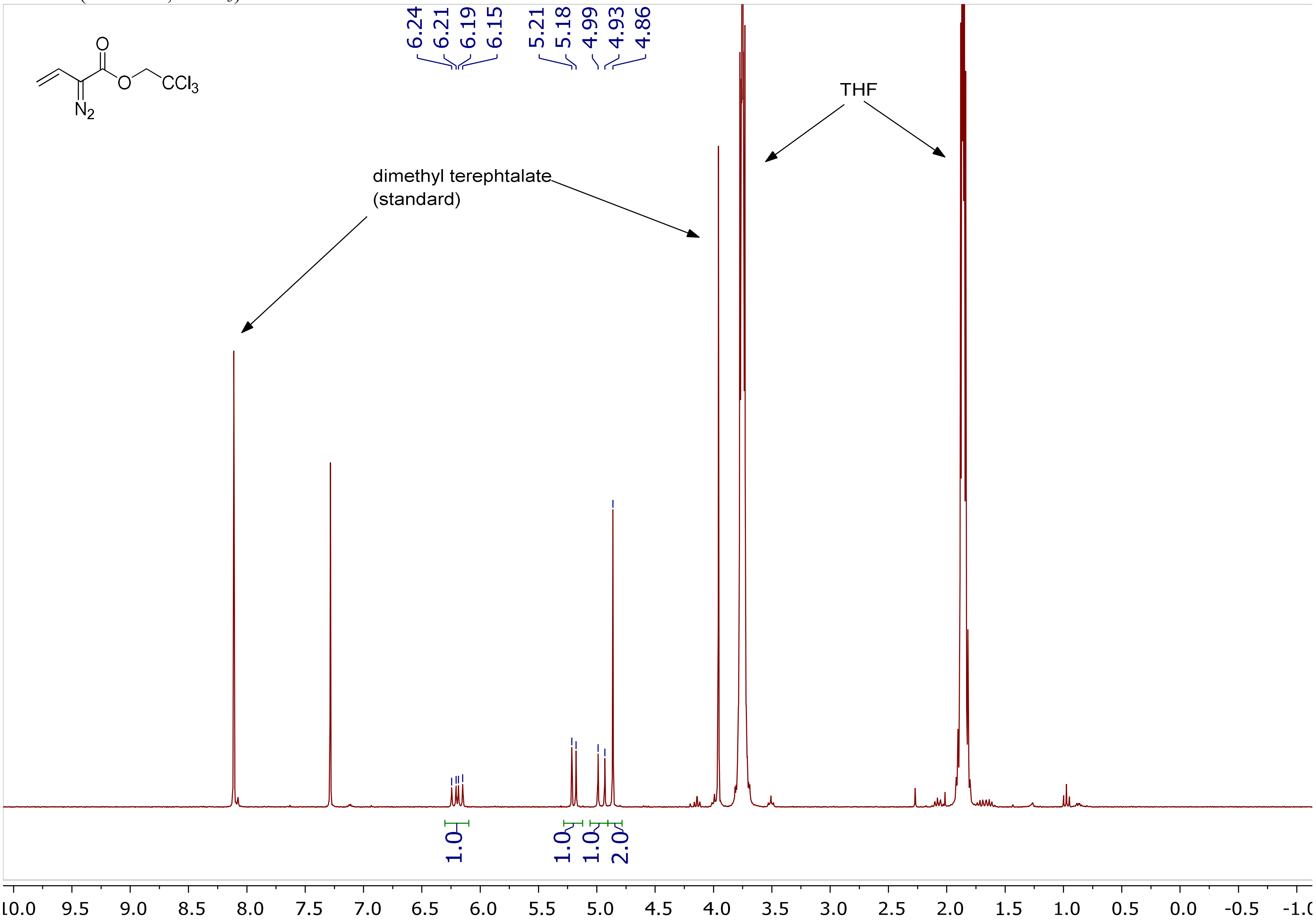

4-Nitrobenzyl 2-diazobut-3-enoate 2e

^{1}H NMR (300 MHz, $CDCl_3$)

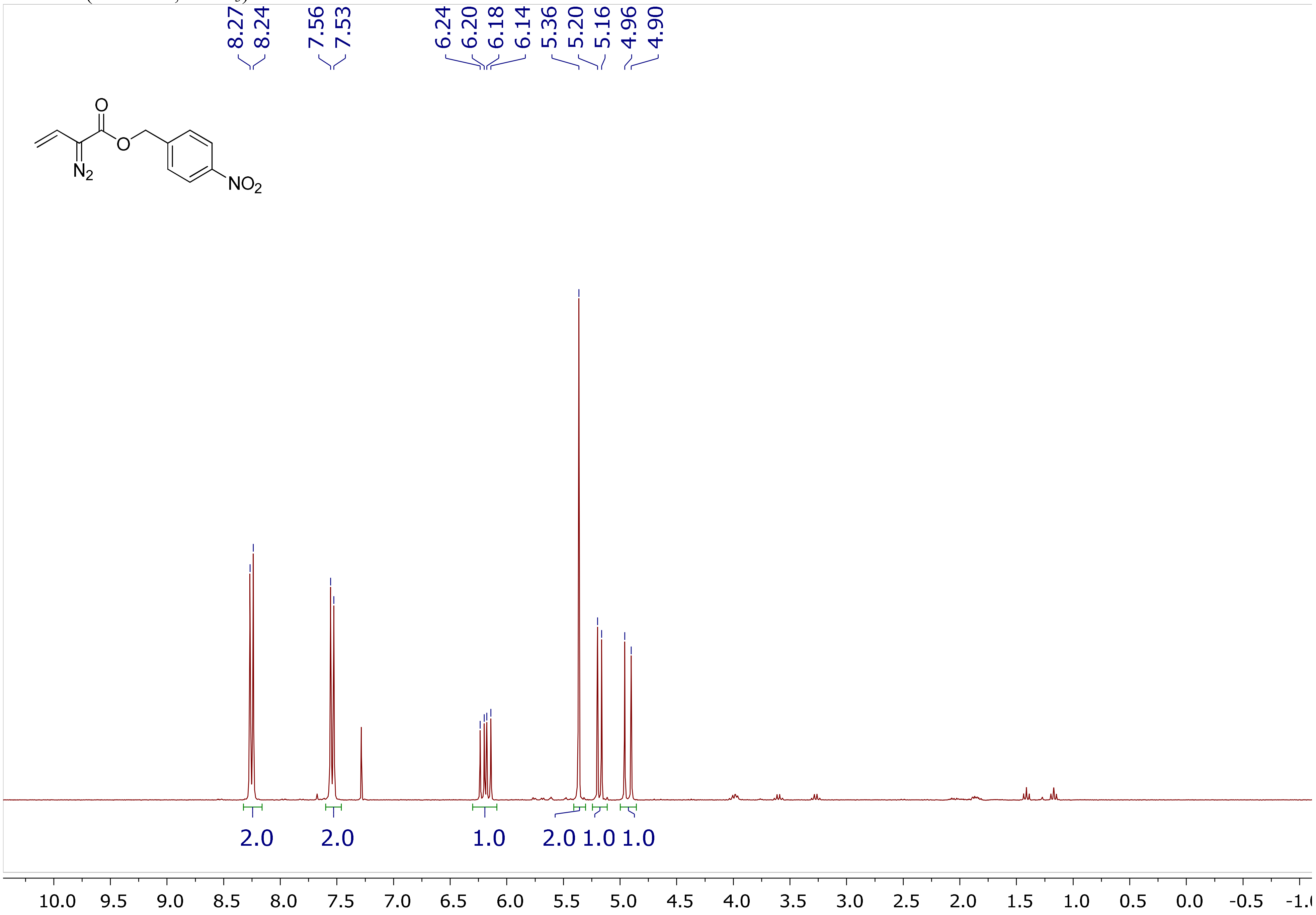

^{13}C NMR (75 MHz, $CDCl_3$)

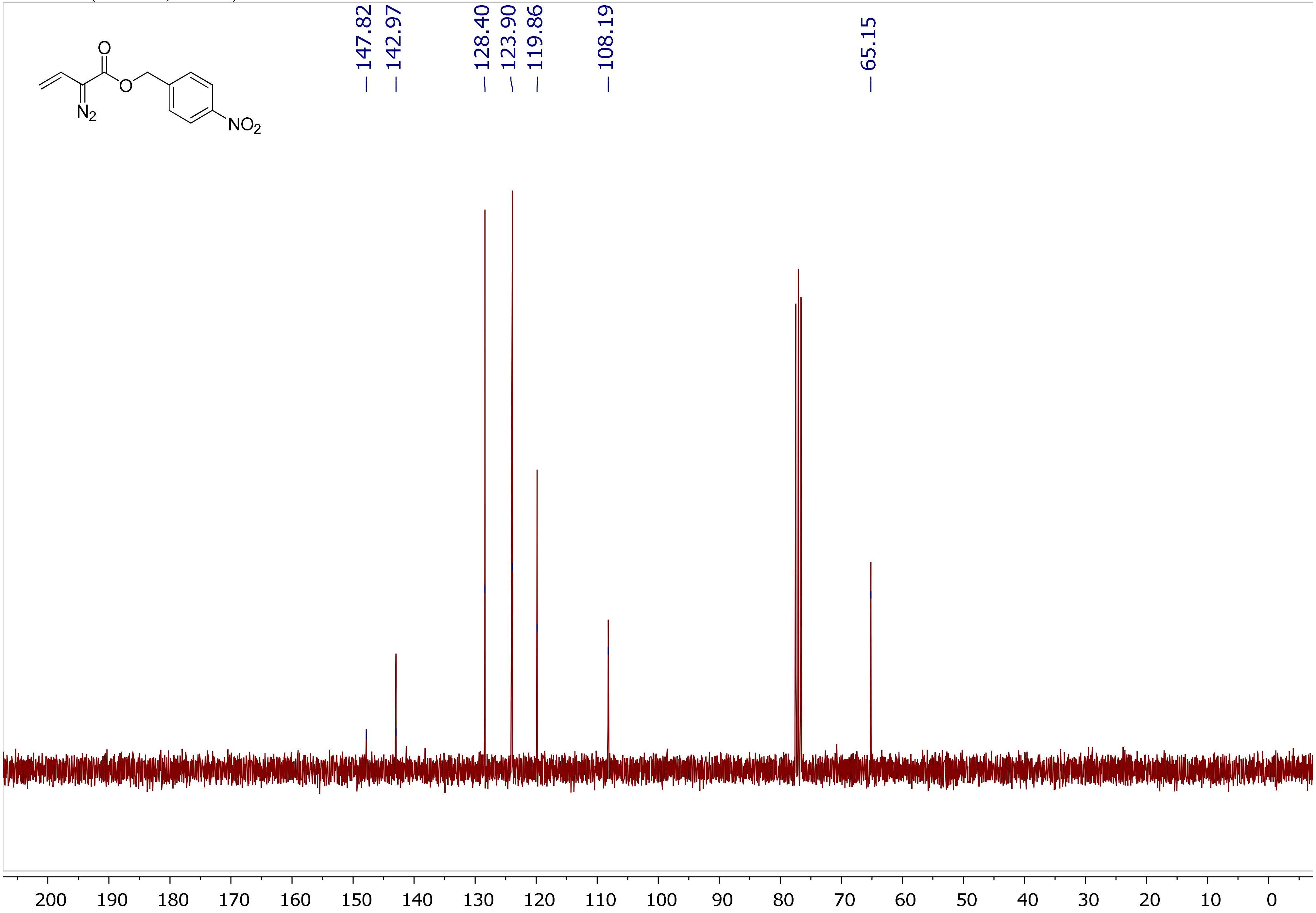

^{13}C DEPT 135 (75 MHz, $CDCl_3$)

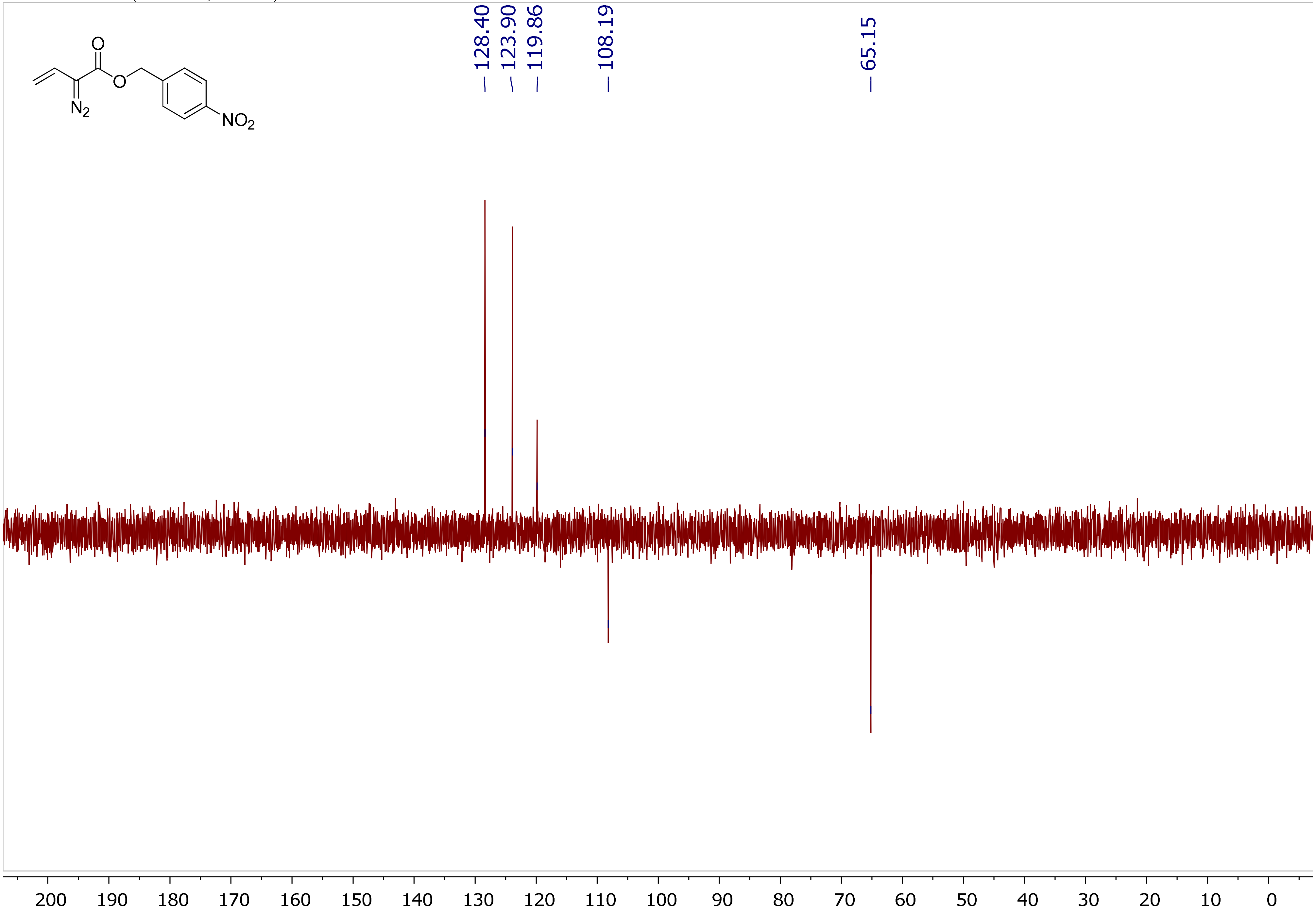

^1H-^{13}C HMBC

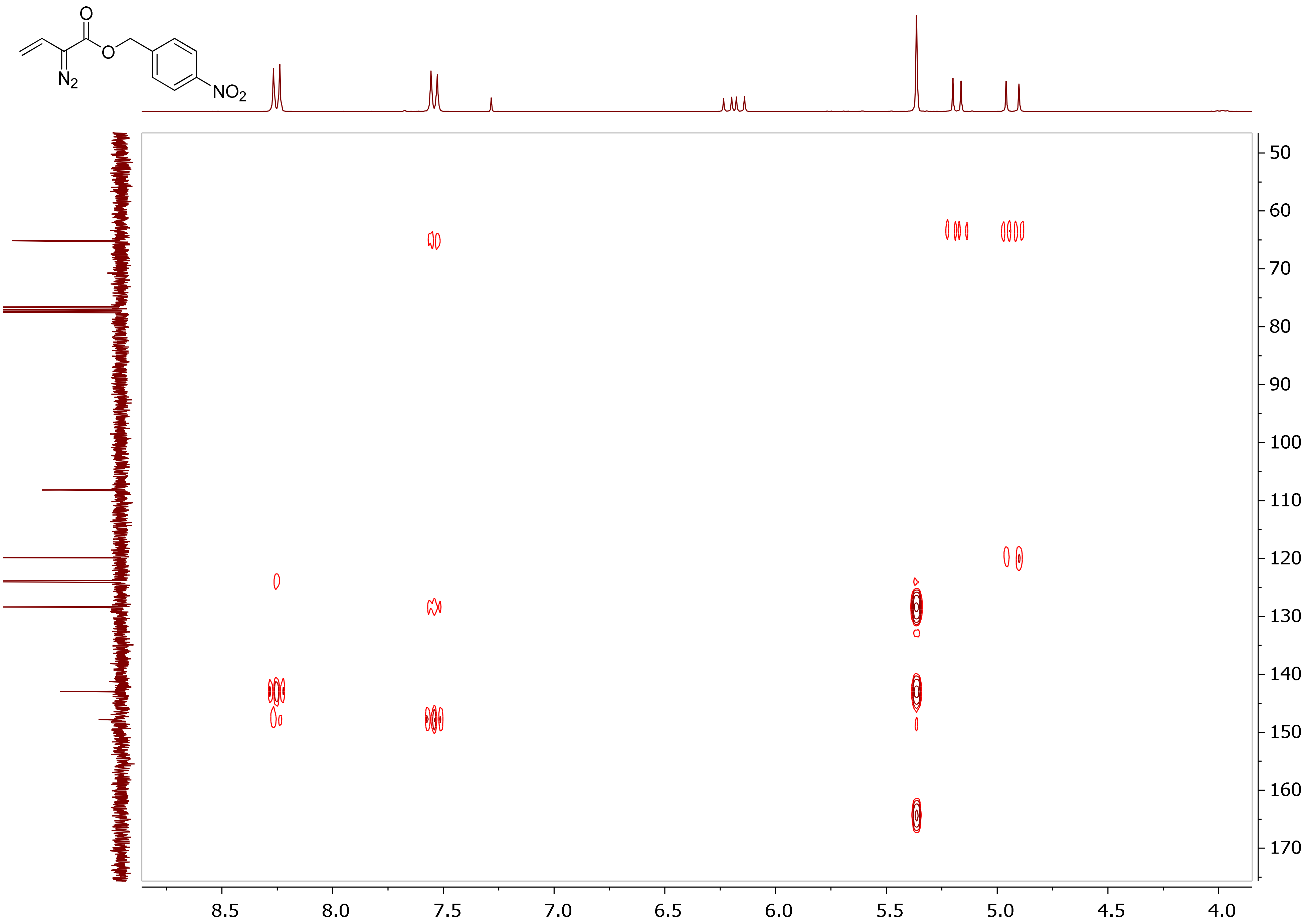
O
O
N2
NO2
50
60
70
80
90
100
110
120
130
140
150
160
170
8.5
8.0
7.5
7.0
6.5
6.0
5.5
5.0
4.5
4.0

Phenyl 2-diazobut-3-enoate 2f

^{1}H NMR (300 MHz, $CDCl_3$)

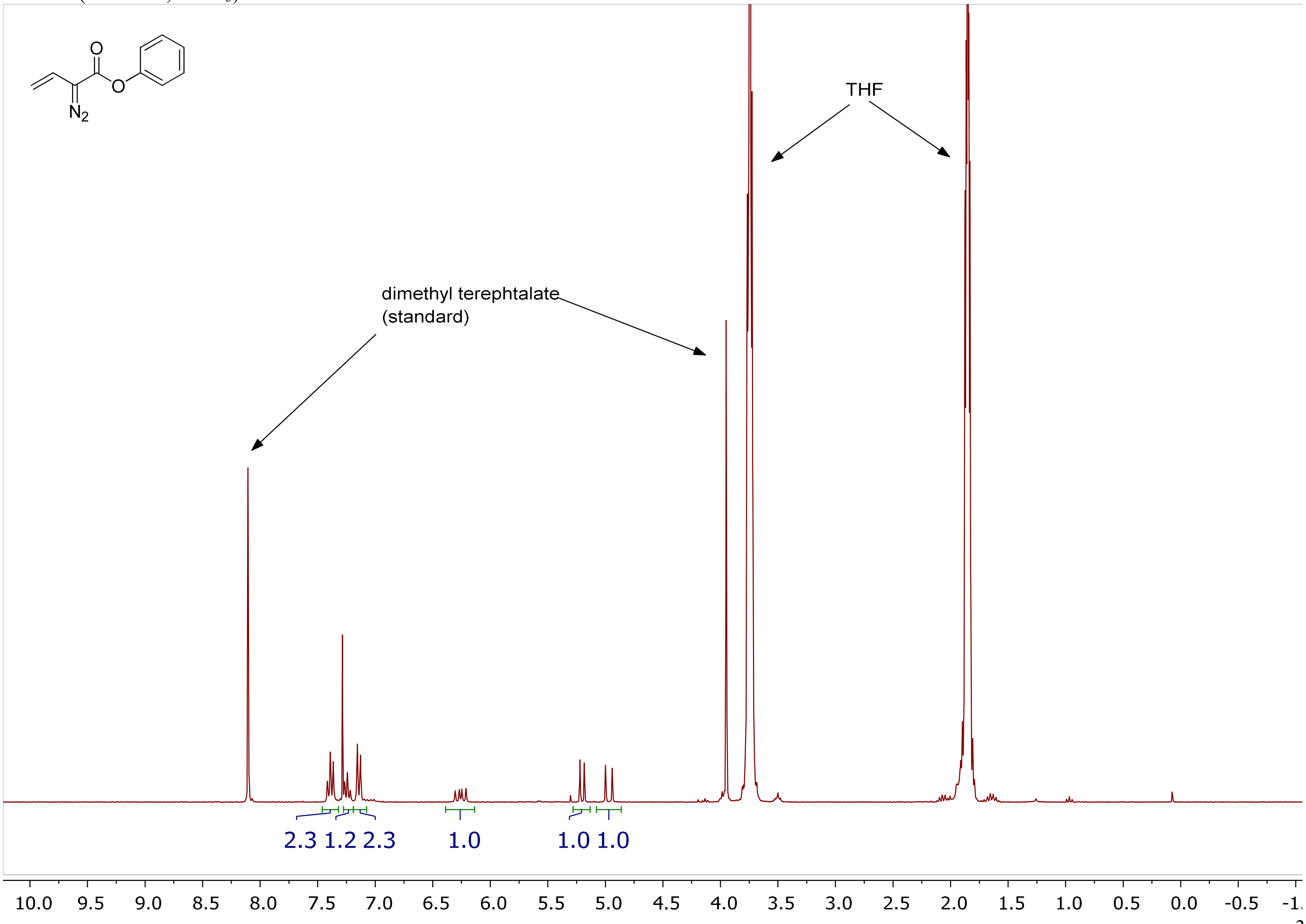

2,2,2-Trichloroethyl (4aR*,5S*)-5-(4-methoxyphenyl)-7,7-dimethyl-4a,5,6,7-tetrahydro-2H-[1,2]oxazino[2,3-b][1,2]oxazine-4-carboxylate 3a
^{1}H NMR (300 MHz, $CDCl_3$)

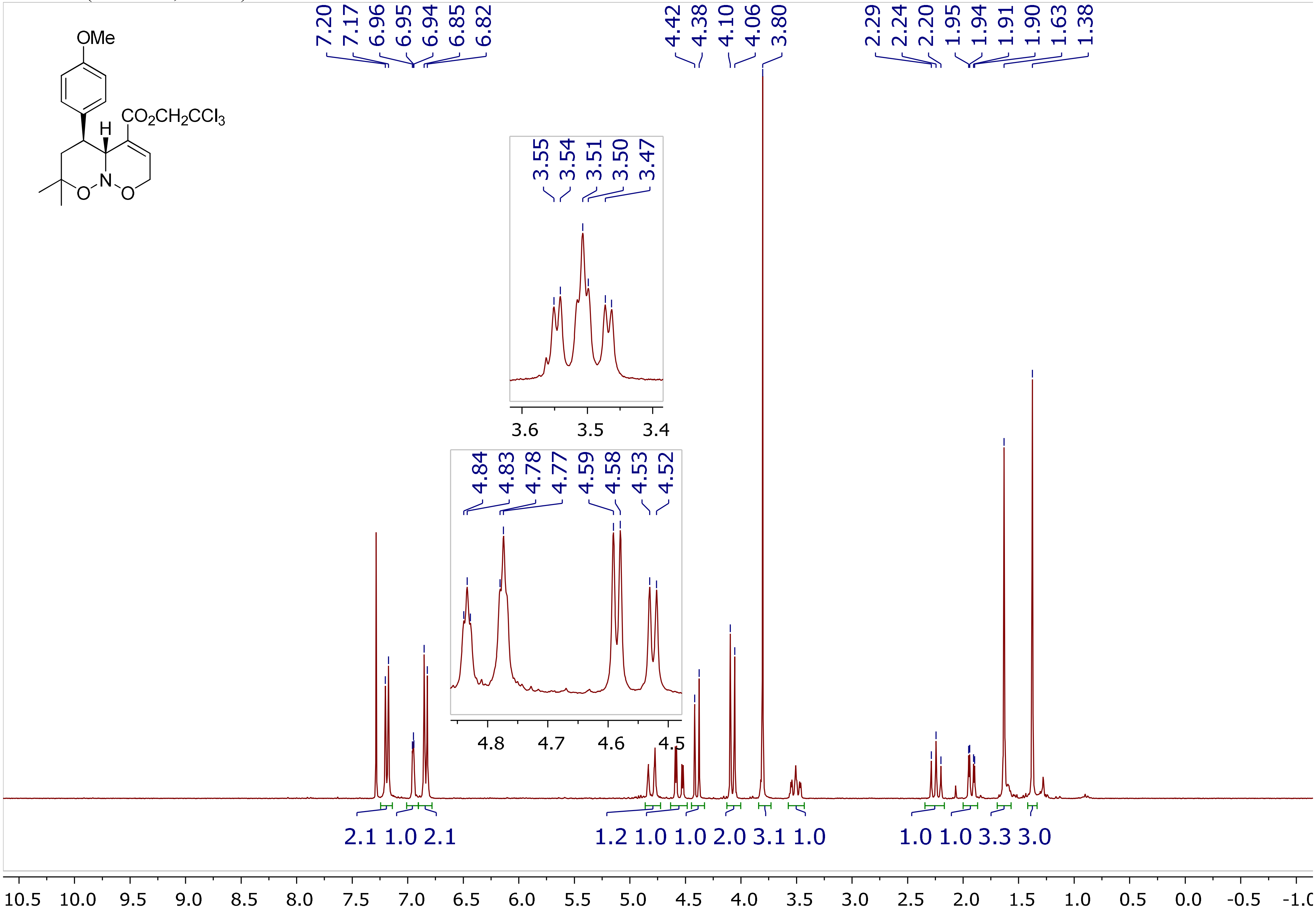

^{13}C NMR (75 MHz, $CDCl_3$)

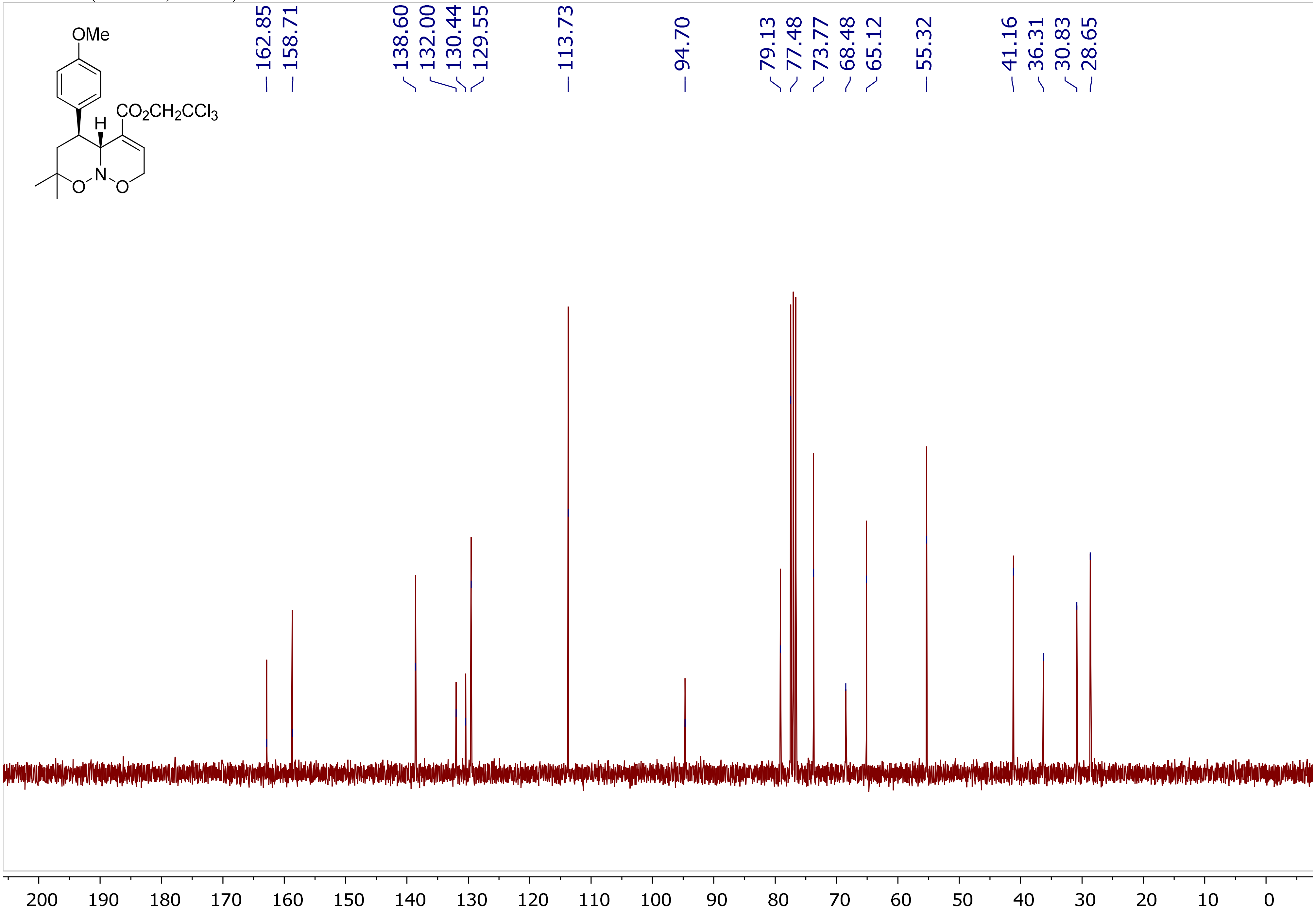

^{13}C DEPT 135 (75 MHz, $CDCl_3$)

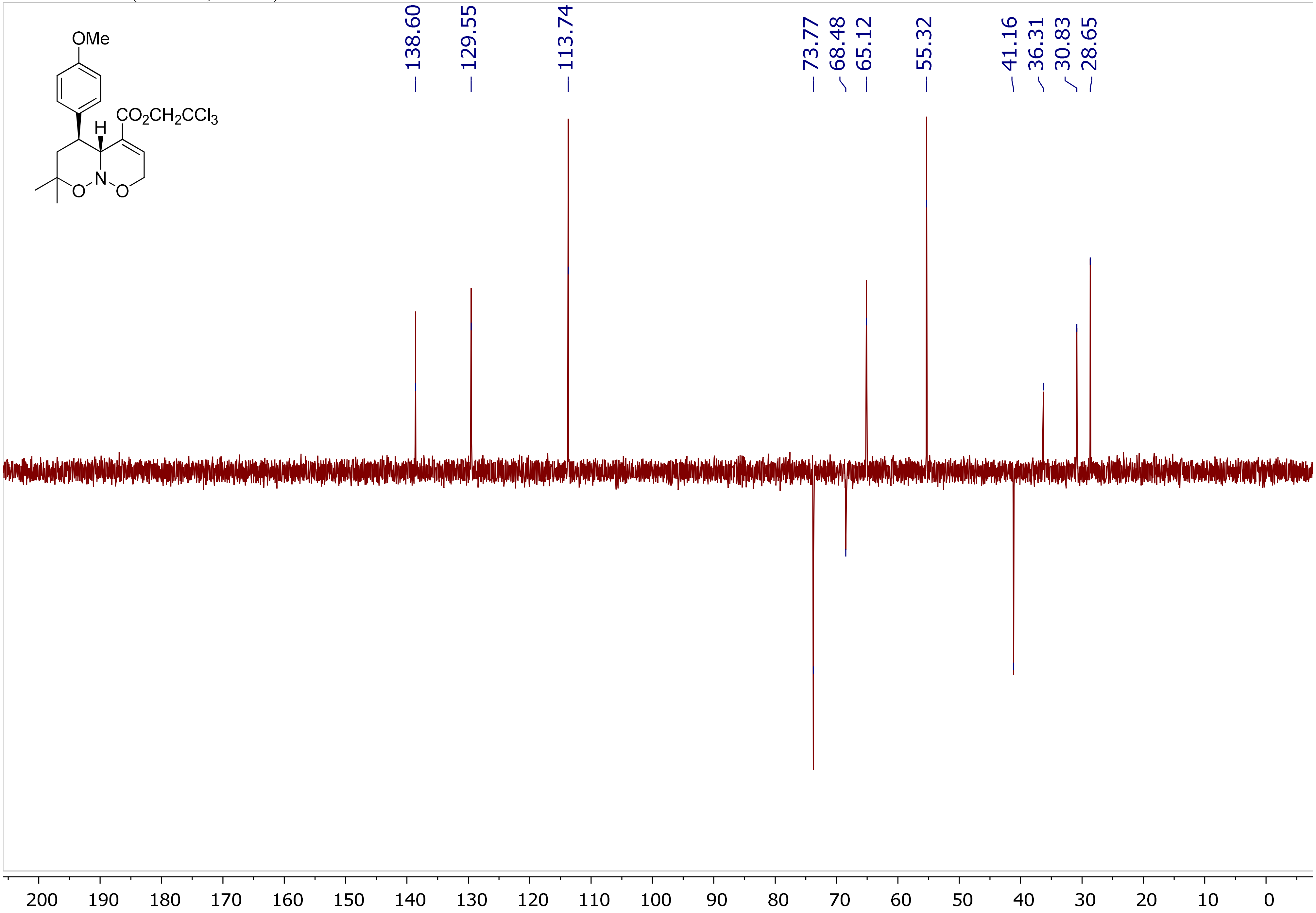

Methyl (4aR*,5S*)-5-(4-methoxyphenyl)-7,7-dimethyl-4a,5,6,7-tetrahydro-2H-[1,2]oxazino[2,3-b][1,2]oxazine-4-carboxylate 3b

^{1}H NMR (300 MHz, $CDCl_3$)

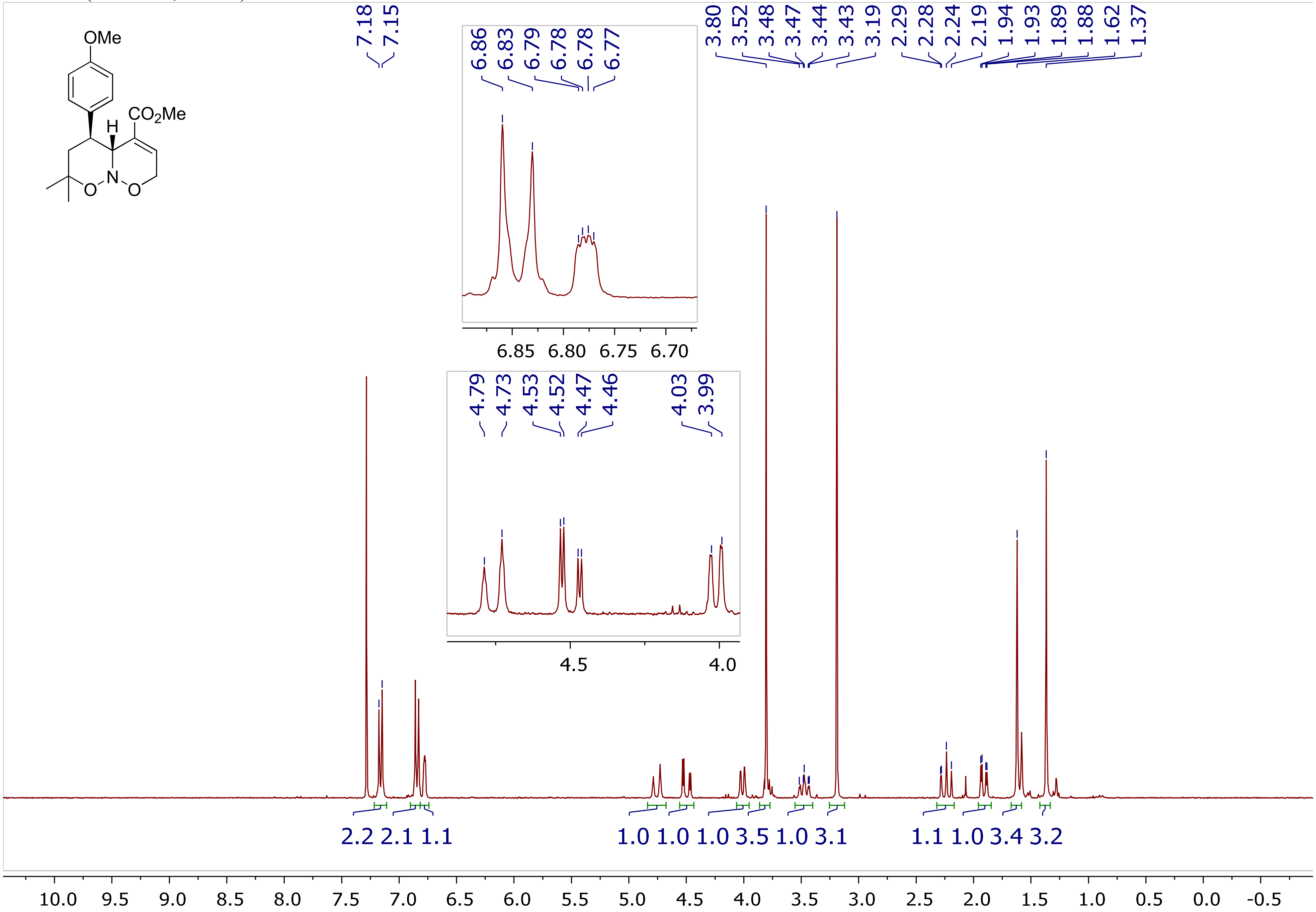

^{13}C NMR (75 MHz, $CDCl_3$)

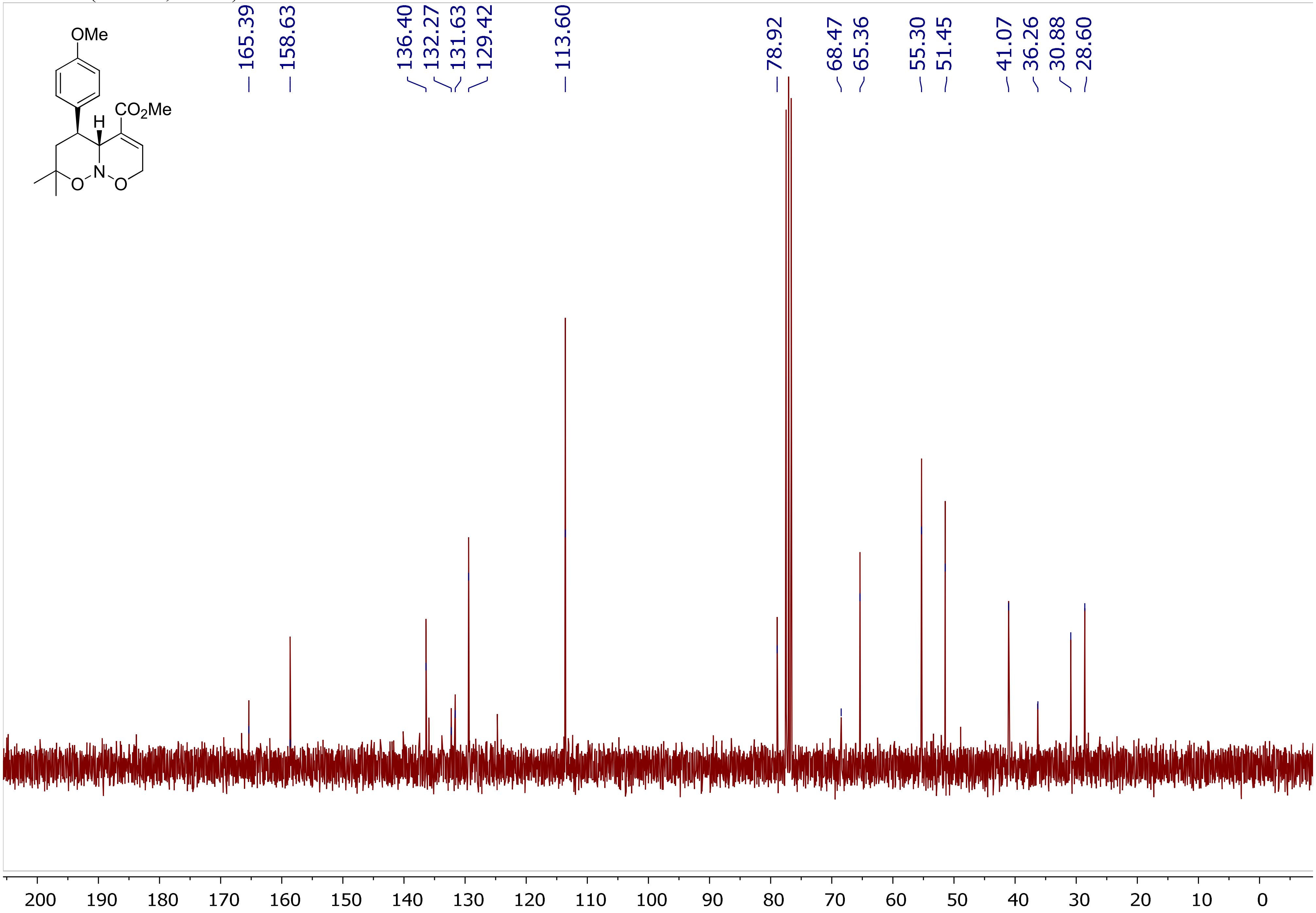

^{13}C DEPT 135 (75 MHz, $CDCl_3$)

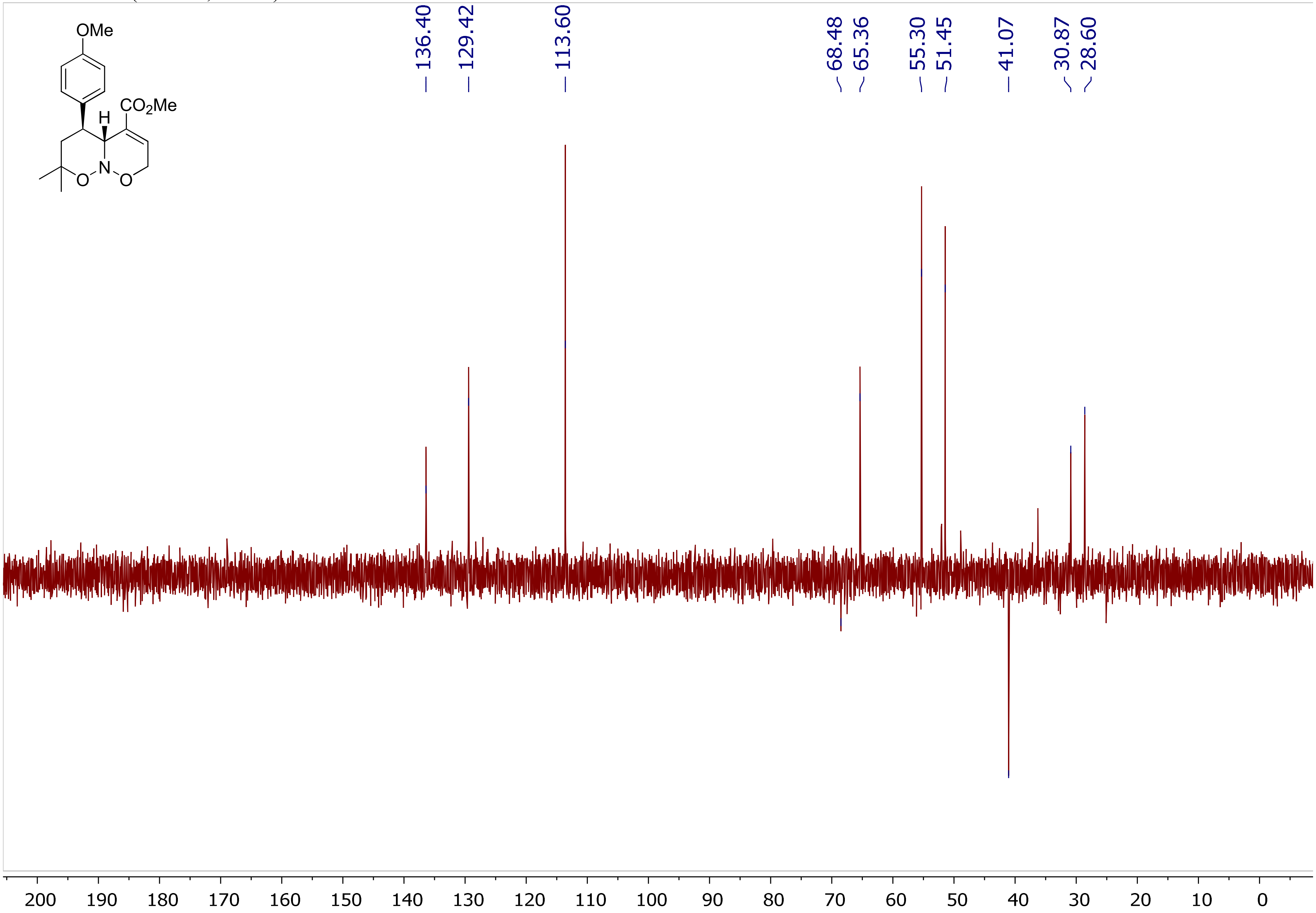

^{1}H-^{1}H COSY

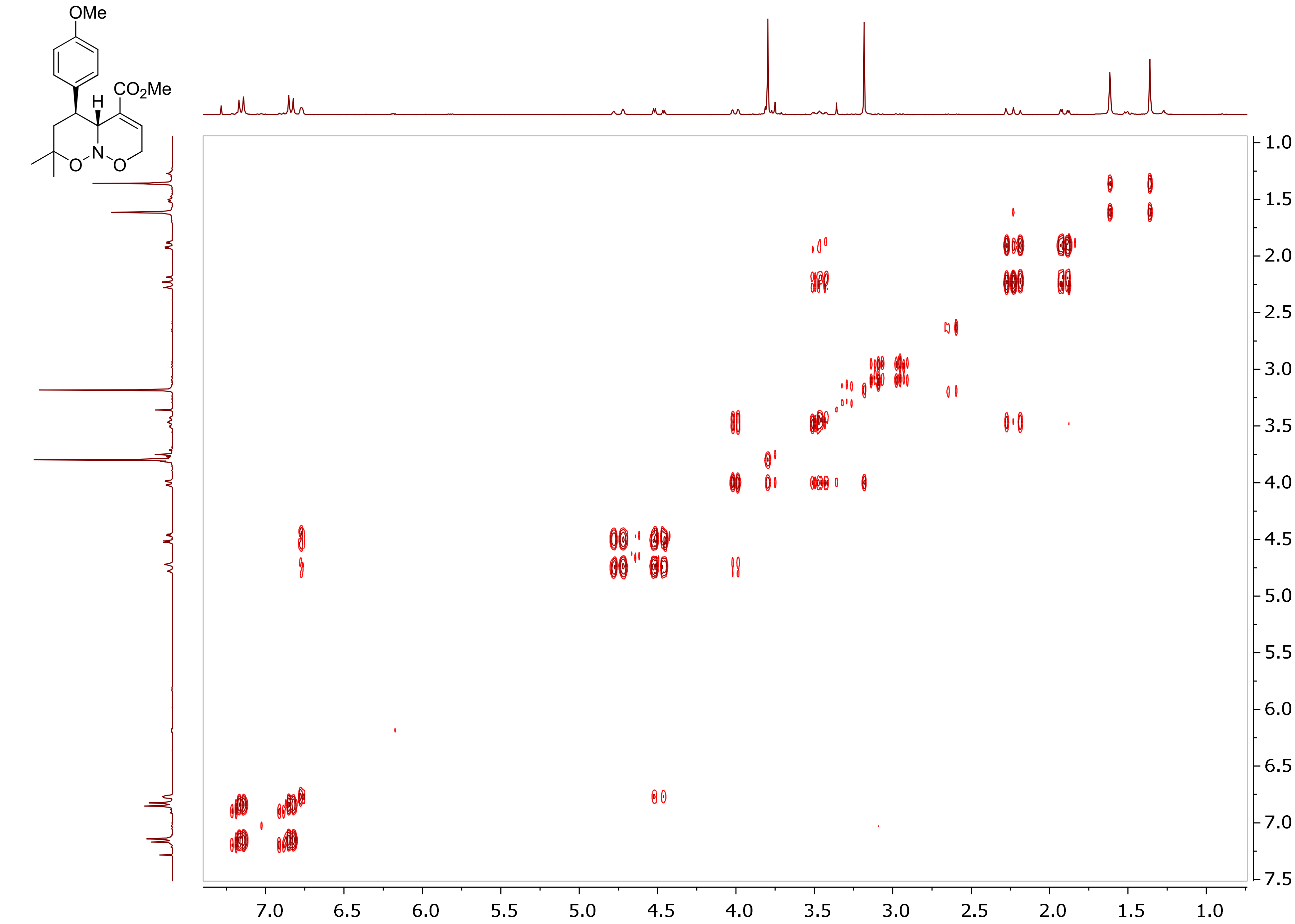
OMe
CO2Me
H
N
O
O
1.0
1.5
2.0
2.5
3.0
3.5
4.0
4.5
5.0
5.5
6.0
6.5
7.0
7.5
7.0
6.5
6.0
5.5
5.0
4.5
4.0
3.5
3.0
2.5
2.0
1.5
1.0

^{1}H-^{13}C HSQC

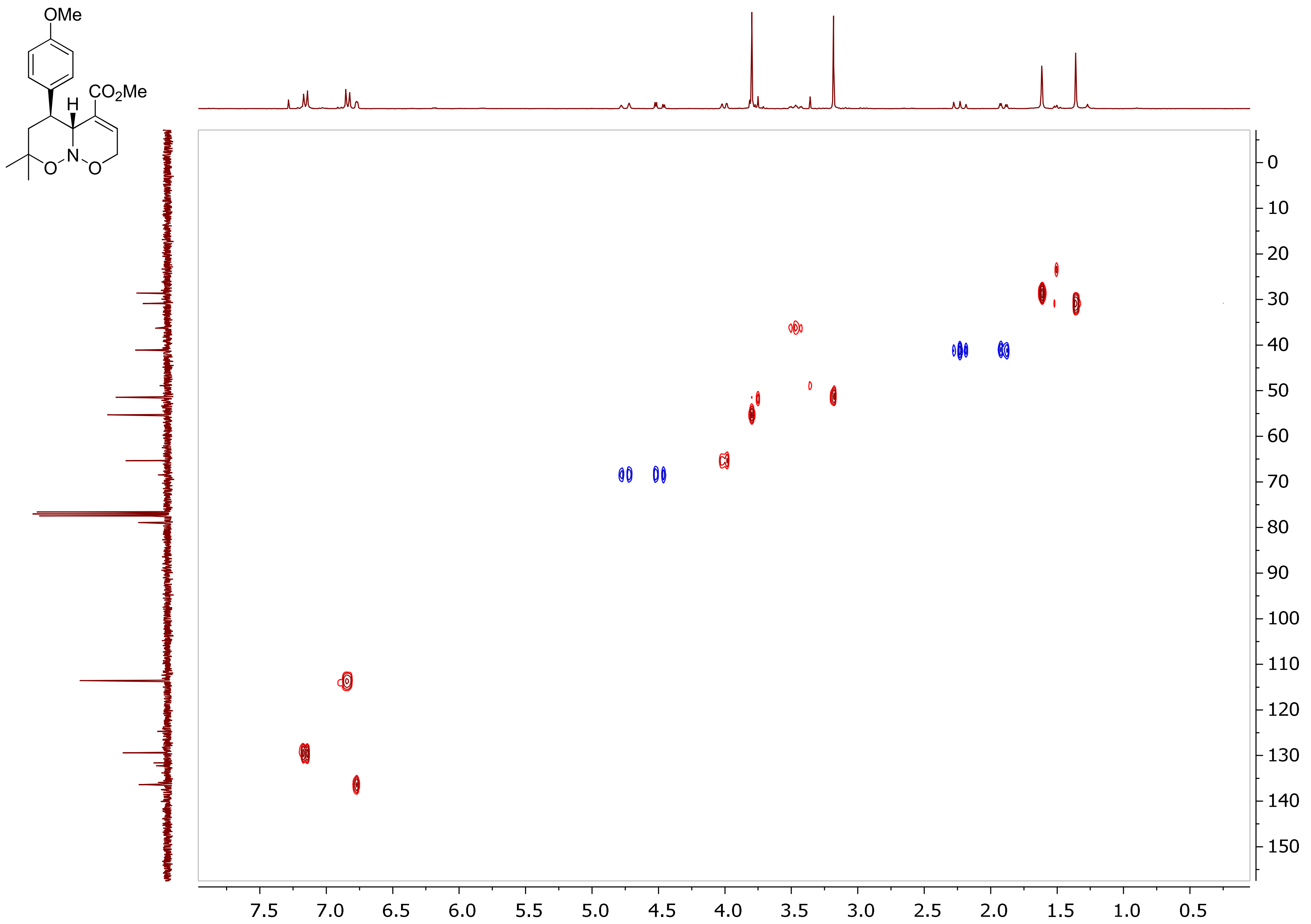
OMe
CO2Me
H
N
O
O
0
10
20
30
40
50
60
70
80
90
100
110
120
130
140
150
7.5
7.0
6.5
6.0
5.5
5.0
4.5
4.0
3.5
3.0
2.5
2.0
1.5
1.0
0.5

^{1}H-^{13}C HMBC

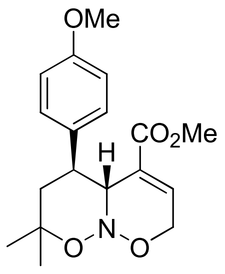

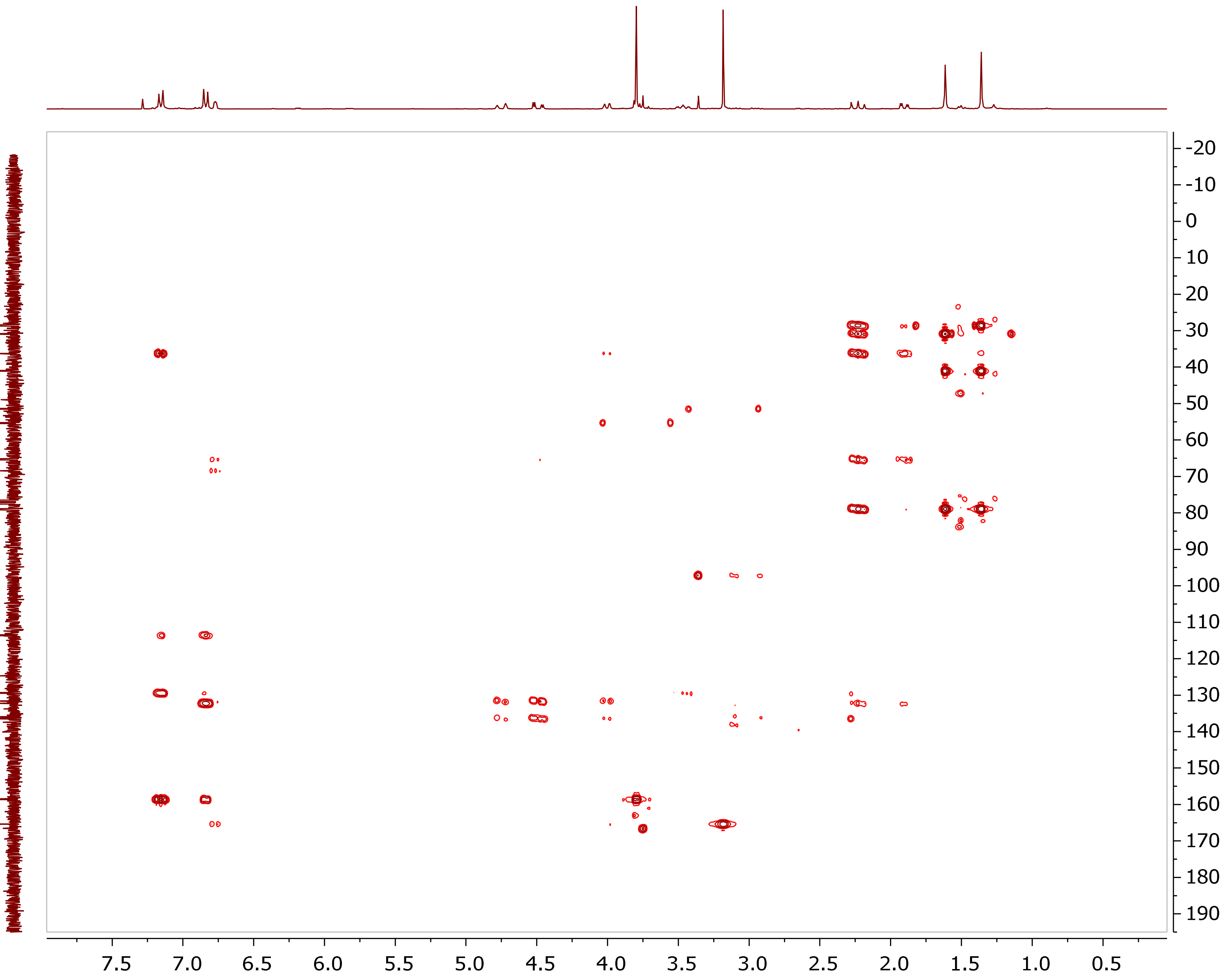

-20
-10
0
10
20
30
40
50
60
70
80
90
100
110
120
130
140
150
160
170
180
190
7.5
7.0
6.5
6.0
5.5
5.0
4.5
4.0
3.5
3.0
2.5
2.0
1.5
1.0
0.5

^{1}H-^{1}H NOESY

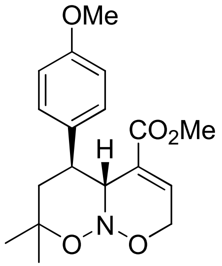
OMe
H
CO2Me
N
O
O

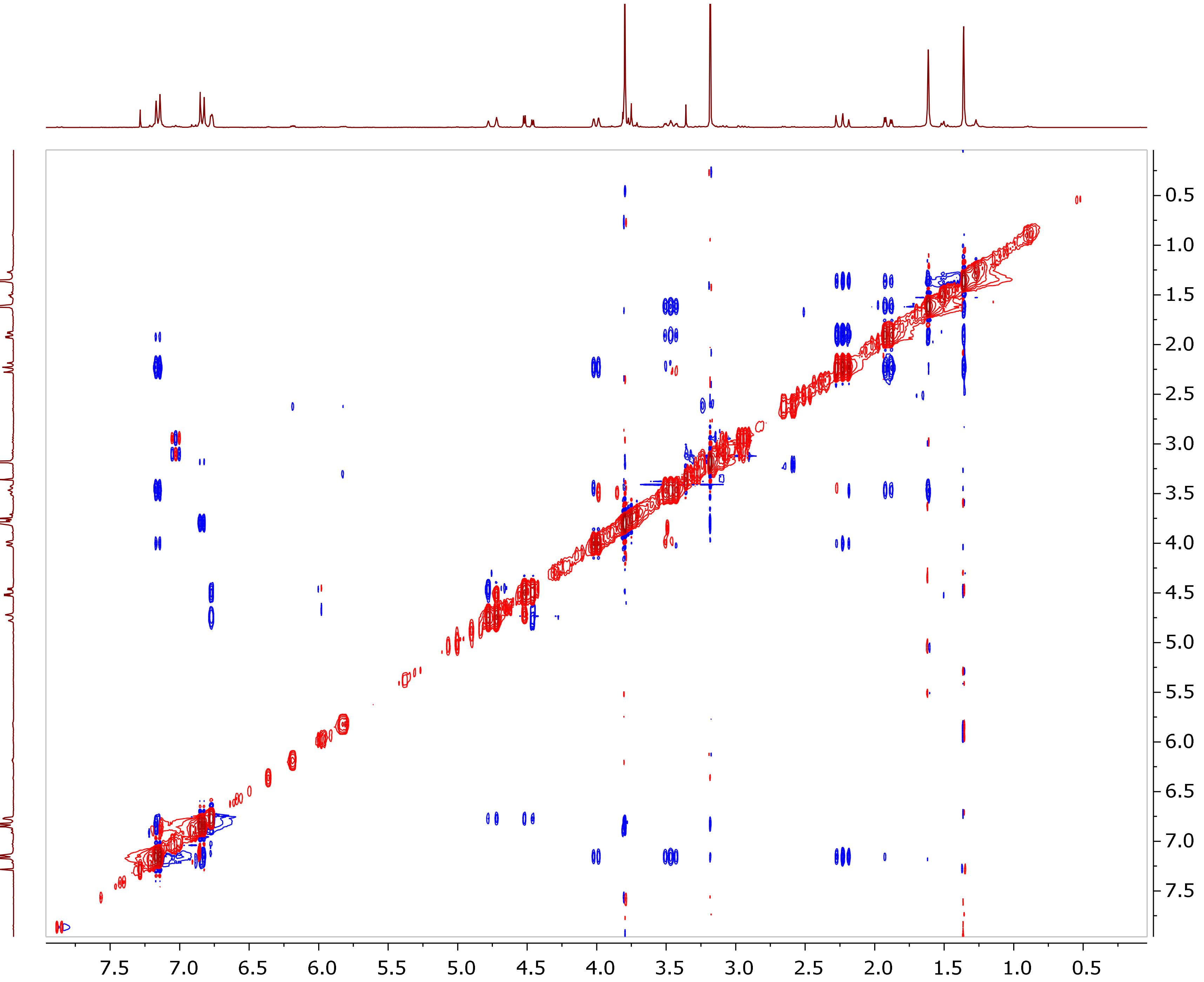
0.5
1.0
1.5
2.0
2.5
3.0
3.5
4.0
4.5
5.0
5.5
6.0
6.5
7.0
7.5
7.5
7.0
6.5
6.0
5.5
5.0
4.5
4.0
3.5
3.0
2.5
2.0
1.5
1.0
0.5

4-Ethyl (4aR*,5S*)-5-(4-methoxyphenyl)-7,7-dimethyl-4a,5,6,7-tetrahydro-2H-[1,2]oxazino[2,3-b][1,2]oxazine-4-carboxylate 3c

^{1}H NMR (300 MHz, $CDCl_3$)

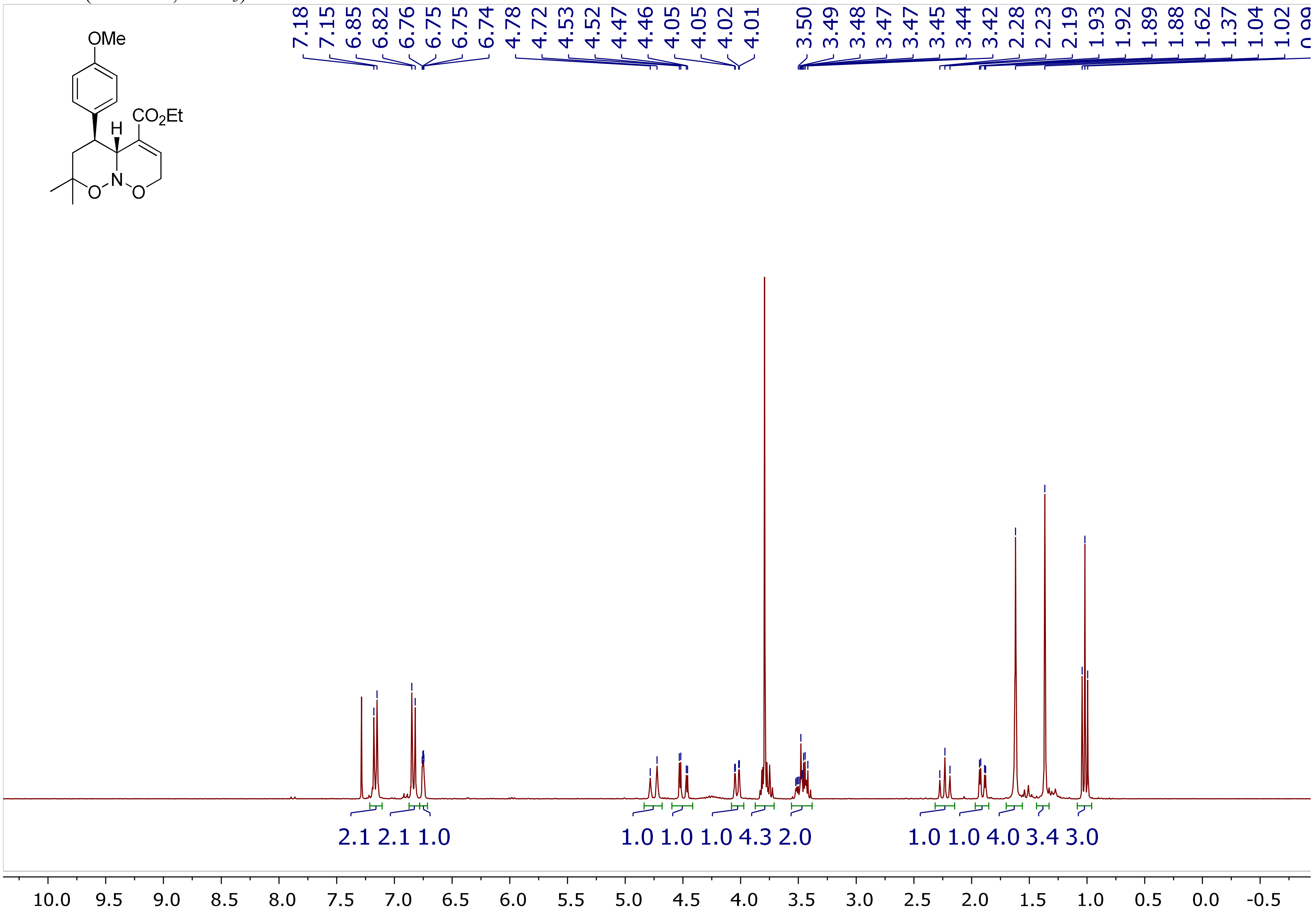

^{13}C NMR (75 MHz, $CDCl_3$)

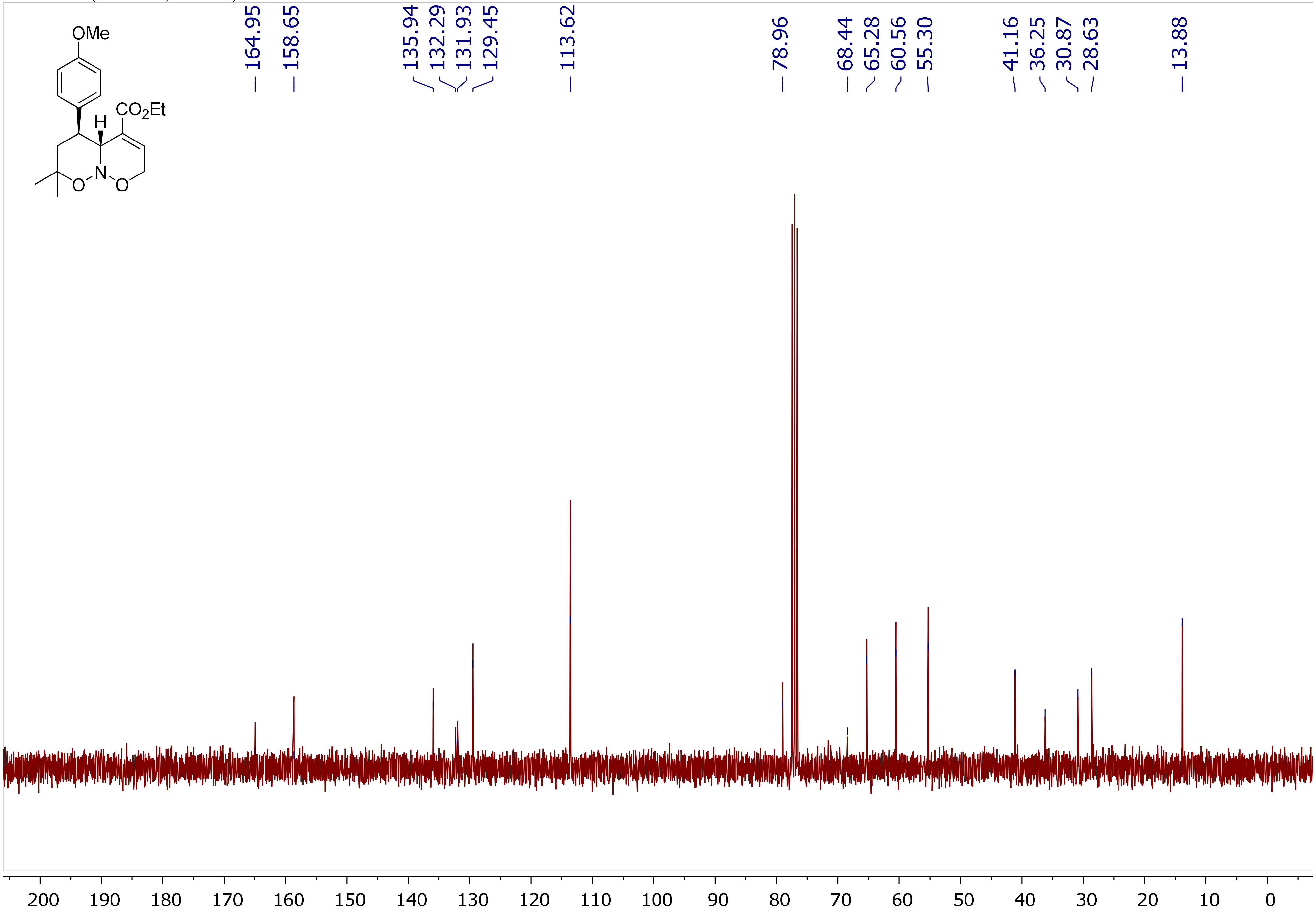

^{13}C DEPT 135 (75 MHz, $CDCl_3$)

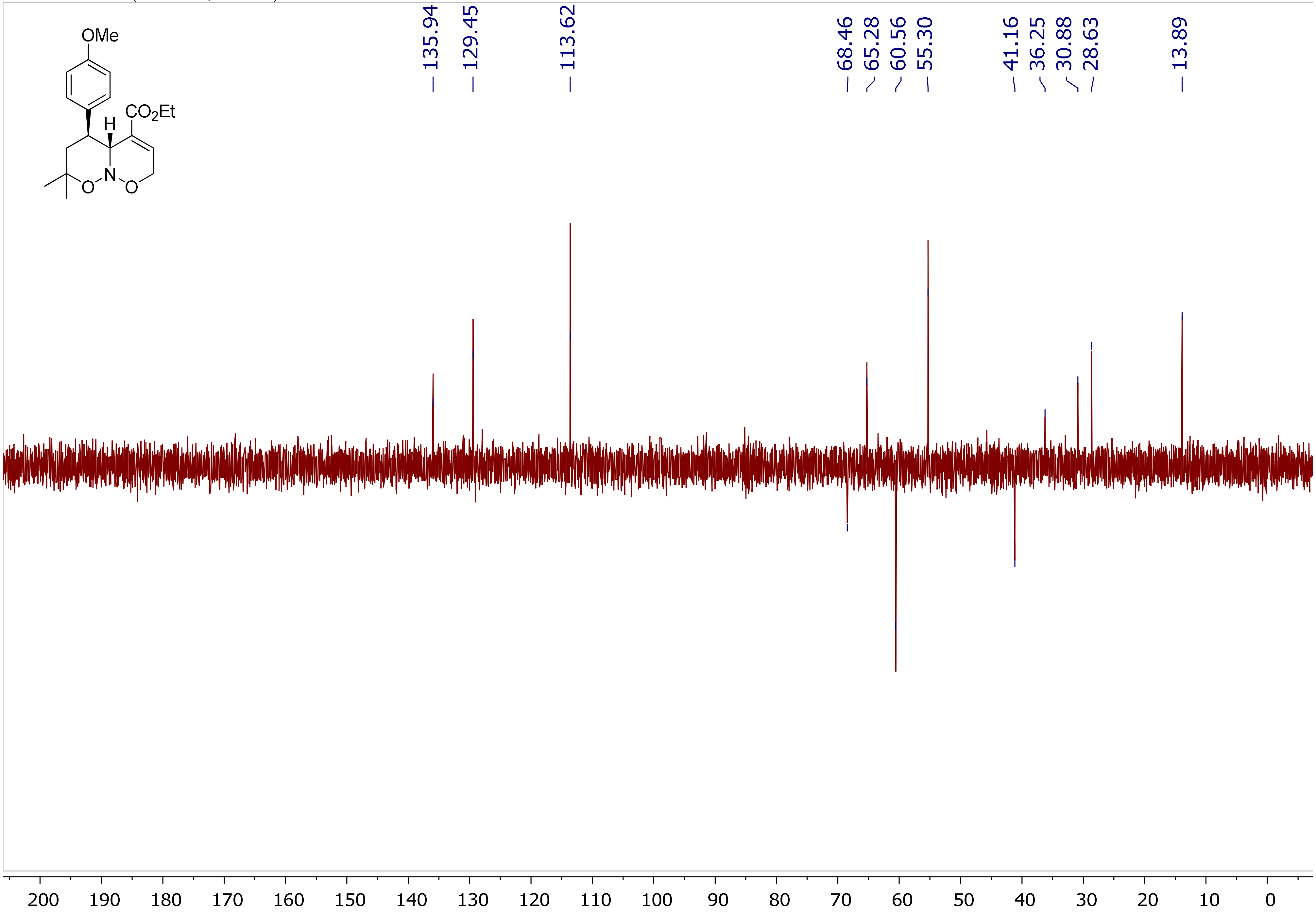

Benzyl (4aR*,5S*)-5-(4-methoxyphenyl)-7,7-dimethyl-4a,5,6,7-tetrahydro-2H-[1,2]oxazino[2,3-b][1,2]oxazine-4-carboxylate 3d
^{1}H NMR (300 MHz, $CDCl_3$)

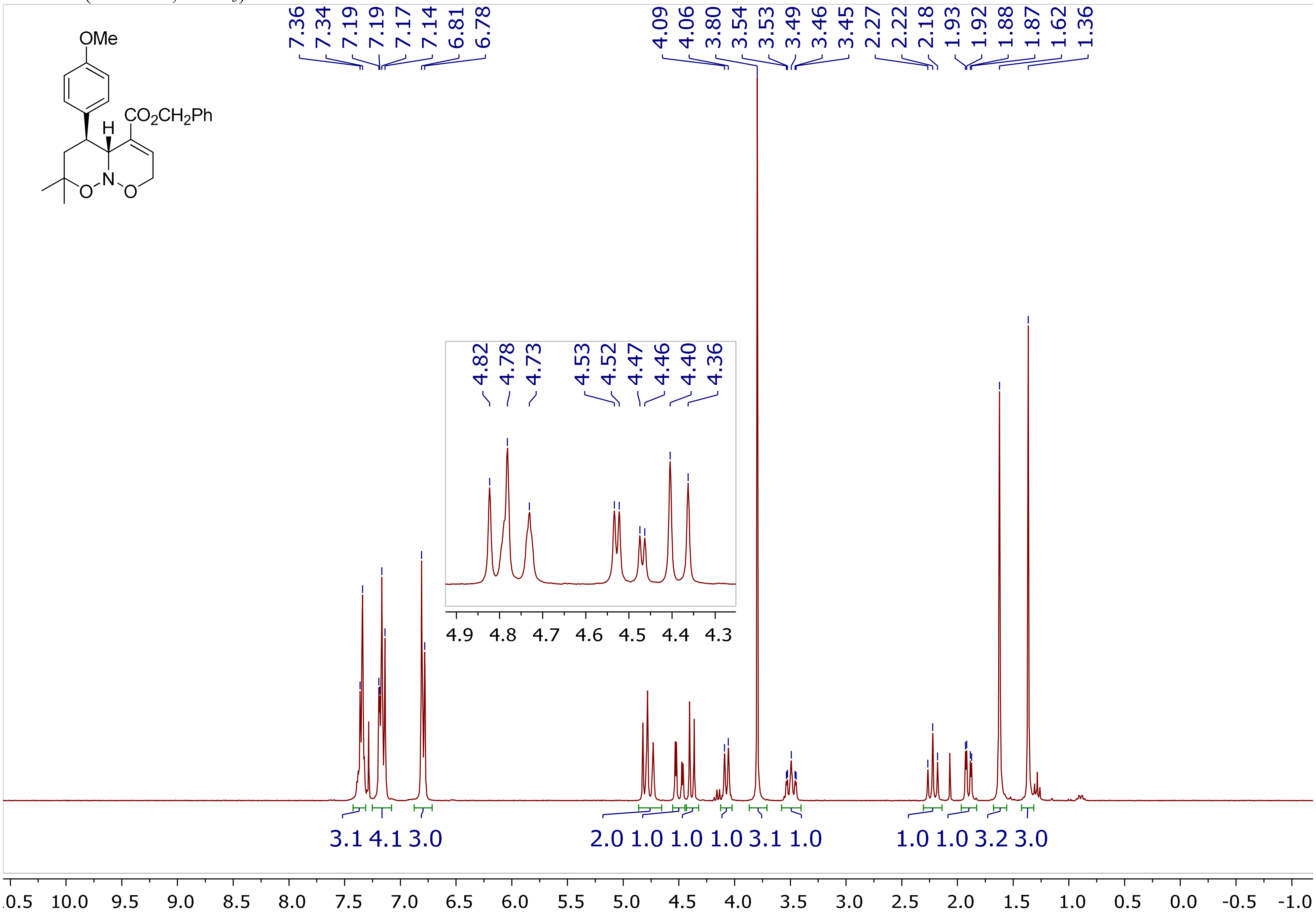

^{13}C NMR (75 MHz, $CDCl_3$)

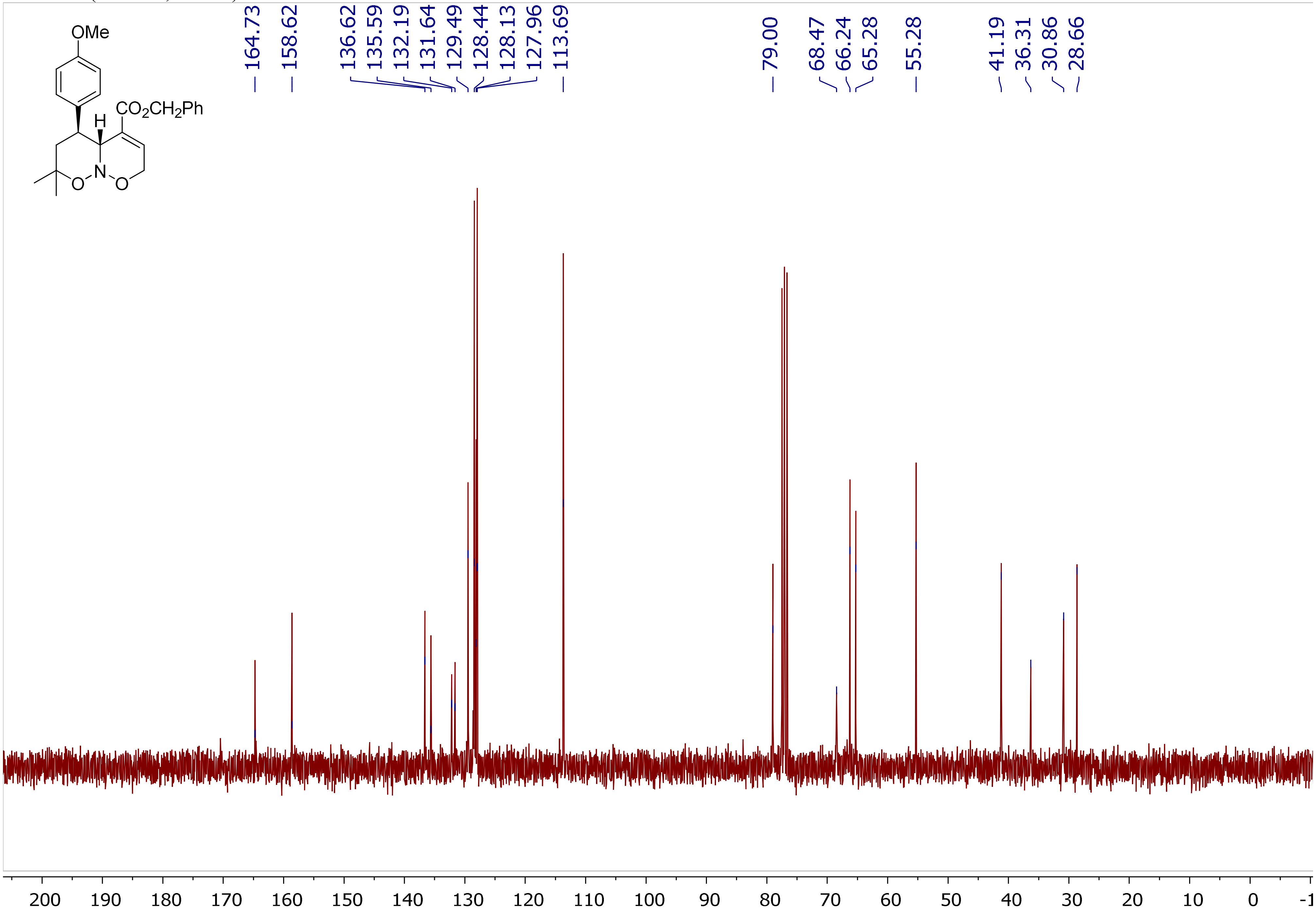

^{13}C DEPT 135 (75 MHz, $CDCl_3$)

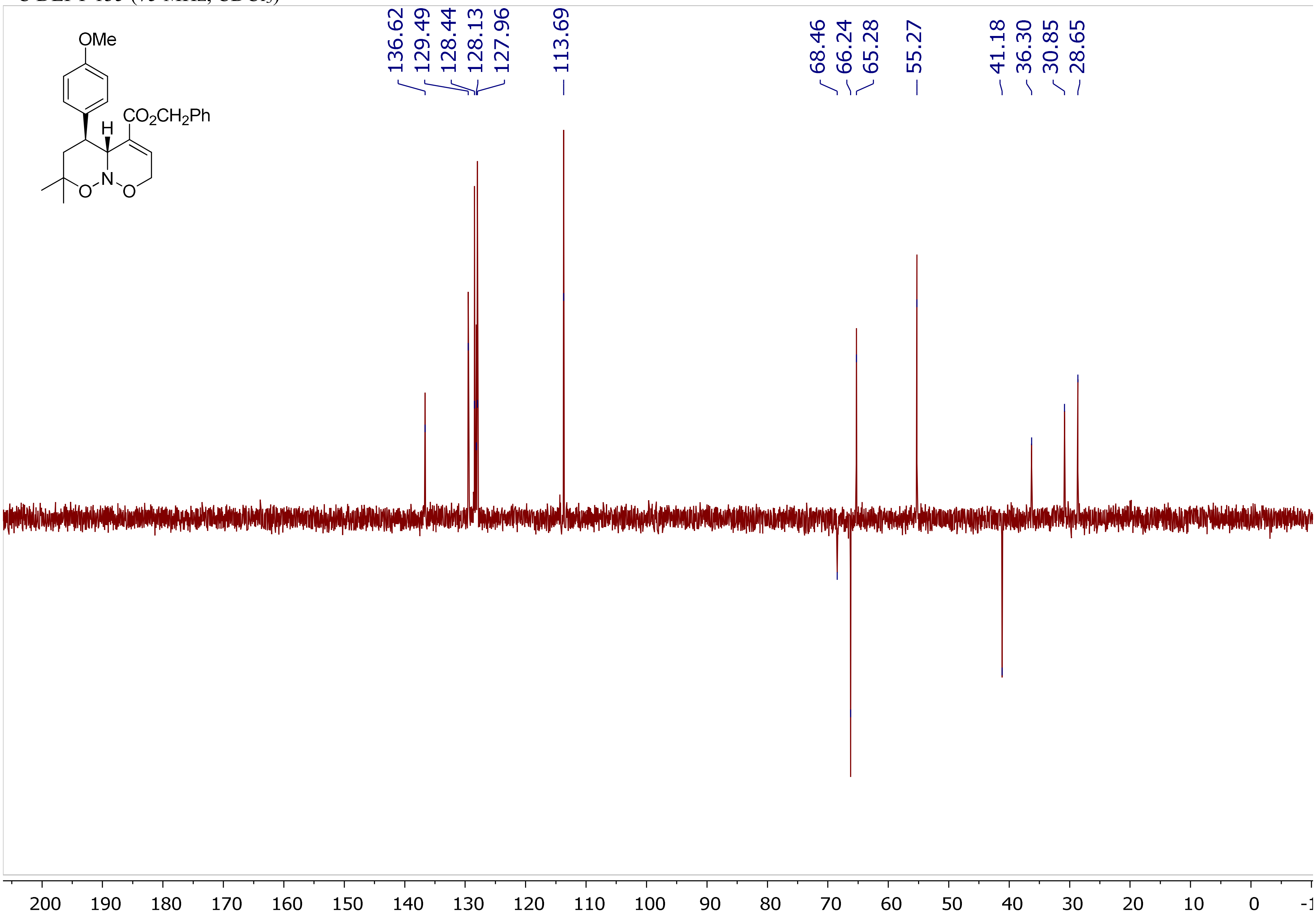

4-Nitrobenzyl (4aR*,5S*)-5-(4-methoxyphenyl)-7,7-dimethyl-4a,5,6,7-tetrahydro-2H-[1,2]oxazino[2,3-b][1,2]oxazine-4-carboxylate 3e

^{1}H NMR (300 MHz, $CDCl_3$)

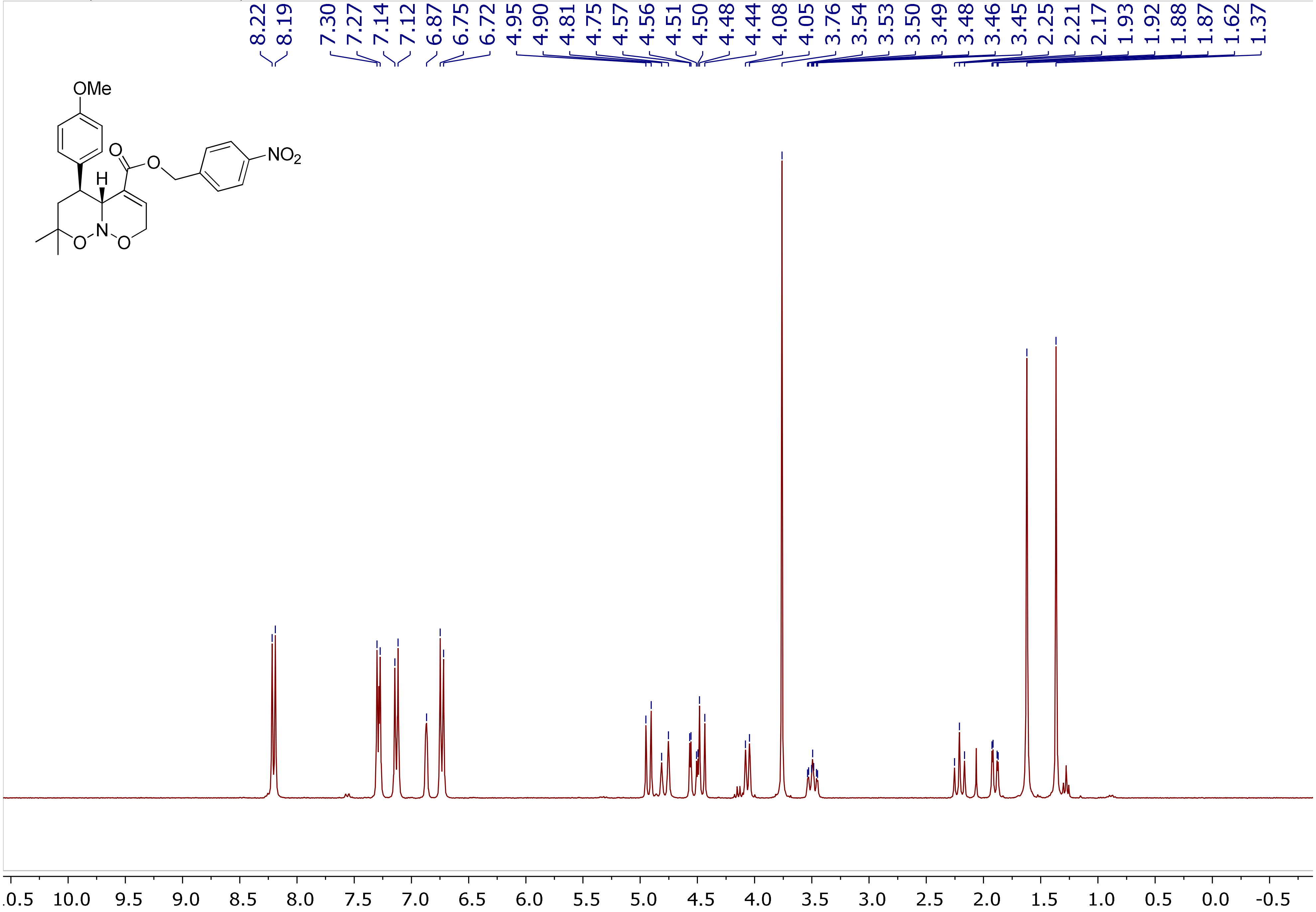

^{13}C NMR (75 MHz, $CDCl_3$)

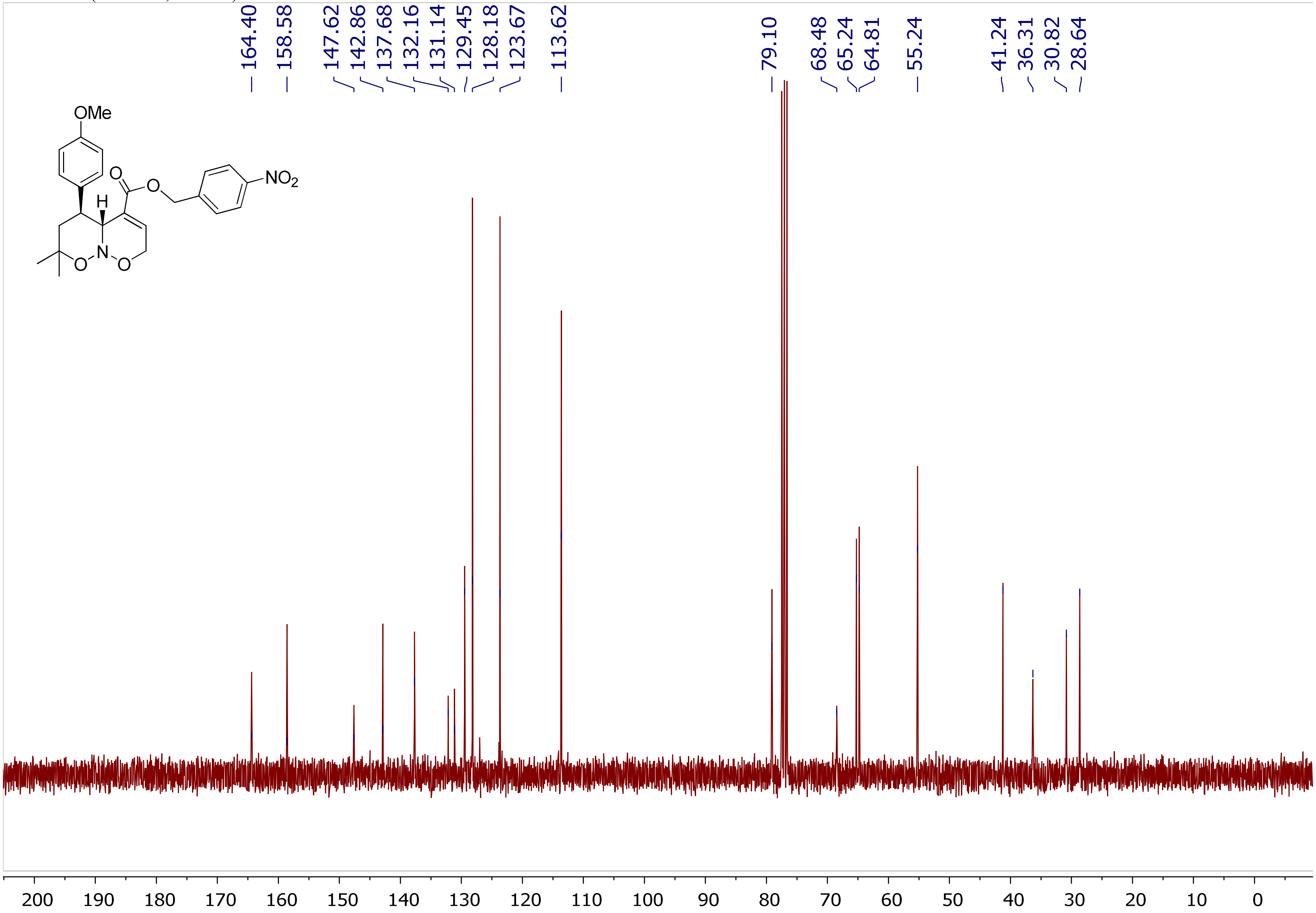

^{13}C DEPT 135 (75 MHz, $CDCl_3$)

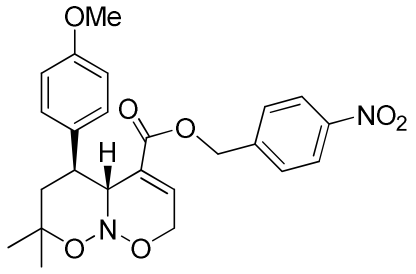

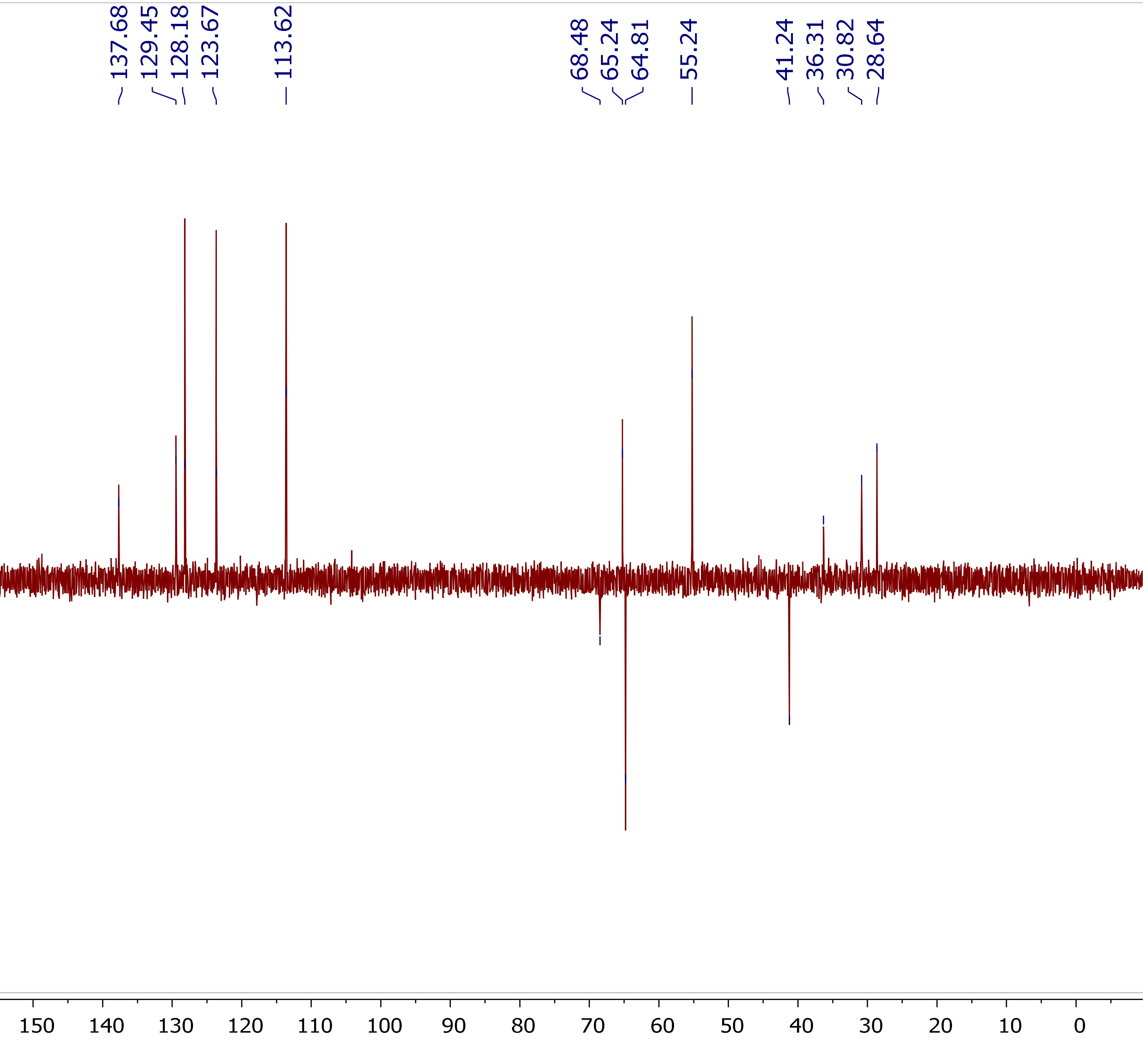

^{1}H-^{13}C HSQC

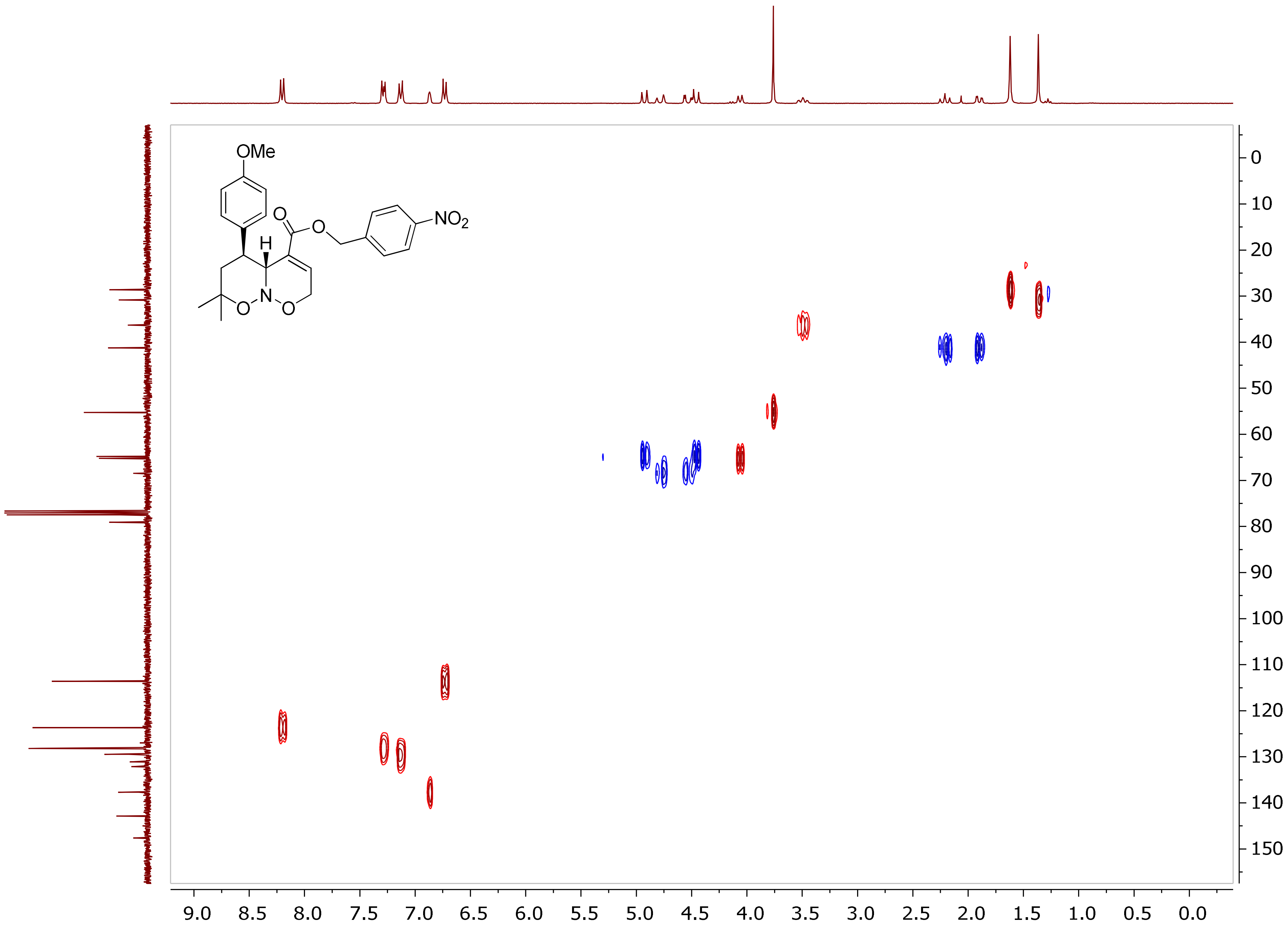

OMe
O
O
NO2
H
N
O
O
9.0
8.5
8.0
7.5
7.0
6.5
6.0
5.5
5.0
4.5
4.0
3.5
3.0
2.5
2.0
1.5
1.0
0.5
0.0
0
10
20
30
40
50
60
70
80
90
100
110
120
130
140
150

Phenyl (4aR*,5S*)-5-(4-methoxyphenyl)-7,7-dimethyl-4a,5,6,7-tetrahydro-2H-[1,2]oxazino[2,3-b][1,2]oxazine-4-carboxylate 3f

^{1}H NMR (300 MHz, $CDCl_3$)

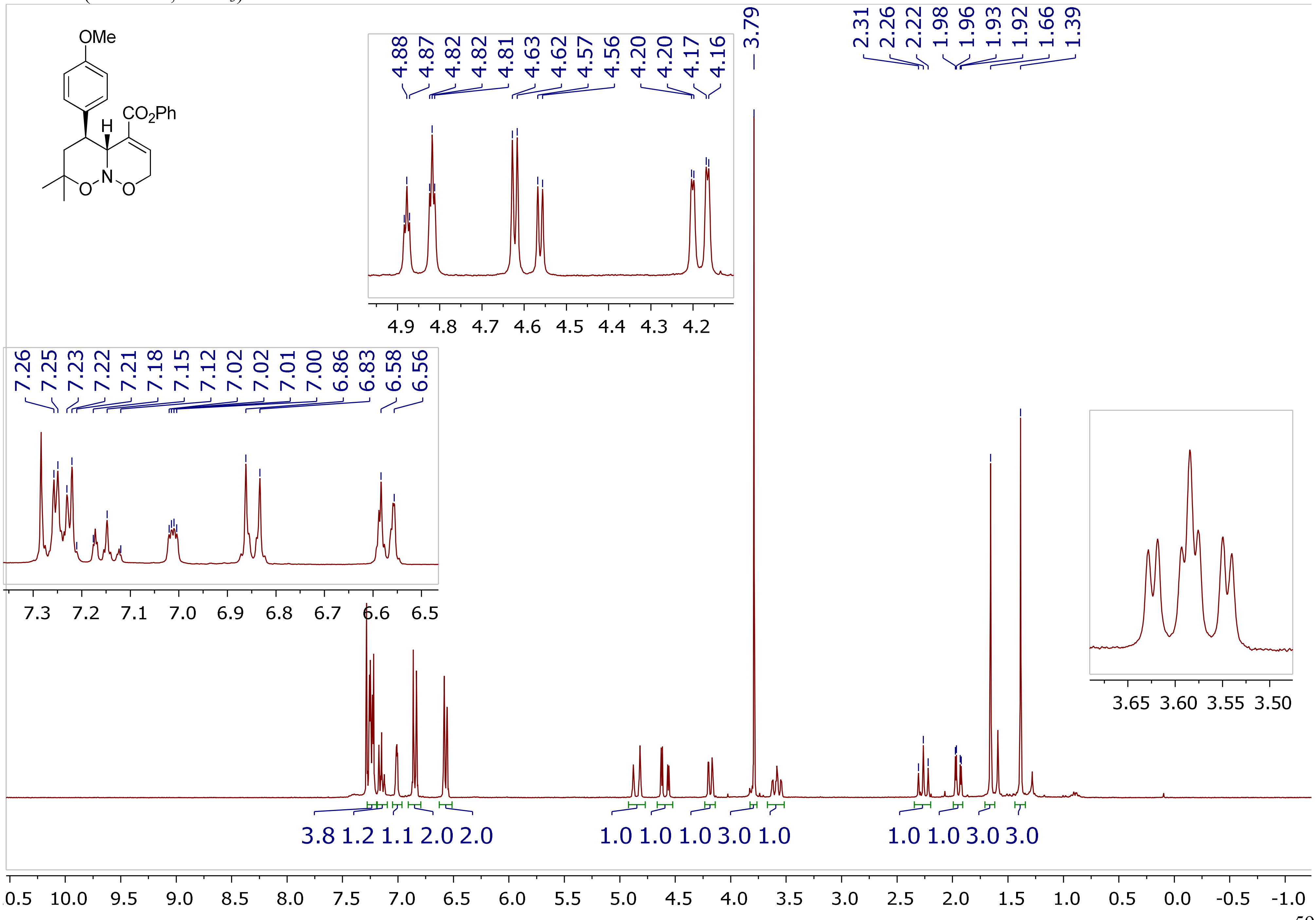

^{13}C NMR (75 MHz, $CDCl_3$)

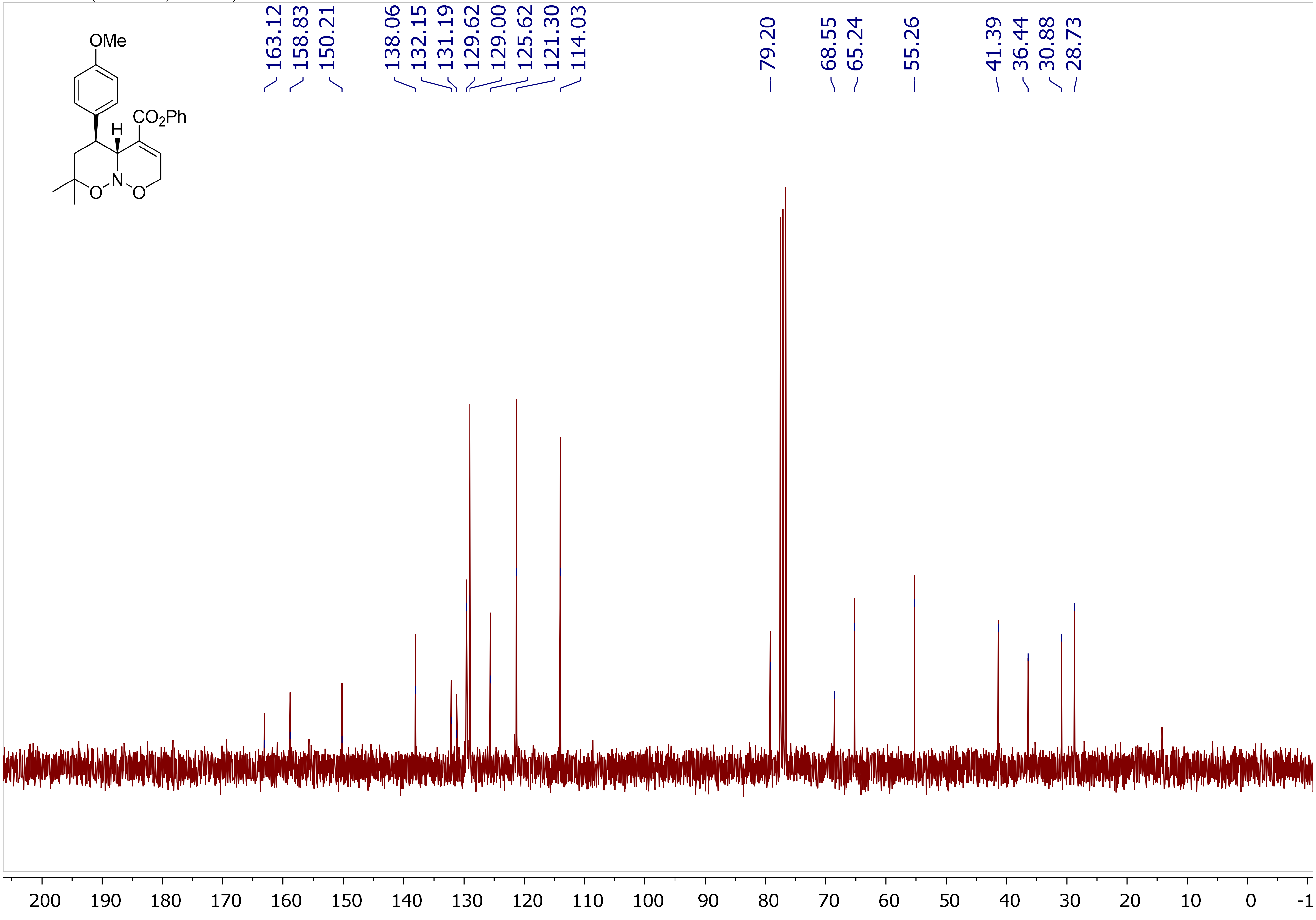

^{13}C DEPT 135 (75 MHz, $CDCl_3$)

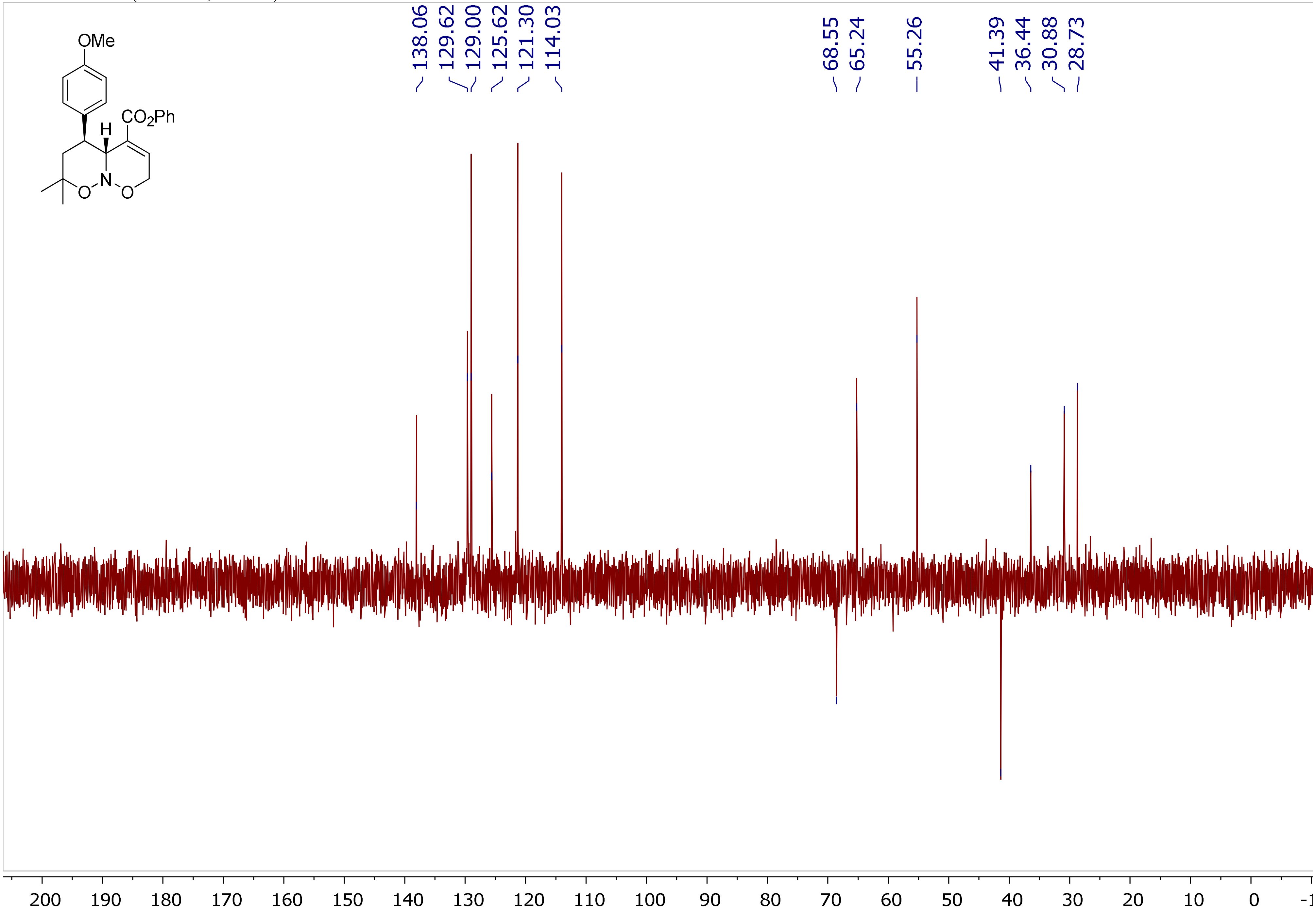

2,2,2-Trichloroethyl (4aR*,5S*)-5-(4-chlorophenyl)-7,7-dimethyl-4a,5,6,7-tetrahydro-2H-[1,2]oxazino[2,3-b][1,2]oxazine-4-carboxylate 3g
^{1}H NMR (300 MHz, $CDCl_3$)

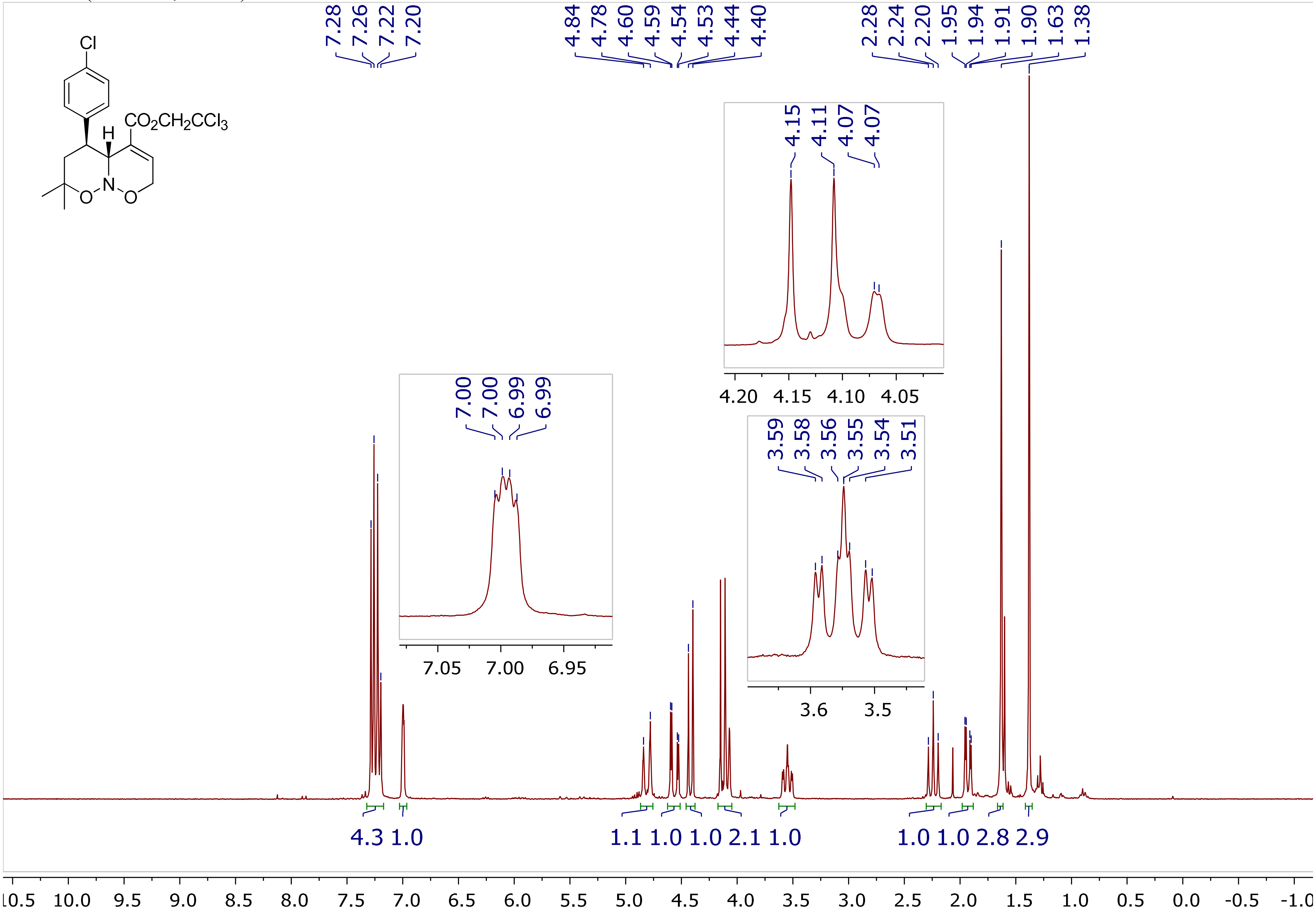

^{13}C NMR (75 MHz, $CDCl_3$)

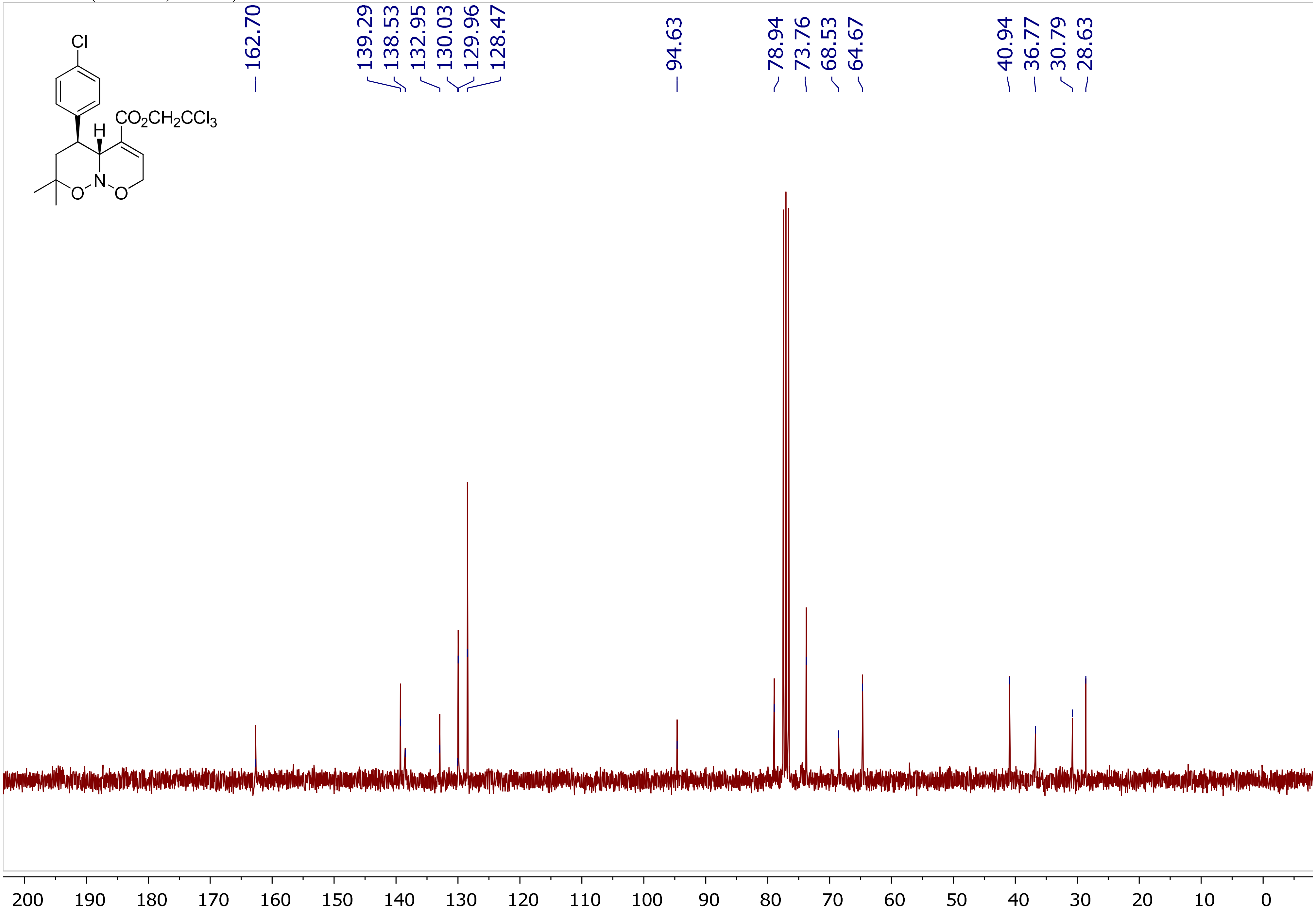

^{13}C DEPT 135 (75 MHz, $CDCl_3$)

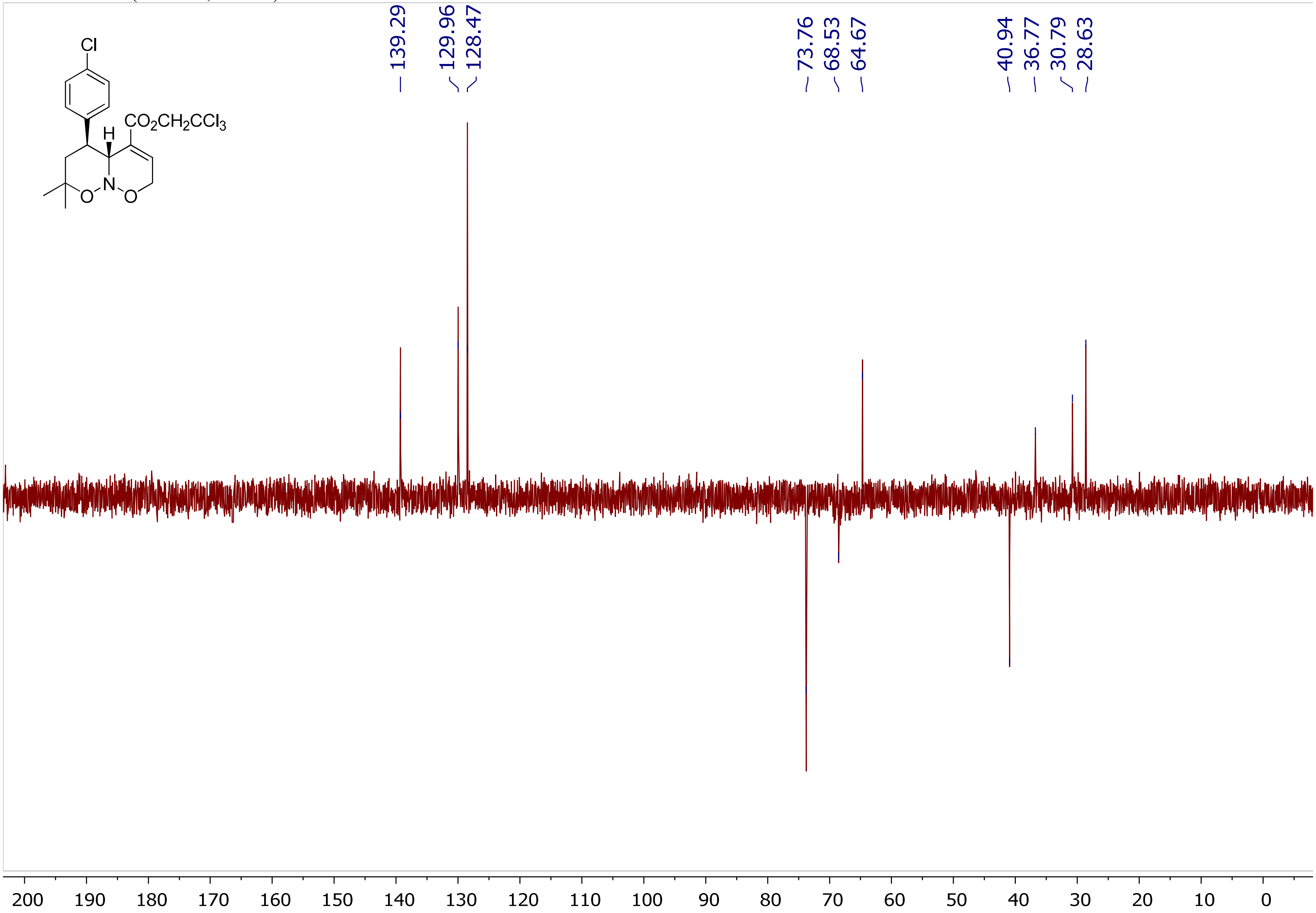

2,2,2-Trichloroethyl (4aR*,5S*)-7,7-dimethyl-5-(4-nitrophenyl)-4a,5,6,7-tetrahydro-2H-[1,2]oxazino[2,3-b][1,2]oxazine-4-carboxylate 3h

^{1}H NMR (300 MHz, $CDCl_3$)

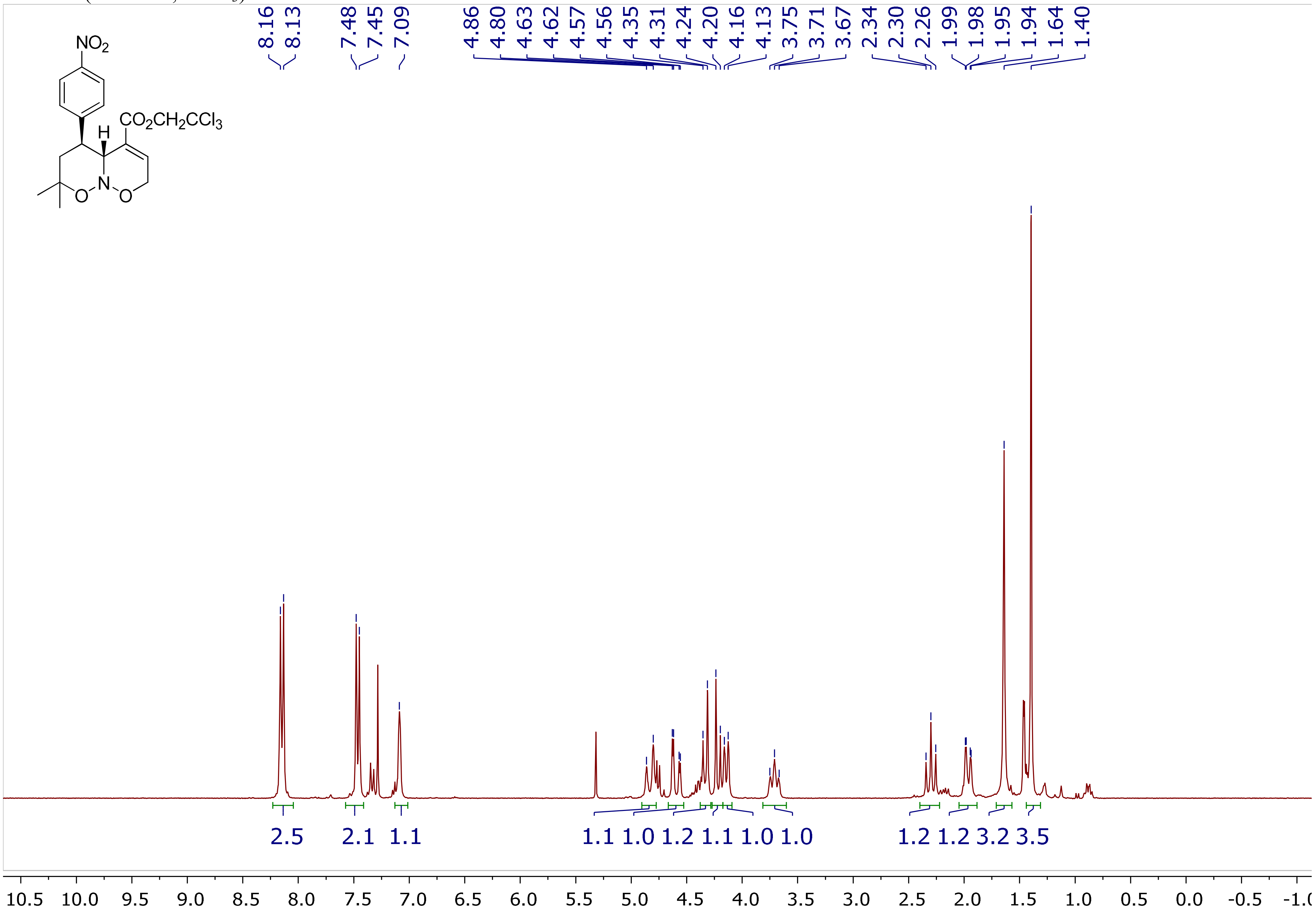

^{13}C NMR (75 MHz, $CDCl_3$)

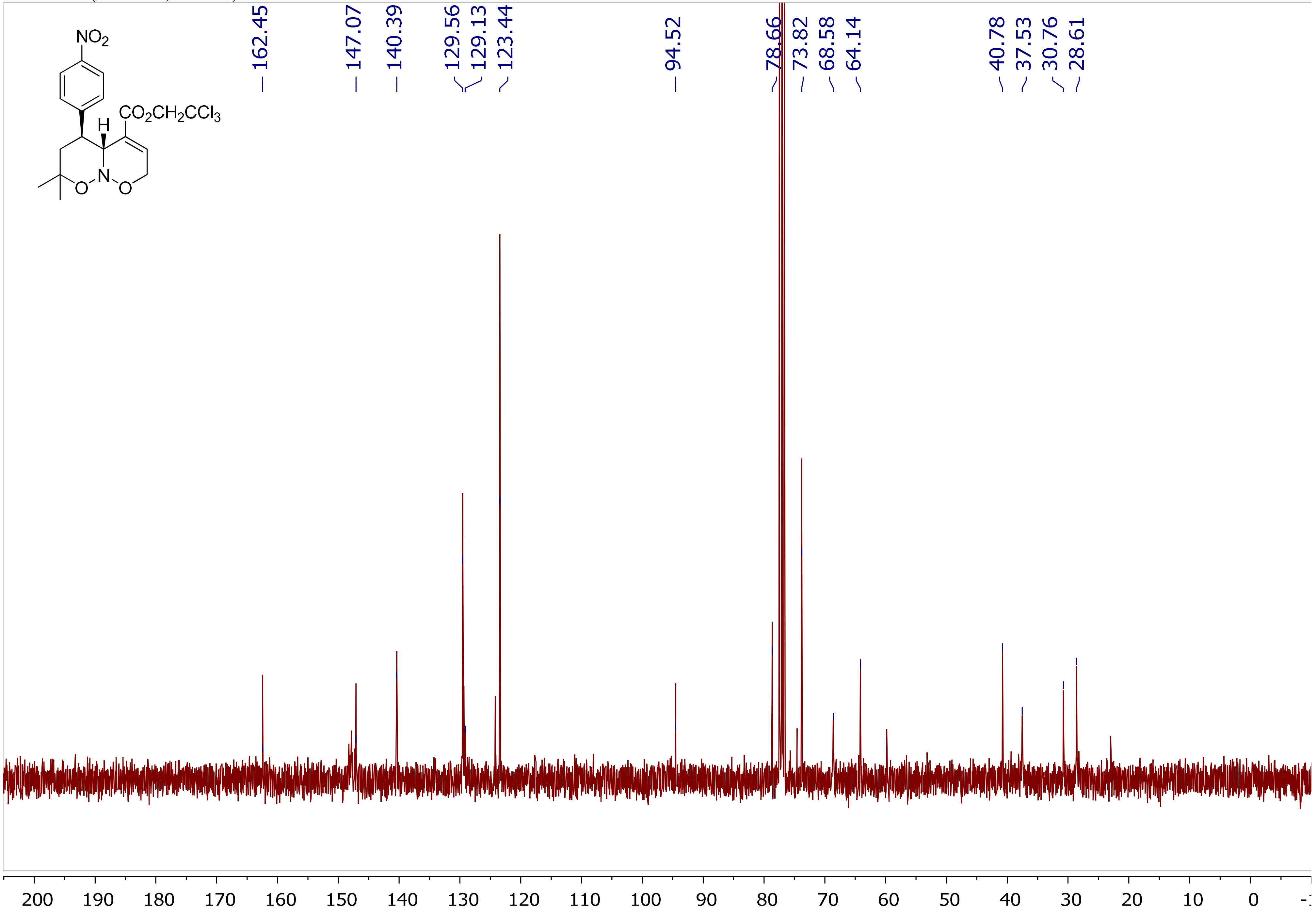

^{13}C DEPT 135 (75 MHz, $CDCl_3$)

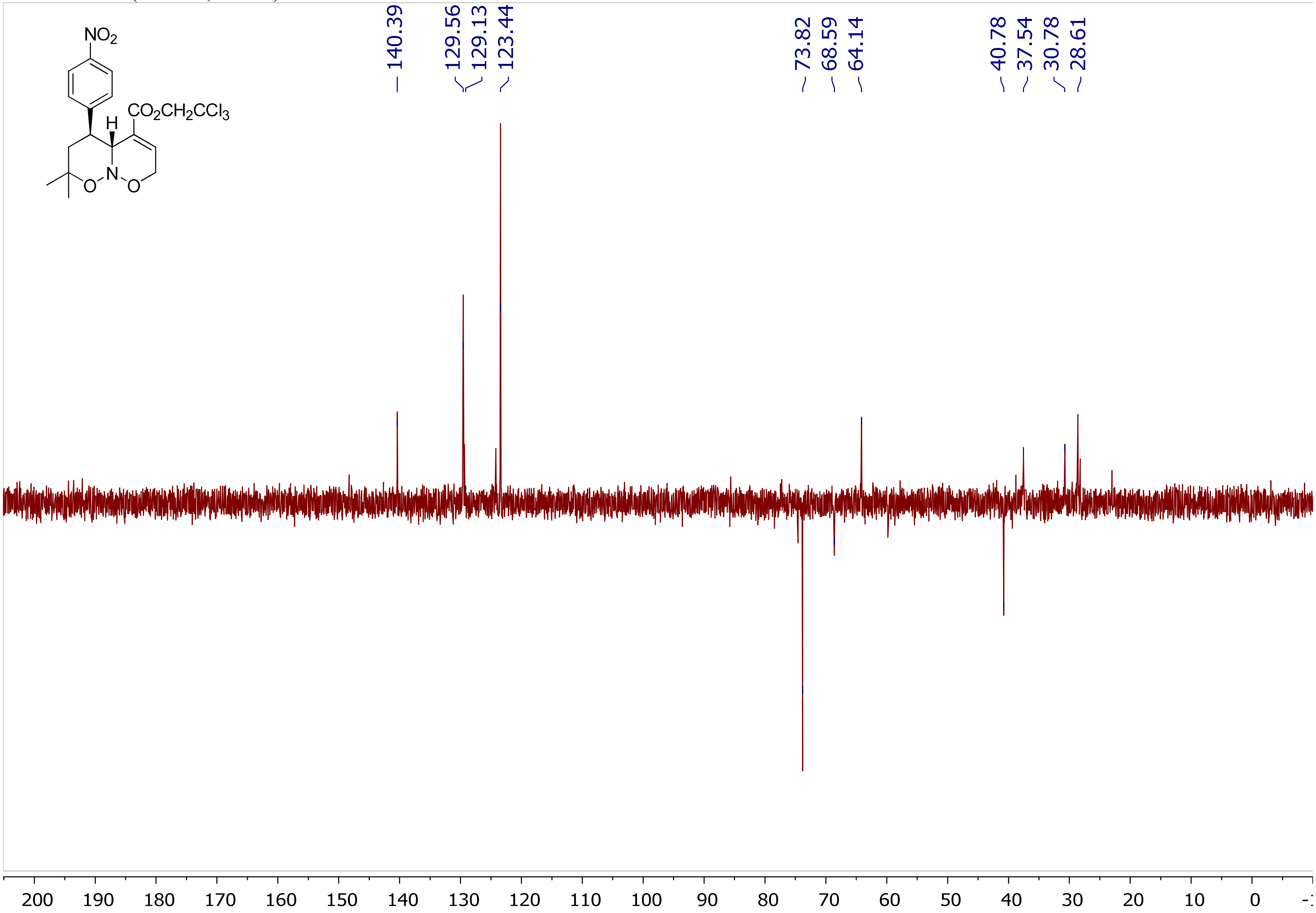

2,2,2-Trichloroethyl (4aR*,5S*)-5-(3-methoxyphenyl)-7,7-dimethyl-4a,5,6,7-tetrahydro-2H-[1,2]oxazino[2,3-b][1,2]oxazine-4-carboxylate 3i

^{1}H NMR (300 MHz, $CDCl_3$)

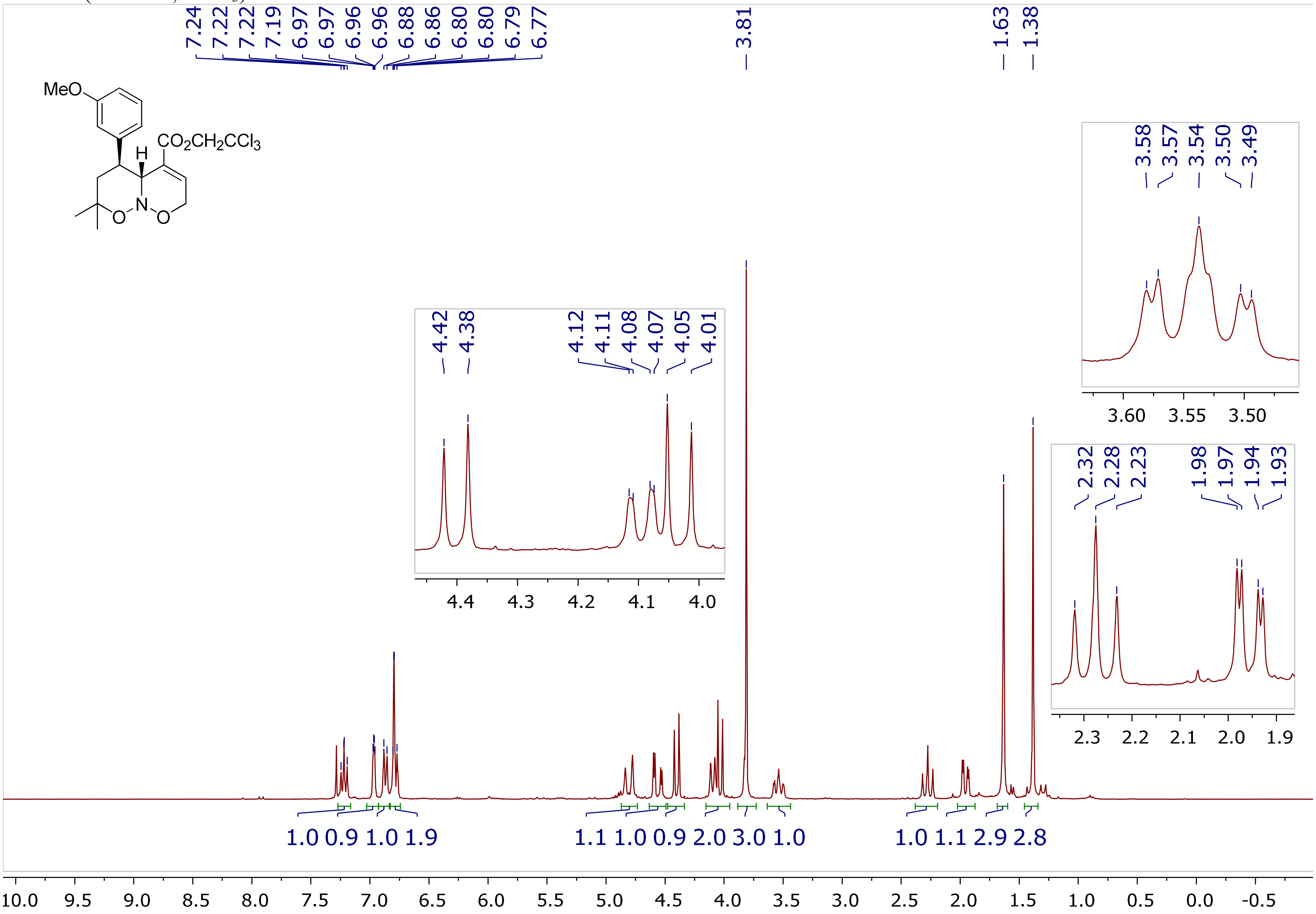

^{13}C NMR (75 MHz, $CDCl_3$)

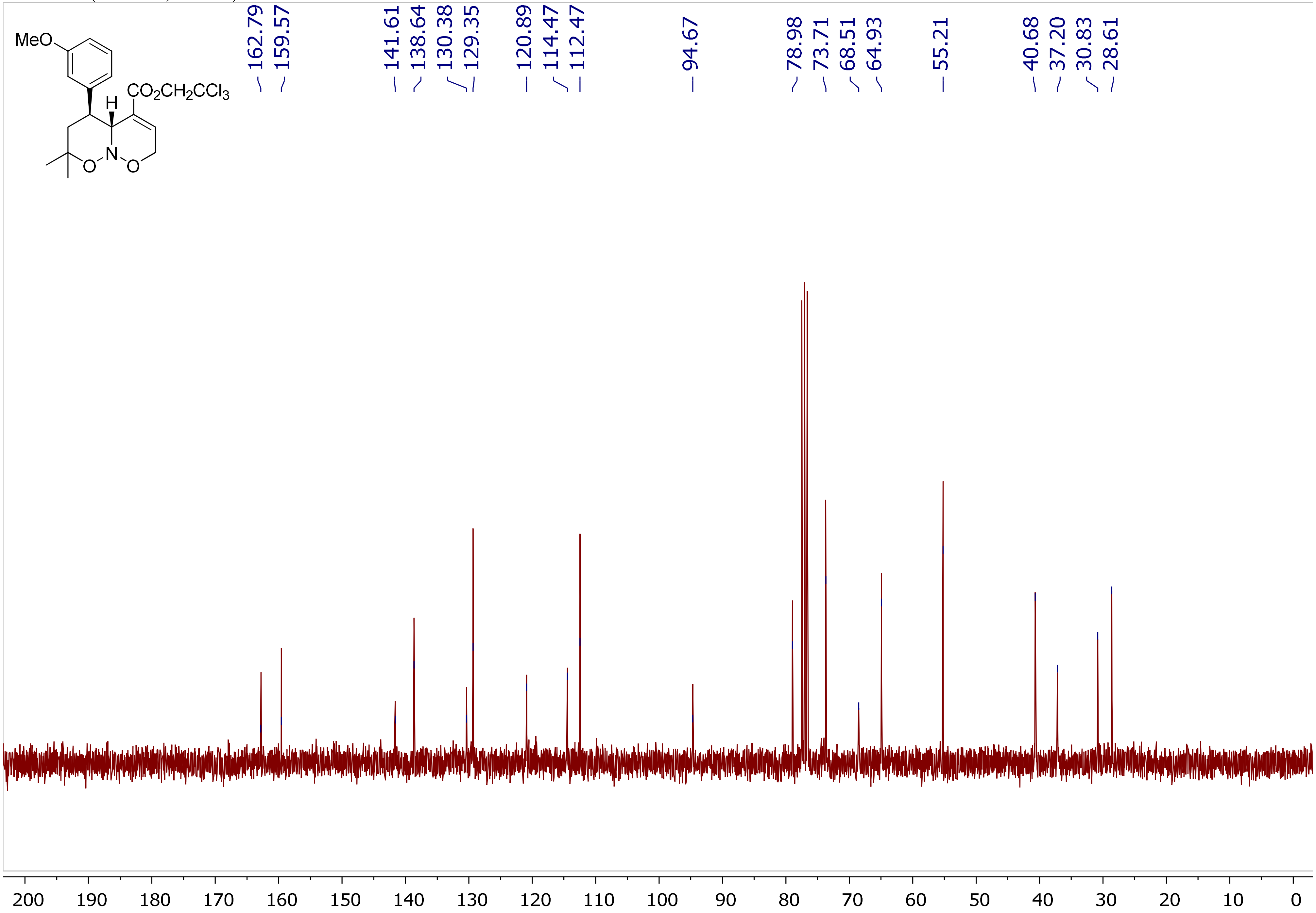

^{13}C DEPT 135 (75 MHz, $CDCl_3$)

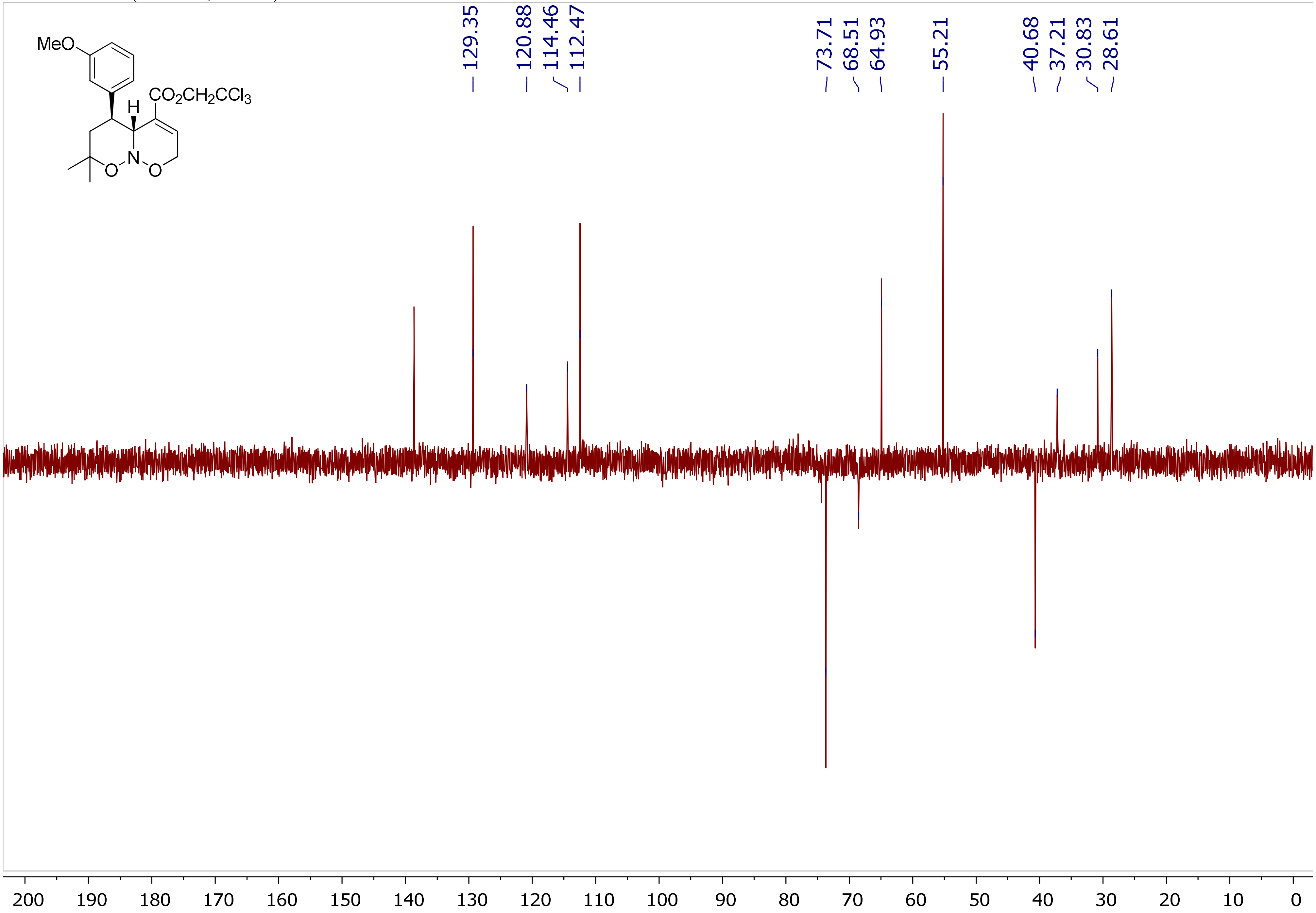

2,2,2-Trichloroethyl (4aR*,5S*)-5-(2-chlorophenyl)-7,7-dimethyl-4a,5,6,7-tetrahydro-2H-[1,2]oxazino[2,3-b][1,2]oxazine-4-carboxylate 3j

^{1}H NMR (300 MHz, $CDCl_3$)

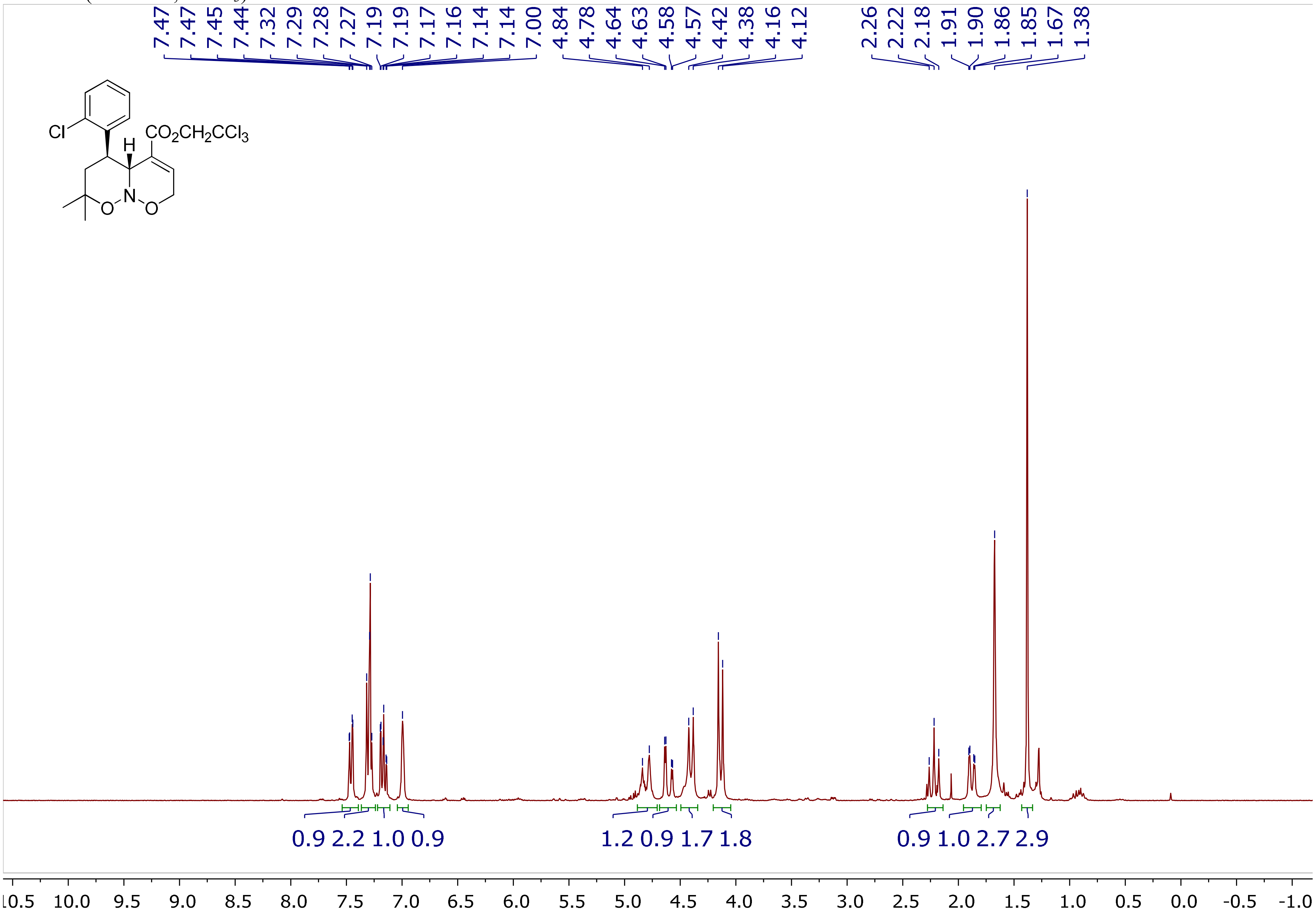

^{13}C NMR (75 MHz, $CDCl_3$)

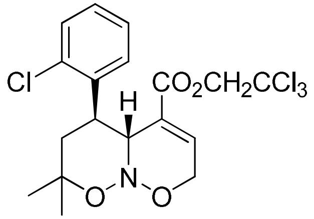

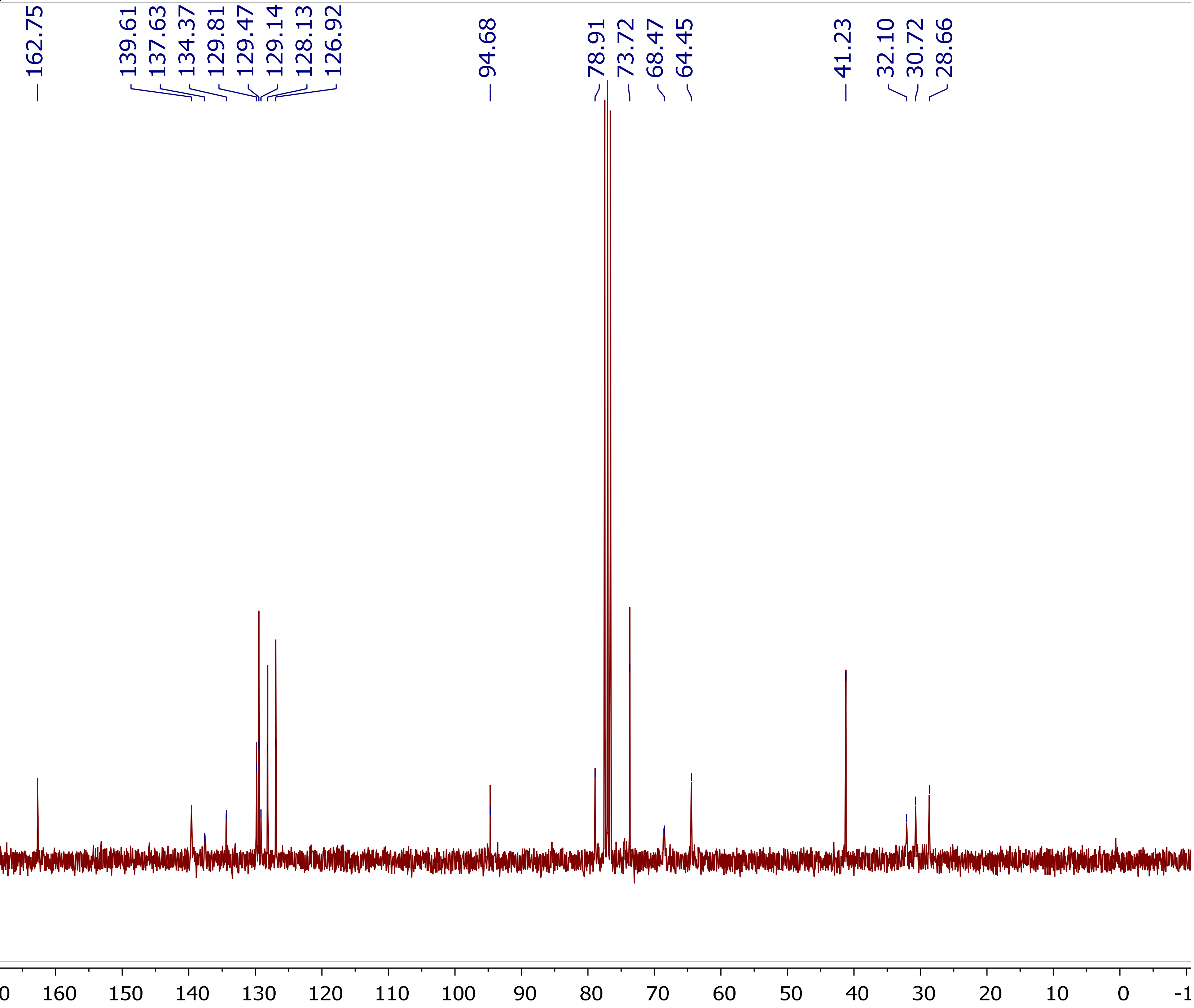

^{13}C DEPT 135 (75 MHz, $CDCl_3$)

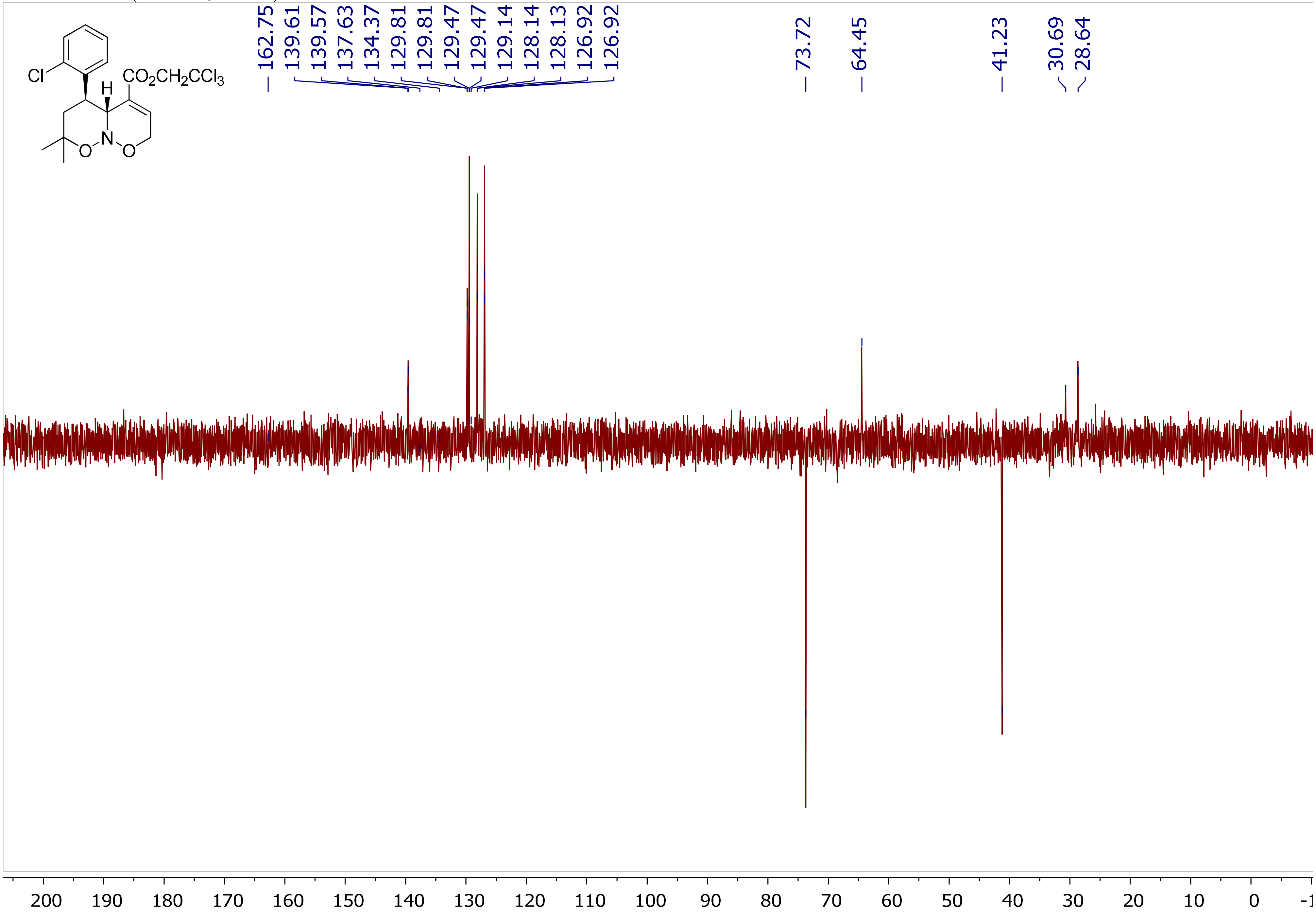

2,2,2-Trichloroethyl (4aR*,5S*)-5-(3-(cyclopentyloxy)-4-methoxyphenyl)-7,7-dimethyl-4a,5,6,7-tetrahydro-2H-[1,2]oxazino[2,3-b][1,2]oxazine-4-carboxylate 3k

^{1}H NMR (300 MHz, $CDCl_3$)

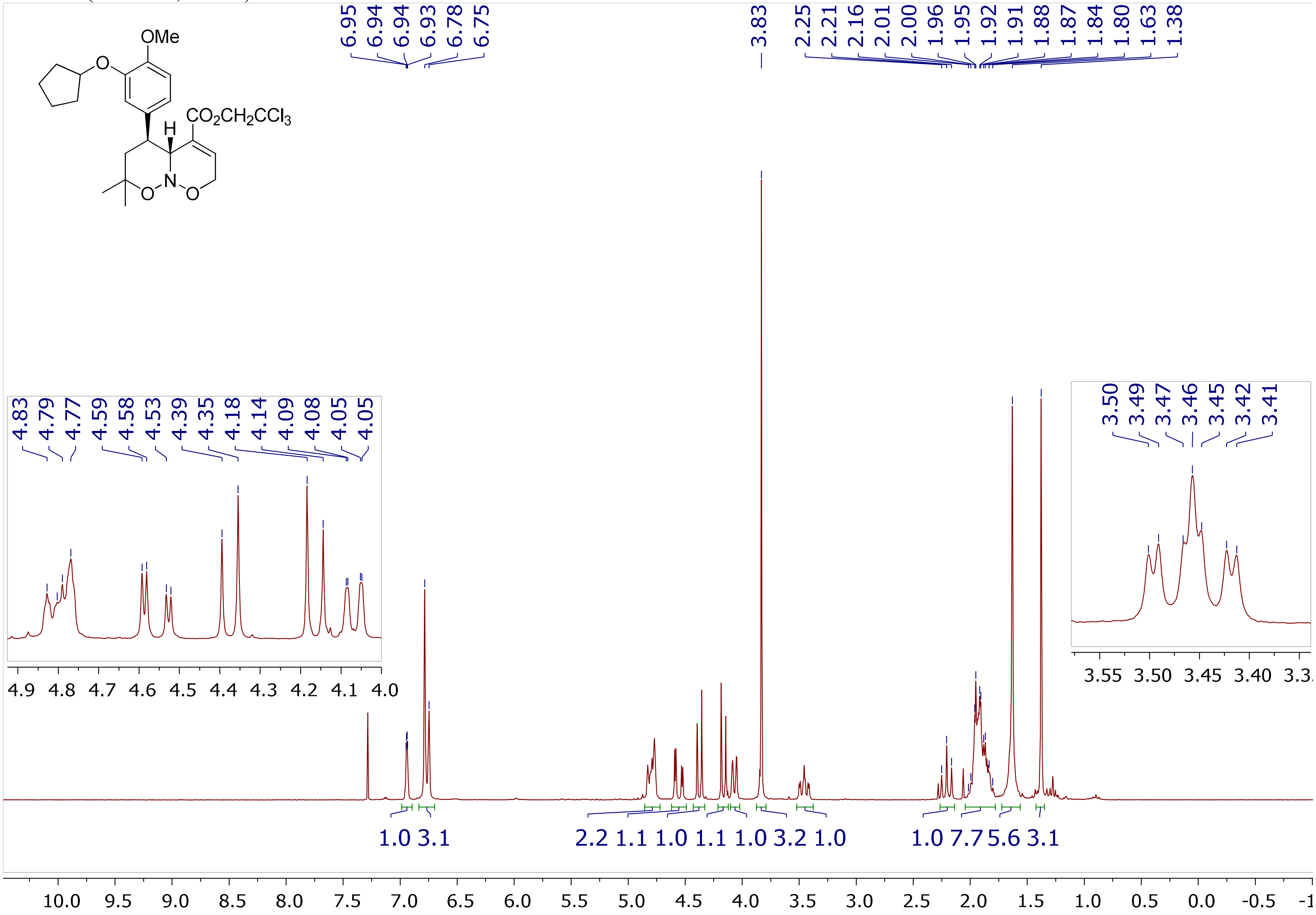

^{13}C NMR (75 MHz, $CDCl_3$)

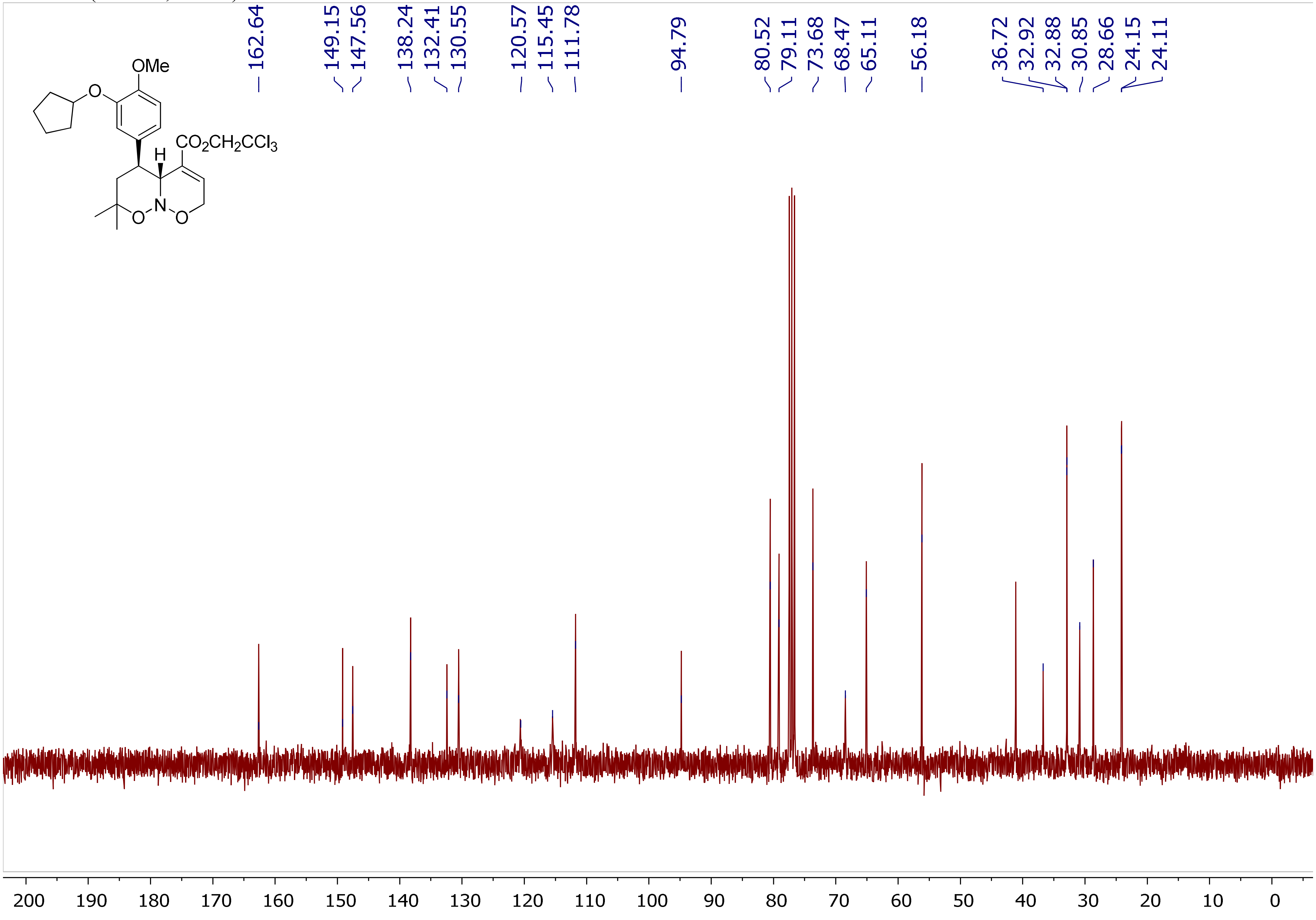

^{13}C DEPT 135 (75 MHz, $CDCl_3$)

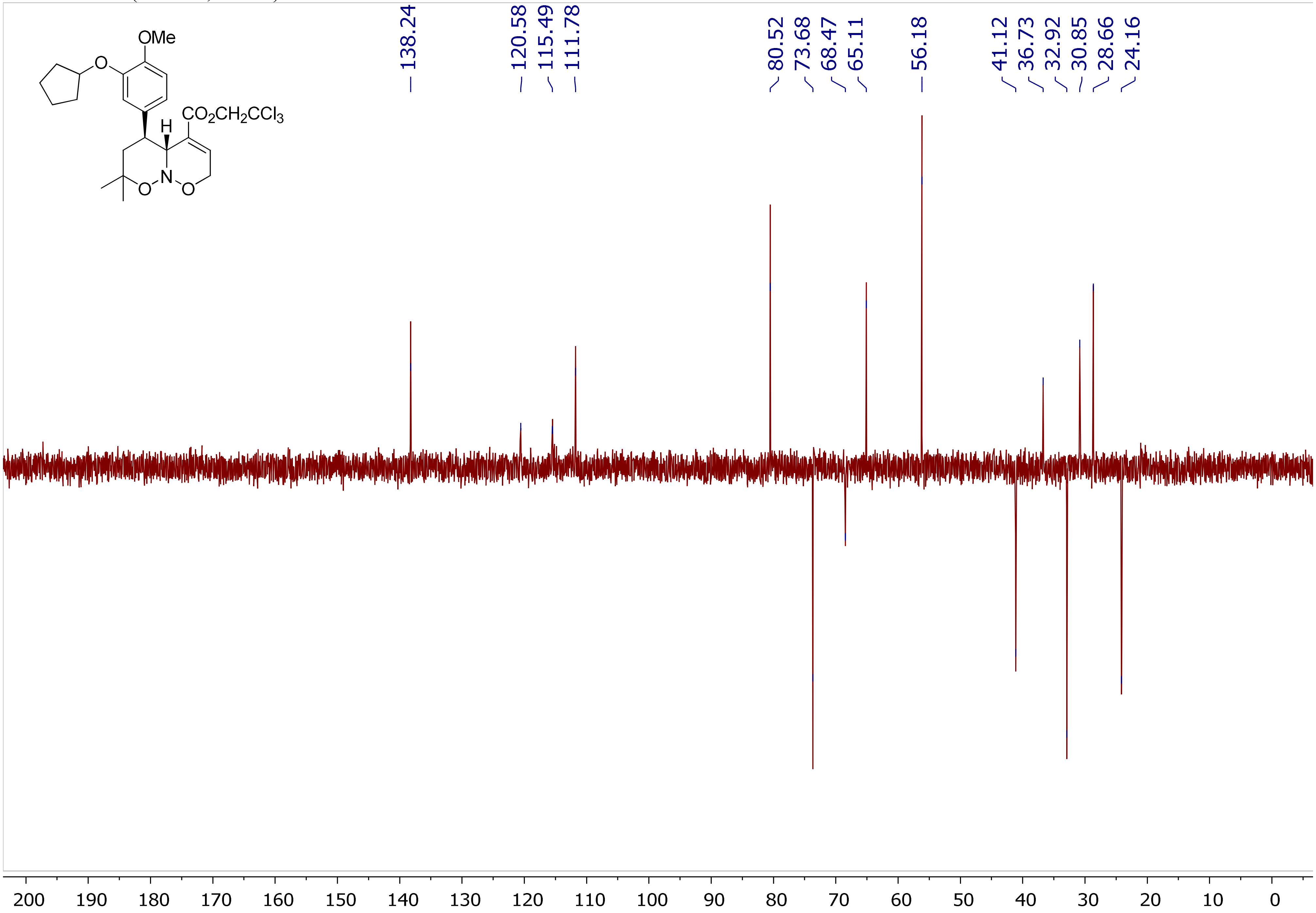

2,2,2-Trichloroethyl (4aS*,5R*)-5-(benzoyloxy)-7,7-dimethyl-4a,5,6,7-tetrahydro-2H-[1,2]oxazino[2,3-b][1,2]oxazine-4-carboxylate 3l

^{1}H NMR (300 MHz, $CDCl_3$, 320K)

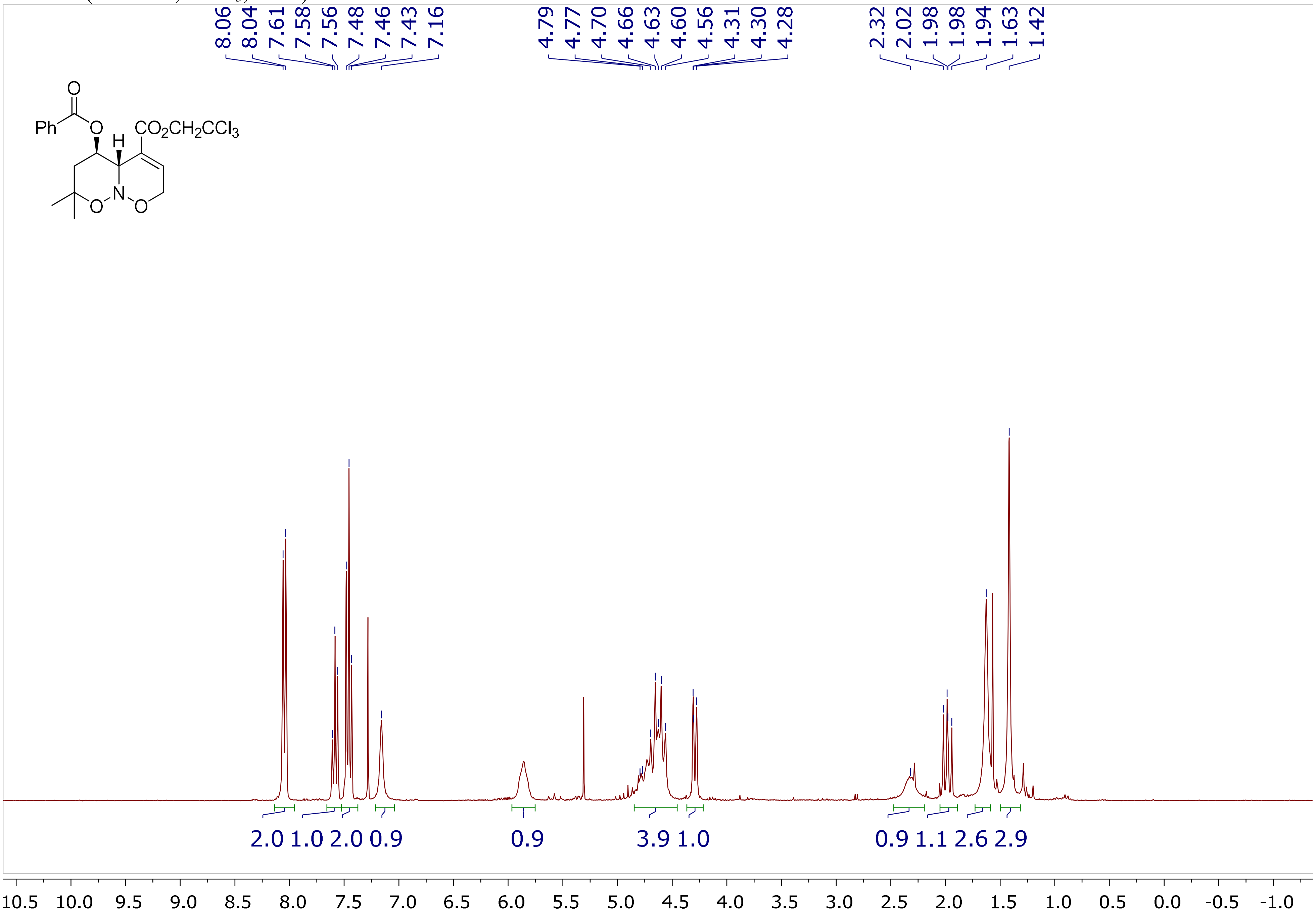

^{13}C NMR (75 MHz, $CDCl_3$, 320K)

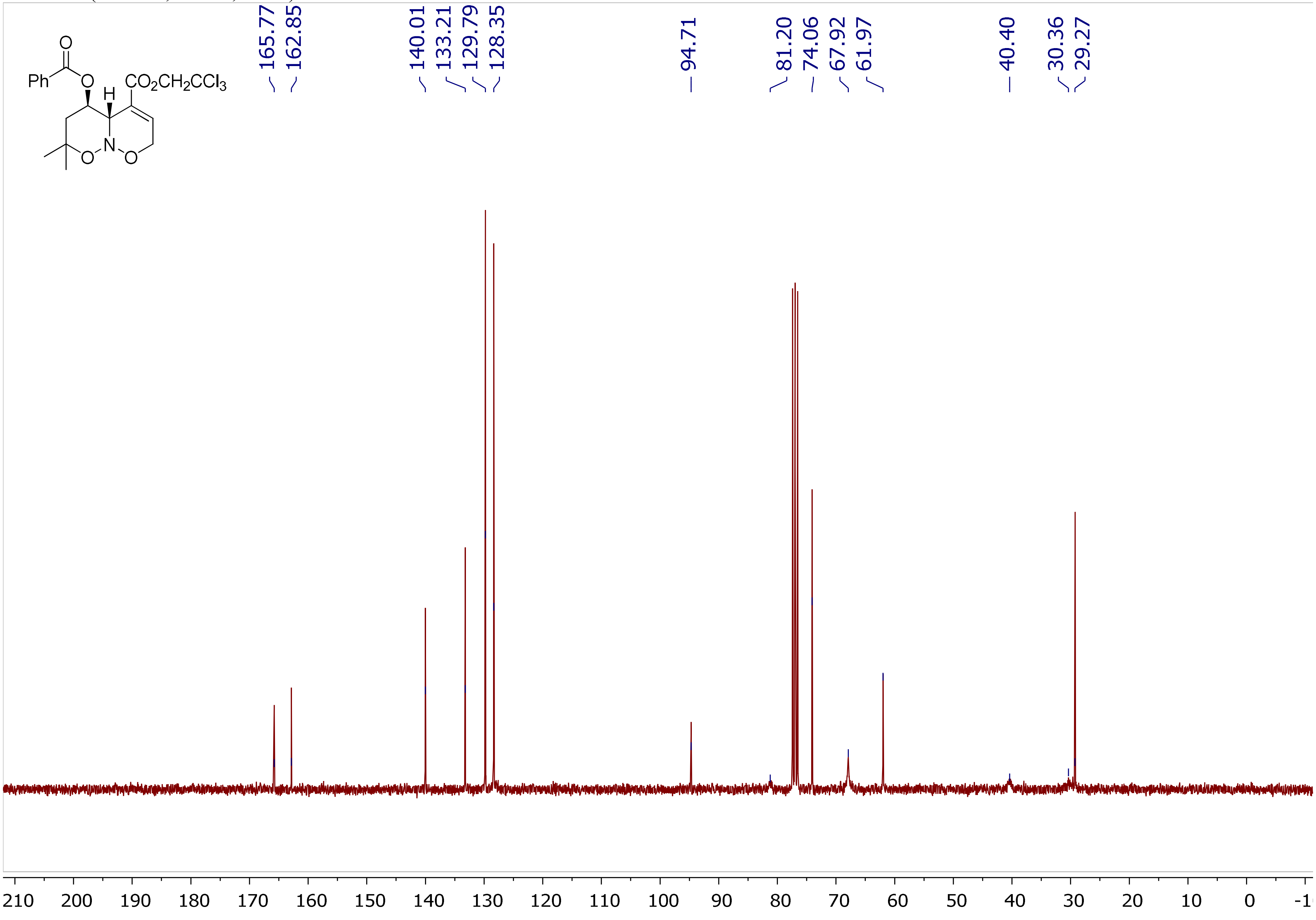

^{13}C DEPT 135 (75 MHz, $CDCl_3$, 320K)

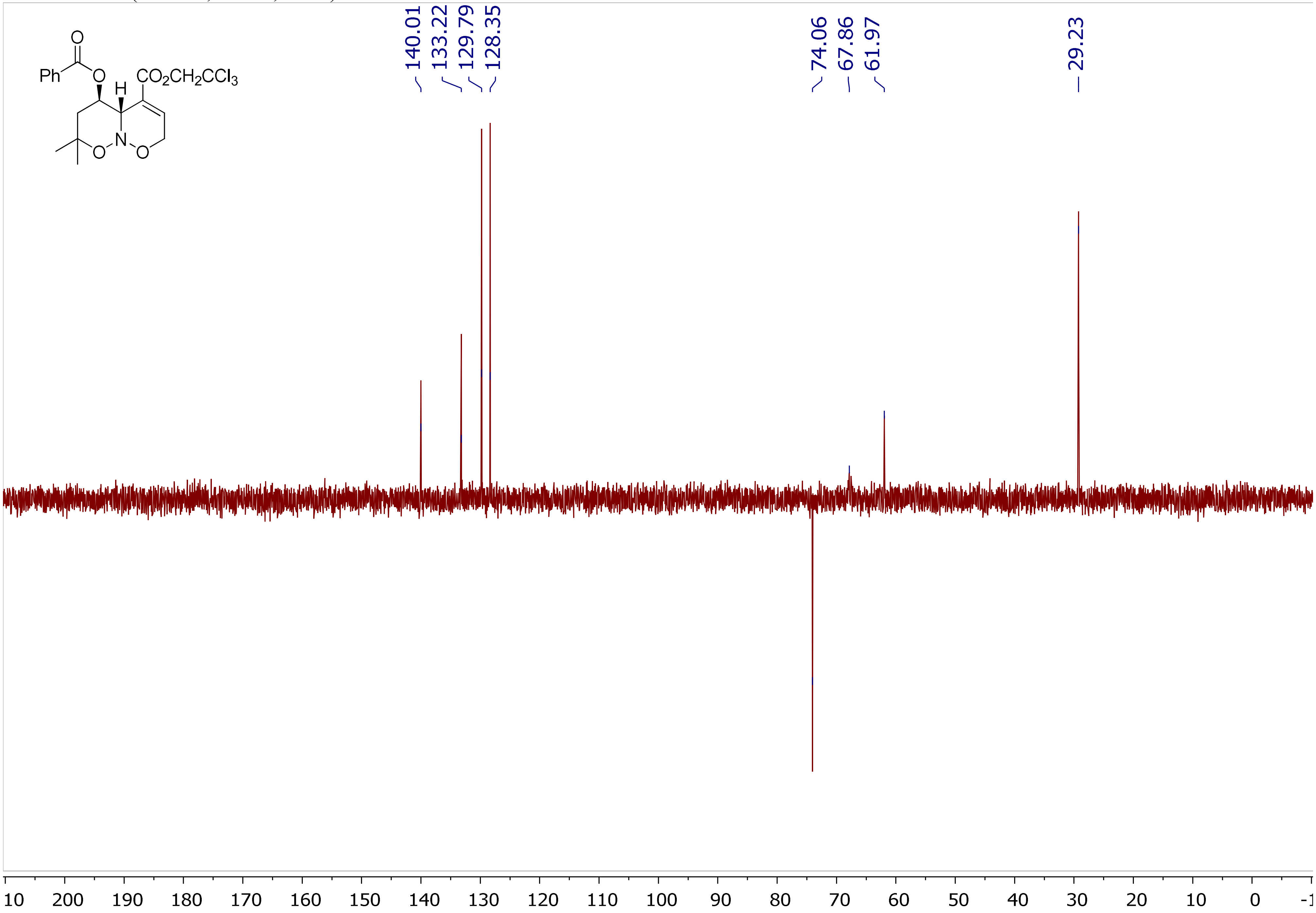

^{1}H-^{1}H COSY

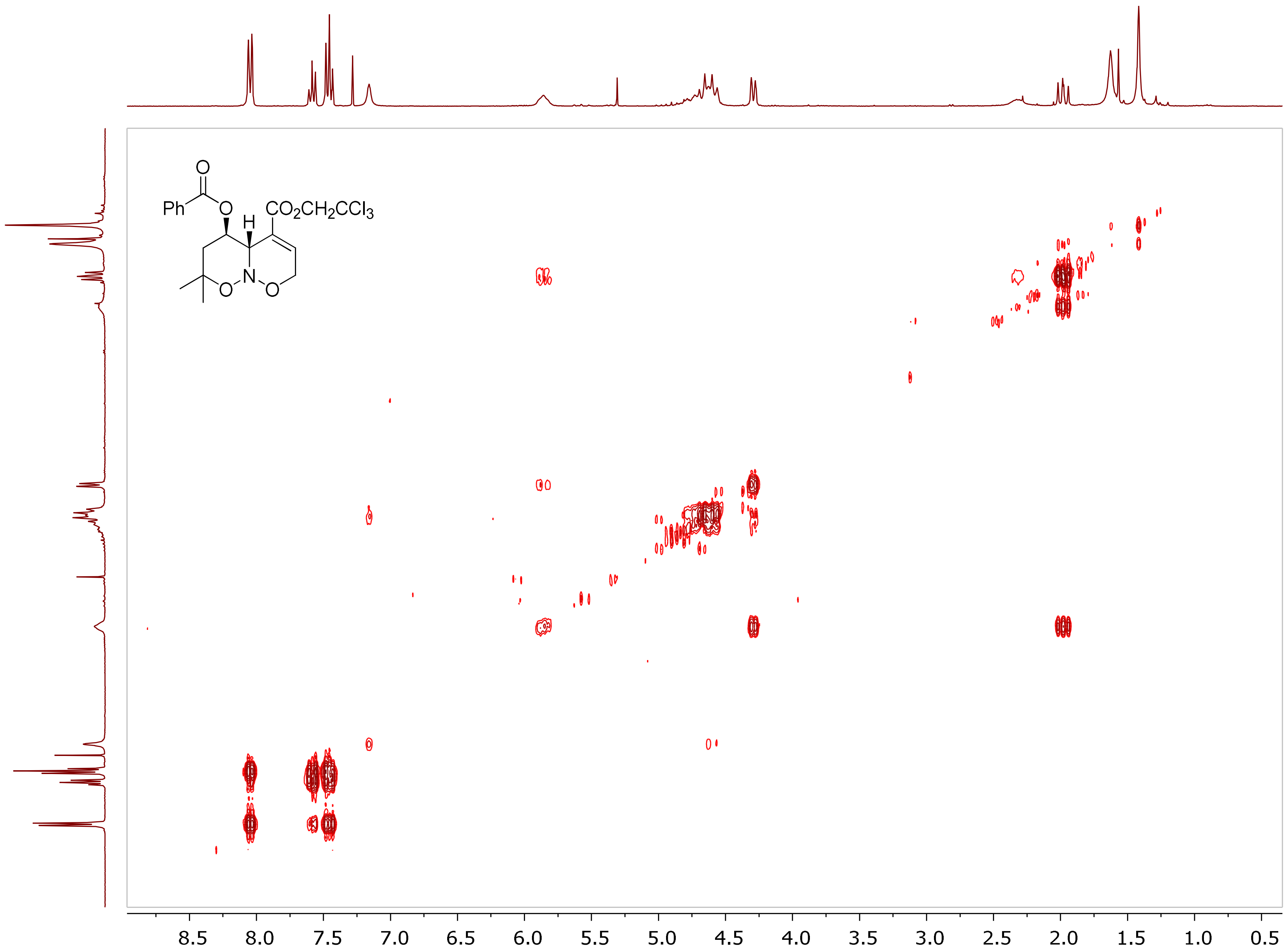
Ph
O
O
H
CO2CH2CCl3
N
O
O
8.5
8.0
7.5
7.0
6.5
6.0
5.5
5.0
4.5
4.0
3.5
3.0
2.5
2.0
1.5
1.0
0.5

^{1}H-^{13}C HSQC

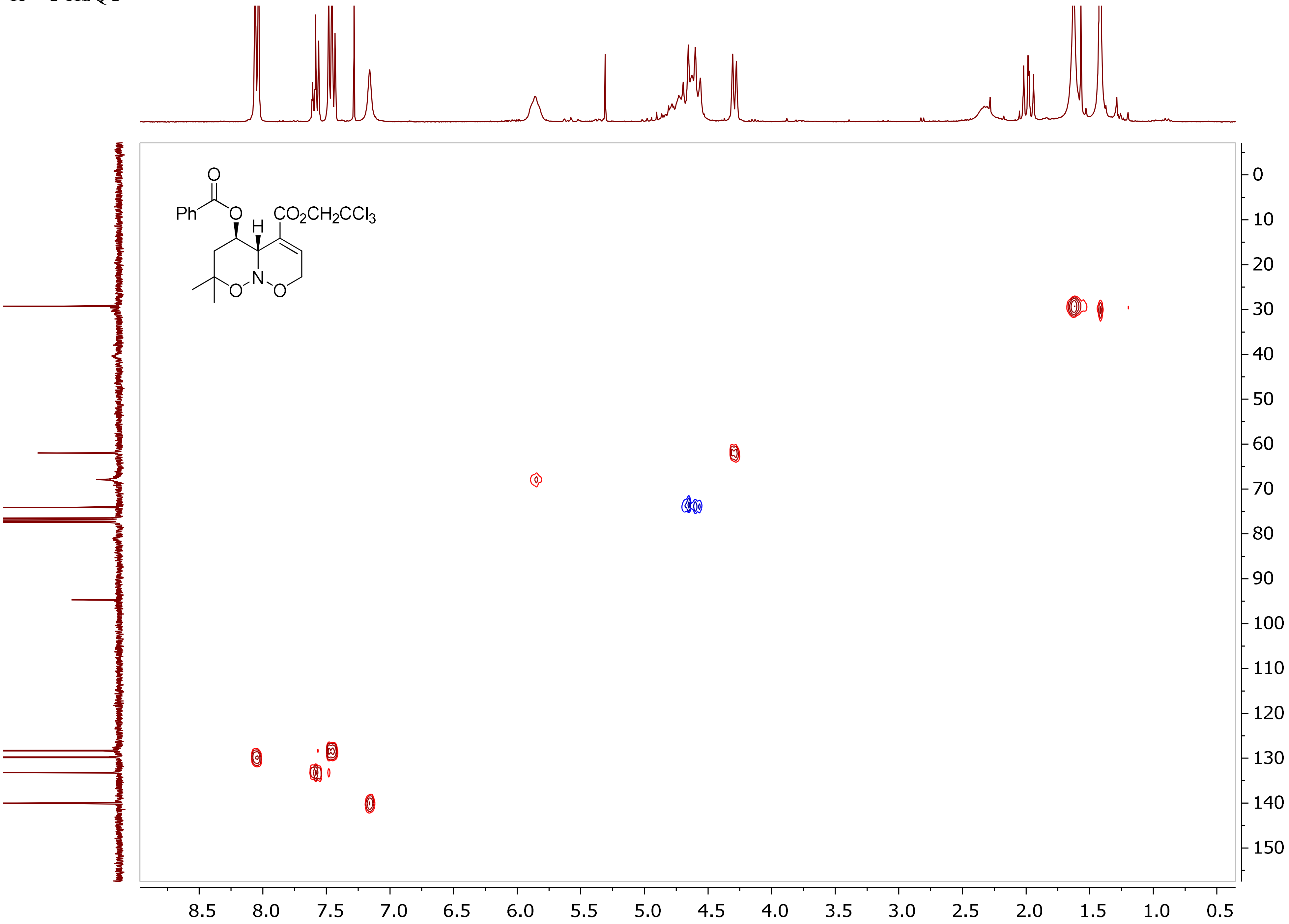

Ph
O
O
H
CO2CH2CCl3
N
O
O
0
10
20
30
40
50
60
70
80
90
100
110
120
130
140
150
8.5
8.0
7.5
7.0
6.5
6.0
5.5
5.0
4.5
4.0
3.5
3.0
2.5
2.0
1.5
1.0
0.5

^{1}H-^{13}C HMBC

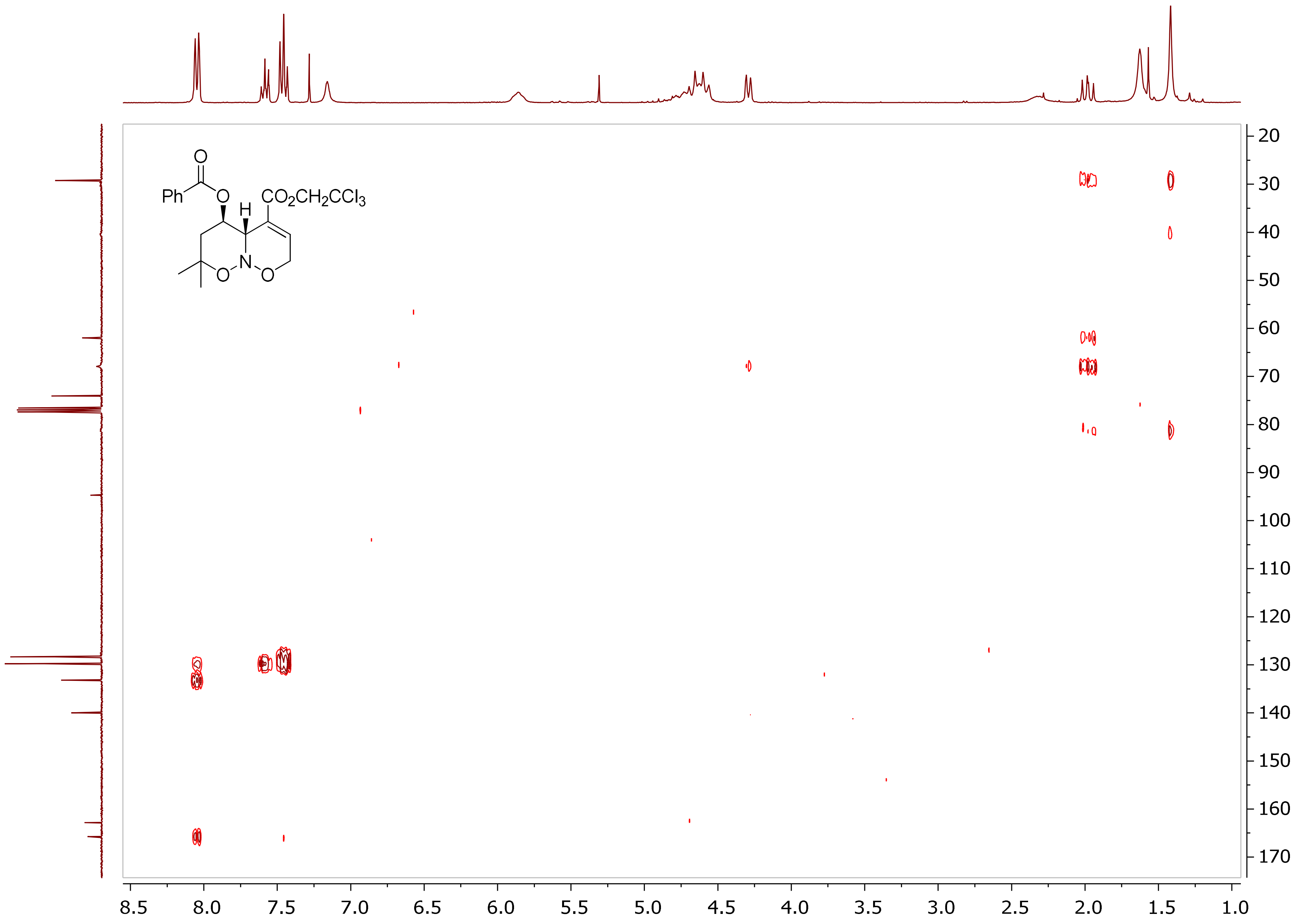
Ph
O
O
H
$CO_2CH_2CCl_3$
N
O
O
20
30
40
50
60
70
80
90
100
110
120
130
140
150
160
170
8.5
8.0
7.5
7.0
6.5
6.0
5.5
5.0
4.5
4.0
3.5
3.0
2.5
2.0
1.5
1.0

^{1}H NMR (300 MHz, DMSO-d$_6$, 320K)

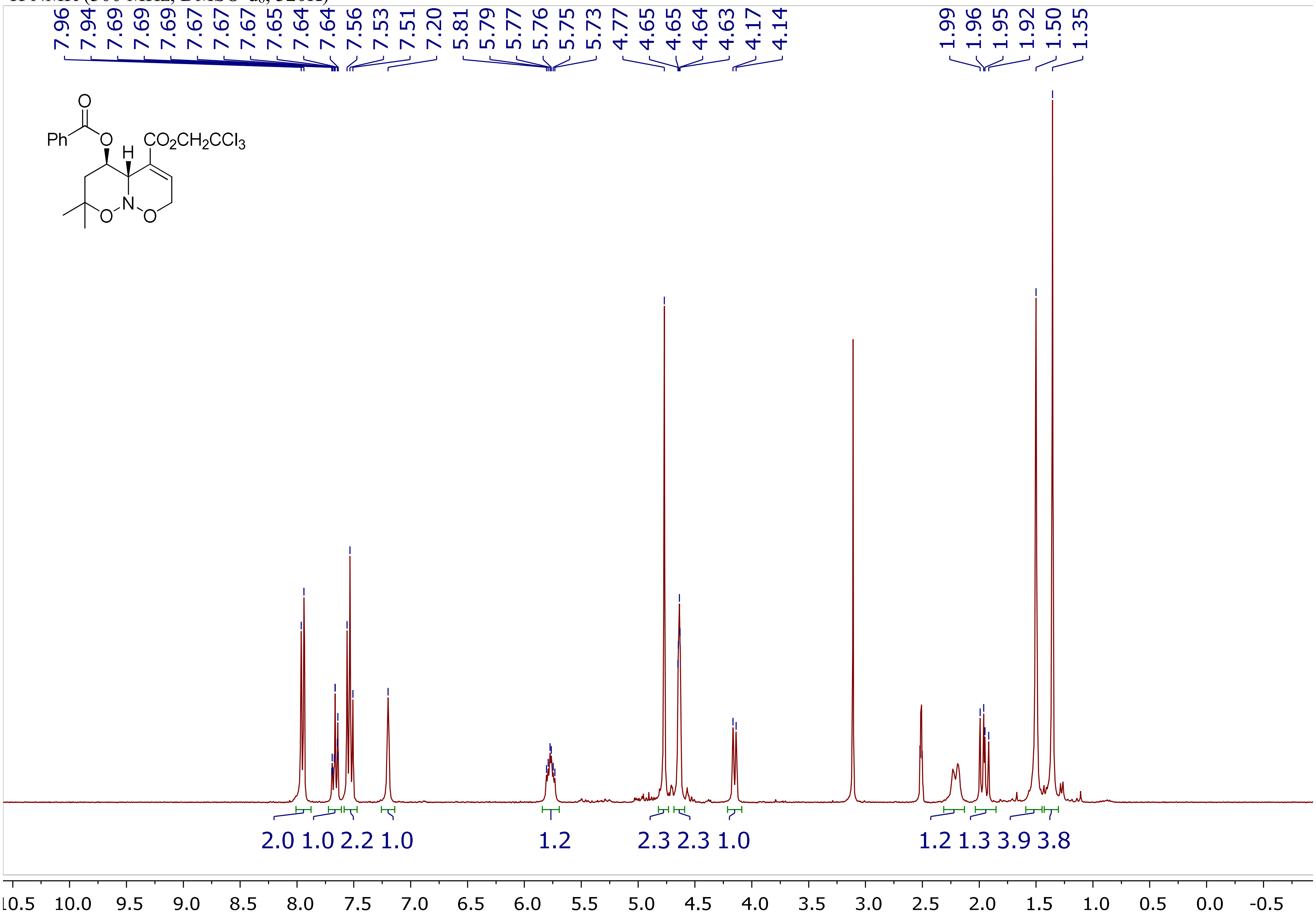

^{13}C NMR (75 MHz, DMSO-d$_6$, 320K)

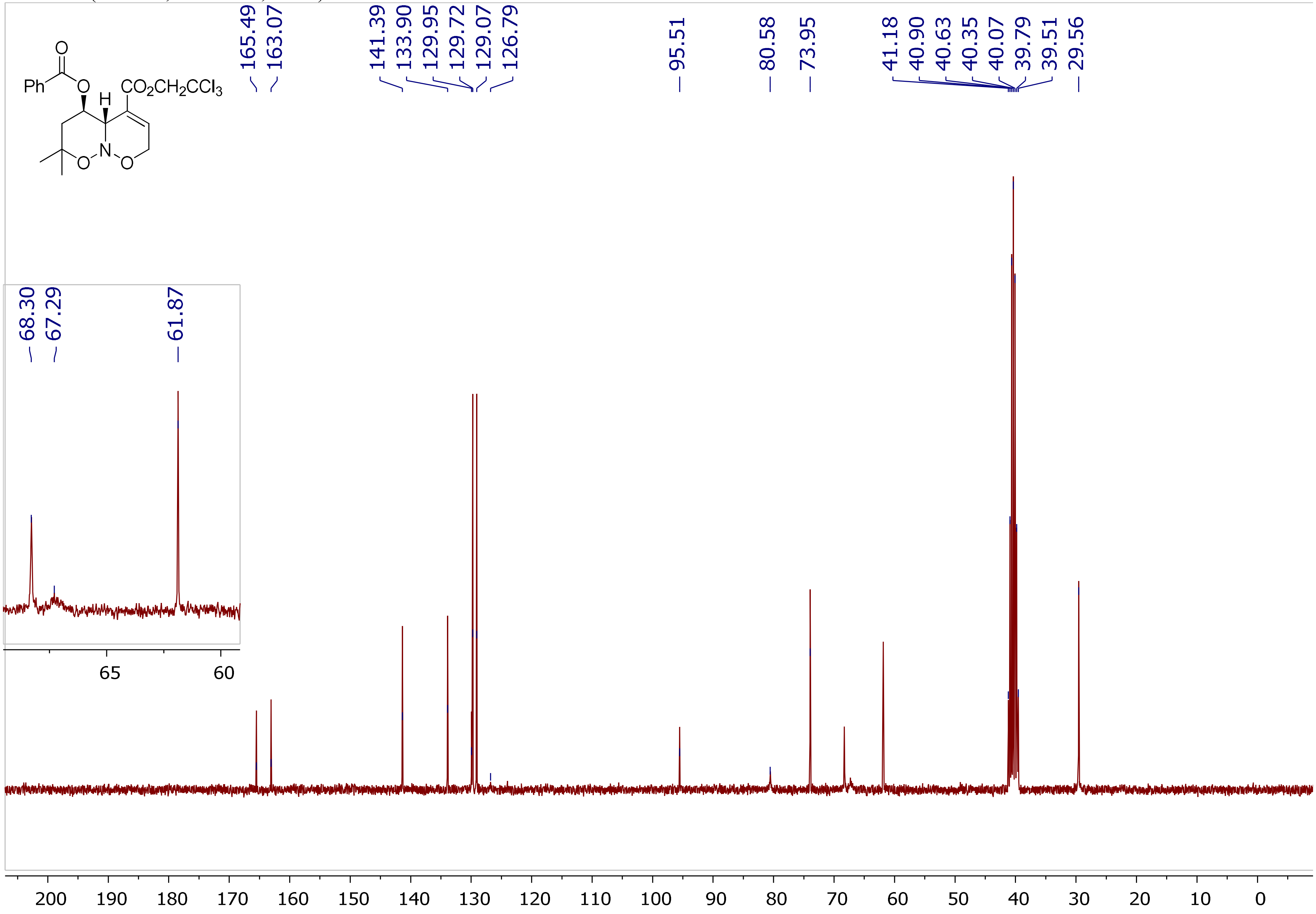

^{13}C DEPT 135 (75 MHz, DMSO-d$_6$, 320K)

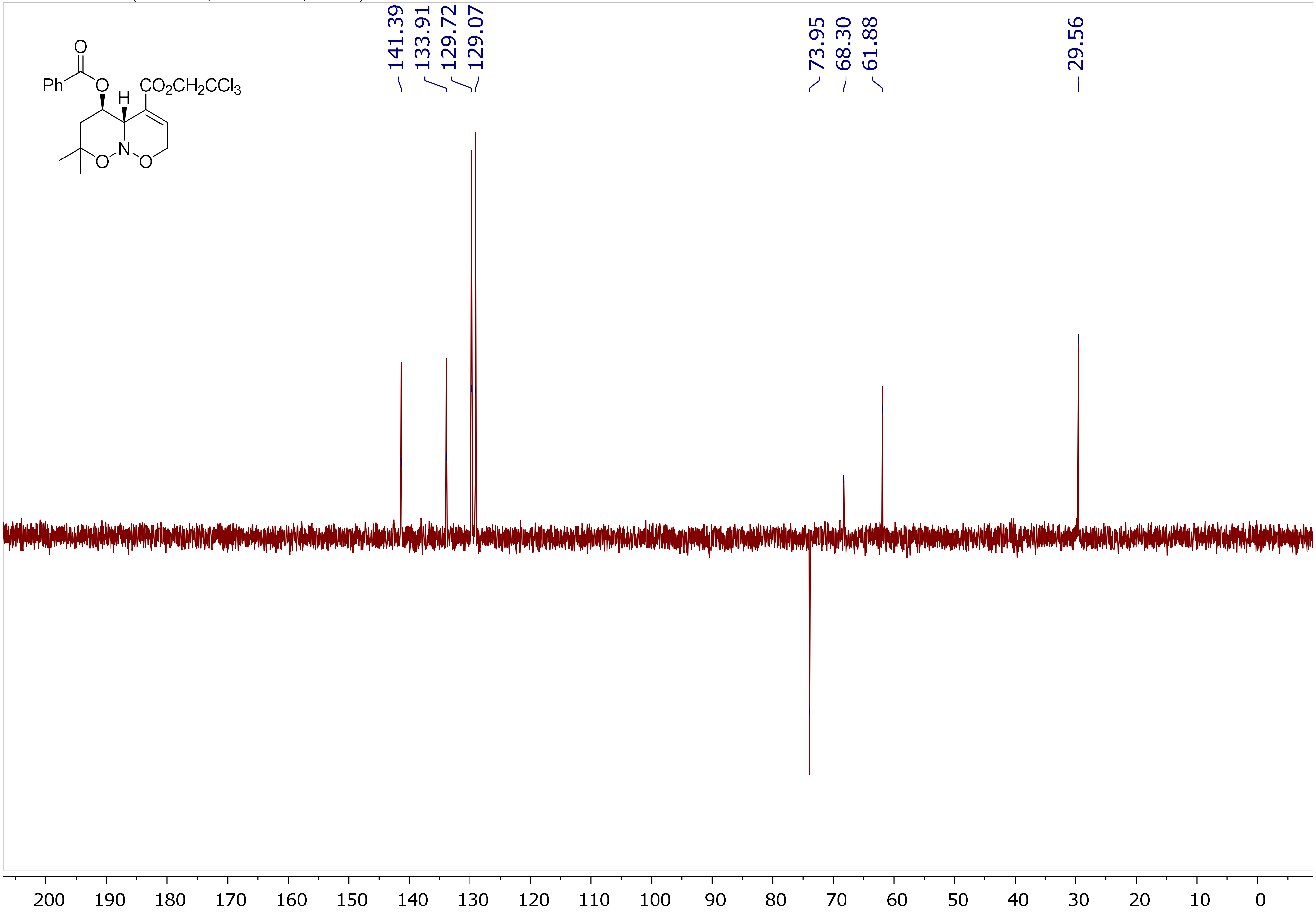

^1H-^1H COSY

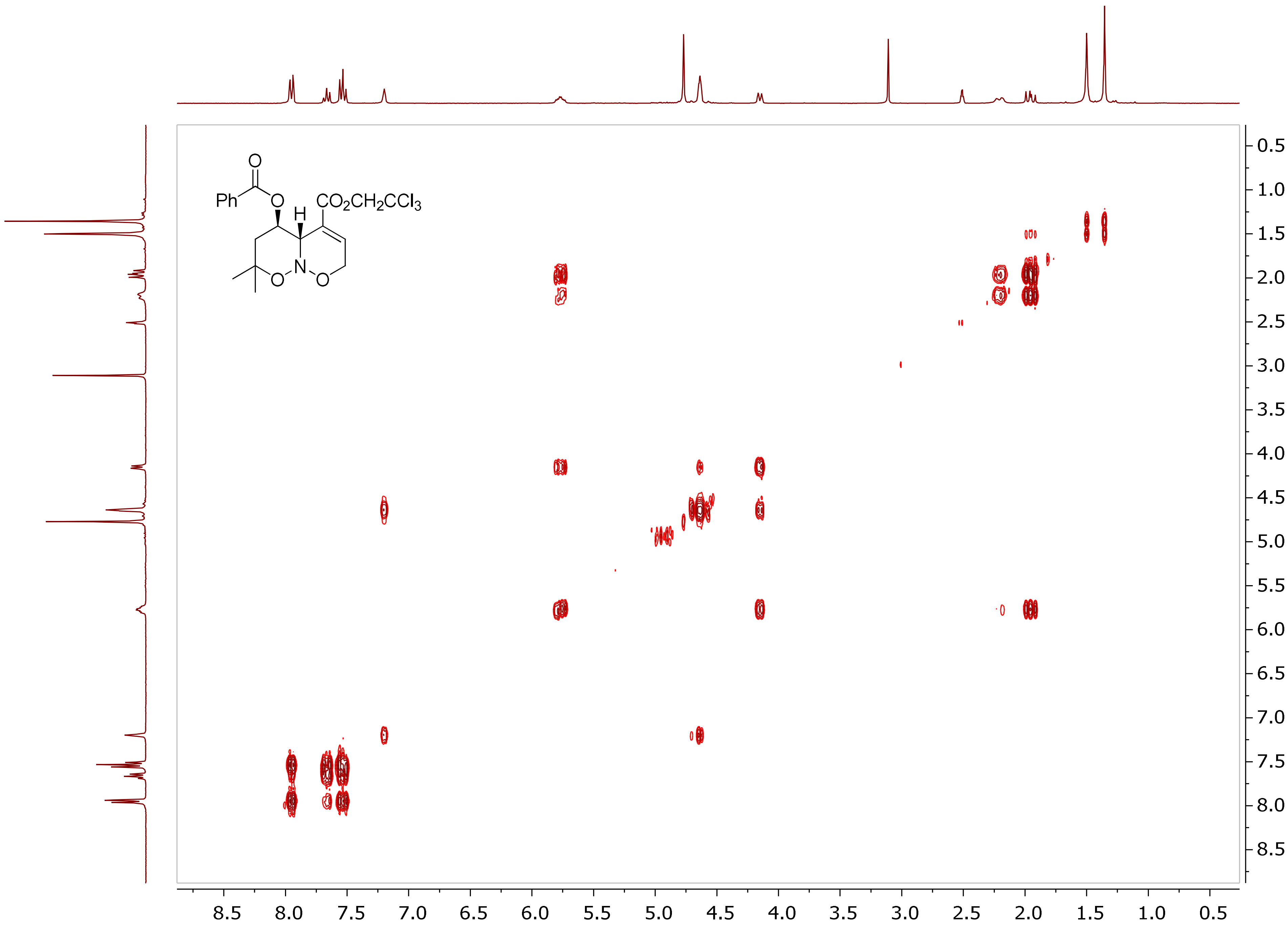

Ph
O
O
H
CO2CH2CCl3
N
O
O
8.5 8.0 7.5 7.0 6.5 6.0 5.5 5.0 4.5 4.0 3.5 3.0 2.5 2.0 1.5 1.0 0.5
0.5 1.0 1.5 2.0 2.5 3.0 3.5 4.0 4.5 5.0 5.5 6.0 6.5 7.0 7.5 8.0 8.5

^{1}H-^{13}C HSQC

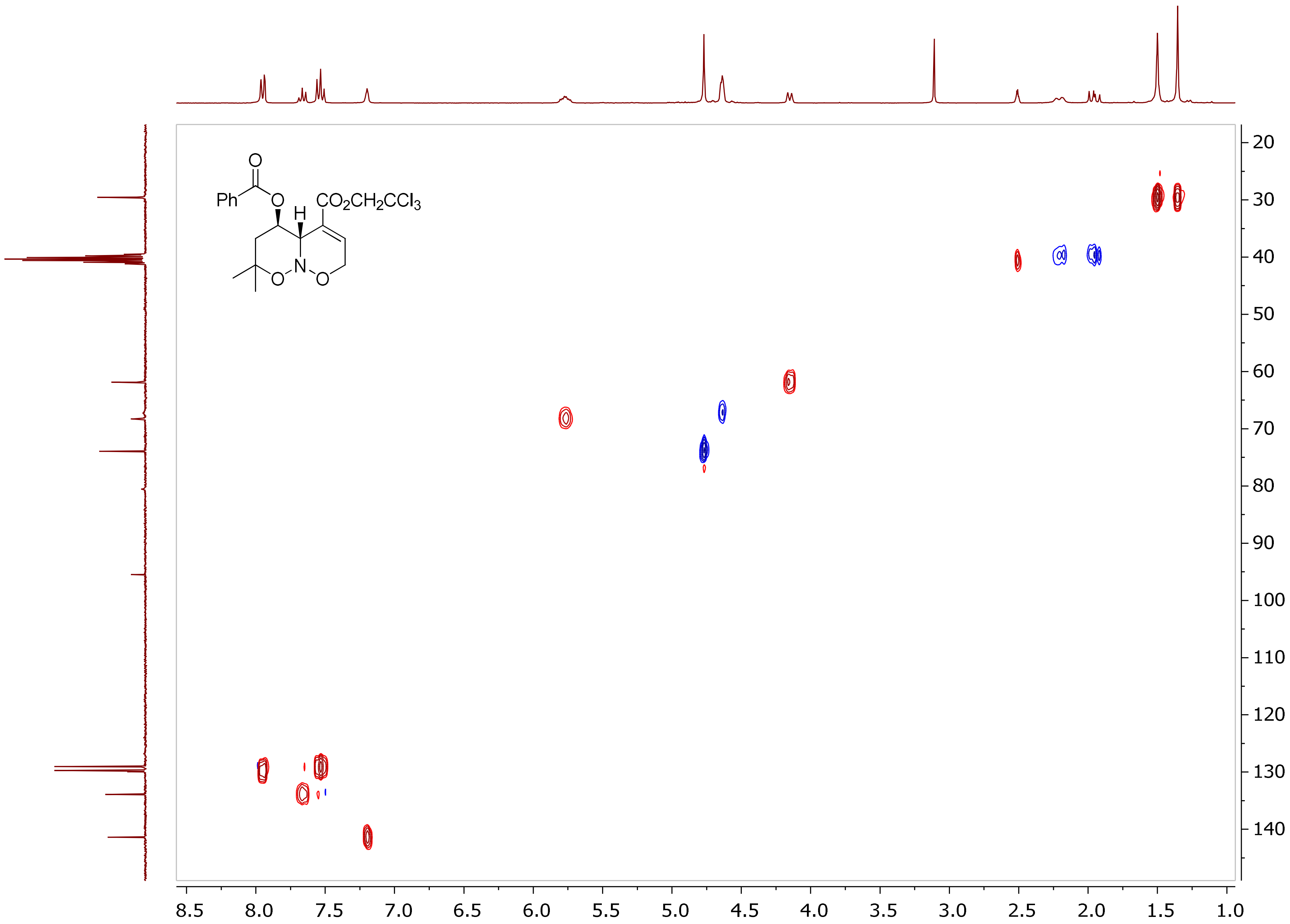

O
Ph
O
H
CO2CH2CCl3
O
N
O
20
30
40
50
60
70
80
90
100
110
120
130
140
8.5
8.0
7.5
7.0
6.5
6.0
5.5
5.0
4.5
4.0
3.5
3.0
2.5
2.0
1.5
1.0

^{1}H-^{13}C HMBC

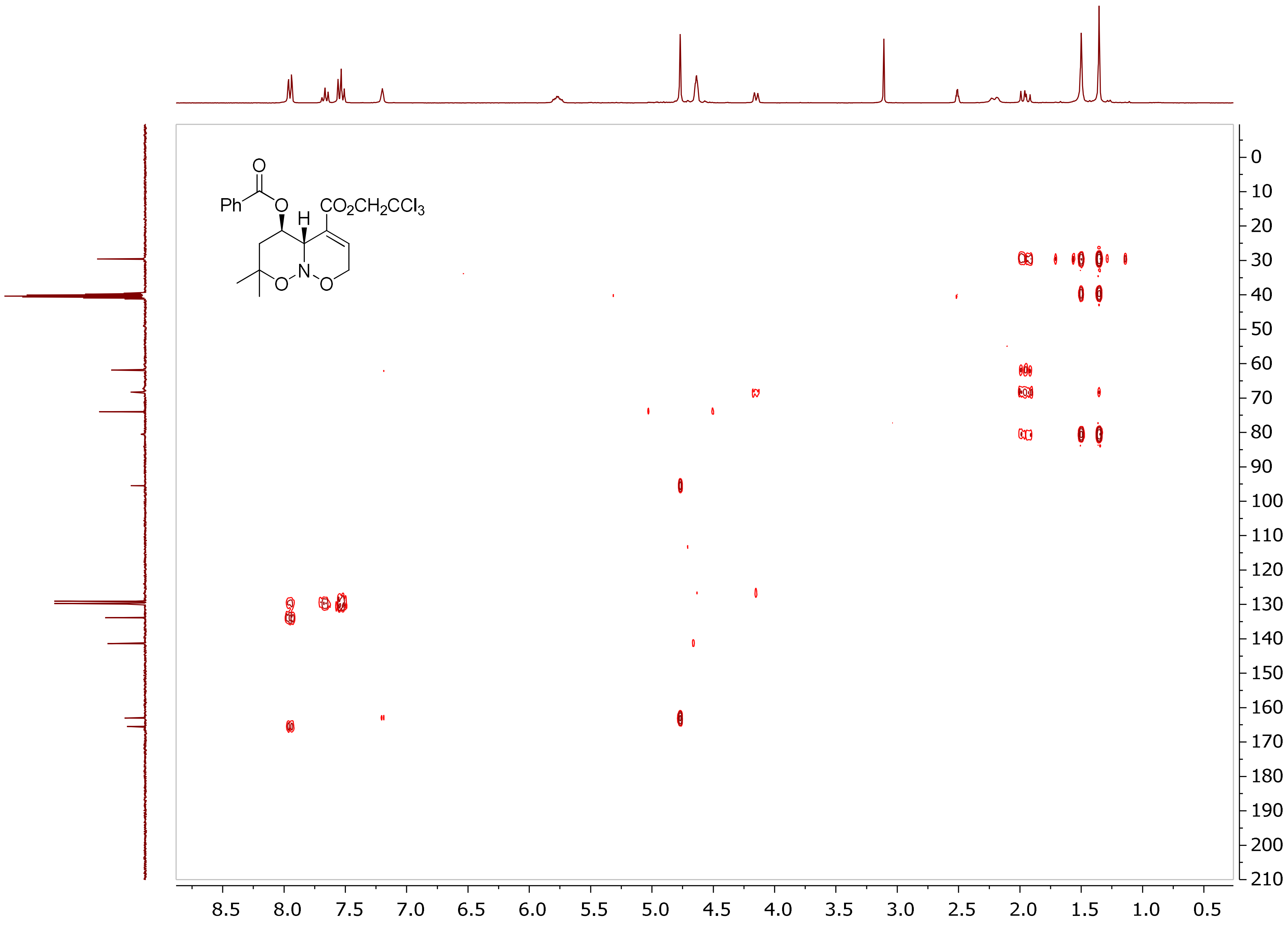

^{1}H-^{1}H NOESY

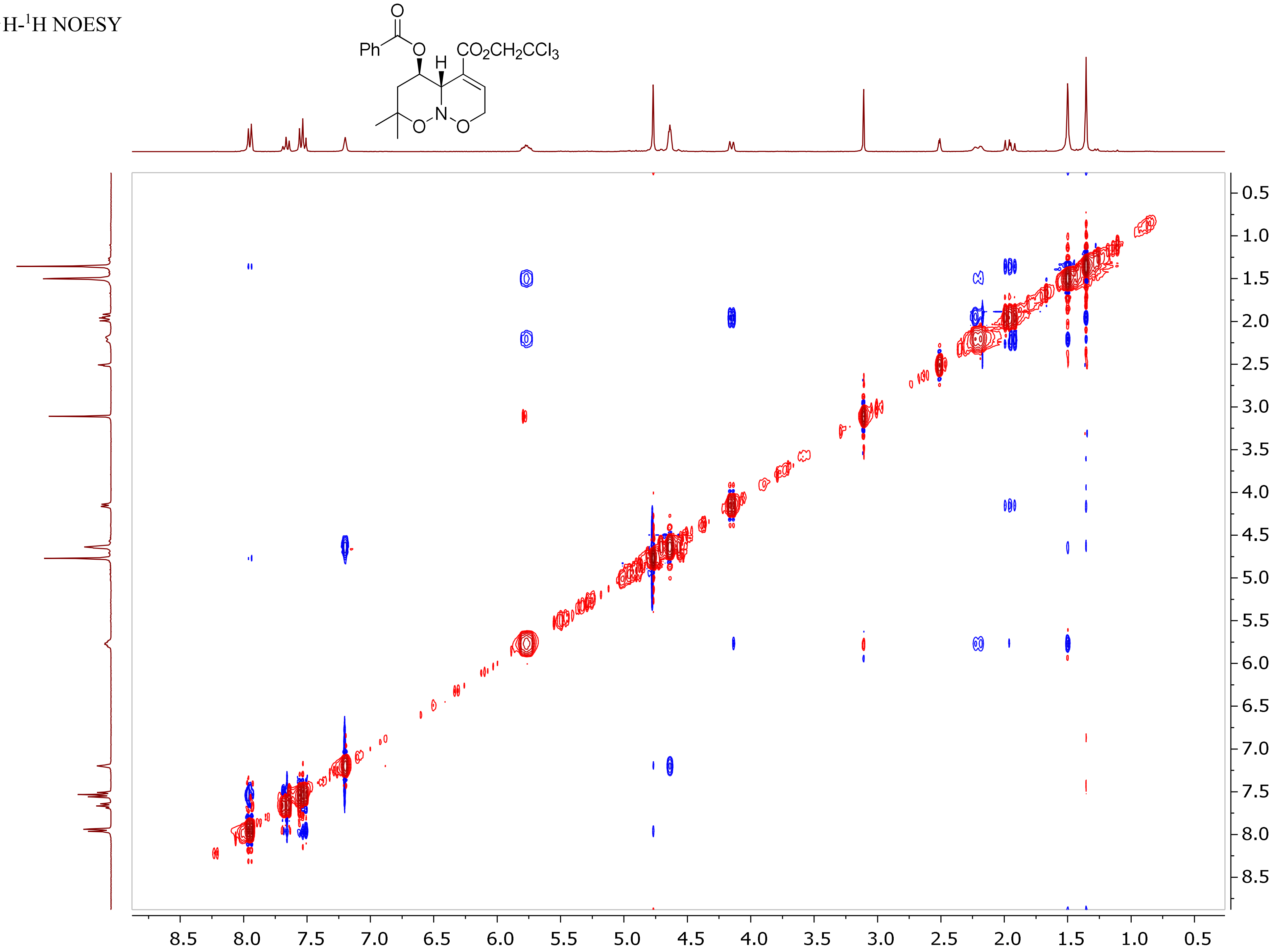
Ph
O
O
H
CO2CH2CCl3
N
O
O
8.5 8.0 7.5 7.0 6.5 6.0 5.5 5.0 4.5 4.0 3.5 3.0 2.5 2.0 1.5 1.0 0.5
0.5 1.0 1.5 2.0 2.5 3.0 3.5 4.0 4.5 5.0 5.5 6.0 6.5 7.0 7.5 8.0 8.5

2,2,2-Trichloroethyl (4aR*,5R*)-7,7-dimethyl-5-phenethyl-4a,5,6,7-tetrahydro-2H-[1,2]oxazino[2,3-b][1,2]oxazine-4-carboxylate 3m
^{1}H NMR (300 MHz, $CDCl_3$)

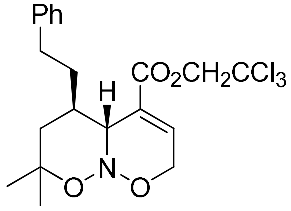

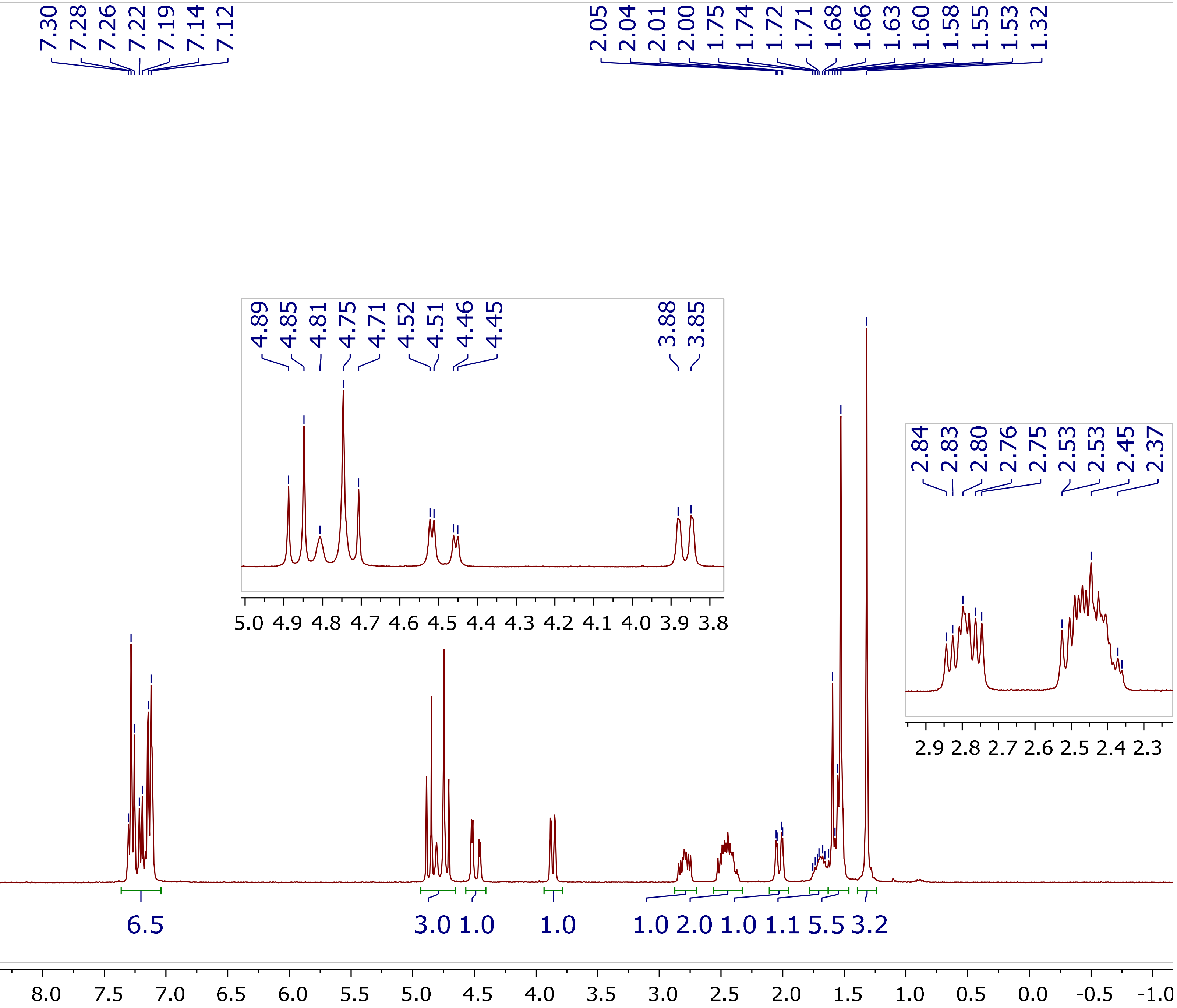

^{13}C NMR (75 MHz, $CDCl_3$)

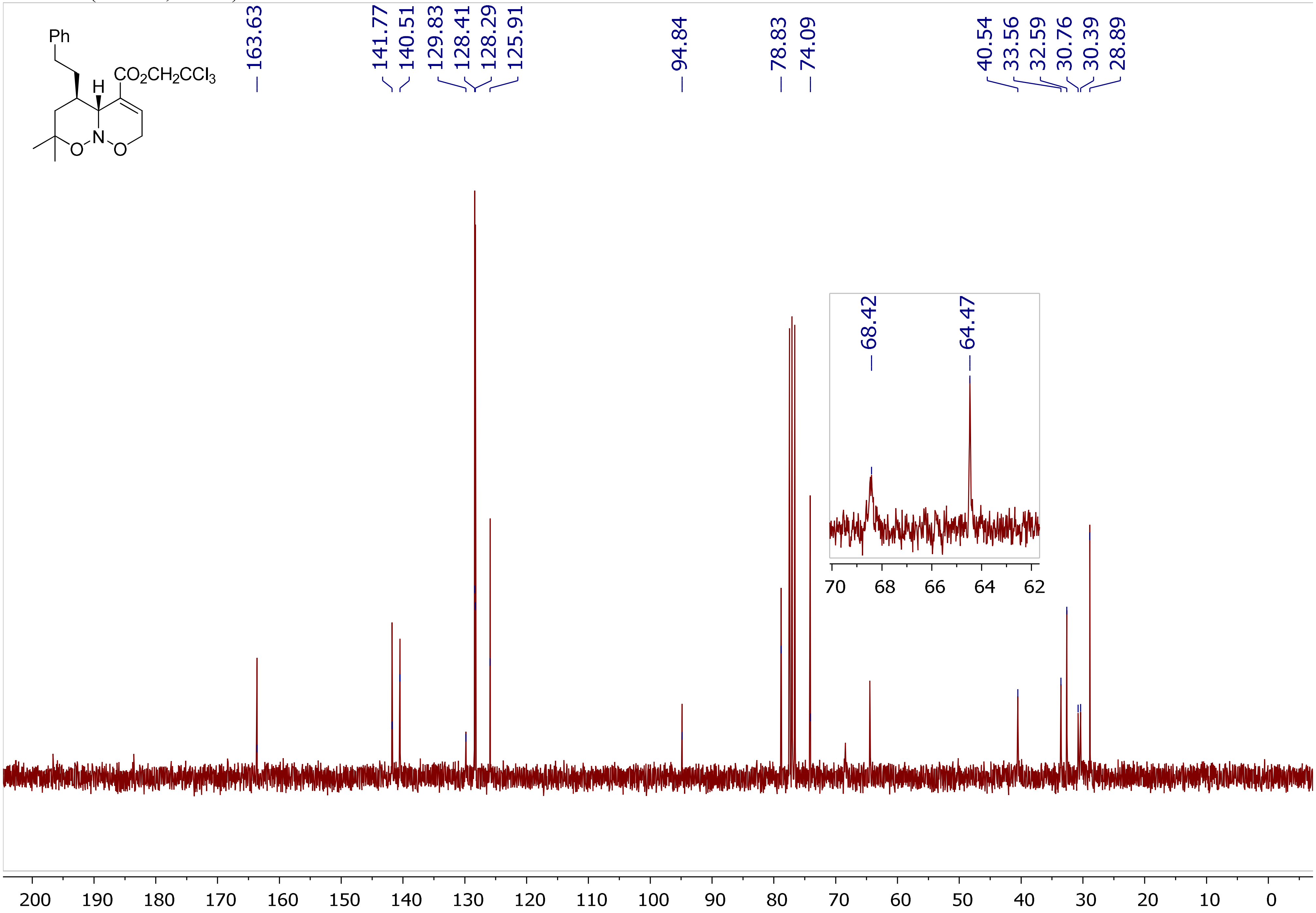

^{13}C DEPT 135 (75 MHz, $CDCl_3$)

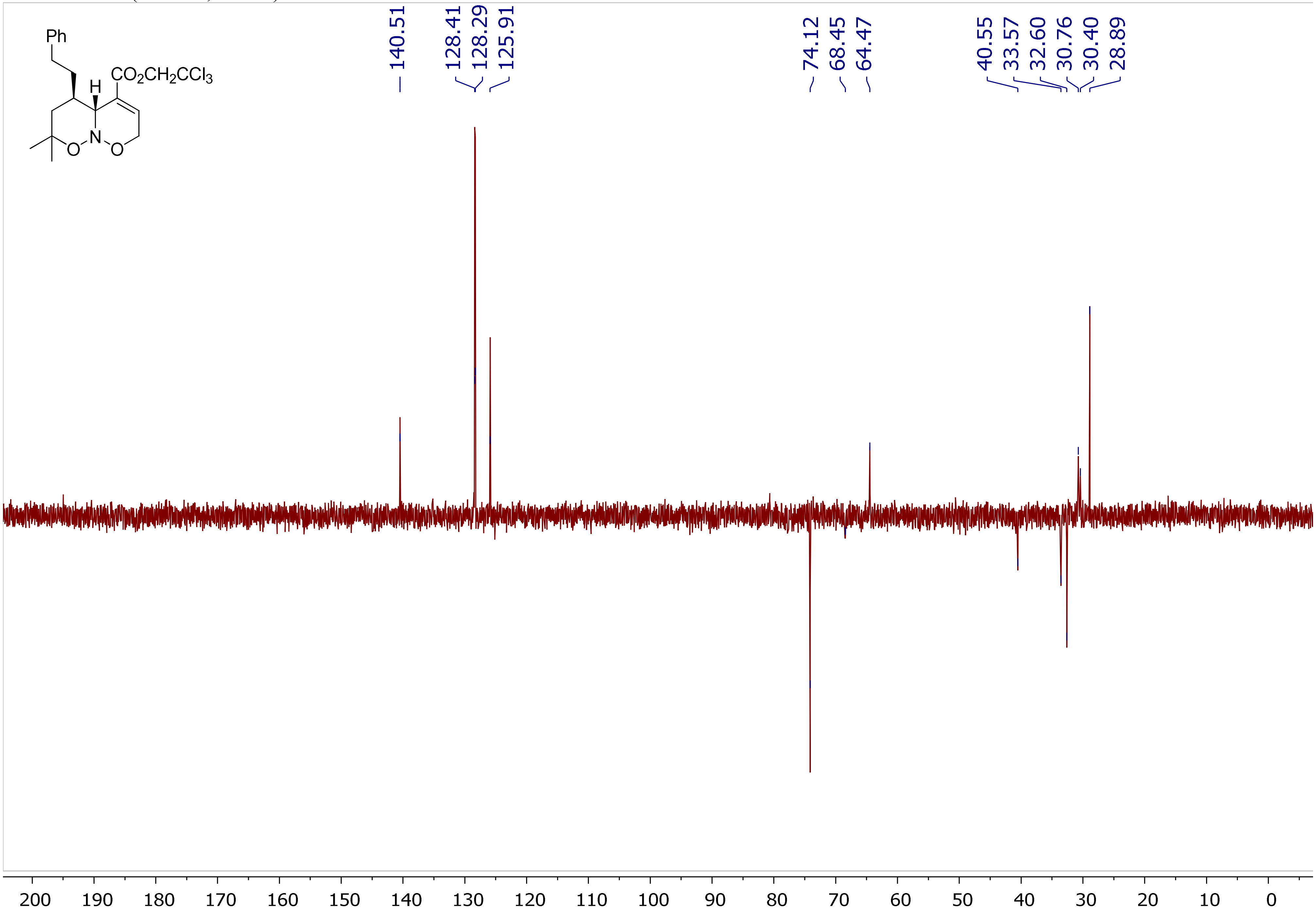

^1H-^1H COSY

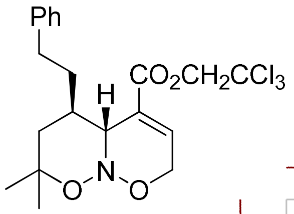

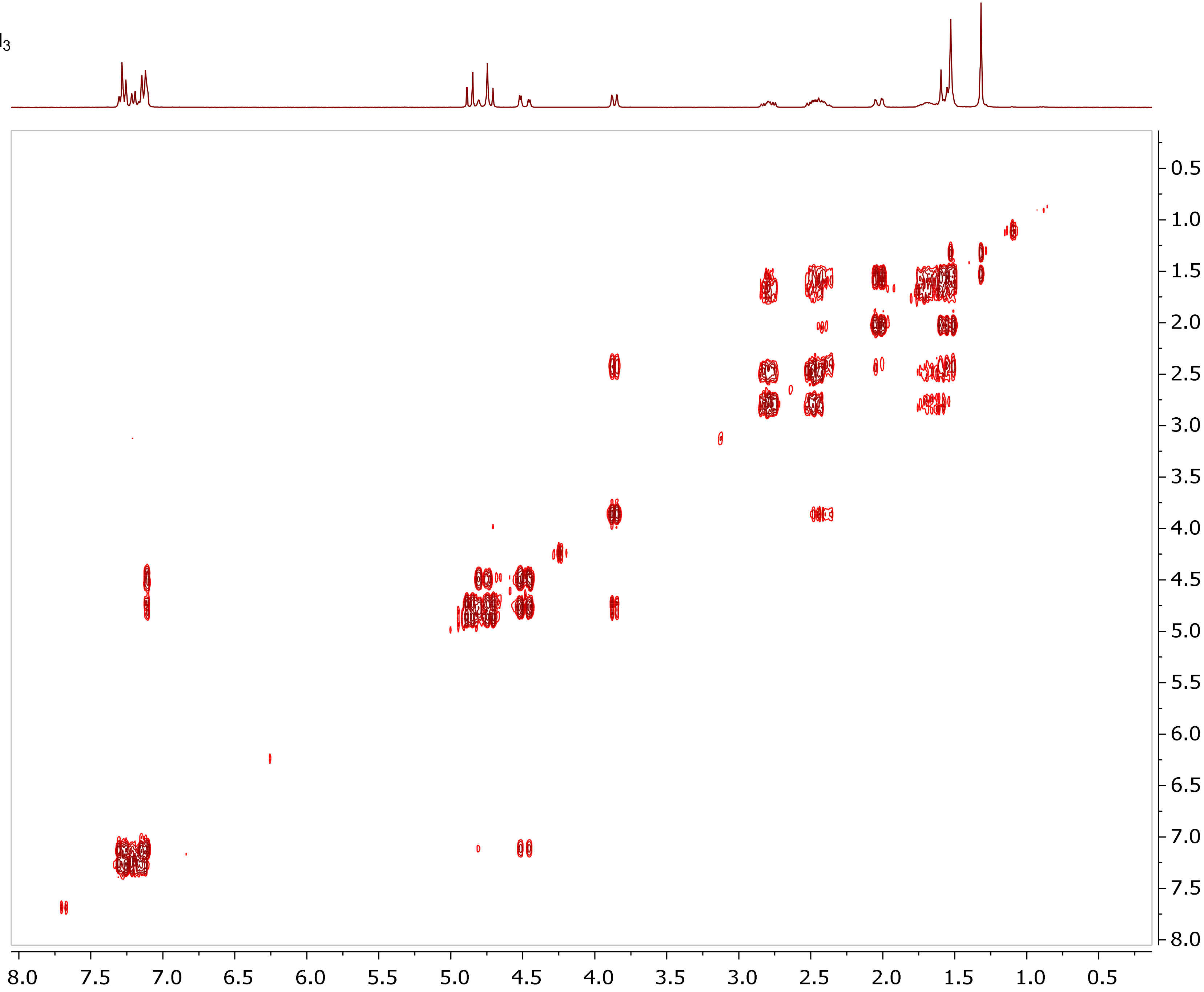

^{1}H-^{13}C HSQC

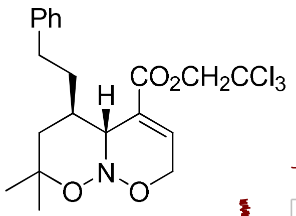

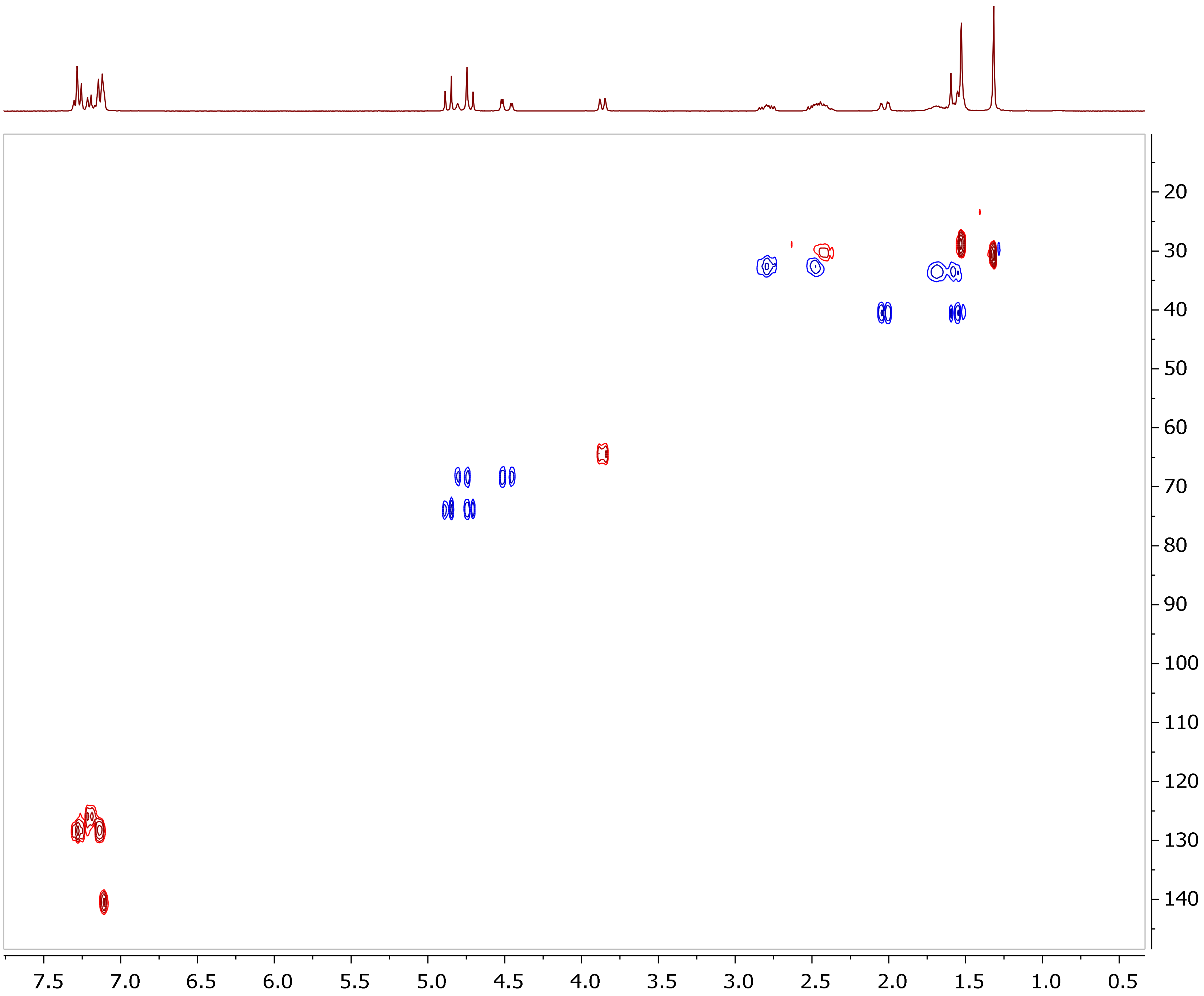
7.5
7.0
6.5
6.0
5.5
5.0
4.5
4.0
3.5
3.0
2.5
2.0
1.5
1.0
0.5
20
30
40
50
60
70
80
90
100
110
120
130
140

2,2,2-Trichloroethyl (4aR*,5S*,7R*)-5,7-diphenyl-4a,5,6,7-tetrahydro-2H-[1,2]oxazino[2,3-b][1,2]oxazine-4-carboxylate 3n

^{1}H NMR (300 MHz, $CDCl_3$)

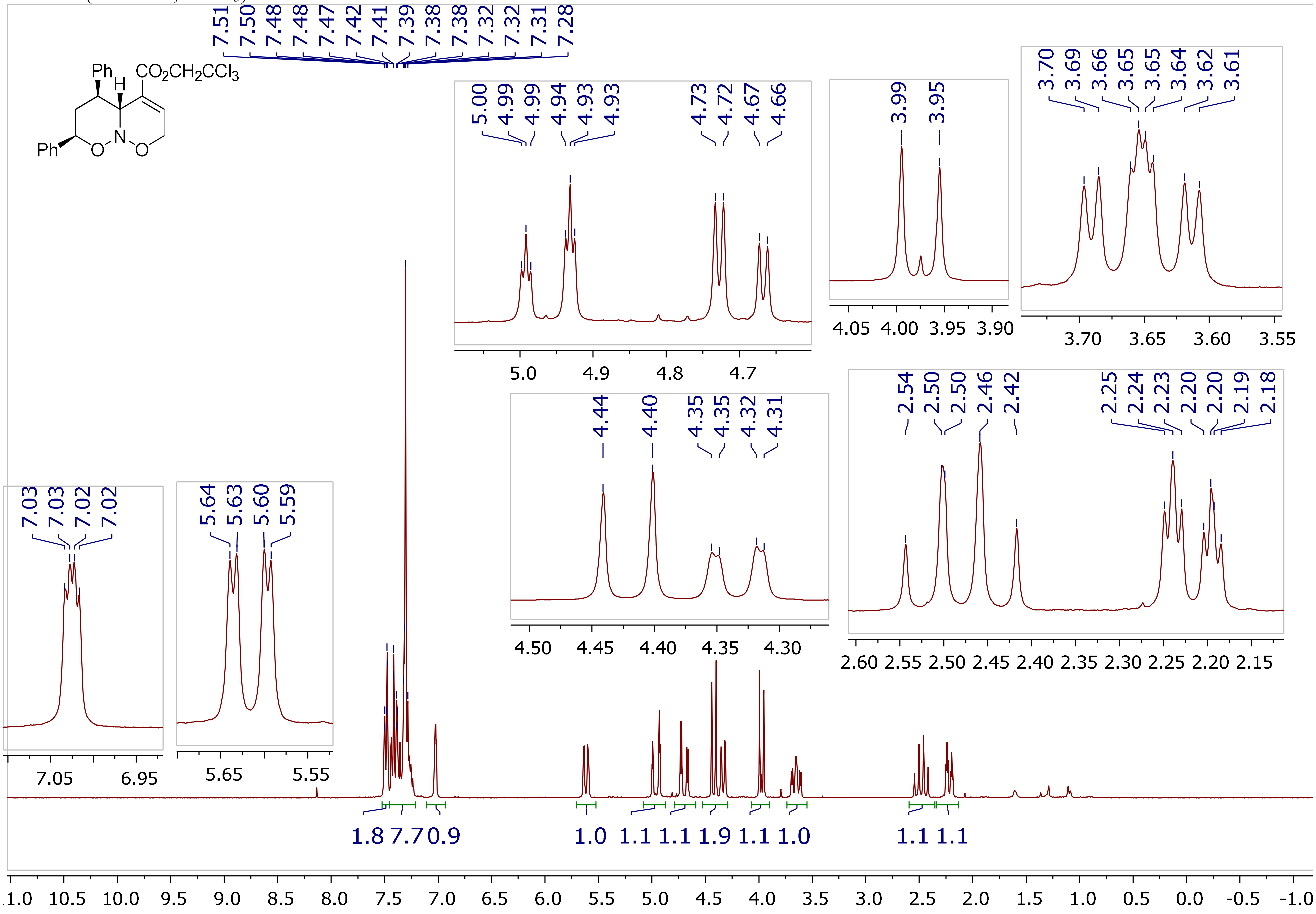

^{13}C NMR (75 MHz, $CDCl_3$)

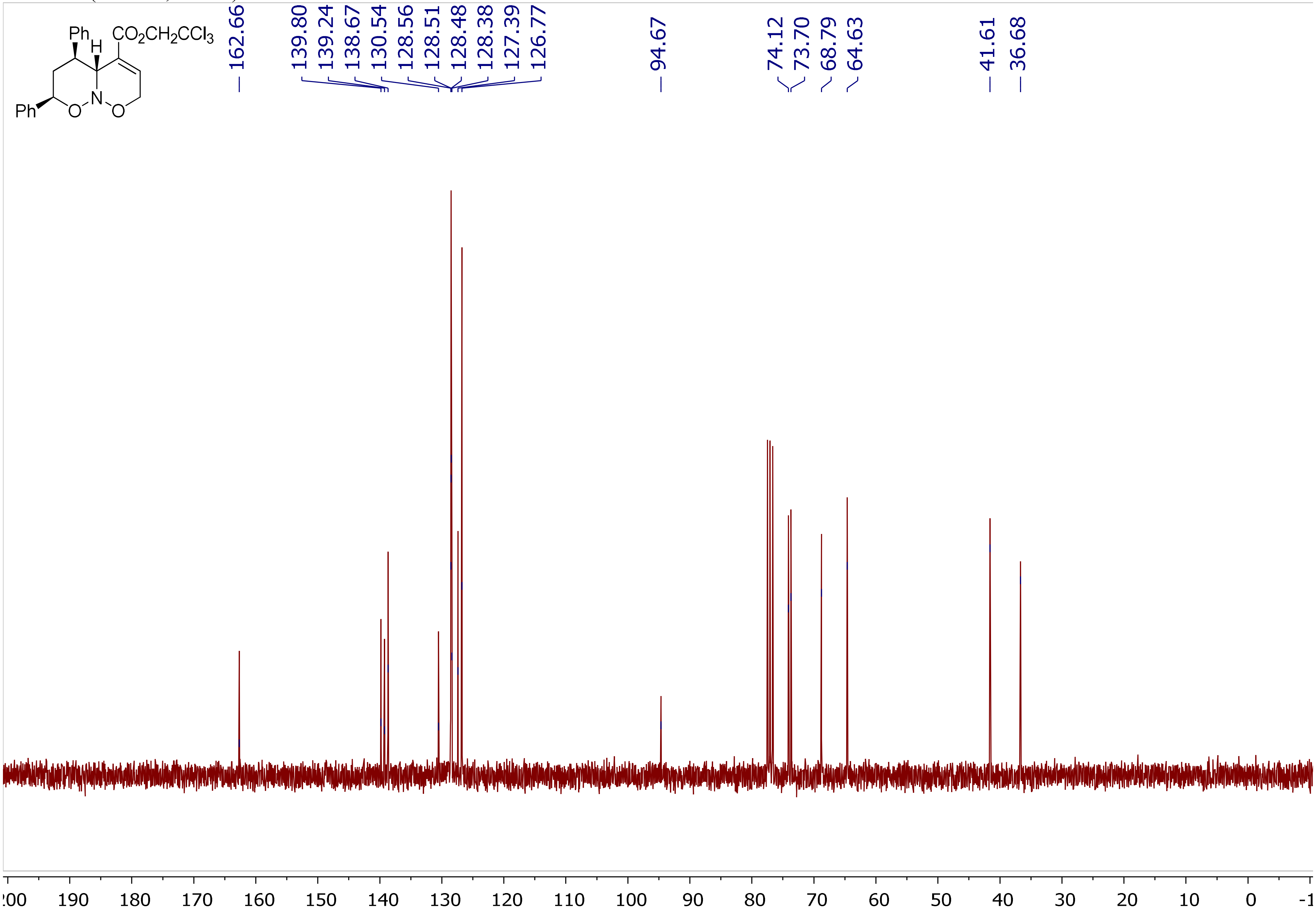

^{13}C DEPT 135 (75 MHz, $CDCl_3$)

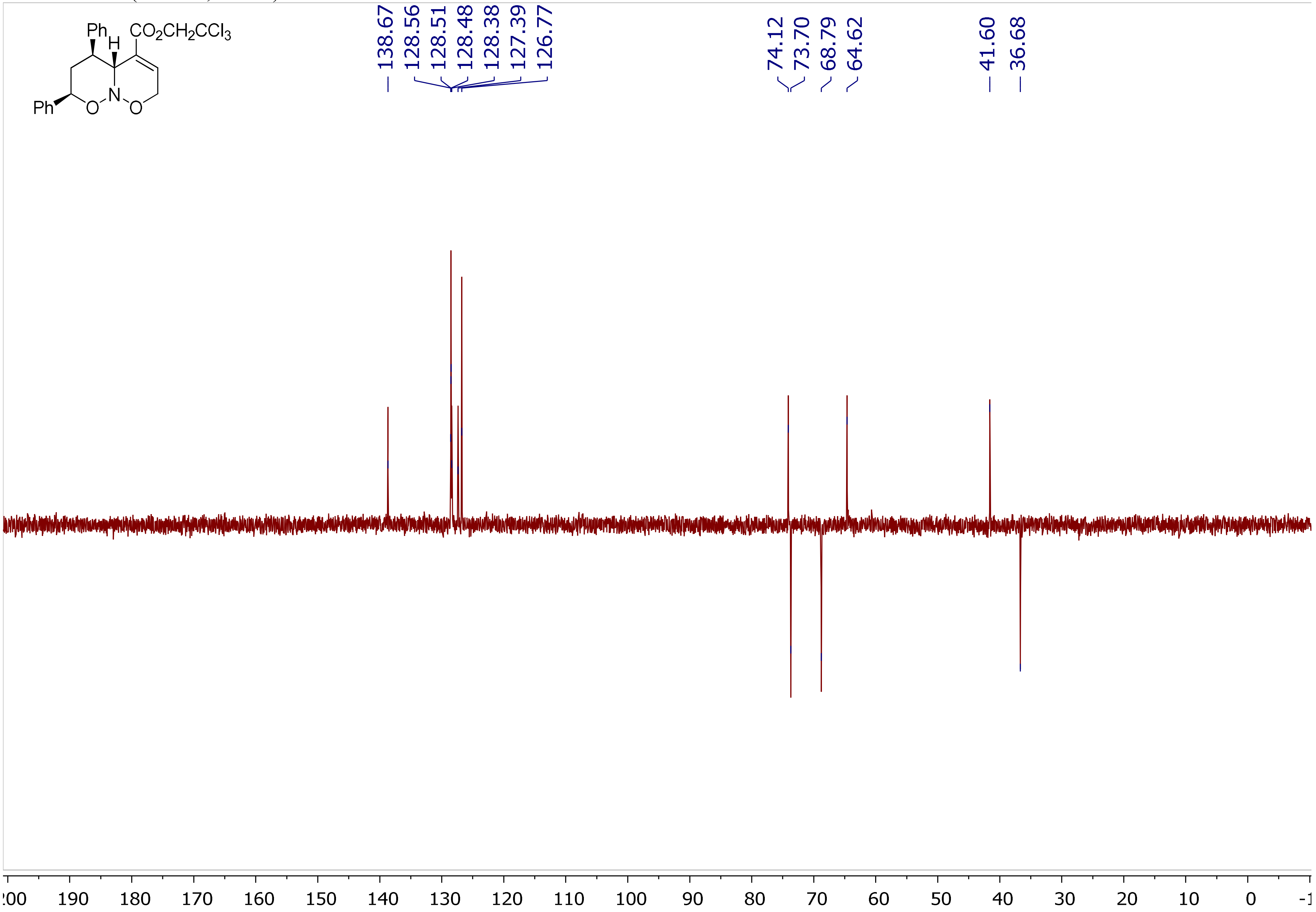

^1H-^1H COSY

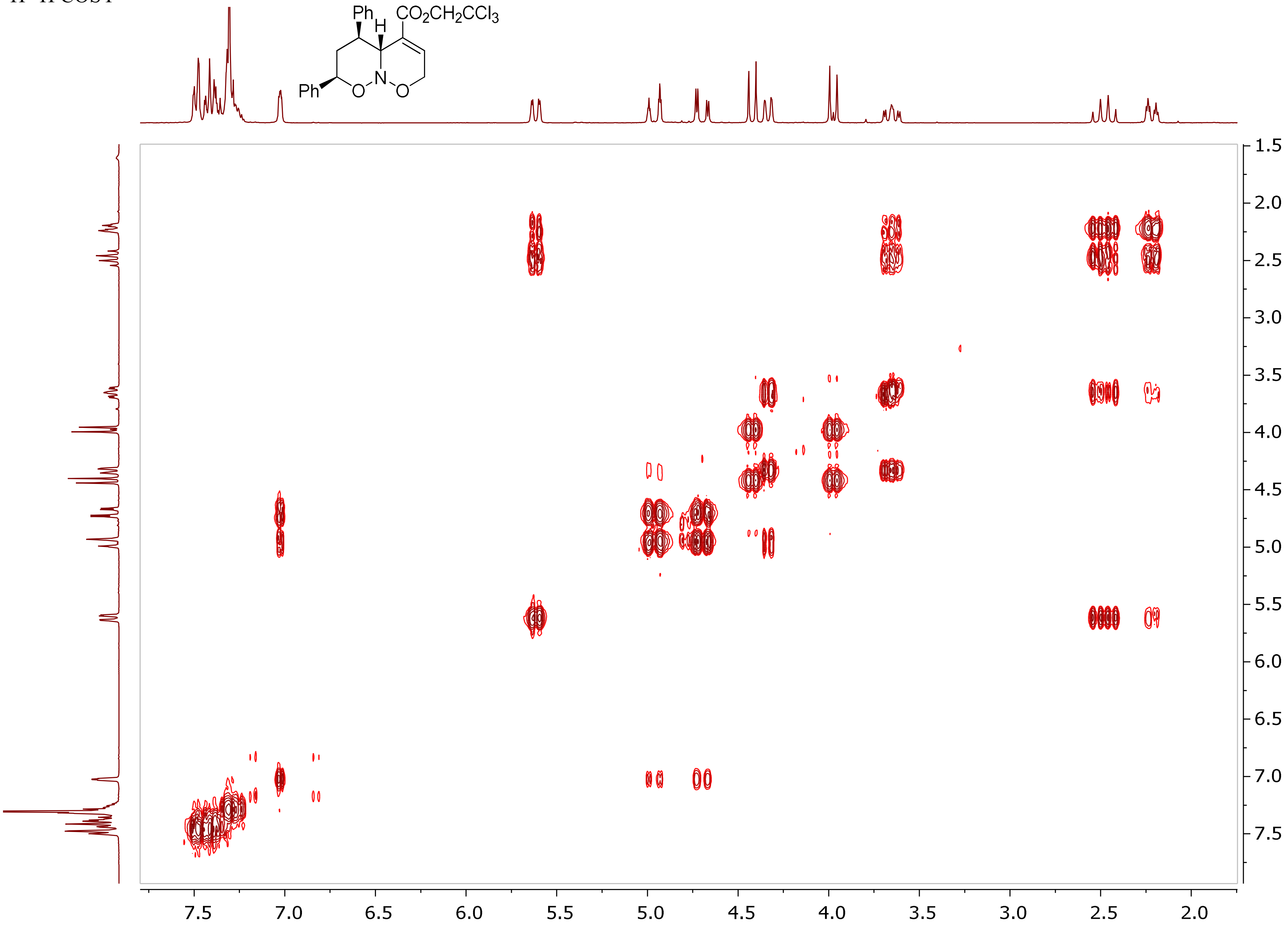

^{1}H-^{13}C HSQC

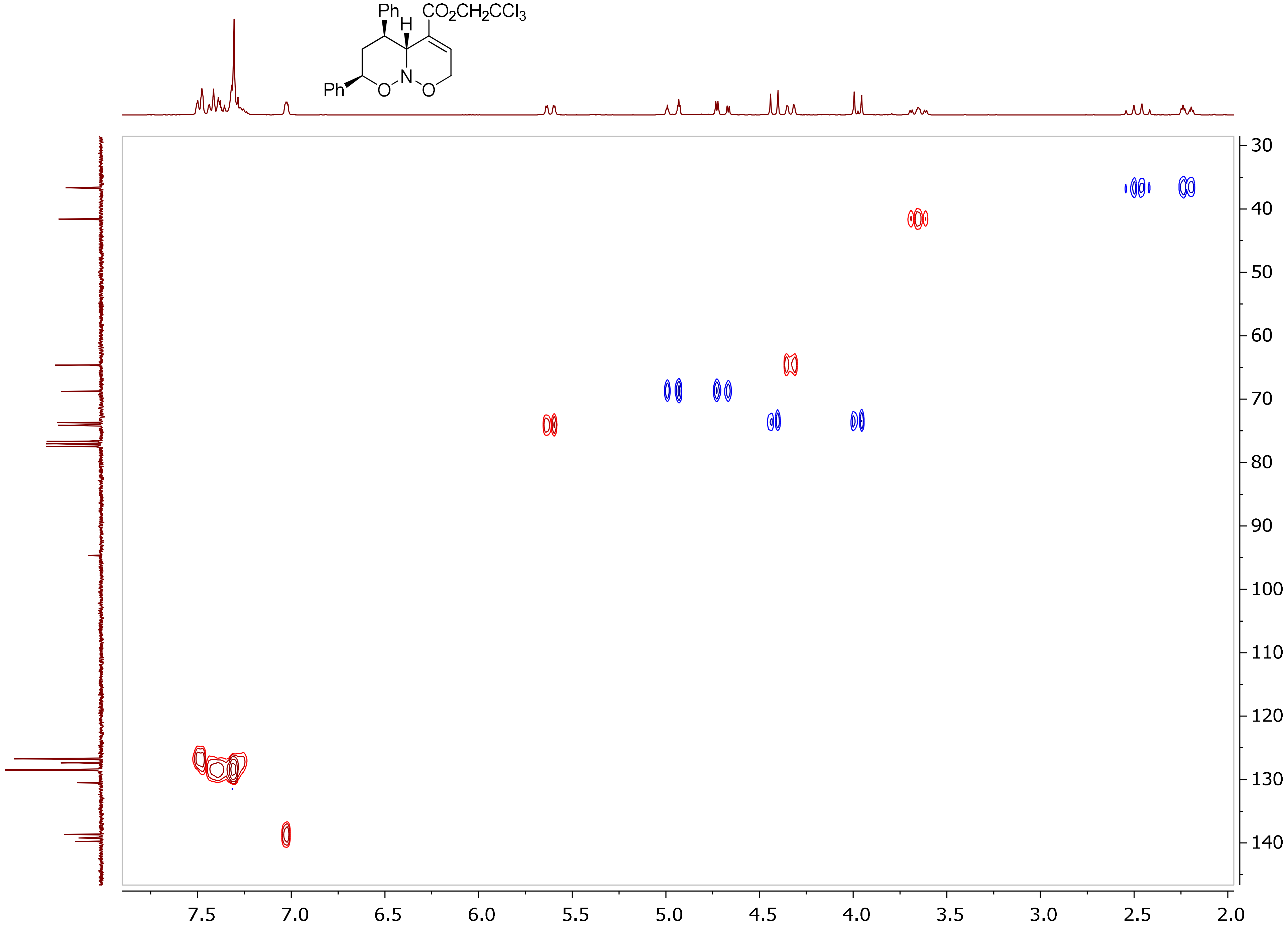
Ph
H
$CO_2CH_2CCl_3$
N
O
O
Ph
7.5
7.0
6.5
6.0
5.5
5.0
4.5
4.0
3.5
3.0
2.5
2.0
30
40
50
60
70
80
90
100
110
120
130
140

^1H-^1H NOESY

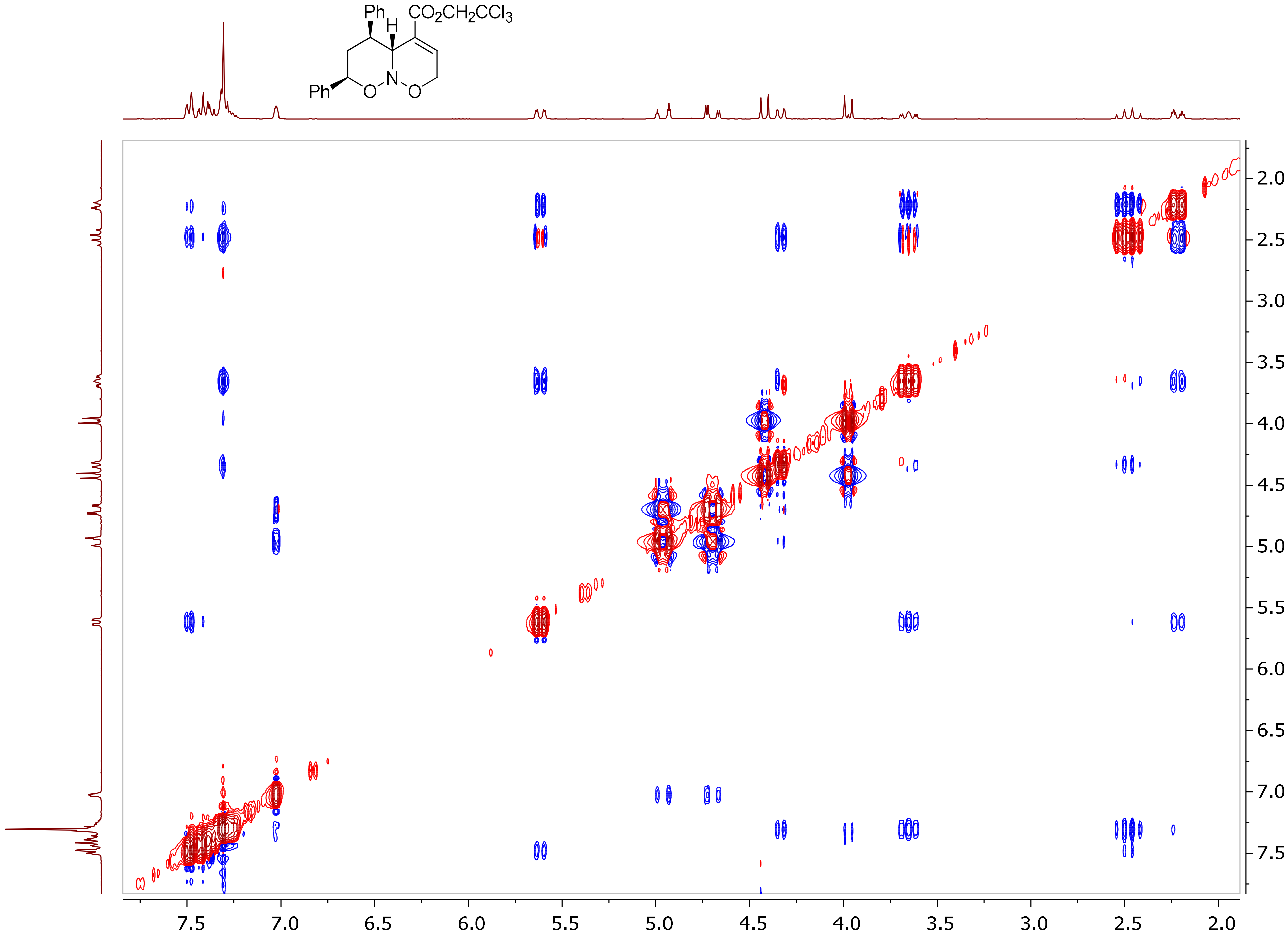

2,2,2-Trichloroethyl (4aR*,5S*,7S*)-7-ethoxy-5-(4-methoxyphenyl)-4a,5,6,7-tetrahydro-2H-[1,2]oxazino[2,3-b][1,2]oxazine-4-carboxylate 3o

^{1}H NMR (300 MHz, $CDCl_3$)

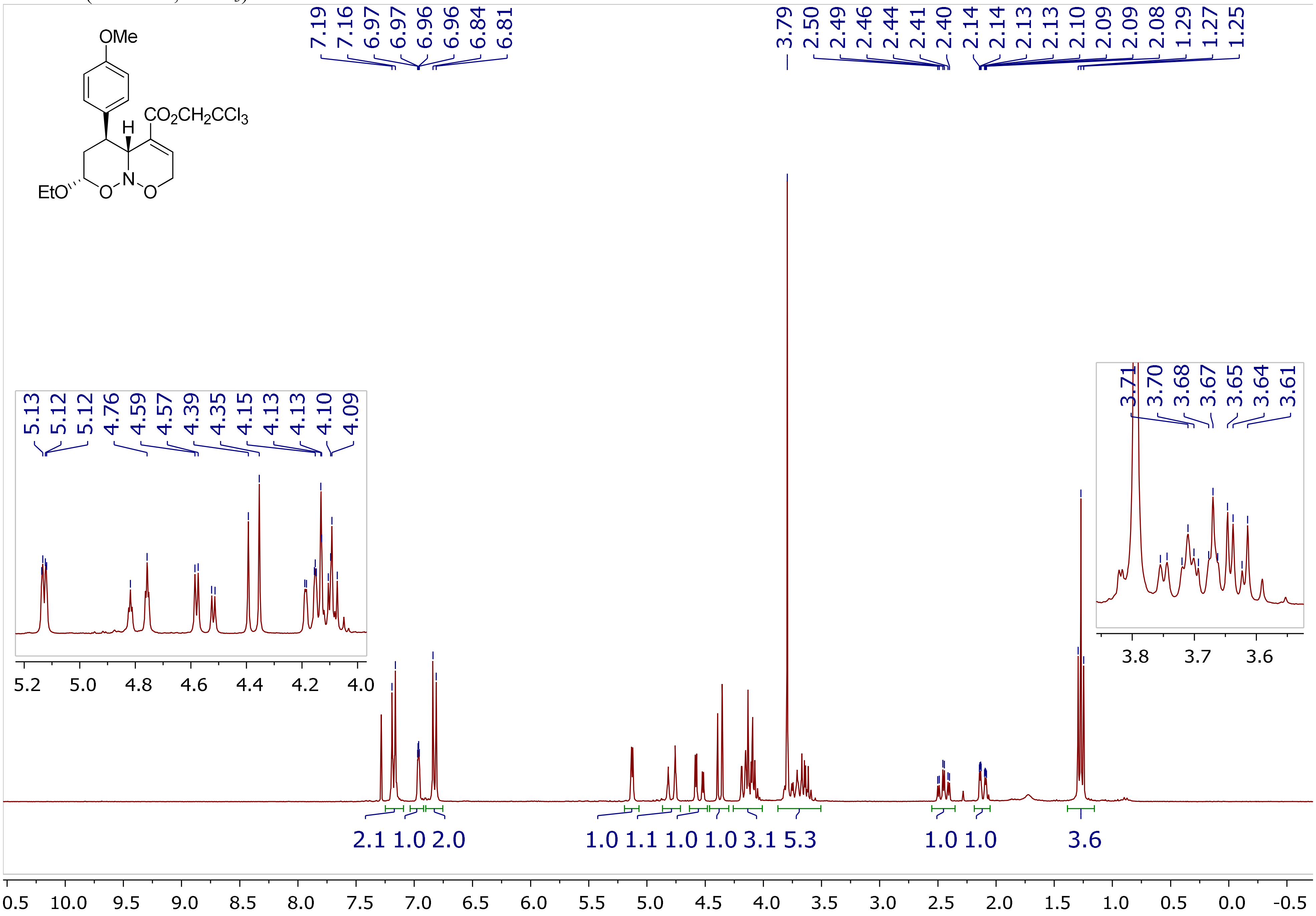

^{13}C NMR (75 MHz, $CDCl_3$)

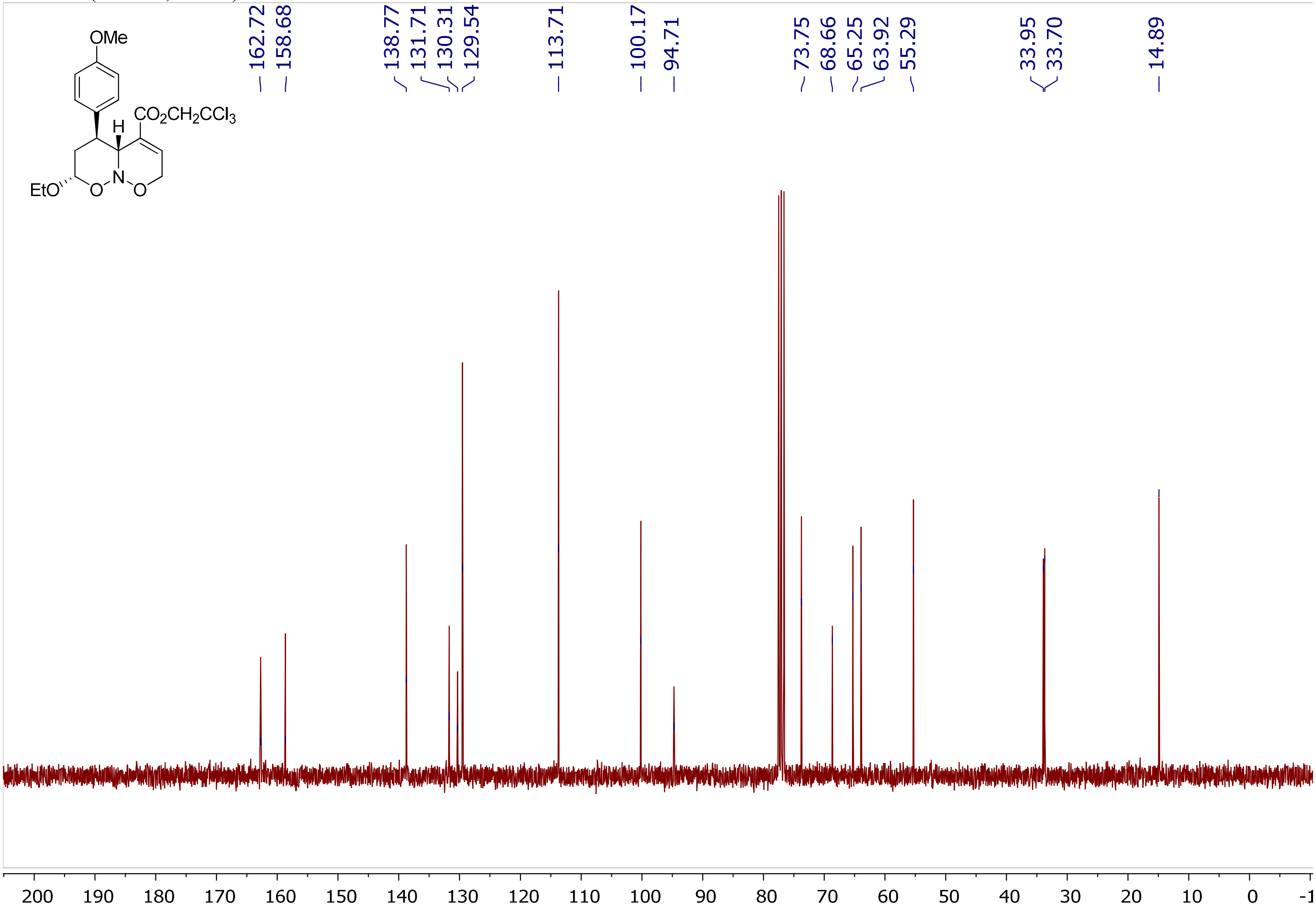

^{13}C DEPT 135 (75 MHz, $CDCl_3$)

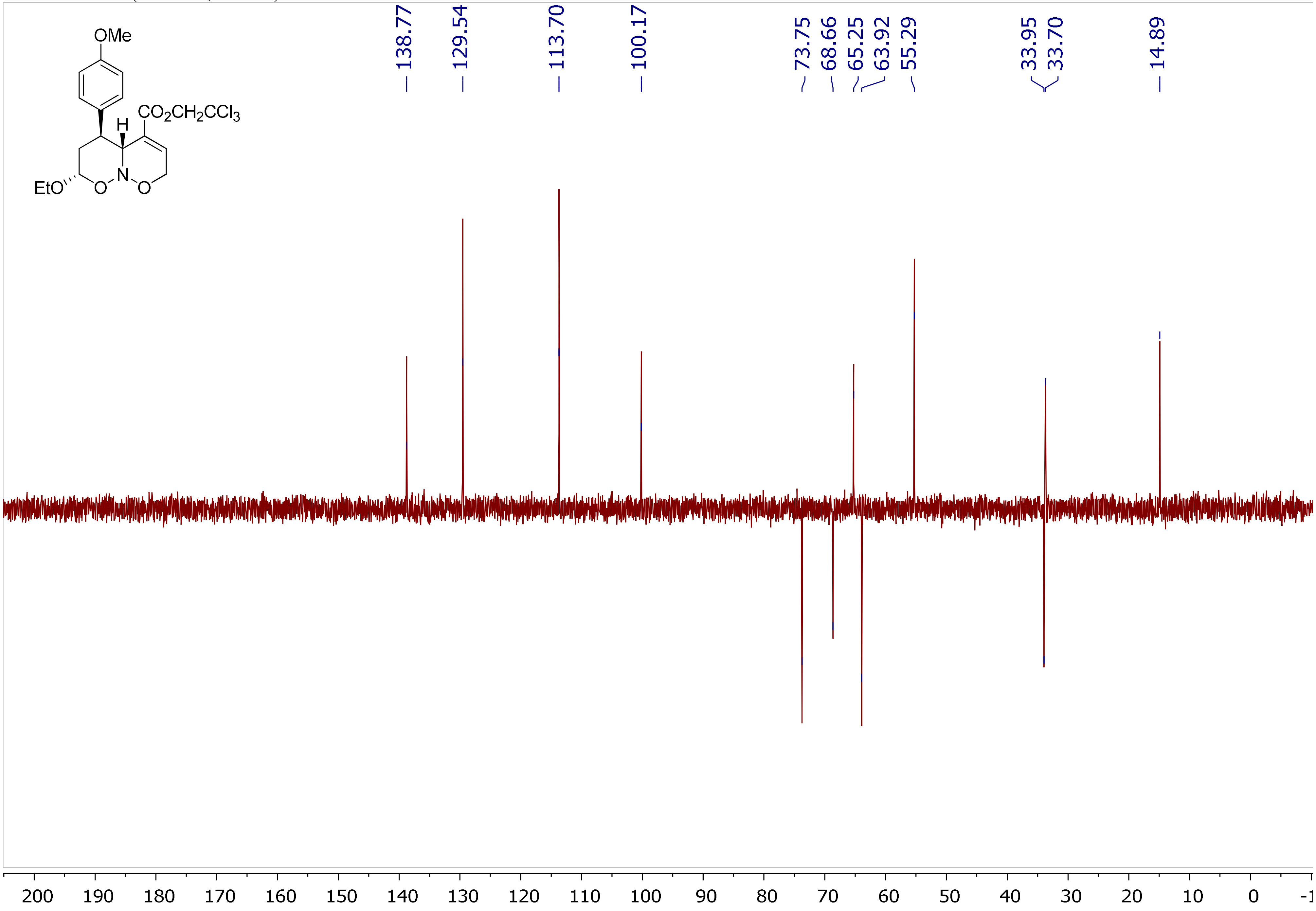

^1H-^1H COSY

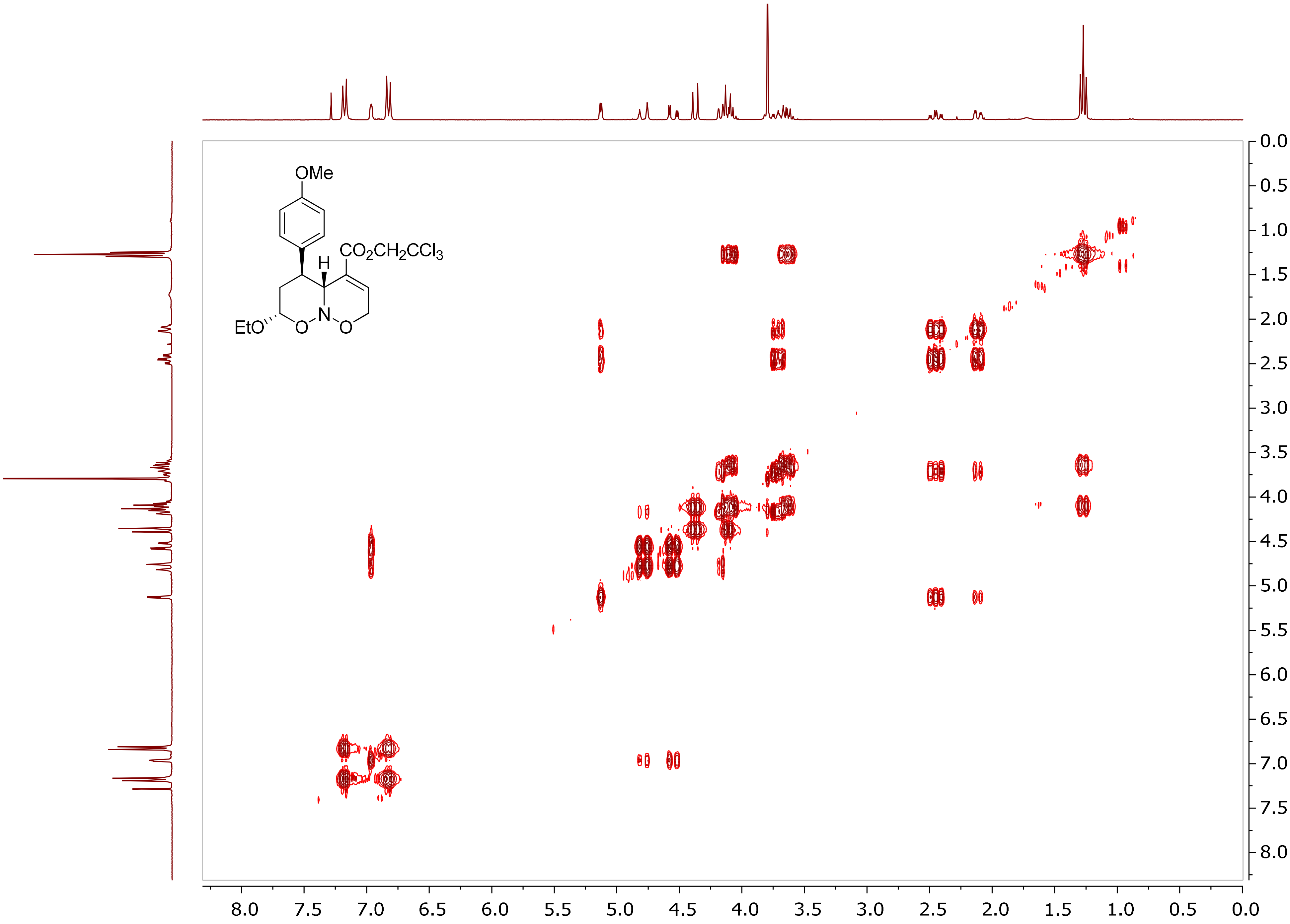
OMe
$CO_2CH_2CCl_3$
H
N
EtO
O
O
0.0
0.5
1.0
1.5
2.0
2.5
3.0
3.5
4.0
4.5
5.0
5.5
6.0
6.5
7.0
7.5
8.0

^1H-^{13}C HSQC

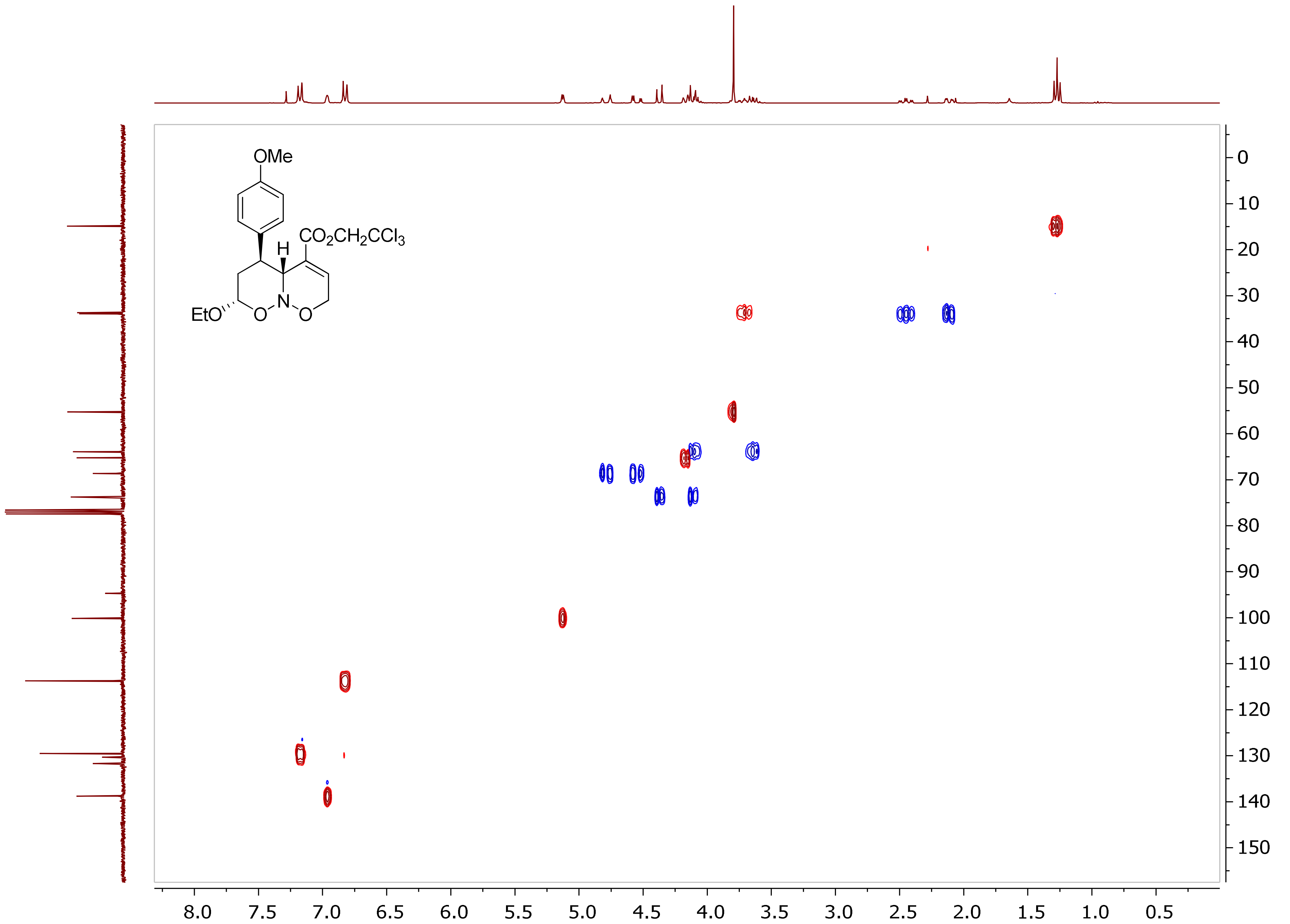

OMe
$CO_2CH_2CCl_3$
H
N
EtO
O
O
0
10
20
30
40
50
60
70
80
90
100
110
120
130
140
150
8.0
7.5
7.0
6.5
6.0
5.5
5.0
4.5
4.0
3.5
3.0
2.5
2.0
1.5
1.0
0.5

^1H-^{13}C HMBC

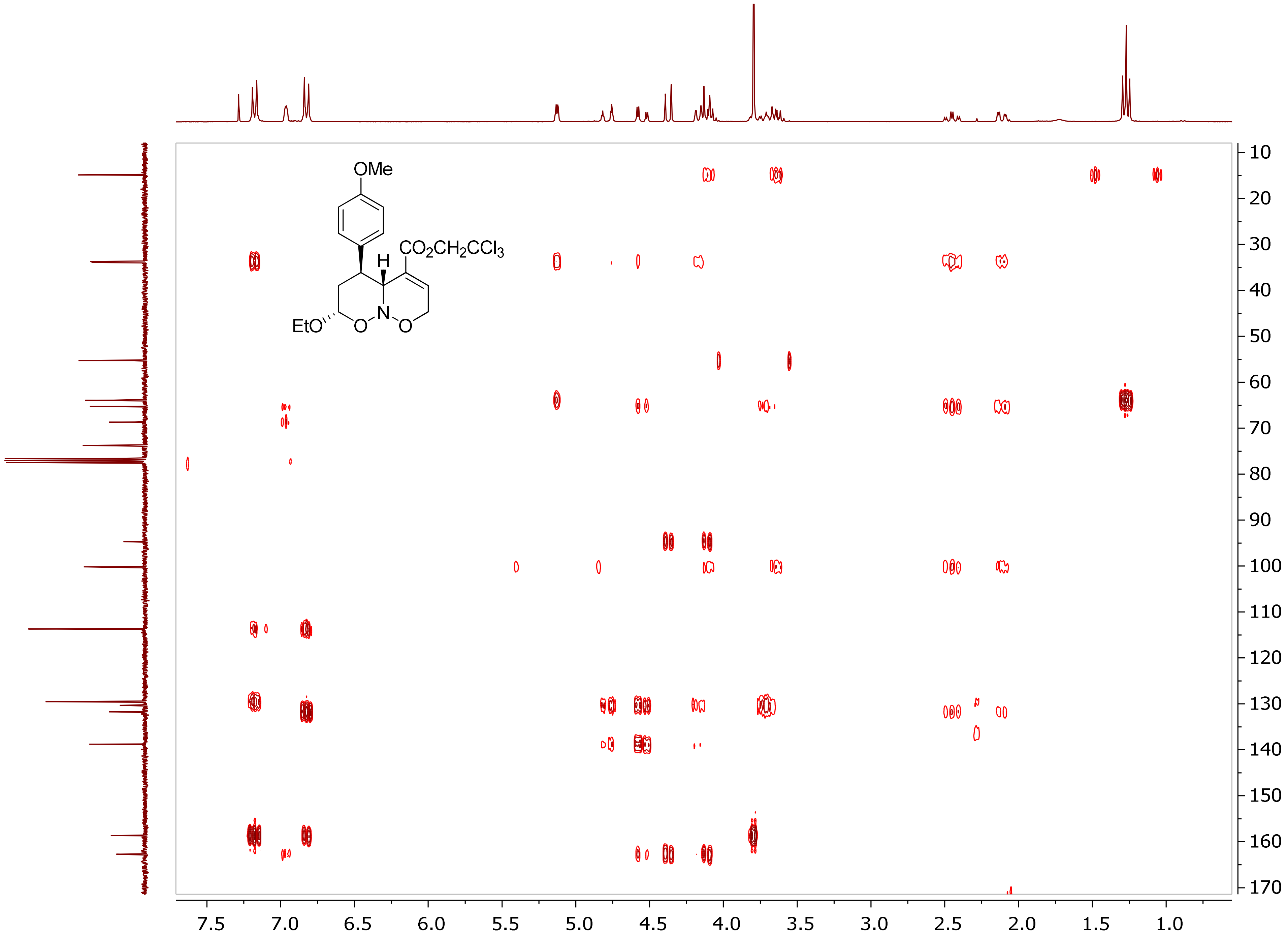
OMe
$CO_2CH_2CCl_3$
H
EtO
O
N
O
7.5
7.0
6.5
6.0
5.5
5.0
4.5
4.0
3.5
3.0
2.5
2.0
1.5
1.0
10
20
30
40
50
60
70
80
90
100
110
120
130
140
150
160
170

^1H-^1H NOESY

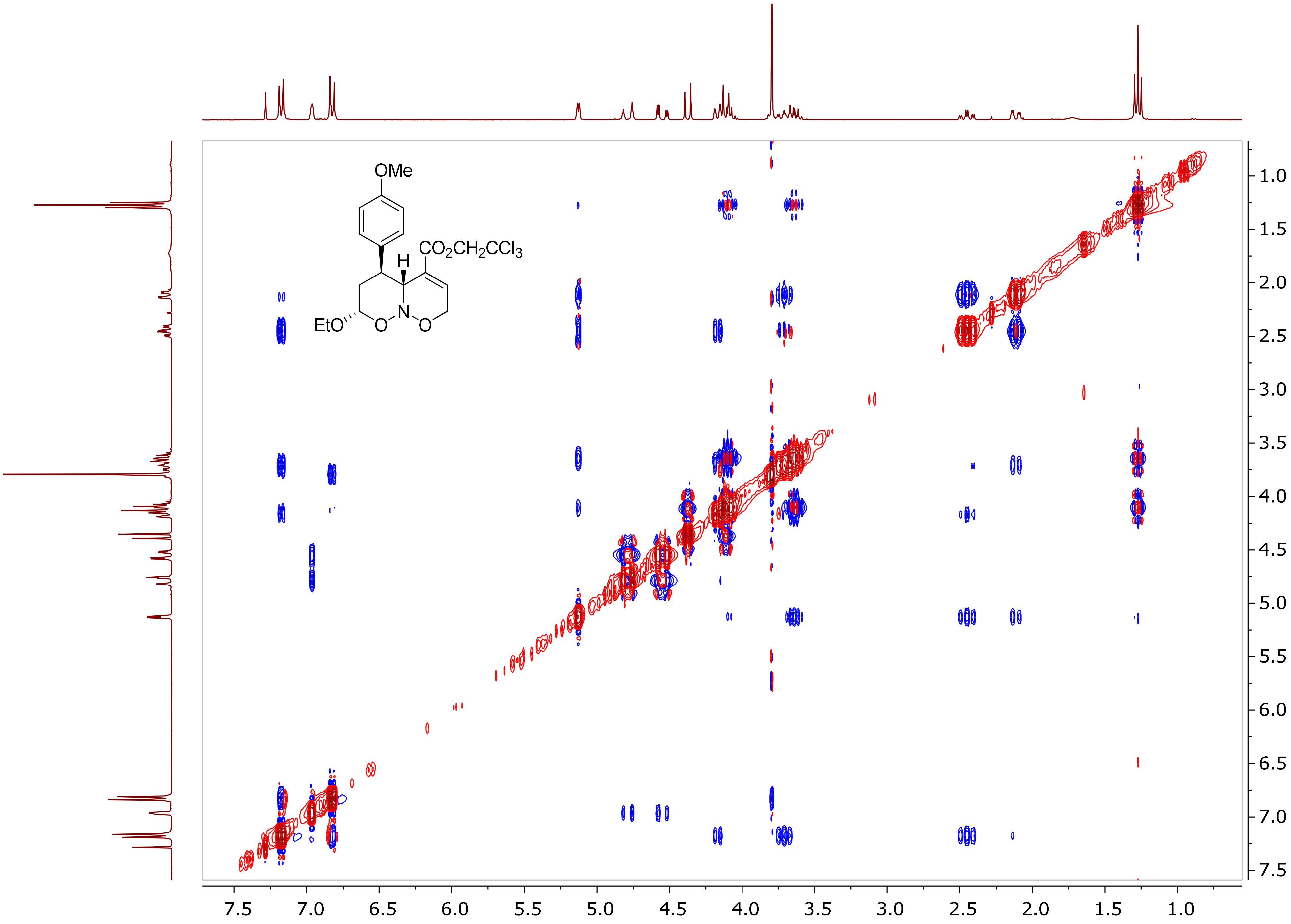

2,2,2-Trichloroethyl (4aR*,5S*,5aR*,9aR*)-5-(4-chlorophenyl)-4a,5,5a,6,7,8,9,9a-octahydro-2H-benzo[e][1,2]oxazino[2,3-b][1,2]oxazine-4-carboxylate 3p

^{1}H NMR (300 MHz, $CDCl_3$, 320 K)

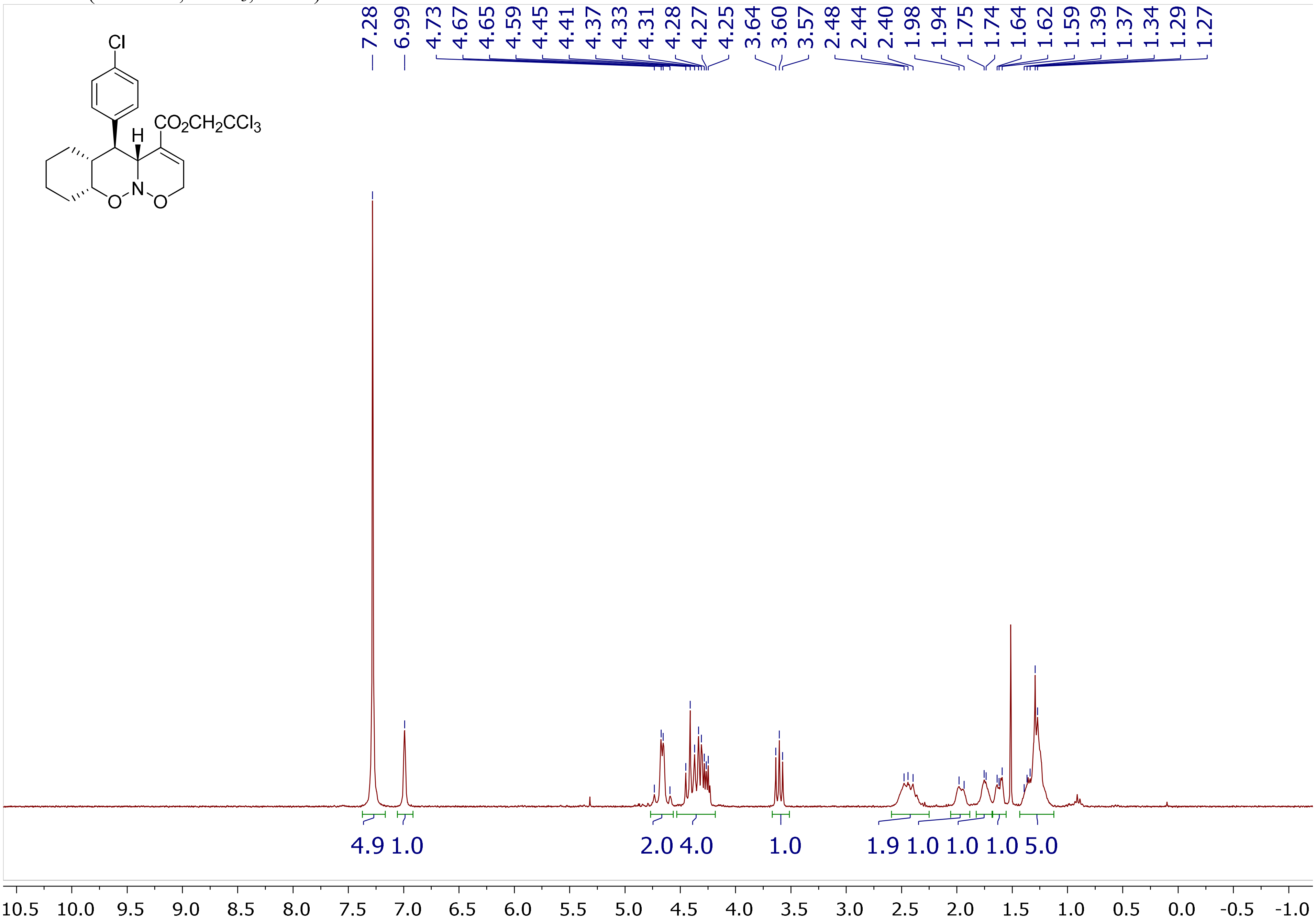

^{13}C NMR (75 MHz, $CDCl_3$, 320 K)

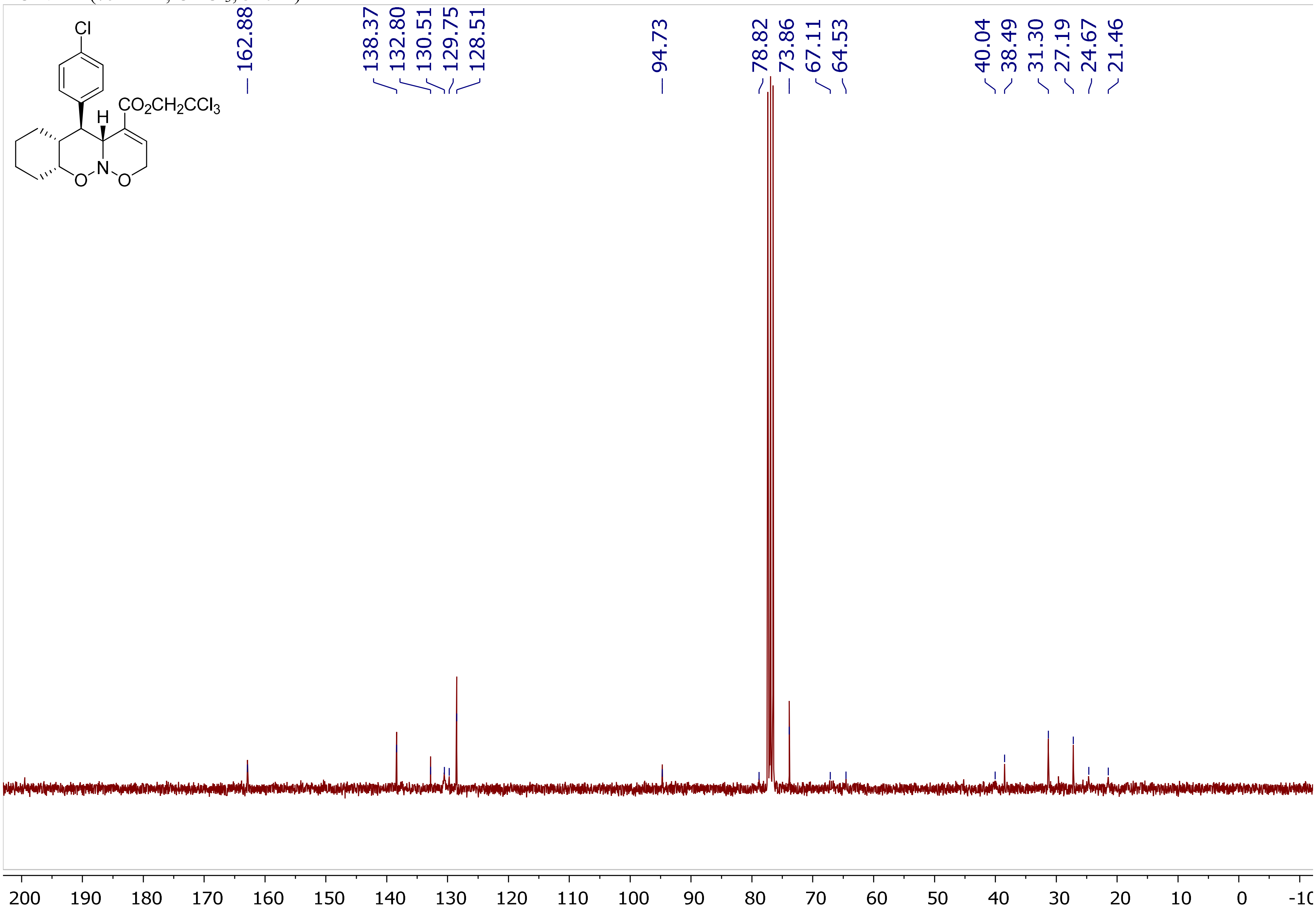

^1H-^1H COSY

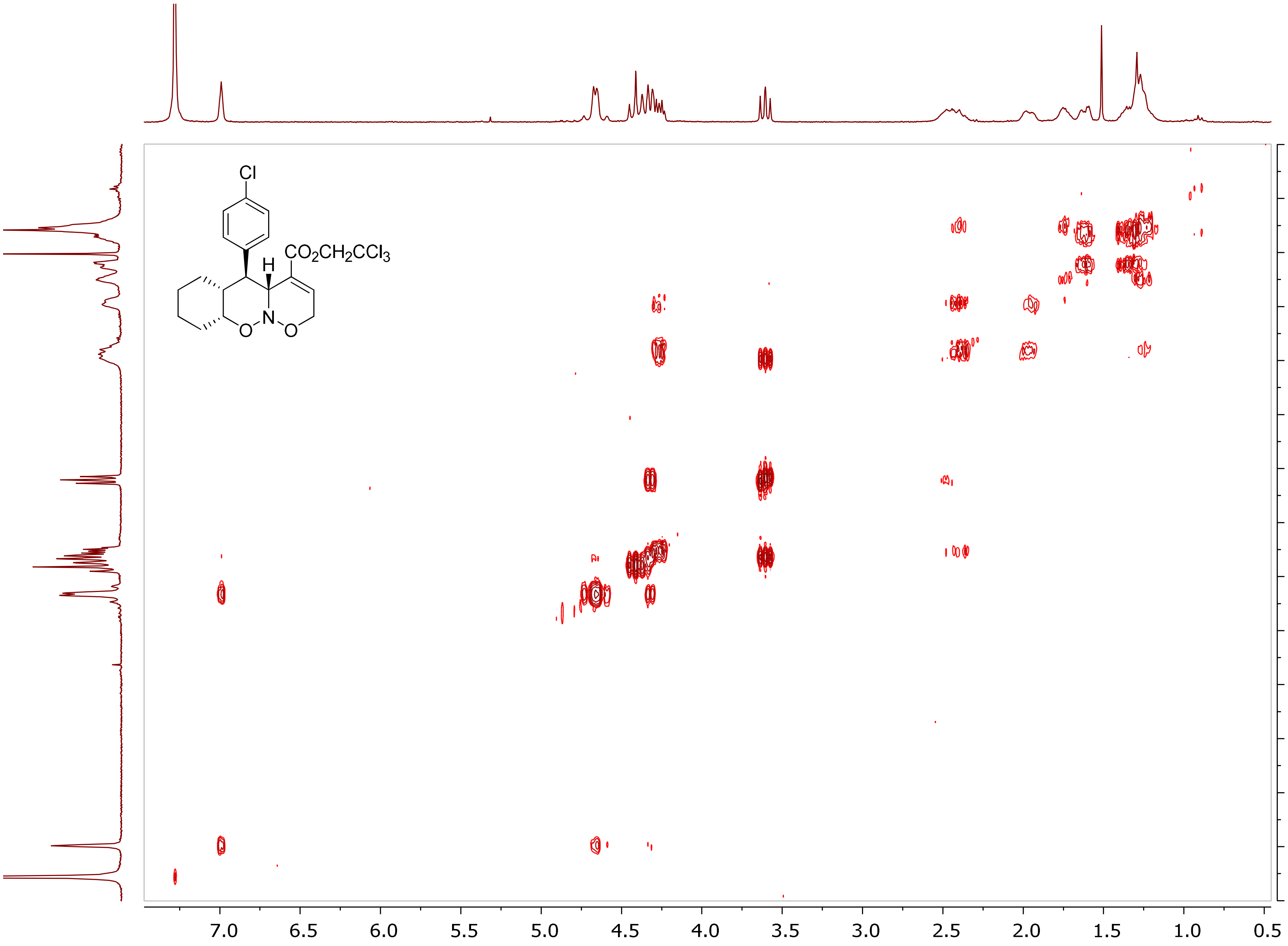

^1H-^{13}C HSQC

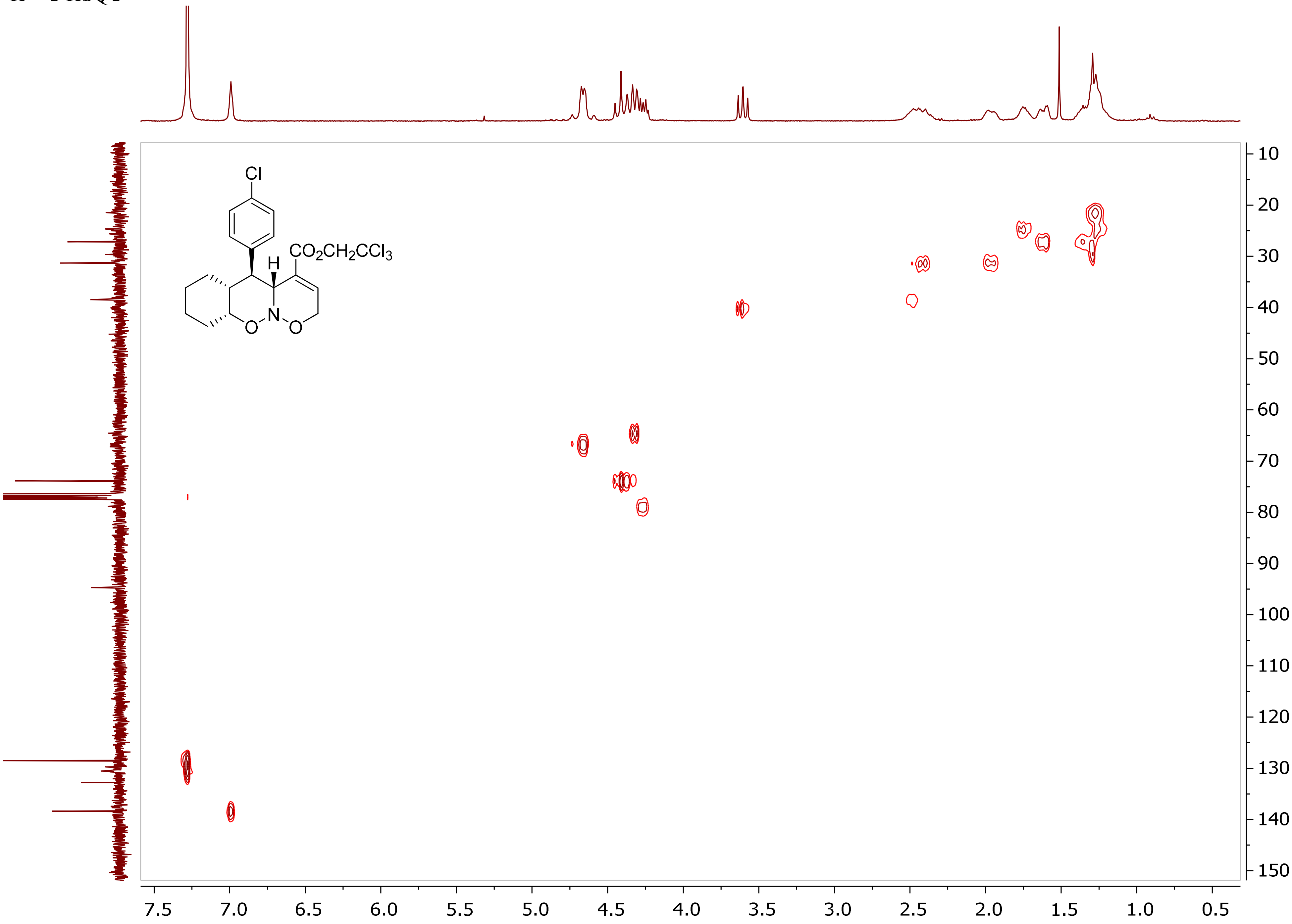
Cl
CO2CH2CCl3
H
N
O
O
7.5
7.0
6.5
6.0
5.5
5.0
4.5
4.0
3.5
3.0
2.5
2.0
1.5
1.0
0.5
10
20
30
40
50
60
70
80
90
100
110
120
130
140
150

2,2,2-Trichloroethyl 4-(4-methoxyphenyl)-2,2-dimethyl-3,4-dihydro-2H-pyrrolo[1,2-b][1,2]oxazine-5-carboxylate 4a

^{1}H NMR (300 MHz, $CDCl_3$)

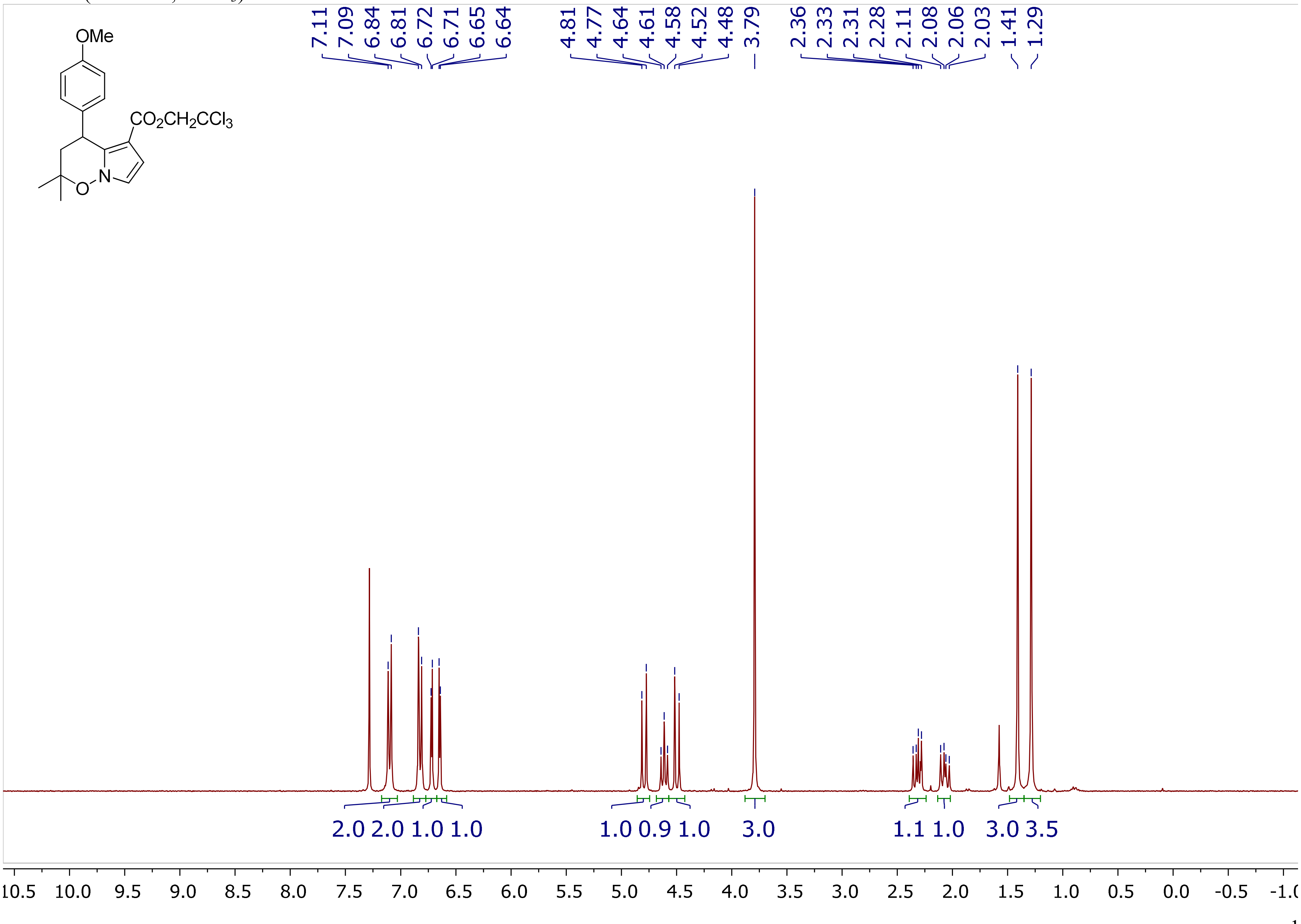

^{13}C NMR (75 MHz, $CDCl_3$)

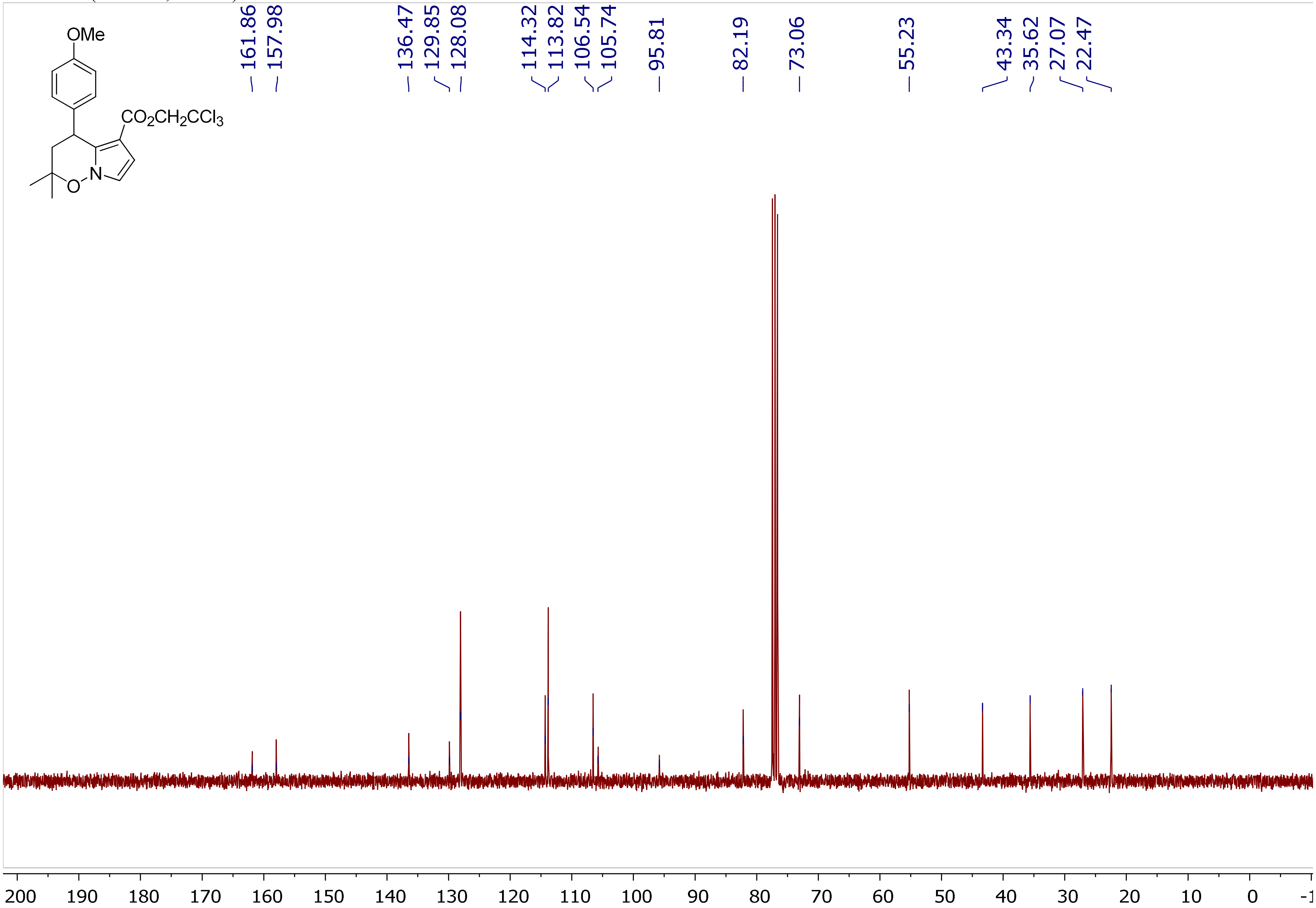

^{13}C DEPT 135 (75 MHz, $CDCl_3$)

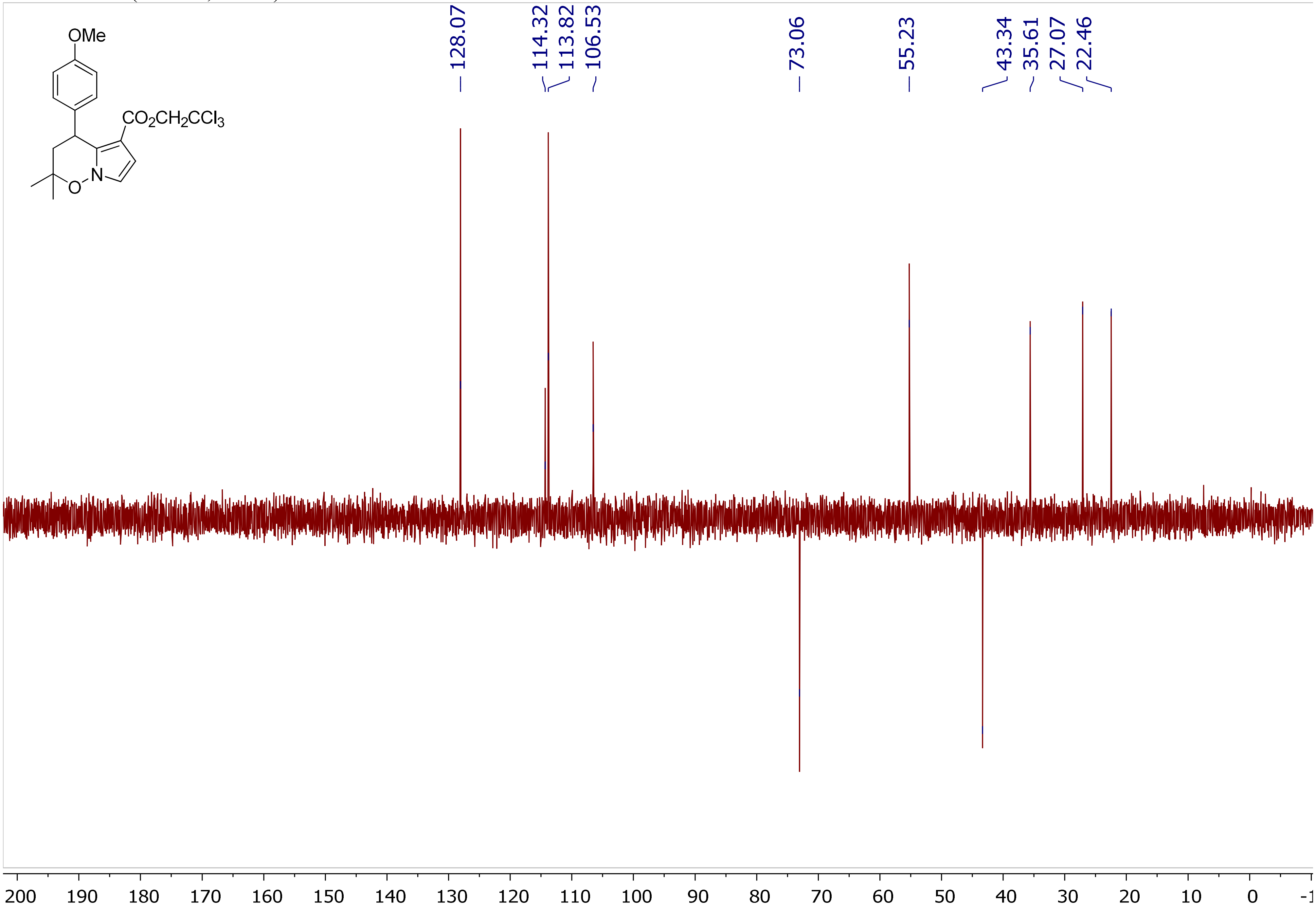

2,2,2-Trichloroethyl 4-(4-chloroxyphenyl)-2,2-dimethyl-3,4-dihydro-2H-pyrrolo[1,2-b][1,2]oxazine-5-carboxylate 4g

^{1}H NMR (300 MHz, $CDCl_3$)

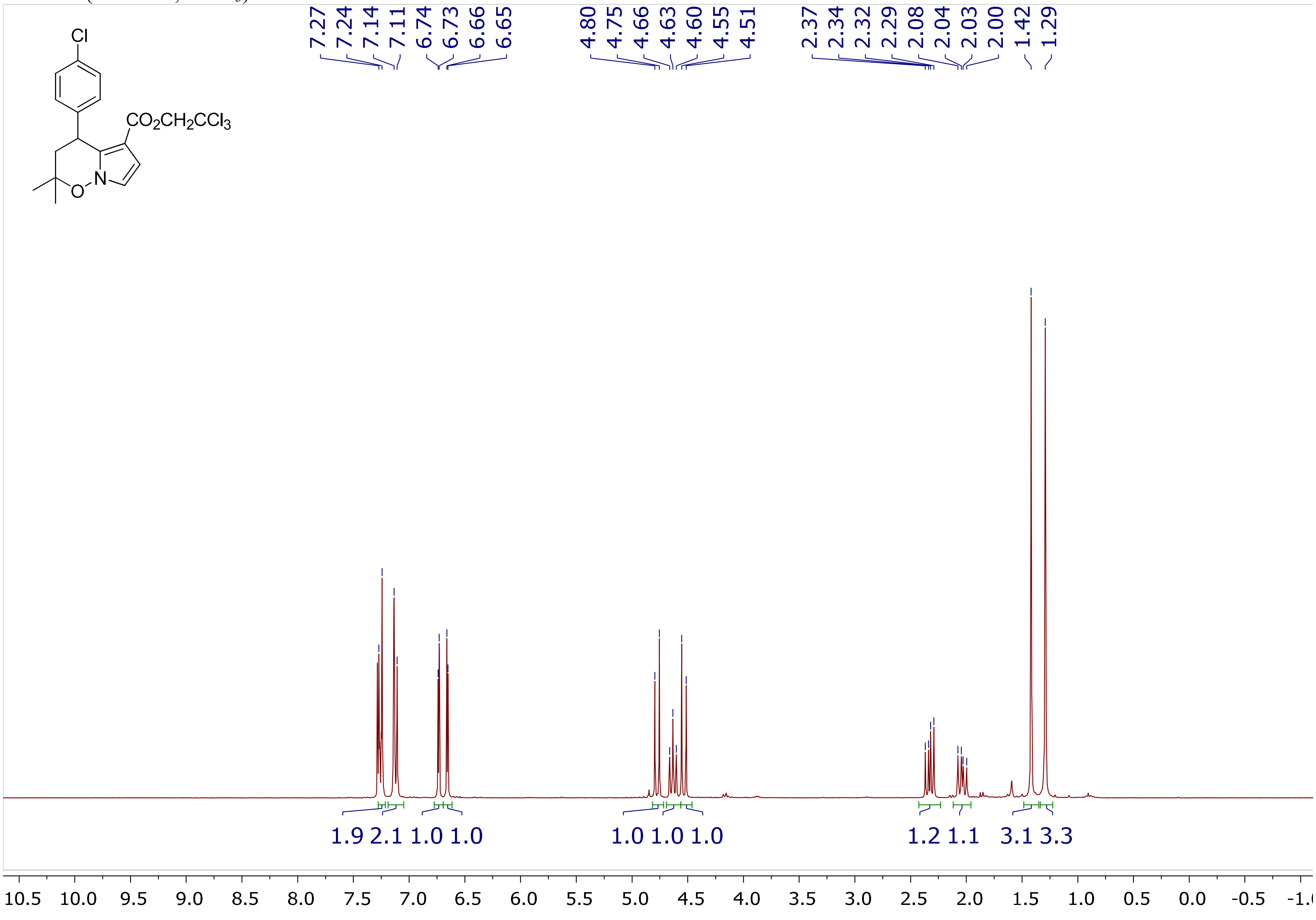

^{13}C NMR (75 MHz, $CDCl_3$)

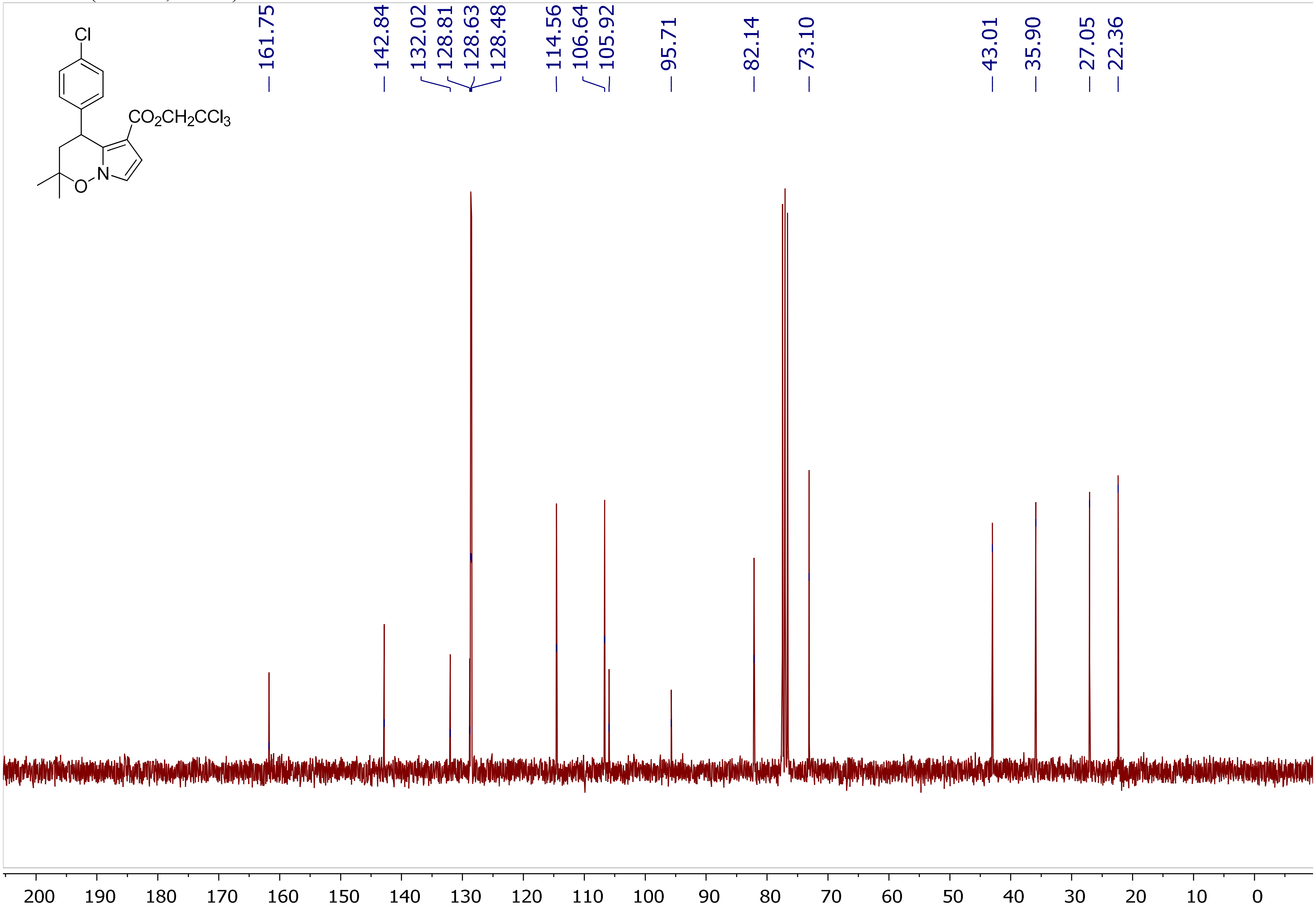

^{13}C DEPT 135 (75 MHz, $CDCl_3$)

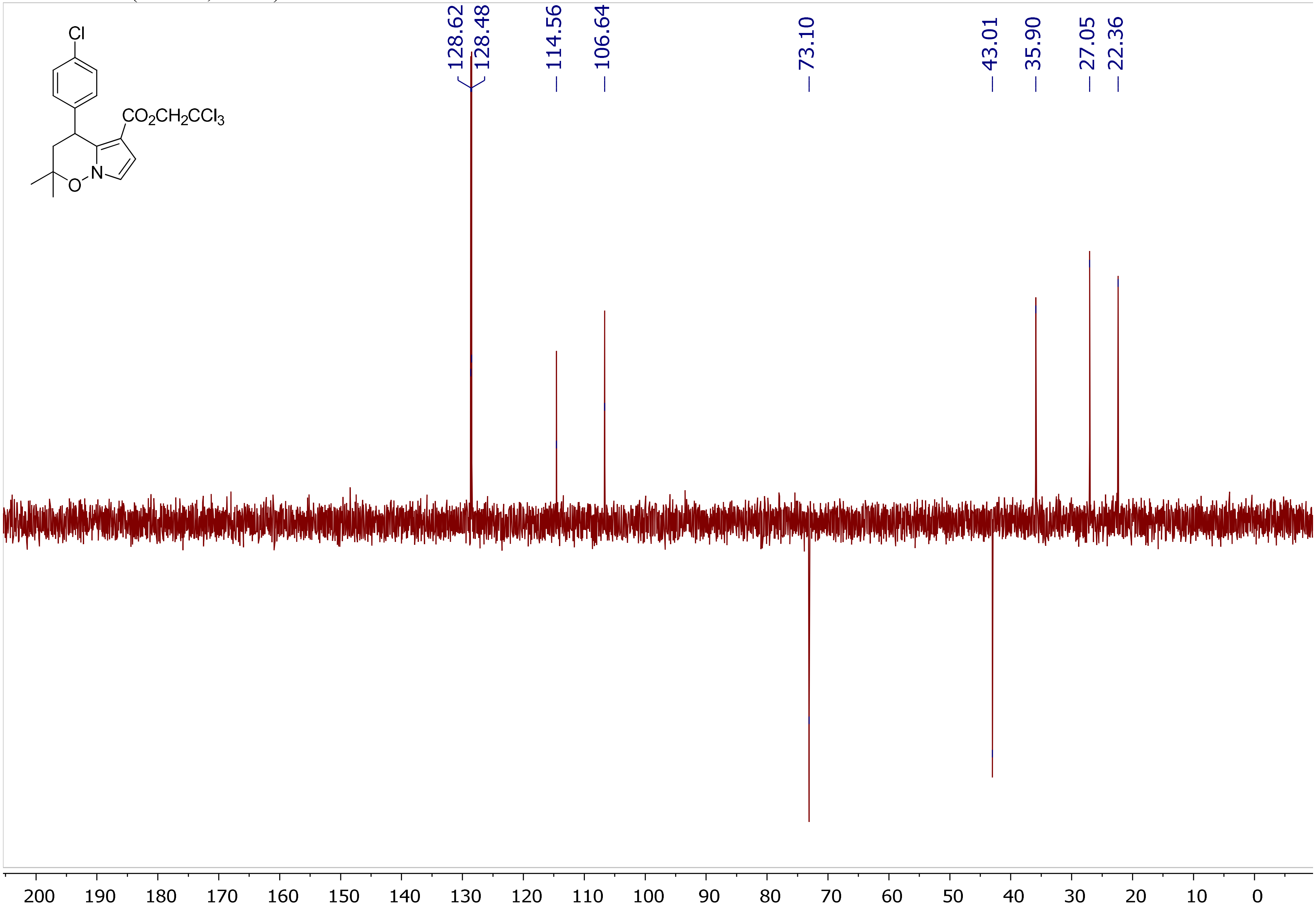

2,2,2-Trichloroethyl 4-(benzoyloxy)-2,2-dimethyl-3,4-dihydro-2H-pyrrolo[1,2-b][1,2]oxazine-5-carboxylate 4l

^1H NMR (300 MHz, $CDCl_3$)

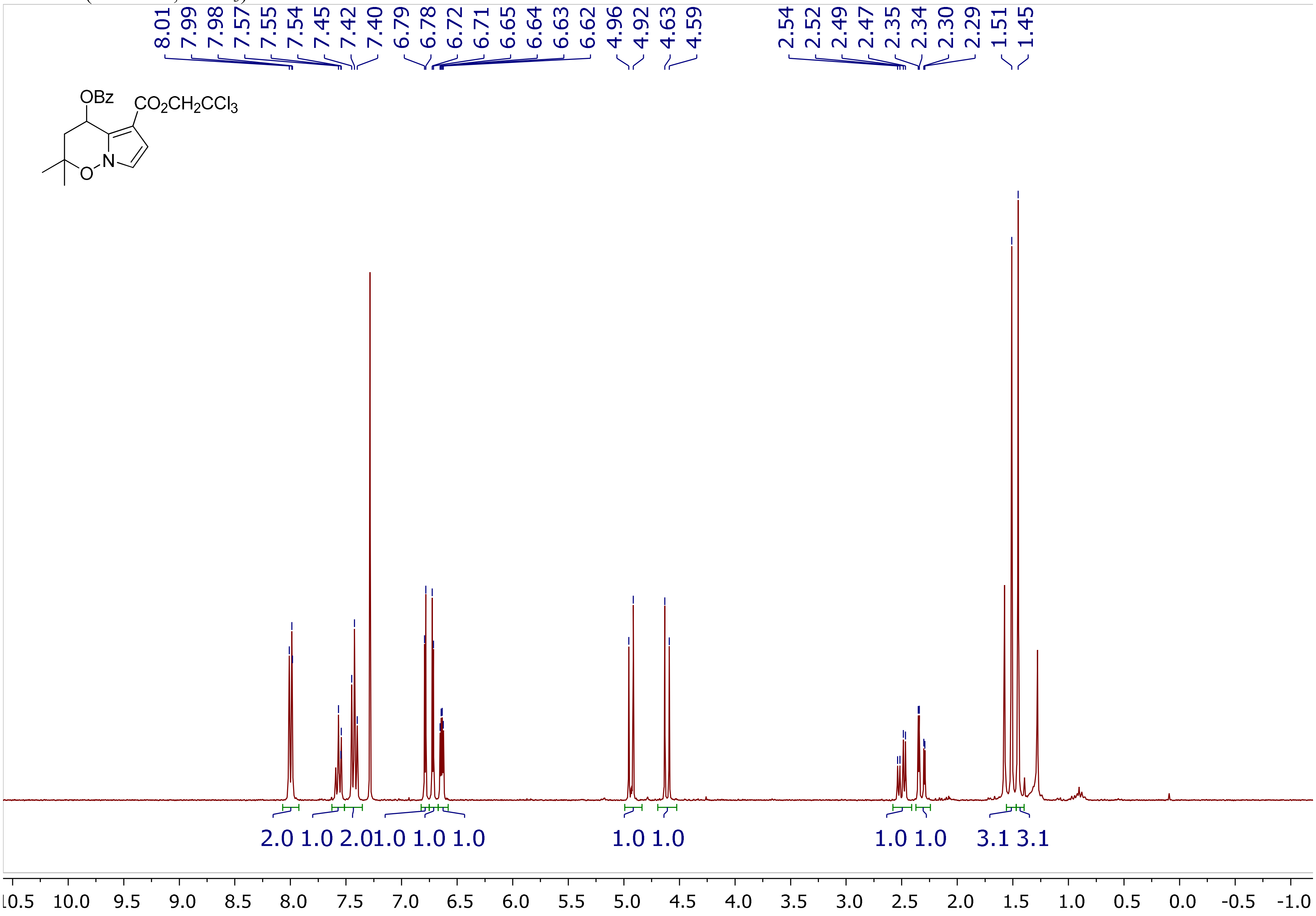

^{13}C NMR (75 MHz, $CDCl_3$)

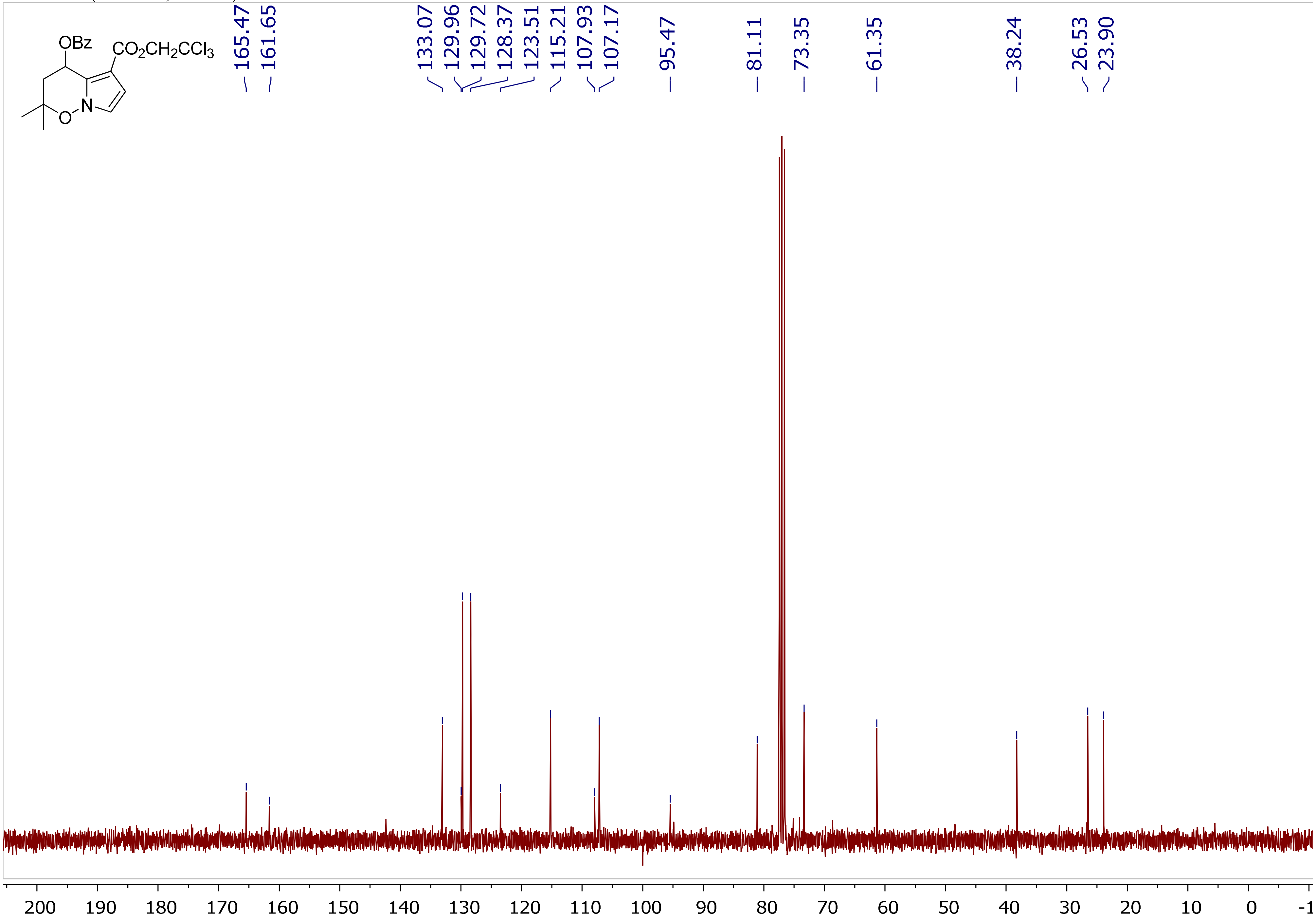

^{13}C DEPT 135 (75 MHz, $CDCl_3$)

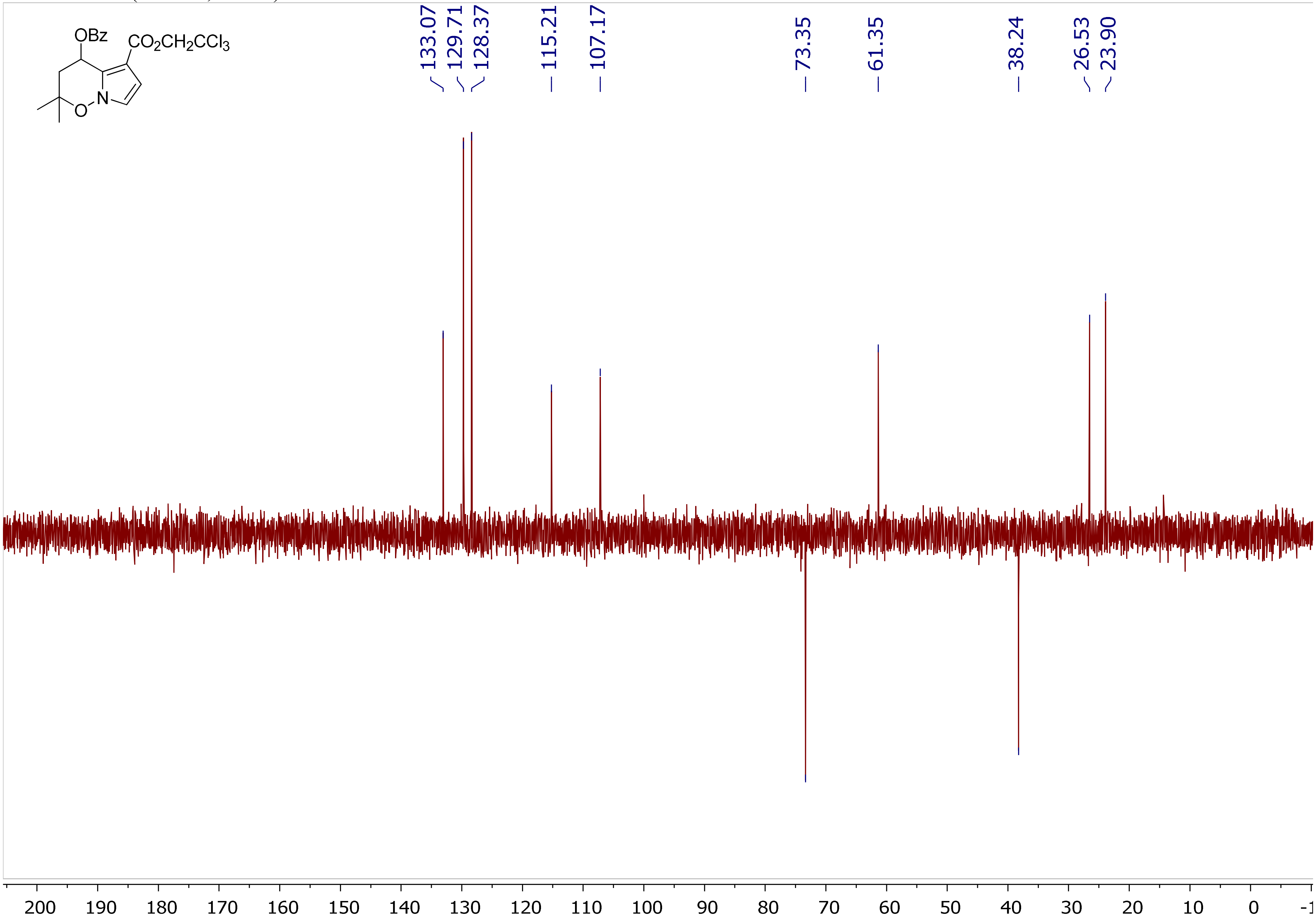

2,2,2-Trichloroethyl 2,2-dimethyl-4-phenethyl-3,4-dihydro-2H-pyrrolo[1,2-b][1,2]oxazine-5-carboxylate 4m

^{1}H NMR (300 MHz, $CDCl_3$)

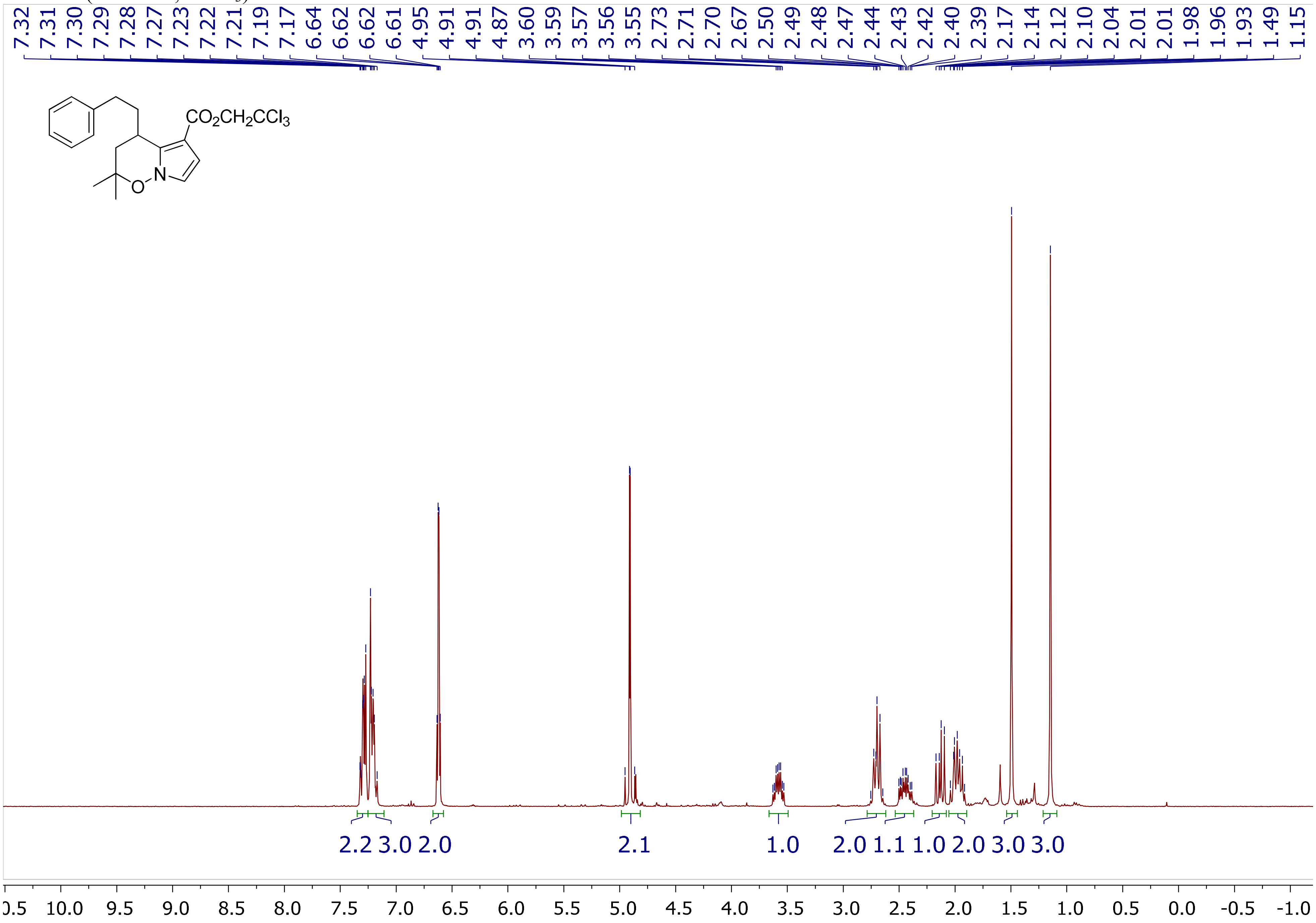

^{13}C NMR (75 MHz, $CDCl_3$)

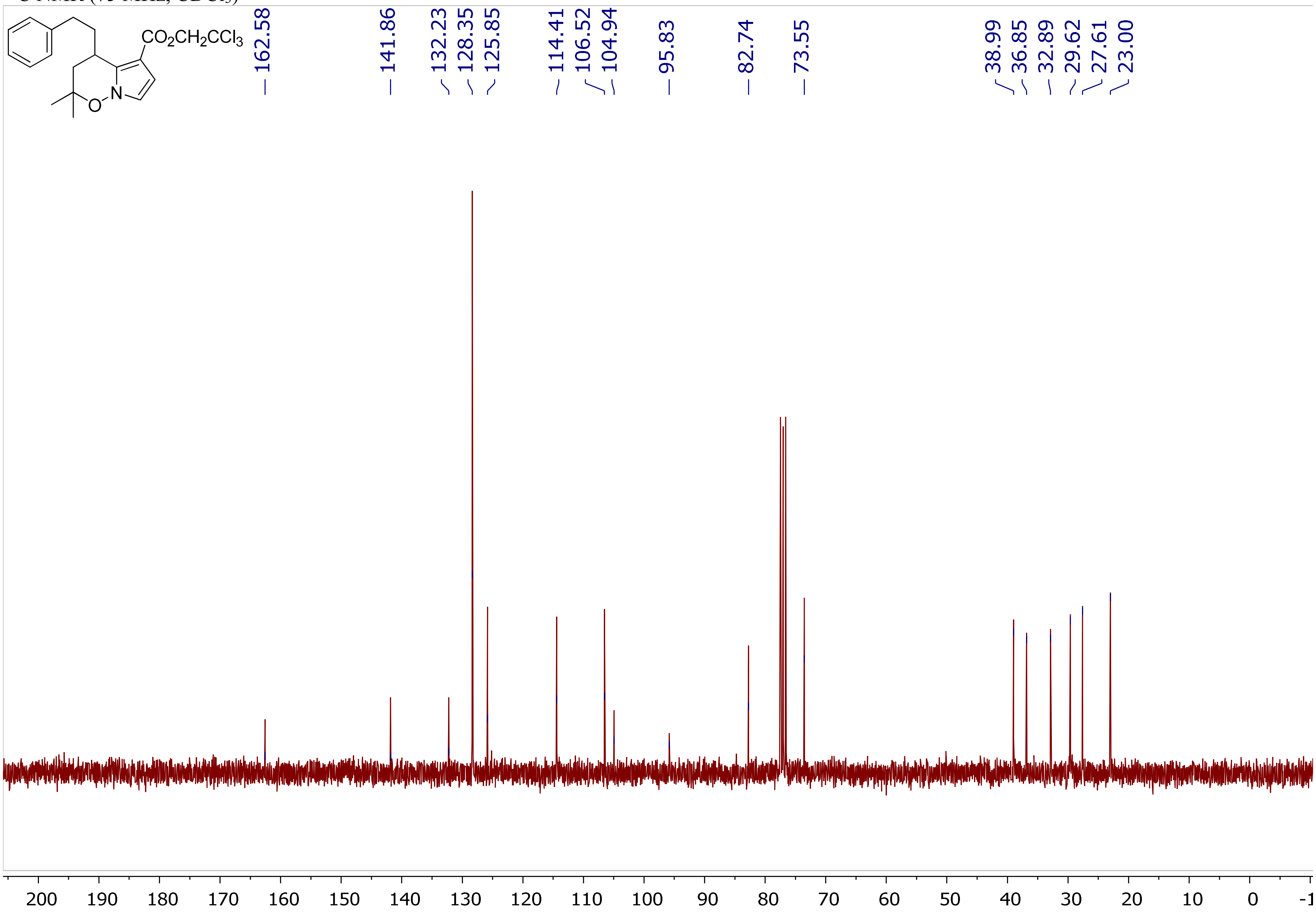

^{13}C DEPT 135 (75 MHz, $CDCl_3$)

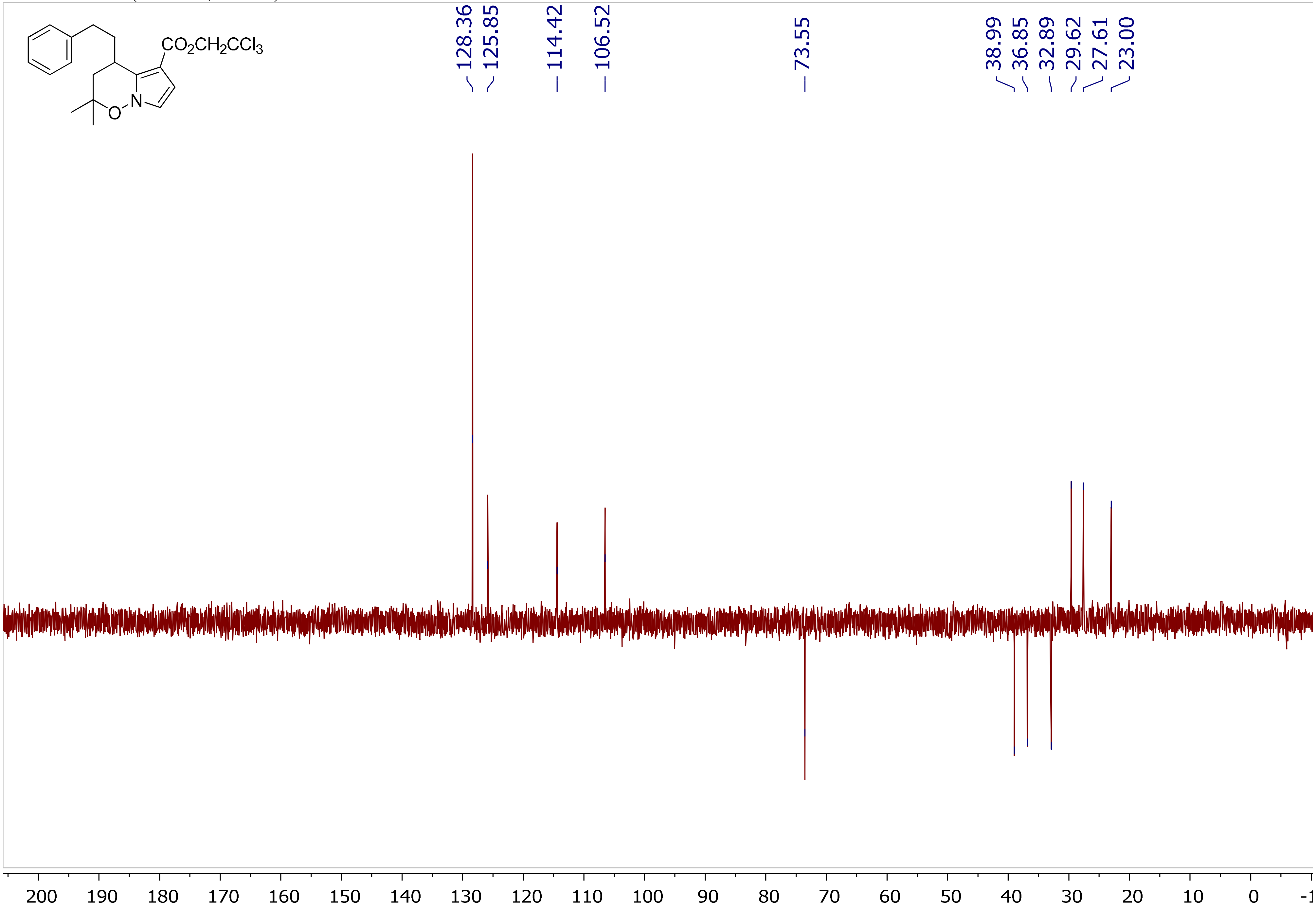

^{1}H-^{1}H COSY

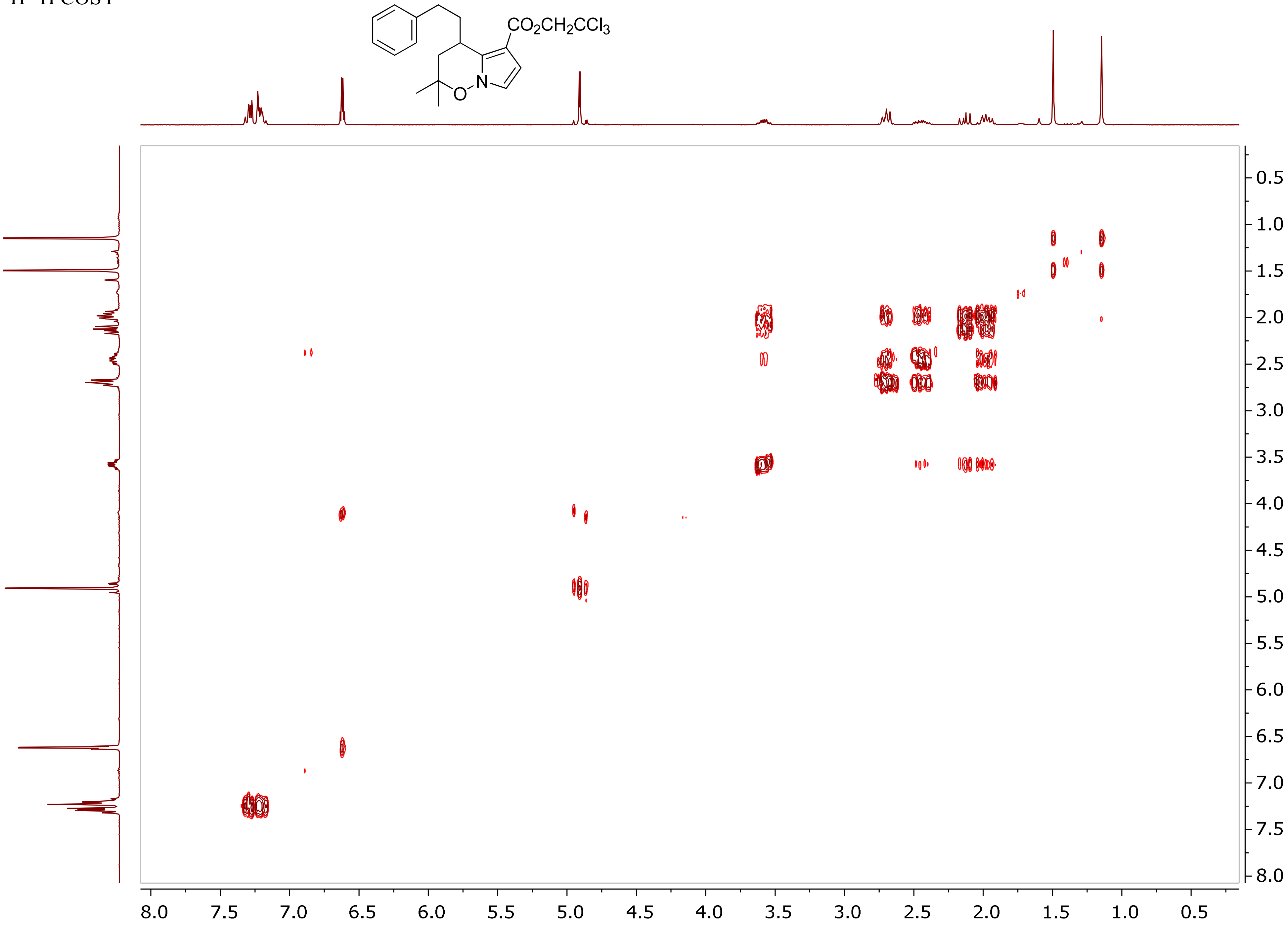

^{1}H-^{13}C HSQC

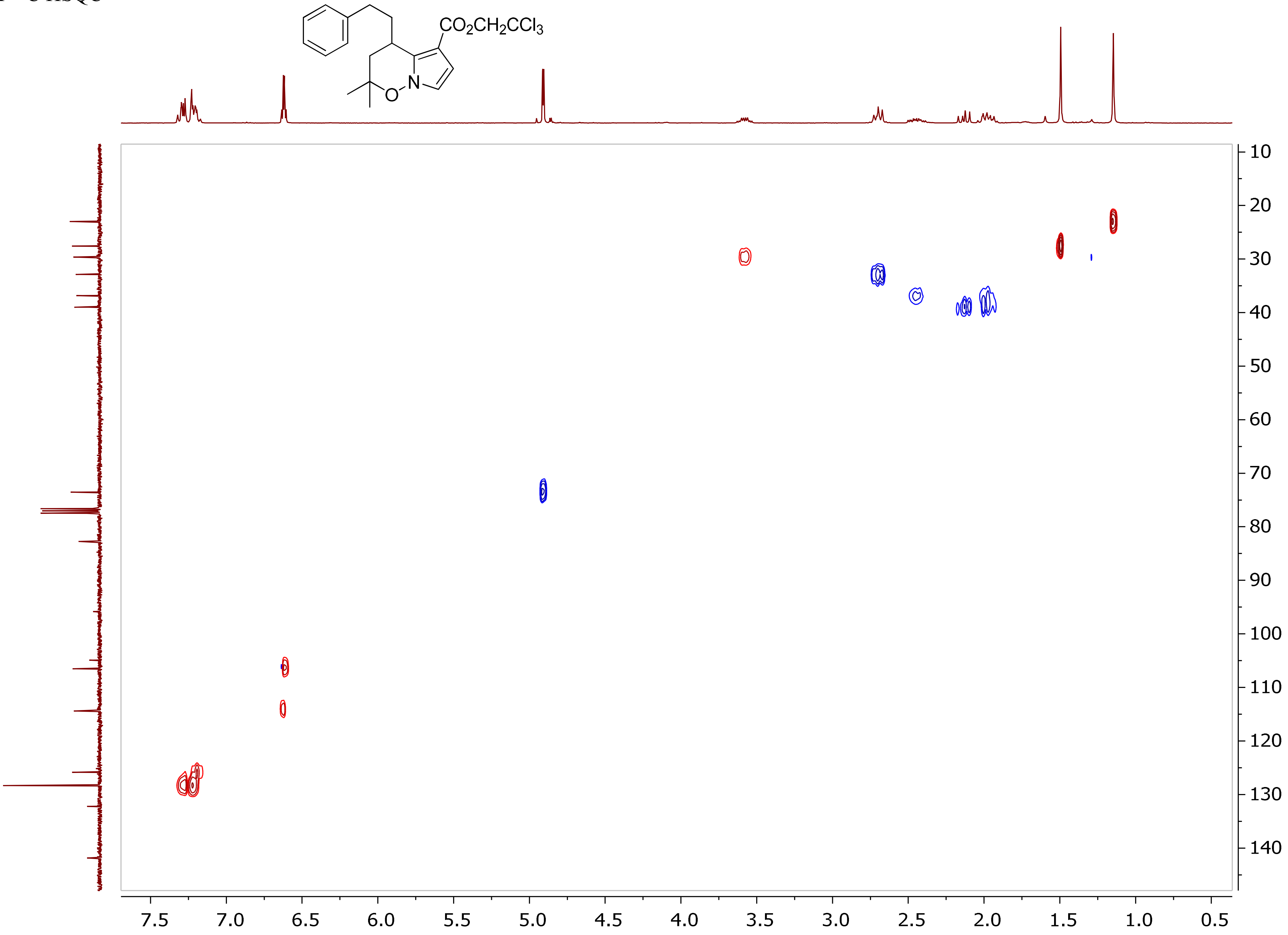

CO2CH2CCl3
O
N
7.5
7.0
6.5
6.0
5.5
5.0
4.5
4.0
3.5
3.0
2.5
2.0
1.5
1.0
0.5
10
20
30
40
50
60
70
80
90
100
110
120
130
140

2,2,2-Trichloroethyl (2R*,4S*)-2,4-diphenyl-3,4-dihydro-2H-pyrrolo[1,2-b][1,2]oxazine-5-carboxylate 4n

^{1}H NMR (300 MHz, $CDCl_3$)

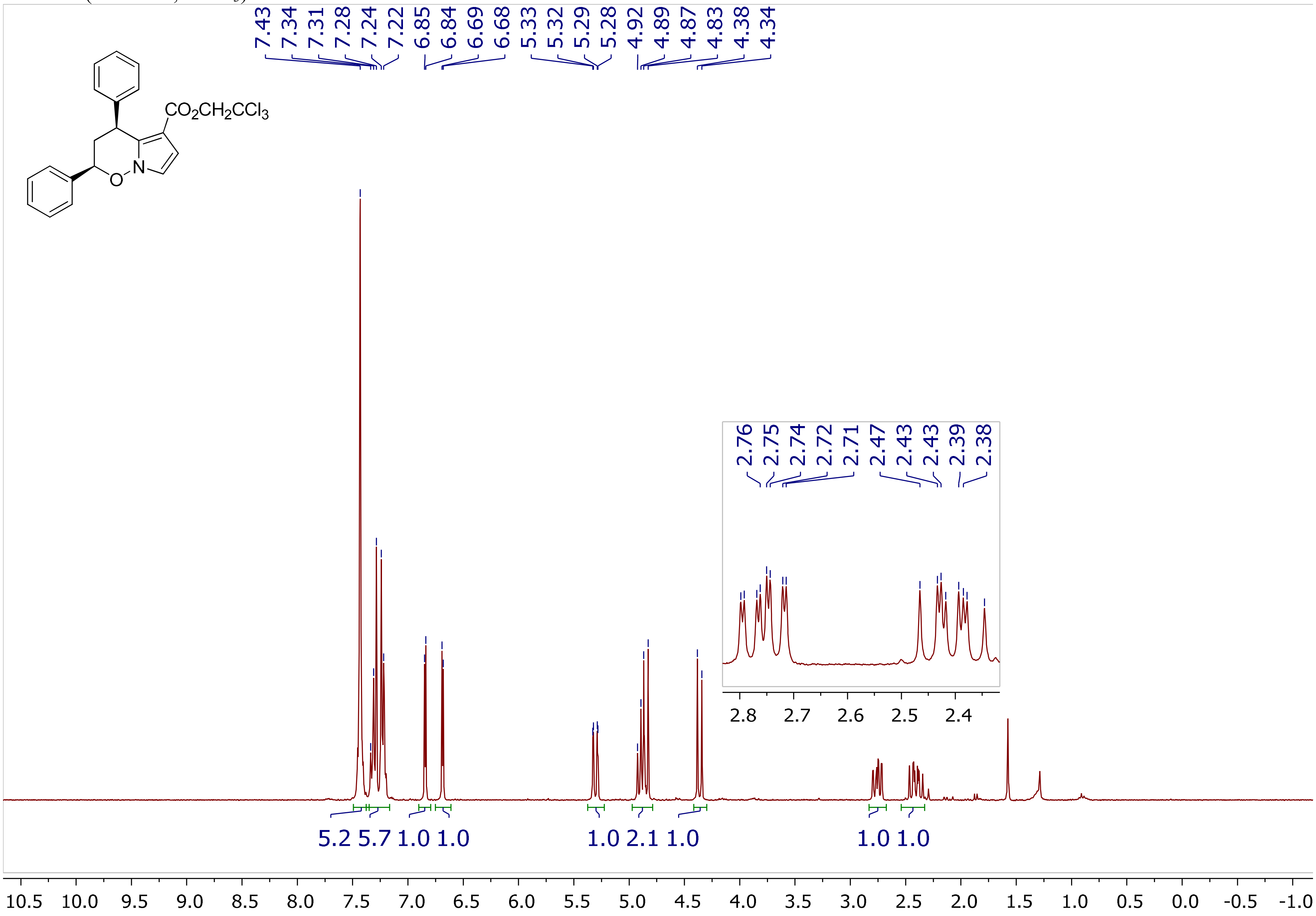

^{13}C NMR (75 MHz, $CDCl_3$)

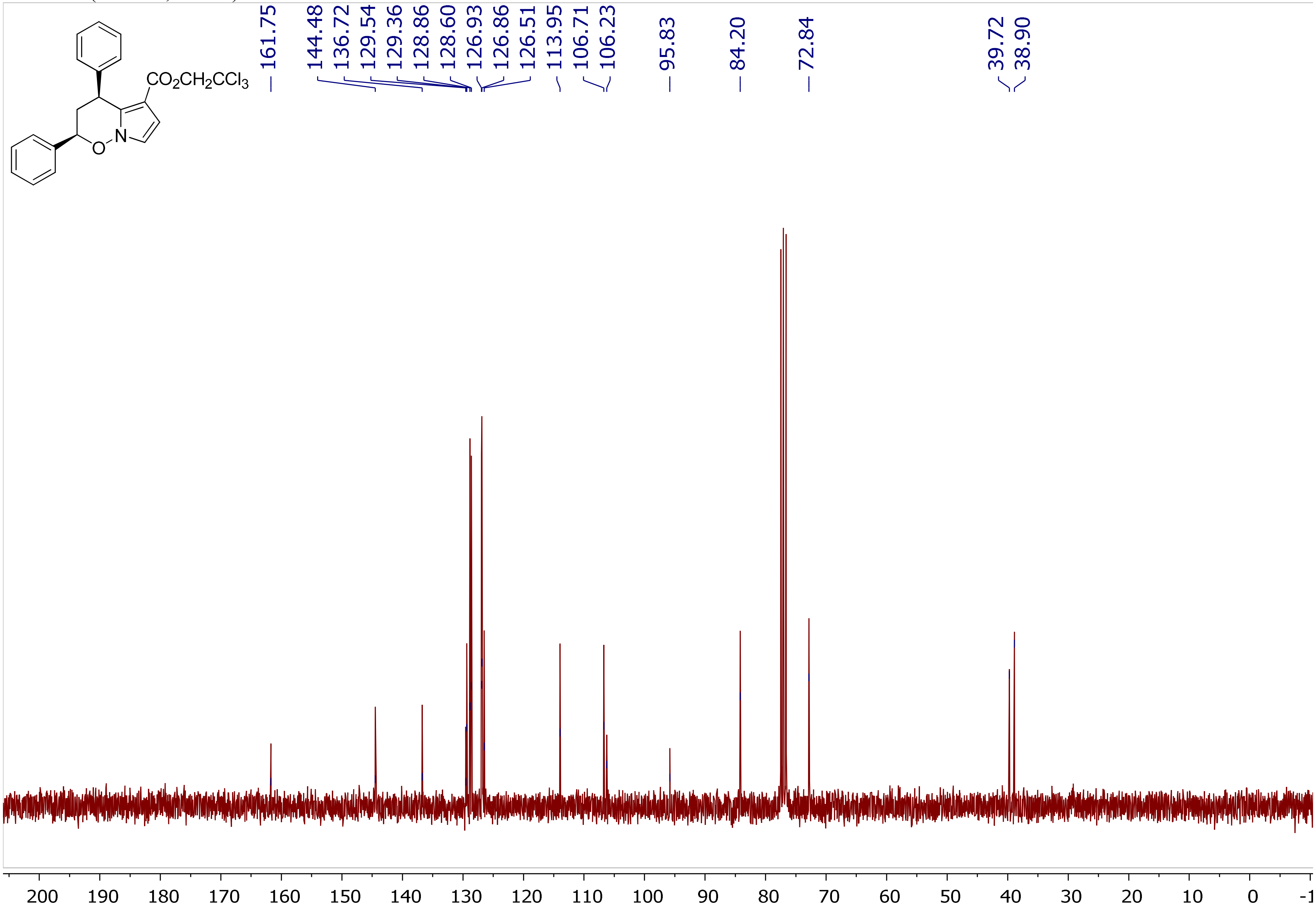

^{13}C DEPT 135 (75 MHz, $CDCl_3$)

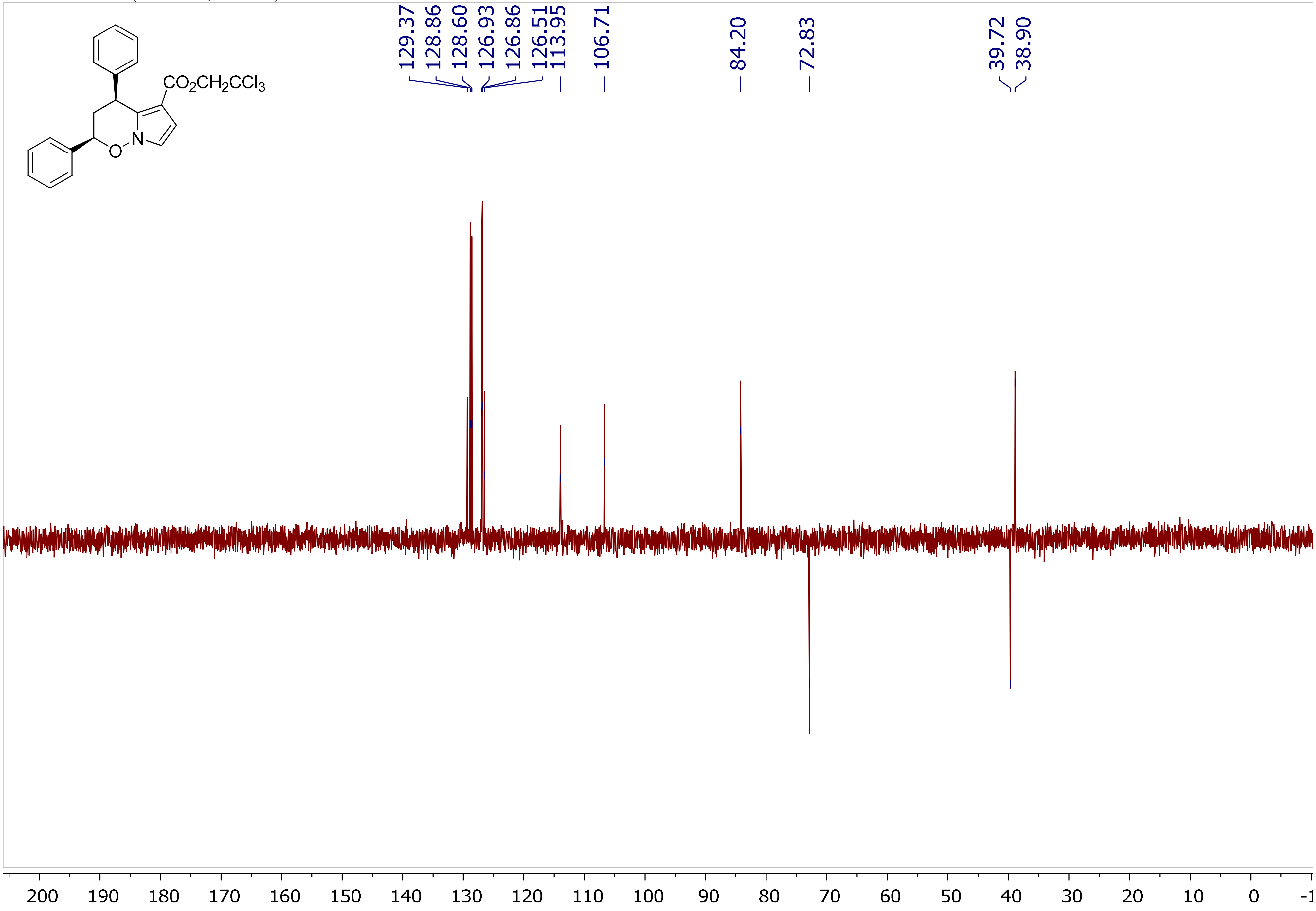

2,2,2-Trichloroethyl (2S*,4S*)-2-ethoxy-4-(4-methoxyphenyl)-3,4-dihydro-2H-pyrrolo[1,2-b][1,2]oxazine-5-carboxylate 4o

^{1}H NMR (300 MHz, $CDCl_3$)

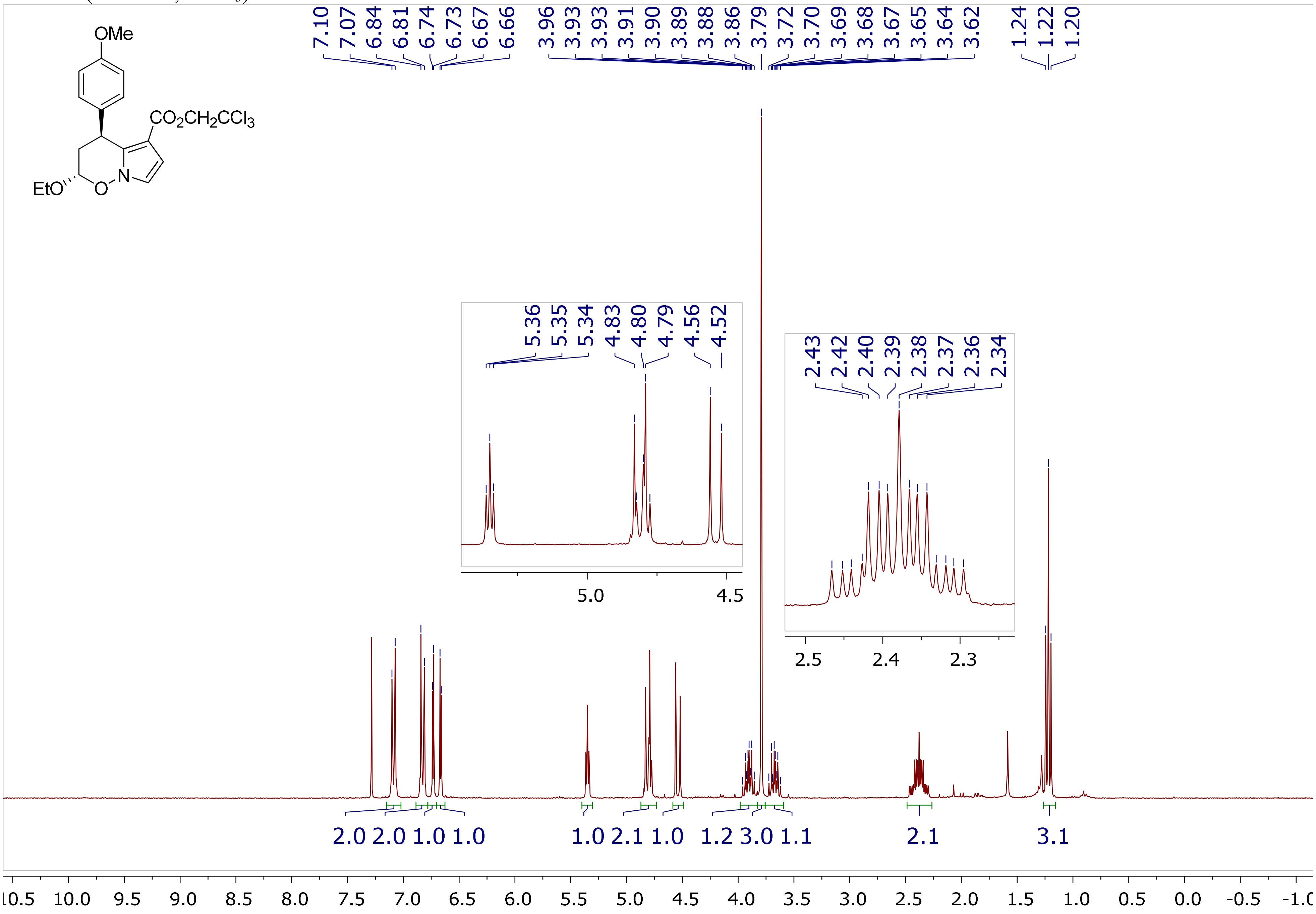

^{13}C NMR (75 MHz, $CDCl_3$)

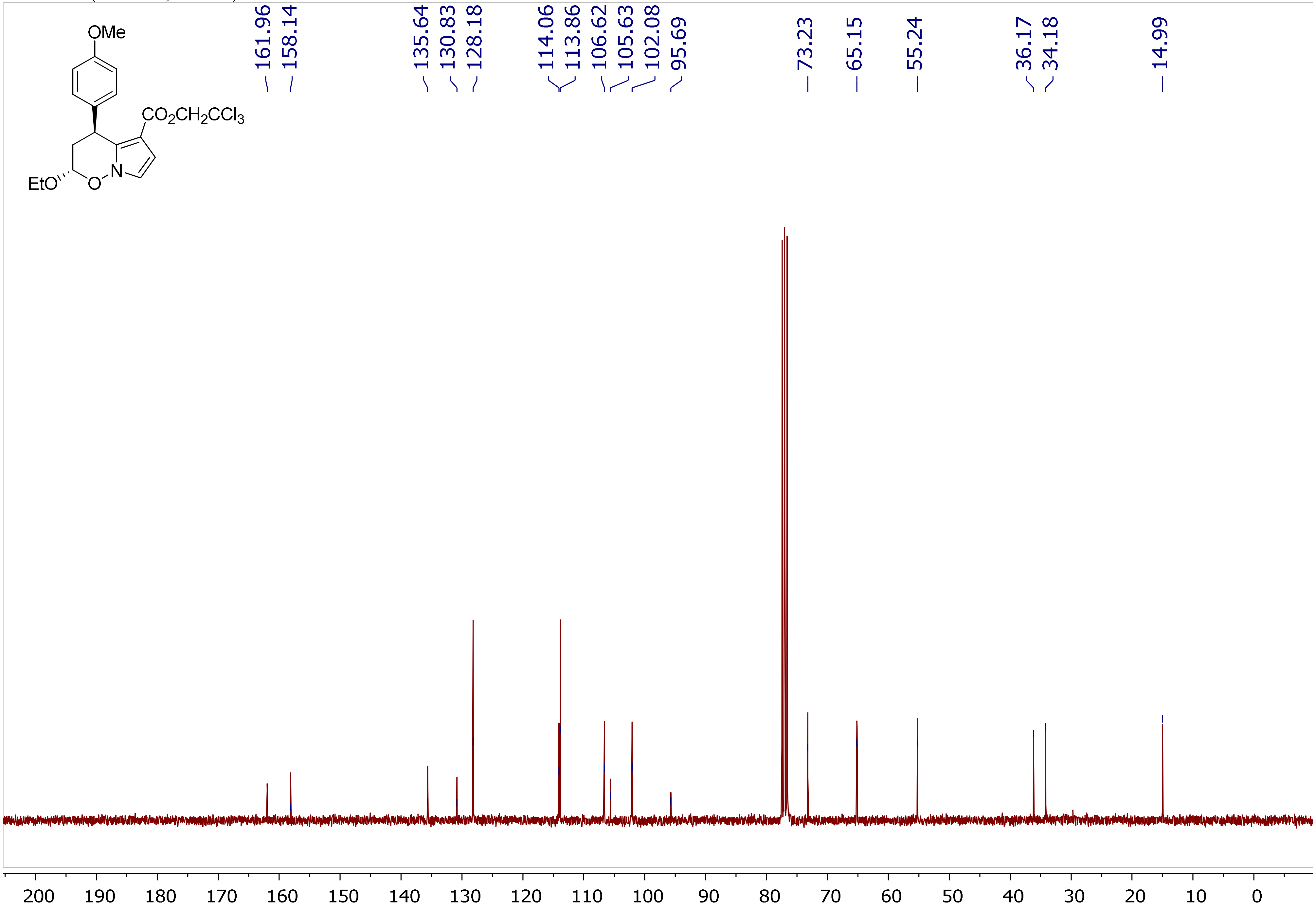

^{13}C DEPT 135 (75 MHz, $CDCl_3$)

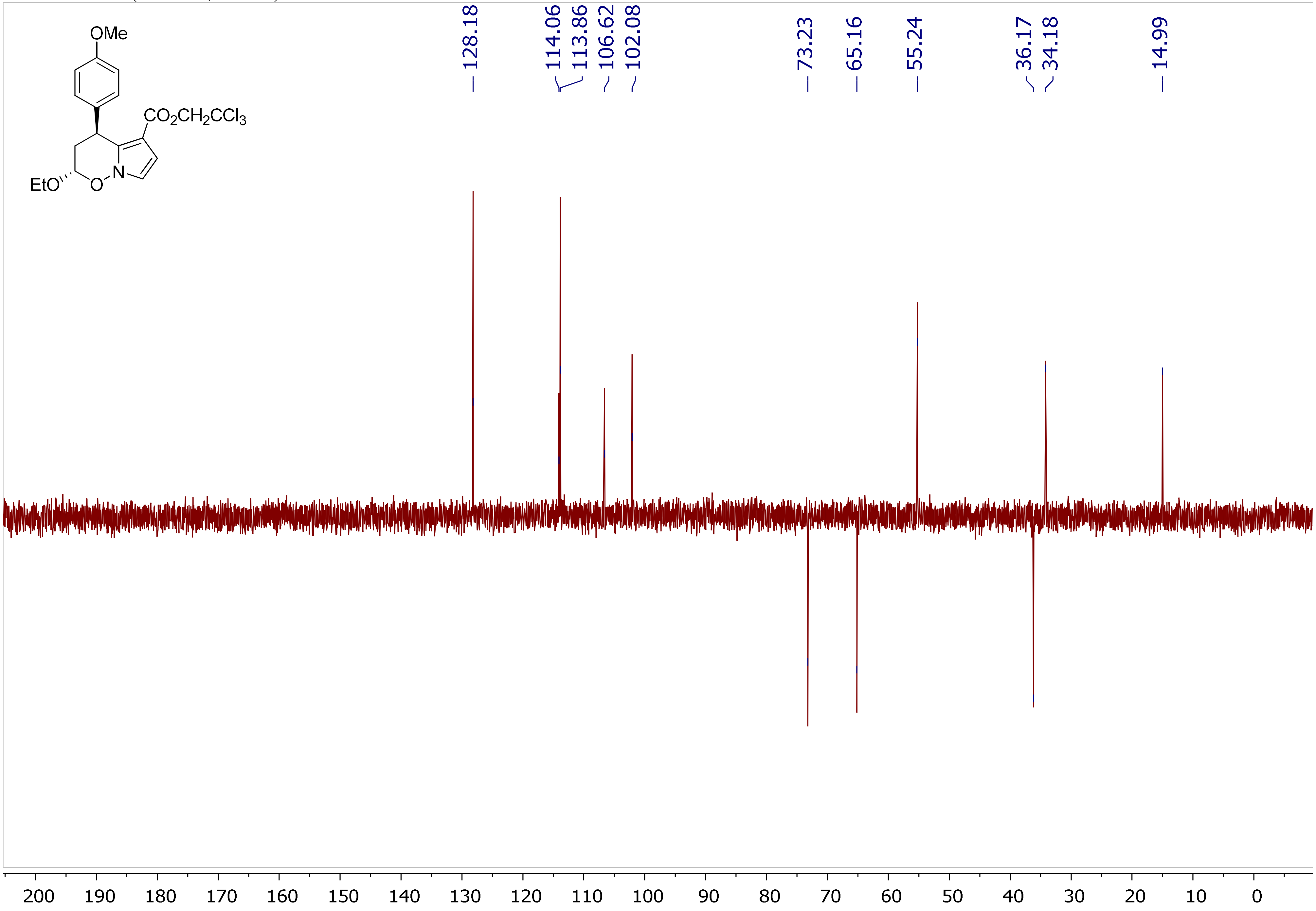

Methyl 4-(4-methoxyphenyl)-2,2-dimethyl-3,4-dihydro-2H-pyrrolo[1,2-b][1,2]oxazine-5-carboxylate 4b

^{1}H NMR (300 MHz, $CDCl_3$)

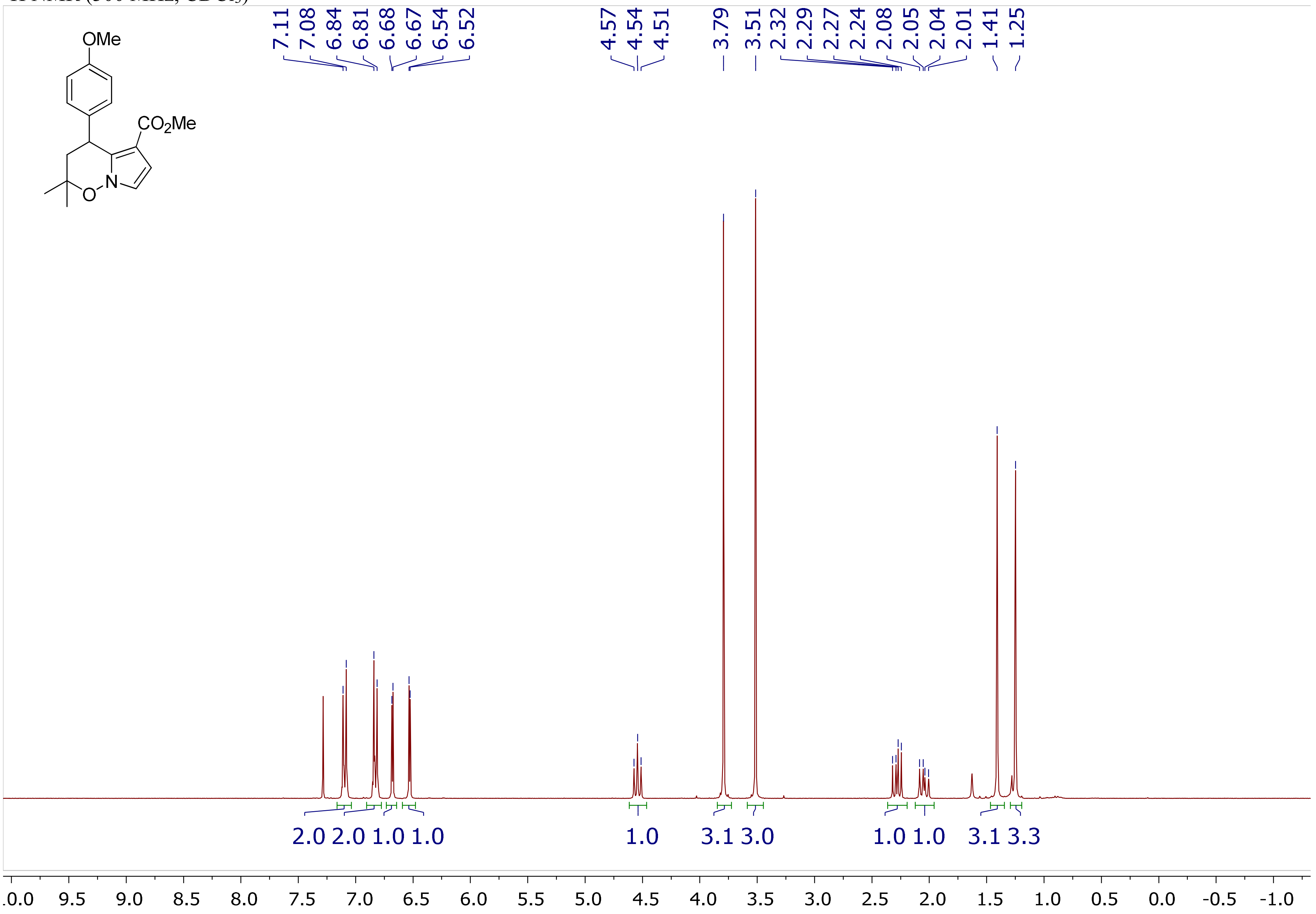

^{13}C NMR (75 MHz, $CDCl_3$)

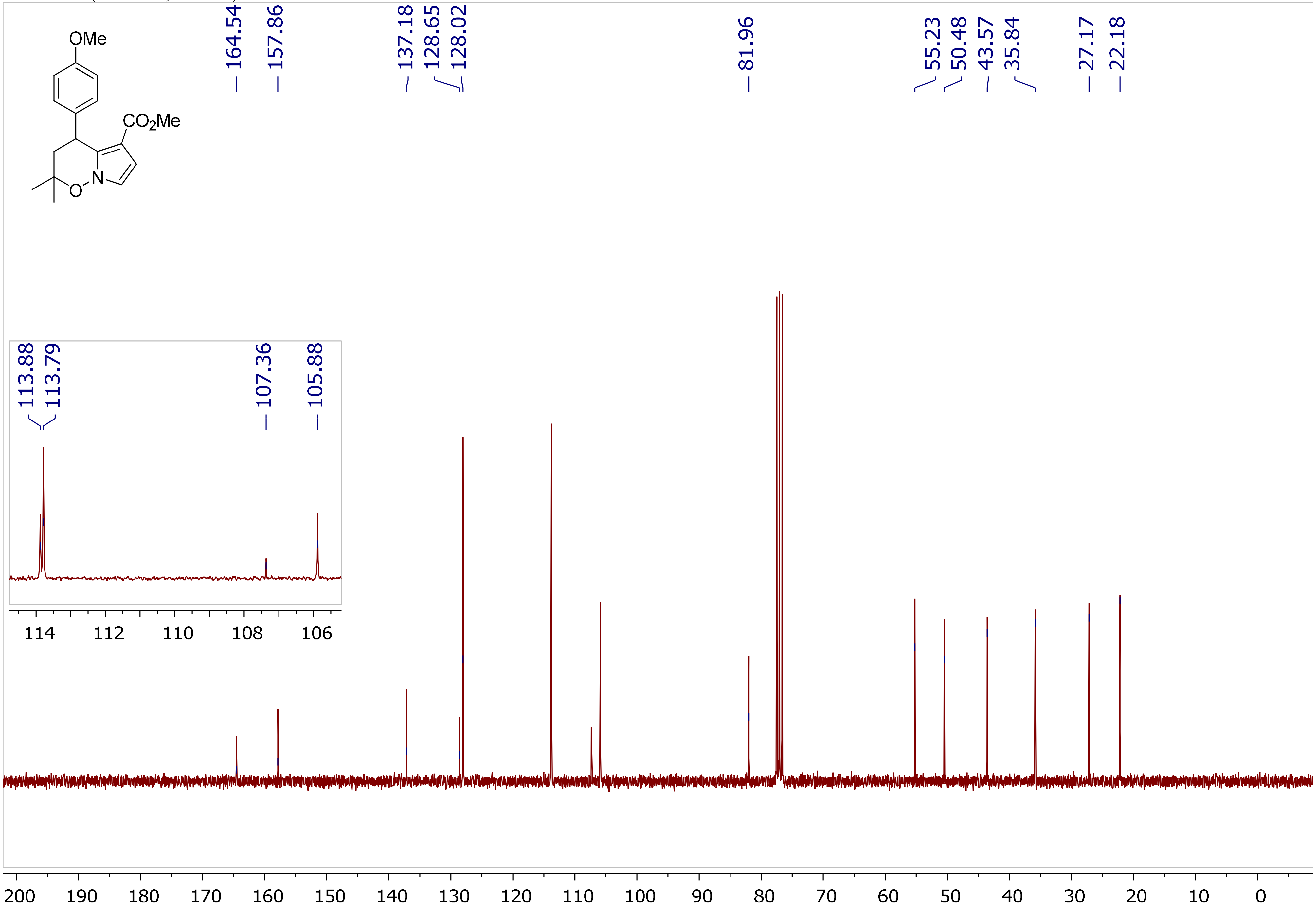

^{13}C DEPT 135 (75 MHz, $CDCl_3$)

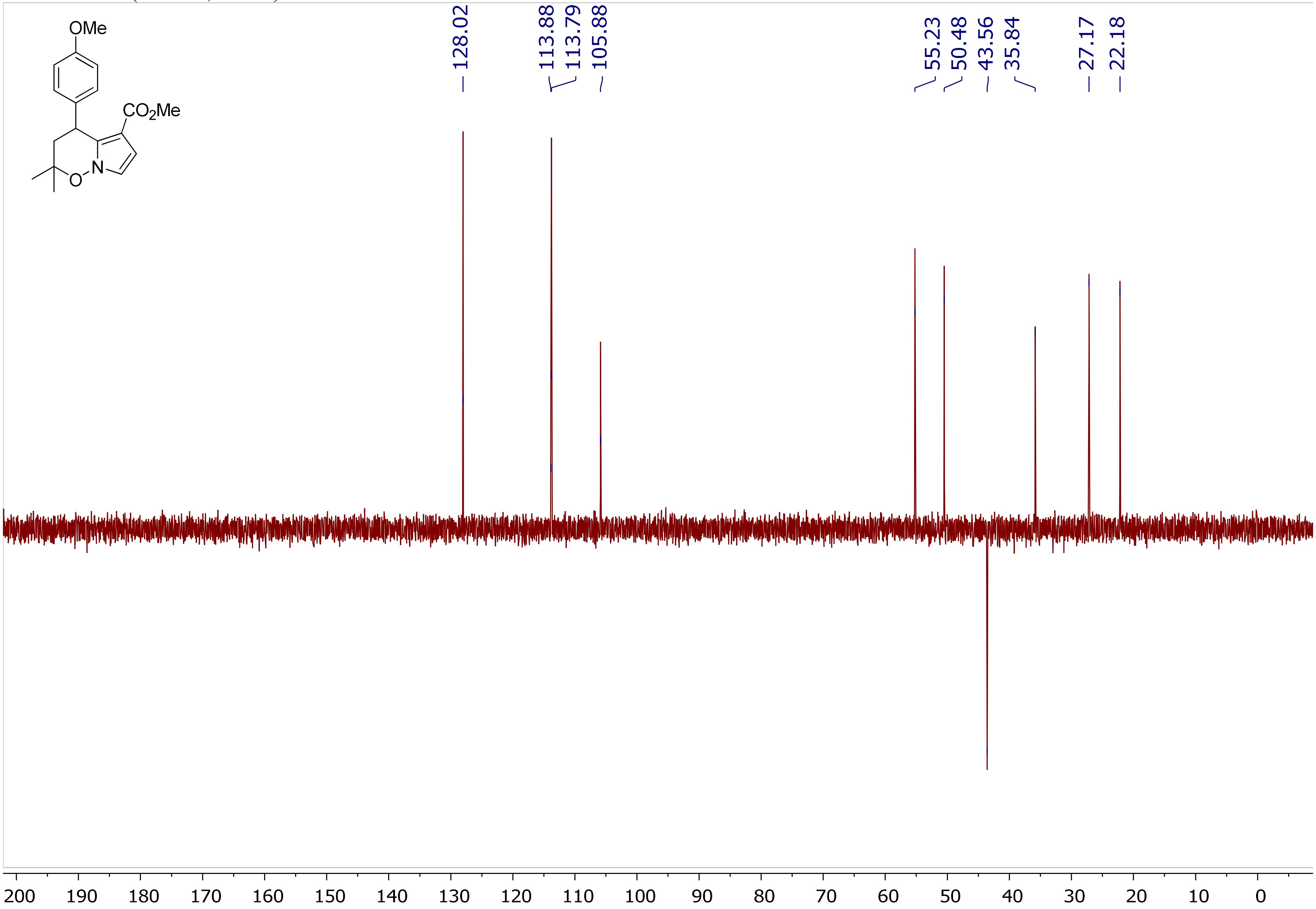

^{1}H-^{1}H COSY

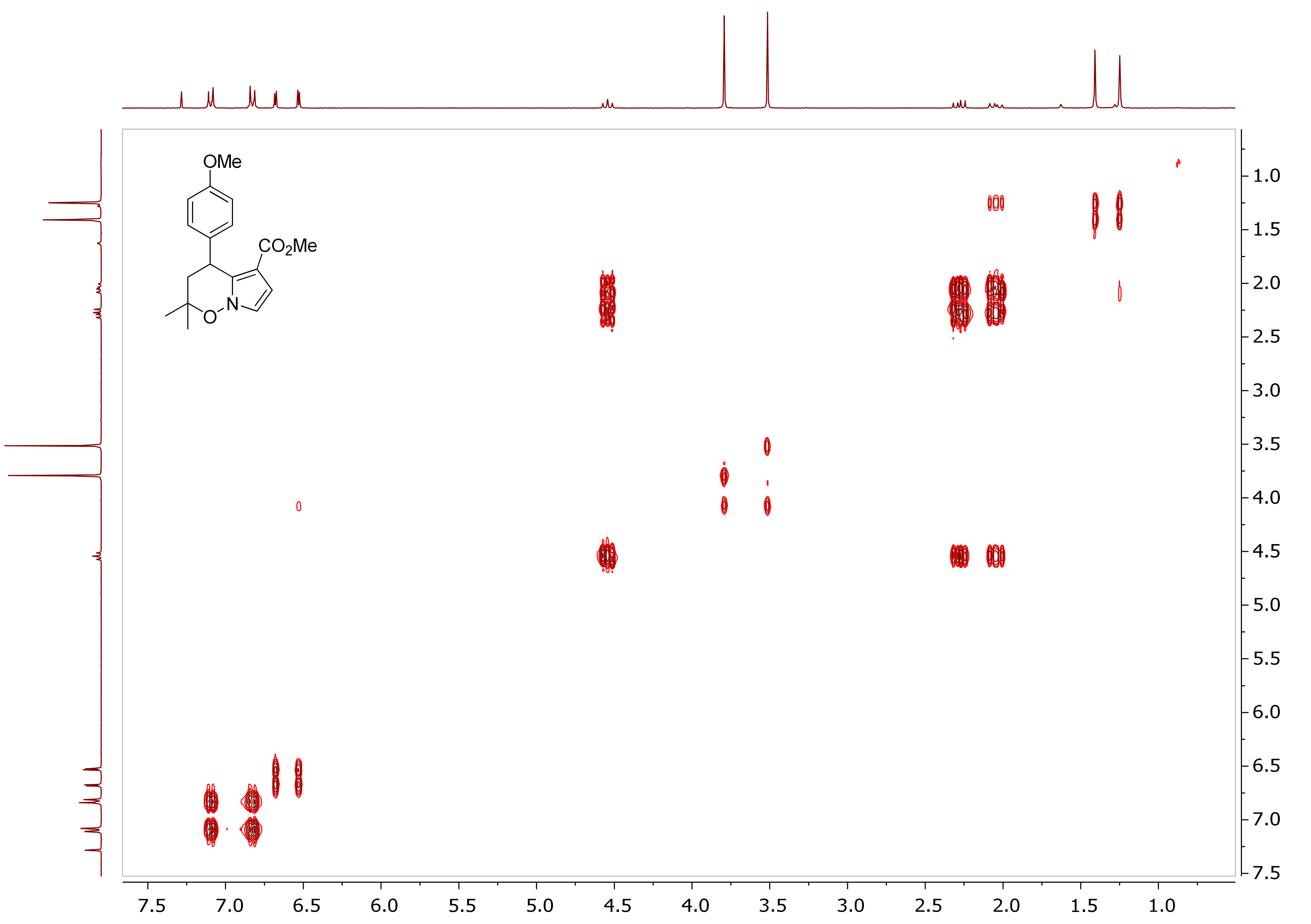
OMe
CO2Me
N
O
1.0
1.5
2.0
2.5
3.0
3.5
4.0
4.5
5.0
5.5
6.0
6.5
7.0
7.5
7.5
7.0
6.5
6.0
5.5
5.0
4.5
4.0
3.5
3.0
2.5
2.0
1.5
1.0

^{1}H-^{13}C HSQC

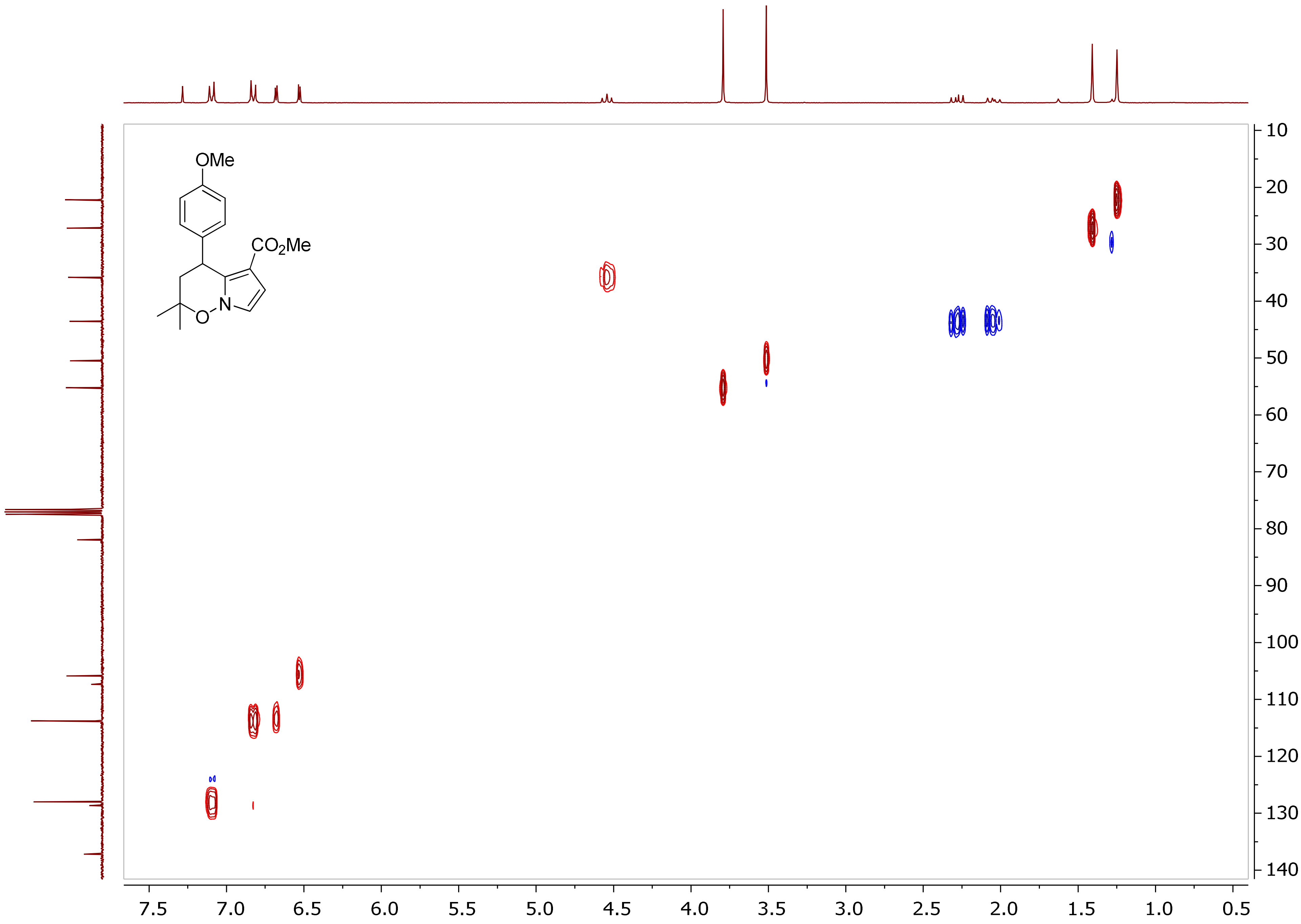
OMe
CO_2Me
N
O
10
20
30
40
50
60
70
80
90
100
110
120
130
140
7.5
7.0
6.5
6.0
5.5
5.0
4.5
4.0
3.5
3.0
2.5
2.0
1.5
1.0
0.5

^{1}H-^{13}C HMBC

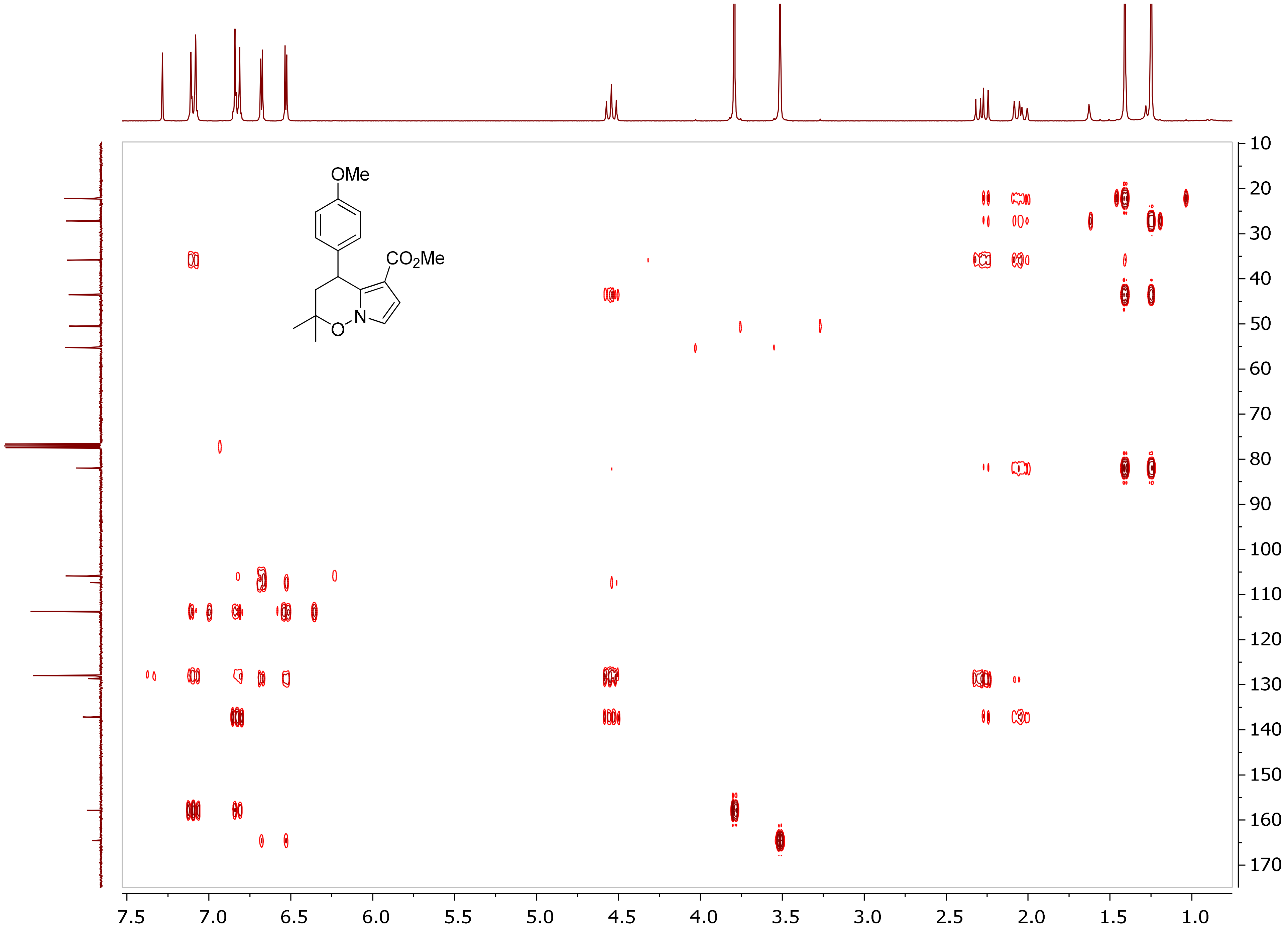
OMe
CO_2Me
N
O
7.5
7.0
6.5
6.0
5.5
5.0
4.5
4.0
3.5
3.0
2.5
2.0
1.5
1.0
10
20
30
40
50
60
70
80
90
100
110
120
130
140
150
160
170

4-(4-Methoxyphenyl)-2,2-dimethyl-3,4-dihydro-2H-pyrrolo[1,2-b][1,2]oxazine-5-carboxylic acid 5

^{1}H NMR (300 MHz, $CDCl_3$)

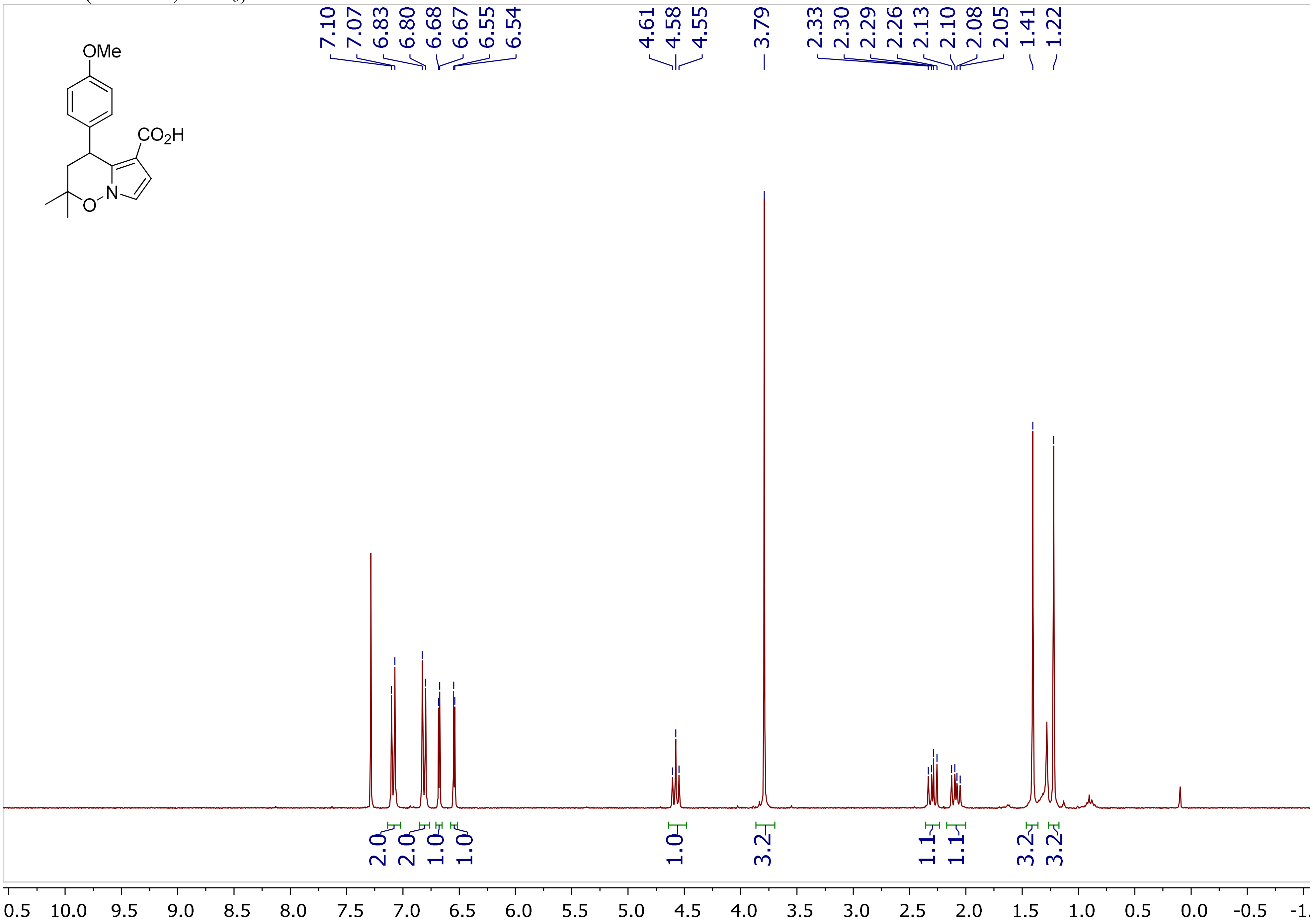

^{13}C NMR (75 MHz, $CDCl_3$)

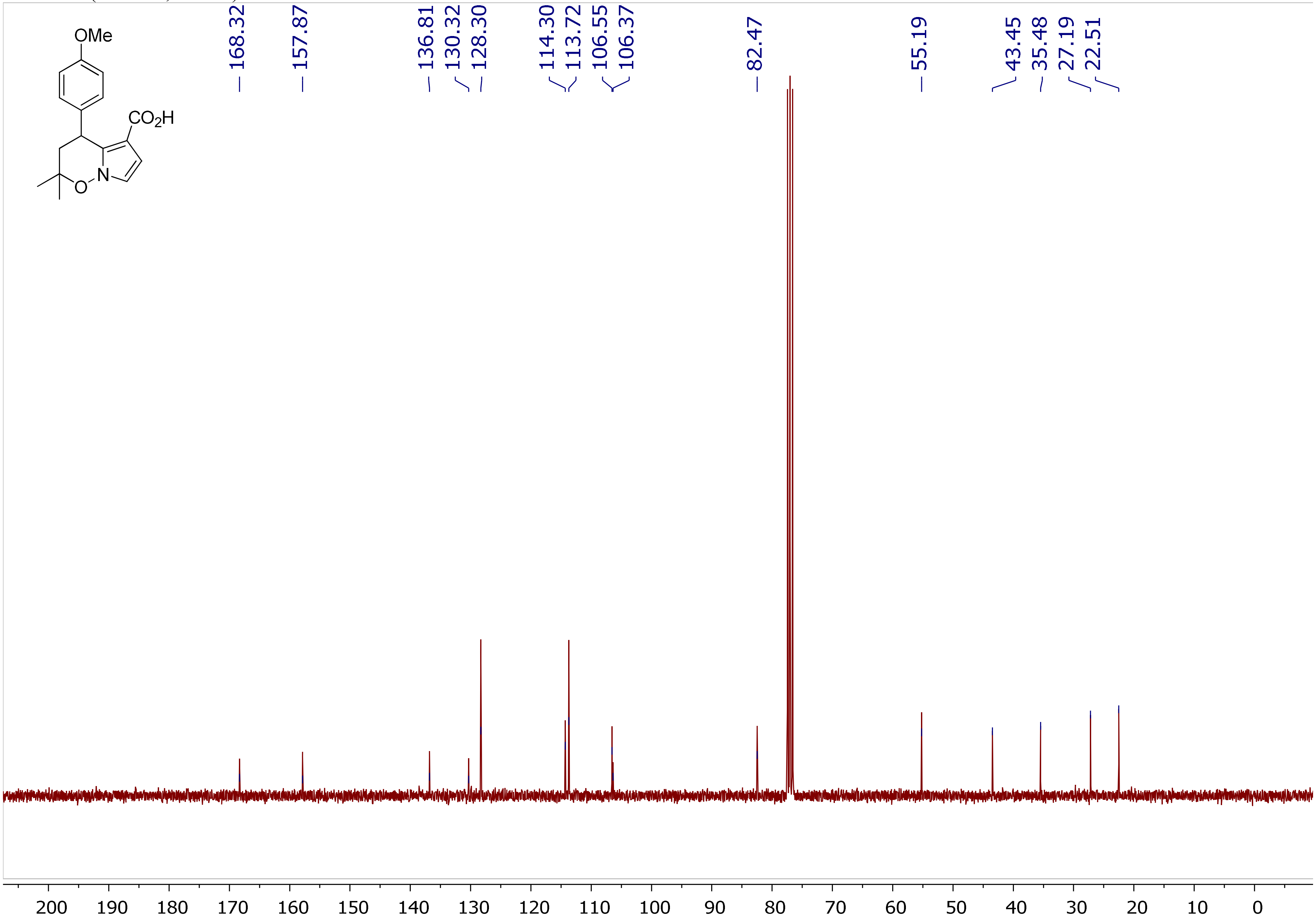

^{13}C DEPT 135 (75 MHz, $CDCl_3$)

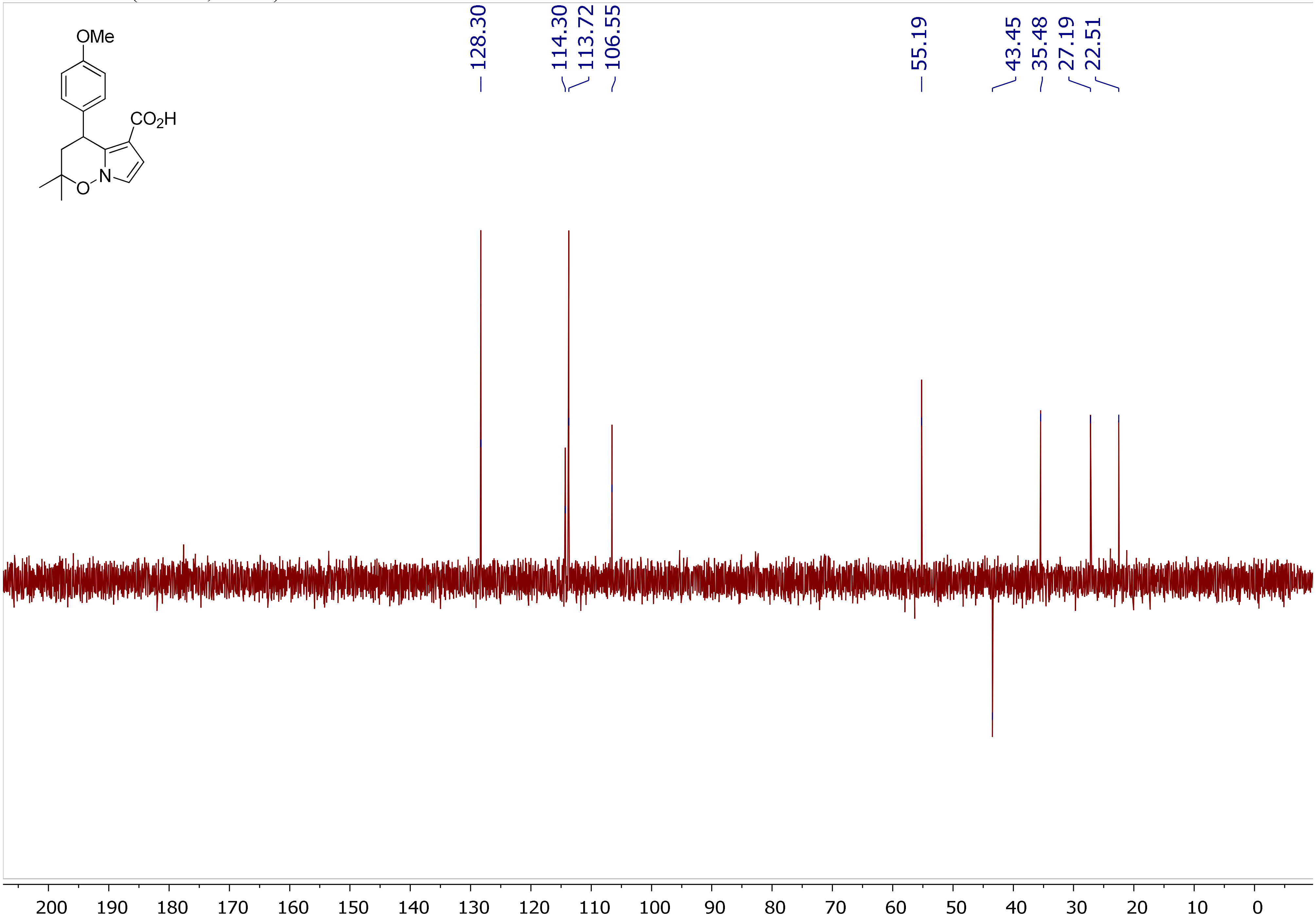

Supplement: Supplementary file 1 [file molecules-28-03025-s001.zip › molecules-2279313-supplementary.pdf]
